# Cytotoxic Alkylynols of the Sponge *Cribrochalina vasculum*: Structure, Synthetic Analogs and SAR Studies

**DOI:** 10.3390/md20040265

**Published:** 2022-04-13

**Authors:** Dimitry Kovalerchik, Ana Zovko, Petra Hååg, Adam Sierakowiak, Kristina Viktorsson, Rolf Lewensohn, Micha Ilan, Shmuel Carmeli

**Affiliations:** 1 Raymond and Beverly Sackler Faculty of Exact Sciences, School of Chemistry, Tel Aviv University, Tel Aviv 69978, Israel; kovaler310@gmail.com; 2 Department of Oncology-Pathology, Karolinska Institute, SE-171 64 Solna, Sweden; zovko.ana@gmail.com (A.Z.); petra.haag@ki.se (P.H.); adam.sie.se@gmail.com (A.S.); kristina.viktorsson@ki.se (K.V.); rolf.lewensohn@ki.se (R.L.); 3 Theme Cancer, Medical Unit Head and Neck, Lung and Skin Tumors, Thoracic Oncology Center, Karolinska University Hospital, SE-171 64 Solna, Sweden; 4 Department of Zoology, George S. Wise Faculty of Life Sciences, Tel Aviv University, Tel Aviv 69978, Israel; milan@tauex.tau.ac.il

**Keywords:** sponge, natural product, *Cribrochalina vasculum*, alkylynols, cytotoxicity, non-small cell lung carcinoma

## Abstract

A series of twenty-three linear and branched chain mono acetylene lipids were isolated from the Caribbean Sea sponge *Cribrochalina vasculum*. Seventeen of the compounds, **1–17**, are new, while six, **18–23**, were previously characterized from the same sponge. Some of the new acetylene-3-hydroxy alkanes **1**, **6**, **7**, **8**, **10** were tested for selective cytotoxicity in non-small cell lung carcinoma (NSCLC) cells over WI-38 normal diploid lung fibroblasts. Compound **7**, presented clear tumor selective activity while, **1** and **8**, showed selectivity at lower doses and **6** and **10**, were not active towards NSCLC cells at all. The earlier reported selective cytotoxicity of some acetylene-3-hydroxy alkanes (***scal*-18** and **23**), in NSCLC cells and/or other tumor cell types were also confirmed for **19**, **20** and **22**. To further study the structure activity relationships (SAR) of this group of compounds, we synthesized several derivatives of acetylene-3-hydroxy alkanes, *rac*-**18**, *scal-S*-**18**, *R*-**18**, *rac*-**27**, *rac*-**32**, *R*-**32**, *S*-**32**, *rac*-**33**, *rac*-**41**, *rac*-**42**, *rac*-**43**, *rac*-**45**, *rac*-**48** and *rac*-**49**, along with other 3-substituted derivatives, *rac*-**35**, *rac*-**36**, *rac*-**37**, *rac*-**38**, *rac*-**39** and *rac*-**40**, and assessed their cytotoxic activity against NSCLC cells and diploid fibroblasts. SAR studies revealed that the alcohol moiety at position 3 and its absolute *R* configuration both were essential for the tumor cell line selective activity while for its cytotoxic magnitude the alkyl chain length and branching were of less significance.

## 1. Introduction

Acetylene containing lipids are produced by a wide variety of plant species, higher fungi, marine algae and marine invertebrates [1]. Many of these compounds are reported to exhibit interesting biochemical activities including anti-tumor, anti-bacterial, anti-fungal, phototoxic and additional chemical and medicinal properties [2,3]. One of the most pivotal sources of acetylene containing metabolites are sponges (Porifera). More than 400 acetylene derived compounds, with cytotoxicity to eucaryotic cells and antimicrobial activities, have been isolated from selected genera of sponges, belonging to the order *Haplosclerida*, or sponges related to the genus *Petrosia* (i.e., *Petrosia*, *Xestospongia*, *Callyspongia*) until 2015 [1]. These include short chain (up C_15_) alkylynenes, such as petroraspailyne A1 [4], symmetric polyacetylene, such as callydiyne [5], halogenated polyacetylenic acids, such as xestospongic acid [6], medium chain (up to C_30_) poly acetylene alkylenols, such as siphonodiol [7], and long chain (up to C_48_), such as osirisyne A [8]. Earlier studies of the marine sponge *Cribrochalina vasculum* revealed a group of the alkyl-4*E*-ene-1-yn-3-ols (i.e., compounds **18**, 16-Me-**18**, **19**, **21**, and **22**) with cytotoxic activity against P388 mouse leukemia cell line and immunosuppressive activity in MLR (mixed lymphocyte reaction) assays [9], 3*R*-alkyl-4*E*-ene-1-yn-3-ols (*i.e.,* compounds 16-Me-**18**, **19**, **21**, **23**, 3*R*-16-methyleicos-1-yn-3-ol, 3*R*-19-methyleicos-1-yn-3-ol, and docosa-3*E*,15*Z*-dien-1-yne) with brine shrimp toxicity [10], and 3*S*-alkyl-4*E*-ene-1-yn-3-ols (i.e., compounds **18**, 16-Me-**18**, **19**, **20**, **21**, **22**, **23**, and 5*S*-16-Me of **13**) with selective cytotoxicity against H-522 non-small cell lung carcinoma (NSCLC) and IGROV-1 ovarian carcinoma (Ovca.) cell lines [11]. However, very few new alkylynols were described in recent years [12,13,14]. The cytotoxic activity of this group of compounds has drawn the attention of synthetic chemists who synthesized several of these sponge metabolites [15,16]. During our screening for putative anti-tumor compounds from marine sponges, we isolated 17 new (compounds **1–17**) and six known (compounds **18–23**) derivatives of this family of acetylene lipids, some of which we earlier reported (***Scal*-18**, **23**) to possess cytotoxic activity in NSCLC, small cell lung carcinoma (SCLC) and Ovca cell lines, but not in WI-38 diploid fibroblasts isolated from the lung [17,18]. In order to further study these metabolites, we synthesized 19 additional derivatives, some based on **18**, and studied their cytotoxic effects in an NSCLC cell line, U-1810 and in WI-38 diploid fibroblasts.

## 2. Results and Discussion 

**Figure 1:**
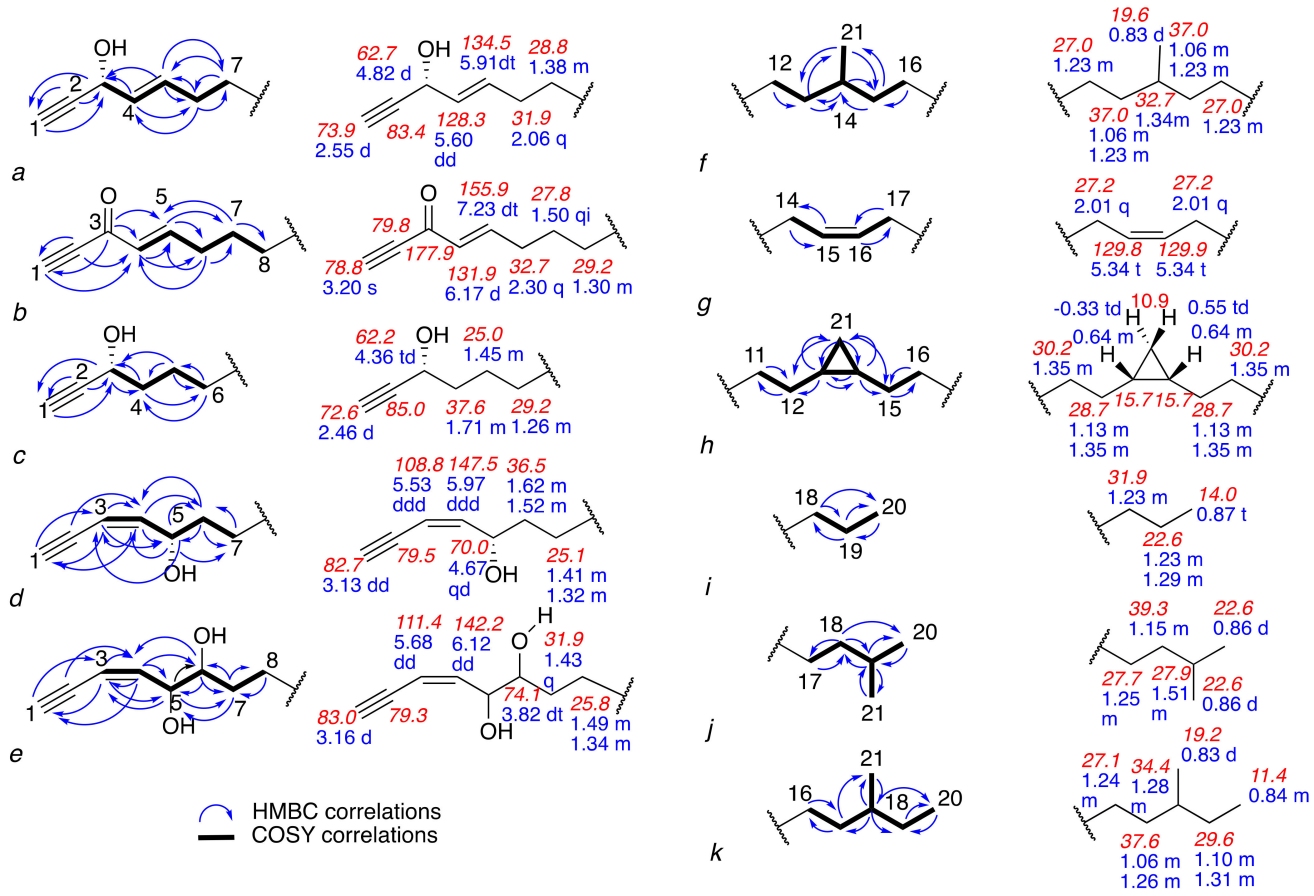
Common substructures of compounds **1–23** with characteristic ^1^H and ^13^C chemical shifts and the COSY and HMBC correlations that aloud their elucidation.

**Figure 2:**
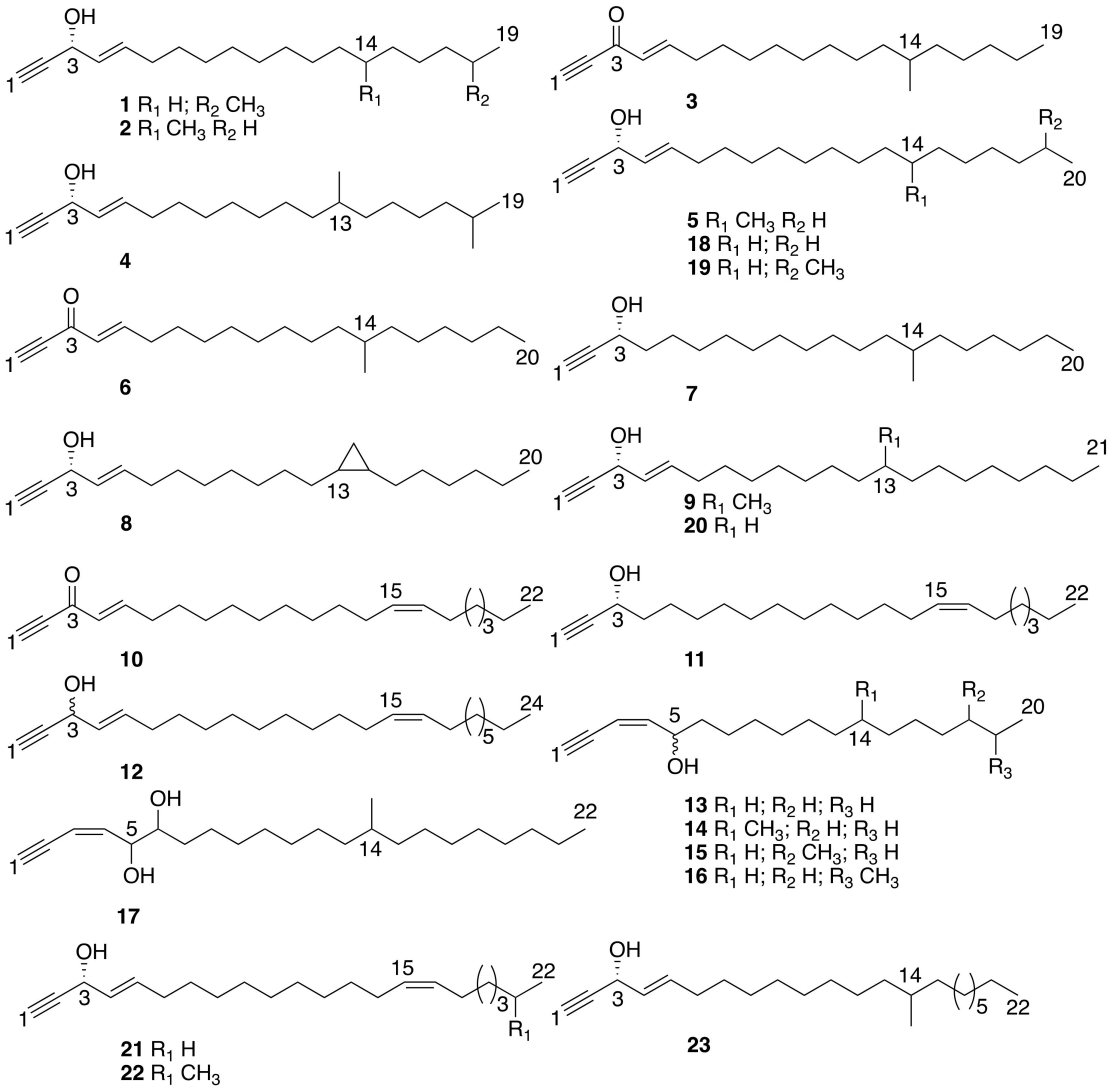
Structure of the metabolites isolated from *C. vasculum* sponge collected in Key Largo, Florida.

**Figure 3:**
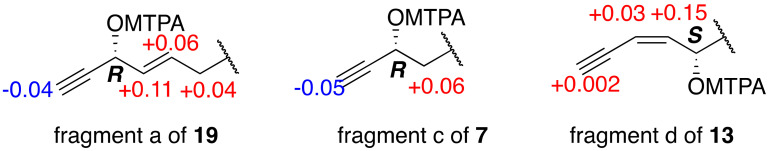
Mosher method [19] Δδ values.

**Figure 4:**
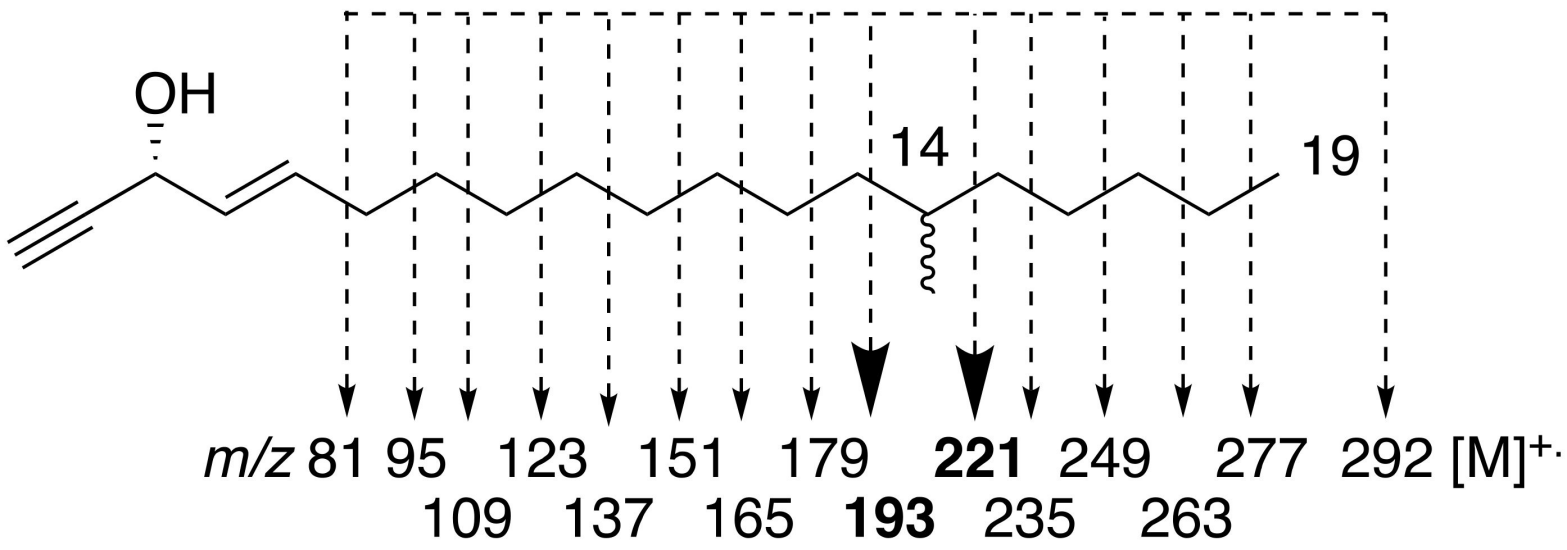
Fragmentation pattern of compound **2** in EIMS.

The crude extract of freeze-dried *Cribrochalina vasculum* material was separated on a reversed phase flash column, followed by Sephadex LH-20 gel filtration and repeated reversed phase HPLC separations guided by cytotoxicity bioassay to afford twenty-three pure compounds (**1–23**, Figure 1). We earlier reported on cytotoxicity of **18** (i.e., *scal*-**18**) and **23**, in NSCLC cells which involved action on the insulin like growth factor receptor (IGF-1R) and inhibition of downstream proliferative signaling circuits [17,18]. The compounds could be grouped into five clusters based on the structure of the acetylene end of the molecules (substructures a–e in Figure 2) while the rest of their chains varied in length (19 to 24 carbons), saturation (15*Z*), point of methylation (at carbons 13, 14, 18, 19, and 21), and in a single case also in presenting a cyclopropane ring (**8**). The alkyl-4*E*-ene-1-yn-3-ols, compounds, **1**, **2**, **4**, **5**, **8**, **9**, **18**, **19**, **20**, **21**, **22** and **23** (Figure 1) were isolated as scalemic mixtures based on a comparison of their optical rotations with those of **18** and **23**, for which the enantiomeric excess at C-3 was determined by the modified Mosher method [19]. The alkyl-4*E*-ene-1-yn-3-ol, **12** and the alkyl-3*Z*-ne-1-yn-5-ols, **13–16** (zero optical rotation), were isolated as racemic mixtures and the alkyl-1-yn-3-ols **7** and **11** were isolated as pure 3*R*-enantiomers, based on the results of the modified Mosher method [19].

(3*R*)-18-Methylnonadec-(4*E*)-en-1-yn-3-ol (**1**) presented an HRAPGCMS pseudomolecular ion, [M + H]^+^, at *m/z* 293.2848 consistent with the molecular formula C_20_H_36_O and three degrees of unsaturation. The NMR data (Table S1 in Supporting Material) indicated the presence of a terminal acetylene, a secondary alcohol, a double bond, an aliphatic chain and terminal isopropyl moieties. Its fragments, *a* (C-1 to C-7) and *j* (C-16 to C-20) were deduced based on COSY, HSQC and HMBC correlations (Figure 2). The two fragments are connected through an aliphatic chain whose length is deduced from the molecular formula of **1**. The *E*-configuration of the 4,5-double bond was inferred from the 15.2 Hz coupling constant of H-4 and H-5. The 3*R*-absolute configuration of the chiral center of **1** was based on its negative optical rotation, which was similar to those of compounds **18** and **23** [17], for which the 3*R*-configurations were obtained by the modified Mosher method [19] (Figure 3, 55 and 36 enantiomeric excesses, respectively). Based on these arguments the structure of compound **1** was determined to be a scalemic mixture in which (3*R*)-18-methylnonadec-(4*E*)- en-1-yn-3-ol was the major enantiomer.

(3*R*)-14-Methylnonadec-(4*E*)-en-1-yn-3-ol (**2**) presented similar MS pseudomolecular ion (*m/z* 293.2862 [M + H]^+^) and molecular formula (C_20_H_36_O) to those of **1**. The NMR data (Table S2) revealed similarity to that of **1** but indicated that the methyl branching was in the middle of the chain in contrast to **1** where it localized to the end of the chain. Fragments *a*, *f* (C-12 to C-16) and *i* (C-18 to C-19), were assigned based on COSY, HSQC and HMBC correlations (Figure 2). The three fragments were connected through the remaining methylene groups. The methyl branching site (partial structure *f*) could not be ascertained by NMR analysis, due to the overlapping signals in the aliphatic region, but rather deduced from the fragmentation patterns of the EIMS data (Figure 4) [20]. The EIMS spectrum of compound **2** presented two key fragments at *m/z* 193 and 221 corresponding to the fragmentation before and after the methyl group (Figure S14), which along with the absence of the ion at *m/z* 207, securely suggests that the methyl group is connected to C-14. Based on similar considerations as for **1**, the structure of **2** was assigned as a mixture of enantiomers at C-3, where the (3*R*)-14-methylnonadec-(4*E*)-en-1-yn-3-ol enantiomer was the major one. The absolute configuration of C-14 was not determined.

14-Methylnonadec-(4*E*)-en-1-yn-3-one (**3**) presented a pseudomolecular ion, [M + H]^+^, in the HRAPGCMS at *m/z* 291.2680 corresponding to the molecular formula of C_20_H_34_O and four degrees of unsaturation. Comparison of the NMR data of **3** (Table S3) with those of **2** (Table S2) revealed that **3**, did not present secondary alcohol protons and carbon, but rather a doubly conjugated ketone (δ 177.9, C-3) with a terminal acetylene (δ 3.20 s, H-1; 78.8 CH, C-1; 79.8 C, C-2) and disubstituted *E*-olefin (δ 6.17 d, *J* = 16.0 Hz, H-4; 7.23 dt, *J* = 16.0, 7.0 Hz, H-5; 2.30 q, *J* = 7.0 H, H_2_-6; 131.4 CH, C-4; 156.0 CH, C-5; 32.7 CH_2_, C-6). The rest of the proton and carbon chemical shifts of **3** were similar to those of **2**. COSY, HSQC and HMBC correlations (Table S3) allowed the assignment of fragments *b*, *f* and *i*. The later fragments, of **3**, could be assembled by comparison of the NMR and EIMS [20] (Figure S18) of **3** with those of **2**. Thus, the structure of **3** was assigned as 14-methylnonadec-(4*E*)-en-1-yn-3-one. The absolute configuration of C-14 was not determined.

(3*R*)-13,18-Dimethylnonadec-(4*E*)-en-1-yn-3-ol (**4**), a colorless oil, presented a HRAPGCMS pseudomolecular ion, [M + H]^+^, at *m/z* 307.3003 corresponding to C_21_H_38_O and three degrees of unsaturation. Analysis of the NMR data of **4** (Table S4) revealed the presence of fragments *a*, *f* and *j*. Furthermore, fragments *f* and *j* could be connected through HMBC correlations of C-16 with H-14a, H-14b, H_2_-15, H_2_-17 and H-18 and by comparison of the carbon chemical shifts of **4** with those calculated by ChemDraw (Figure S22) and based on the fragmentation in the EIMS (Figure S23). The negative optical rotation of **4** suggested that the absolute configuration of its major C-3 enantiomer is, 3*R*. Based on these arguments the structure of compound **4** was assigned as (3*R*)-13,18-dimethylnonadec-(4*E*)-en-1-yn-3-ol. The absolute configuration of C-13 was not determined.

(3*R*)-14-methylicos-(4*E*)-en-1-yn-3-ol (**5**) (*m/z* 307.3027, [M + H]^+^, corresponding to the molecular formula C_21_H_38_O) and 14-methylicos-(4*E*)-en-1-yn-3-one (**6**) (*m/z* 305.2870, [M + H]^+^, corresponding to the molecular formula C_21_H_36_O) presented NMR (Tables S5 and S6, respectively), EIMS [20] (Figures S30 and S34, respectively), and optical rotation data indicating that they are one-carbon homologs of **2** and **3**, respectively. Full data analysis of analogs with **2** and **3**, established the structure of major C-3 enantiomers **5** as (3*R*)-14-methylicos-(4*E*)-en-1-yn-3-ol and that of **6** as 14-methylicos-(4*E*)-en-1-yn-3-one. The absolute configuration of C-14 was not determined.

(3*R*)-14-Methylicos-1-yn-3-ol (**7**), an amorphous white solid, displayed a pseudomolecular ion, [M + H]^+^, at *m/z* 309.3155 in the HRAPGCMS, corresponding to the molecular formula of C_21_H_40_O and three degrees of unsaturation. The EIMS spectrum, of **7**, presented a weak molecular ion and relatively strong [M – H_2_O]^+^ ion, in contrast to the relatively strong molecular ions revealed in the EIMS spectra of **1–6**. The ^1^H and ^13^C NMR data of **7** suggested the existence of a terminal acetylene (δ 2.46 d, *J* = 2.0 Hz, H-1; 72.6 CH, C-1; 85.0 C, C-2) and its adjacent alcohol (δ 4.36 td, *J* = 6.5, 2.0 Hz, H-3; 62.2 CH, C-3), a terminal methyl (δ 0.87 t, *J* = 7.0 Hz, H_3_-20; 14.1 CH_3_, C-20) and a doublet methyl (δ 0.83 d, *J* = 7.0 Hz, H_3_-21; 20.1 CH_3_, C-21). Fragments *c* (C-1 to C-6), f (C-12 to C-16) and *i* (C-18 to C-20) were assigned based on COSY, HSQC and HMBC correlations (Figure 2). The chain length (20 carbons) and the position where the doublet methyl (CH_3_-21) is attached to the chain (C-14) were deduced from the molecular formula and the EIMS fragmentation pattern [20] (Figure S42) of **7**, respectively. The 3*R*-configuration of **7** was determined by the modified Mosher method [19] (Figure 3). Thus, the structure of **7** was assigned as (3*R*)-14-methylicos-1-yn-3-ol. The absolute configuration of C-14 was not determined.

(3*R*,*E*)-12-*cis*-(2-Hexylcyclopropyl)dodec-4-en-1-yn-3-ol (**8**), a colorless oil, presented a HRAPGCMS pseudomolecular ion, [M + H]^+^, at *m/z* 305.2863, corresponding to the molecular formula C_21_H_36_O and four degrees of unsaturation. The ^1^H NMR spectrum of **8,** presented signals indicative of a *cis*-cyclopropane moiety (δ 0.64 m, H-13 and H-14; 0.55 td, *J* = 8.2, 4.0 Hz, H-21a; −0.33 td, *J* = 4.9, 4.0 Hz, H-21b) [21], in addition to a terminal methyl group, chain of methylenes and a (4*E*)-ene-3-ol-1-yne spin system similar to that of **18**. COSY, HSQC and HMBC correlations (Table S8) established fragments, *a* (C-1 to C-7), *h* (C-11 to C-16) and *i* (C-18 to C-20) but failed to establish their connection. The above presented fragments and carbon chemical shift calculated by ChemDraw (Figure S50) suggested that the cyclopropane ring is situated between C-13 and C-15 but did not allow conclusive determination of its position. The fragmentations in the EIMS presented single ion fragments down to *m/z* 201 and multiple (m, m-2, m-4) ions from *m/z* 191 down to *m/z* 51 (Figure S51). The ions at *m/z* 219 and 201 suggest a loss of a C_6_H_13_ radical from the molecular ion or [M − H_2_O]^+^ ion, respectively. The next three multiple ions (each composed of m, m-2, m-4 ions, *m/z* 181, 189, 187; 177, 175, 173 and 163, 161, 159) were weaker in intensity than the ions on both sides (Figure S51). This might be explained by three possible bond cleavages of a cyclopropane fused to C-13 and C-14 of **8** (Figure S53a,b) and derived from its molecular ion or [M − H_2_O]^+^ ion. Based on these arguments the cyclopropane ring was assigned to positions 13 and 14 [22]. The negative sign of the optical rotation of **8** suggests a 3*R* absolute configuration of its chiral center similar to the above-described compounds. Thus, compound **8** was assigned as (3*R*,*E*)-12-*cis*-(2-hexylcyclopropyl)dodec-4-en-1-yn-3-ol.

(3*R*)-13-Methylhenicos-(4*E*)-en-1-yn-3-ol (**9**) was isolated as a colorless oil that presented an HRAPGCMS pseudomolecular ion, [M + H]^+^, at *m/z* 321.3122 corresponding to the molecular formula C_22_H_40_O and three degrees of unsaturation. Its NMR data (Table S9) were similar to that of **2** indicating that the methyl branching was in the middle of the chain. Fragments *a*, *f* (C-12 to C-14) and *i* (C-19 to C-21) were assigned based on COSY, HSQC and HMBC correlations (Figure 2). The three fragments should thus, be connected through the remaining methylene groups. The methyl branching site (partial structure *f*) could not be ascertained by NMR analysis, due to the overlapping signals in the aliphatic region, but could rather be deduced from the fragmentation patterns of the EIMS data [20]. Compound **9** EIMS spectrum presented two sets of consecutive chain fragmentations derived from the molecular ion and water elimination product ion which produced the key fragments at *m/z* 207 and 179, and 189 and 161 corresponding to the fragmentation before and after the methyl group (Figure S56). The negative sign of the optical rotation of **9** suggested a 3*R* absolute configuration of its chiral center similar to the above-described compounds. Thus, compound **9** was assigned as (3*R*)-13-methylhenicos-(4*E*)-en-1-yn-3-ol.

Docos-(4*E*,15*Z*)-dien-1-yn-3-one (**10**) was isolated as a colorless oil presenting an EIMS molecular ion at *m/z* 316 corresponding to the molecular formula C_22_H_36_O and five degrees of unsaturation. Its NMR data revealed a double conjugated ketone (δ 177.9, C-3), a terminal conjugated acetylene (δ 3.20 s, H-1; 78.8 CH, C-1; 79.8 C, C-2), a conjugated *E*-double bond (δ 6.17 d, *J* = 16.0 Hz, H-4; 7.23 dt, *J* = 16.0, 7.0 Hz, H-5; 2.30 q, *J* = 7.0 H, H_2_-6; 131.4 CH, C-4; 156.0 CH, C-5; 32.7 CH_2_, C-6), a *Z*-double bond (δ 5.35 (2H, bs, H-15 & 16; 129.8 CH, C-15; 129.9 CH, C-16), and a terminal methyl group (δ 0.88 t, *J* = 6.7 Hz, H_3_-22) 14.1 (CH_3_, C-22). Analyses of the COSY, HSQC and HMBC correlations (Table S10) established fragments *b* (C-1 to C-7), *g* (C-14 to C-17) and *i* (C-20 to C-22) but failed to bring about their connection. The 2D NMR correlations (Table S10) did not allow an unequivocal connection between the three fragments. However, LC-MS analysis of the periodate-permanganate oxidation products of **10** (Figure S64), identified undecadioic acid as the heaviest product thus establishing the position of the *Z*-double bond between carbons 15 and 16, and the structure of compound **10** as docos-(4*E*,15*Z*)-dien-1-yn-3-one.

(3*R*)-Docos-(15*Z*)-en-1-yn-3-ol (**11**), a colorless oil, presented an EIGCMS molecular ion, [M]^+^, at *m/z* 316 corresponding to the molecular formula C_22_H_36_O and three degrees of unsaturation. Its NMR data revealed the presence of terminal acetylene (δ 2.45 d, *J* = 2.0 Hz, H-1; 72.7 CH, C-1; 85.0 C, C-2) and its adjacent alcohol (δ 4.36 td, *J* = 6.5, 2.0 Hz, H-3; 62.3 CH, C-3), a *Z*-double bond (δ 5.34 m, H-15, H-16; 129.9 2 × CH, C-15, C-16), and a terminal methyl (δ 0.87 t, *J* = 7.0 Hz, H_3_-20; 14.1 CH_3_, C-22). Analysis of the 2D NMR (HSQC, HMBC and COSY) data of **11** (Table S11) revealed the three fragments *d* (C-1 to C-6), *g* (C-14 to C-17) and *i* (C-20 to C-22). The position of the 15,16-*Z*-double bond was proposed in analogy to the position of the *Z*-double bond in compounds **10**, **12**, **21** and **22**. The absolute configuration of the C-3 was determined as *R* by the modified Mosher method [19] establishing the structure of **11** as (3*R*)-docos-(15*Z*)-en-1-yn-3-ol.

*rac*-Tetracos-(4*E*,15*Z*)-dien-1-yn-3-ol (**12**), a colorless oil, presented an EIMS molecular ion, M+, at *m/z* 346 corresponding to the molecular formula C_24_H_42_O and four degrees of unsaturation. It presented NMR data (Table S12) almost identical (fragments *a*, C-1 to C-7, *g*, C-13 to C-17, and *i*, C-18 to C-20, except of the 24H integration of the huge methylene signal between 1.22 and 1.32 ppm) to those of the known **21** [10,11,15], (which was also isolated in this study), suggesting extra two methylenes in **12** relative to **21**. The position of the 15,16-*Z*-double bond was determined as for **10** by a combination of oxidation and LCMS determination of the undecadioic acid (Figure S75) [19]. Thus, the structure of compound **12** was determined to be *rac*-tetracos-(4*E*,15*Z*)-dien-1-yn-3-ol.

*rac*-Icos-(3*Z*)-en-1-yn-5-ol (13) was isolated as colorless oil which presented a HRAPGCMS pseudomolecular ion, [M + H]^+^, at *m/z* 293.2831 corresponding to the molecular formula C_20_H_36_O and three degrees of unsaturation. The NMR spectra of **13** (Table S13) differed from the spectra of **1–12** (Tables S1–S12) in presenting a conjugated acetylene system (fragment *d*, δ 3.13 d, *J* = 1.5 Hz, H-1; 82.7 CH, C-1; 79.5 C, C-2; 5.52 dd, *J* = 11.0, 1.5 Hz, H-3; 108.8 CH, C-3; 5.98 dd, *J* = 11.0, 8.9 Hz, H-4; 147.5 CH, C-4), allylic alcohol (fragment *d*, δ 4.67 q, *J* = 8.5 Hz, H-5; 70.0 CH, C-5), a long aliphatic chain and a terminal methyl group (fragment *i*, δ 0.88 t, *J* = 7.0 Hz, H_3_-20; 14.1 CH_3_, C-20). The structure of fragment *d* of **13** was established based on the correlations of its 2D NMR spectra (Table S13) and the length of the alkyl chain based on its molecular formula calculated from the HRMS measurements, establishing the structure of **13** as *rac*-icos-(3*Z*)-en-1-yn-5-ol.

*rac*-14-Methylicos-(3*Z*)-en-1-yn-5-ol (**14**), was isolated as a colorless oil with an HRAPGCMS pseudomolecular ion, [M + H]^+^, at *m/z* 307.3017 corresponding to a molecular formula of C_21_H_38_O and three degrees of unsaturation. Its NMR data (Table S14) closely resembled those of **13**, except for the additional doublet methyl signal (δ 0.83 d, *J* = 6.5 Hz, H_3_-21; 19.7 CH_3_, C-21) that accounted for the 14 mass-units difference in the mass spectrum (relative to **13**). Analyses of the COSY, HSQC and HMBC correlations (Table S14) revealed fragments *d* (C-1 to C-7), *f* (C-12 to C-16) and *i* (C-18 to C-20) (Figure 1) but failed to demonstrate their connection. The chain length (20 carbons) and the position where the doublet methyl (CH_3_-21) was attached to the chain (C-14) were deduced from the molecular formula and the EIMS fragmentation pattern [20] (Figure S90) of **14**, respectively. Based on these arguments the structure of **14** was established as *rac*-14-methylicos-(3*Z*)-en-1-yn-5-ol.

*rac*-18-Methylicos-(3*Z*)-en-1-yn-5-ol (**15**) and (5*S*)-19-methylicos-(3*Z*)-en-1-yn-5-ol **(16**) were isolated as 2:5 inseparable mixture that presented a single HRAPGCMS pseudomolecular ion, [M + H]^+^, at *m/z* 307.3026 corresponding to a molecular formula of C_21_H_38_O and three degrees of unsaturation. Their NMR spectra (Tables S15 and S16), which resembled those of **13** and **14**, were superimposed on one of each other except for signals of the chain end, for which, **15** presented signals corresponding isobutyl chain end (δ 1.28 m, H-18; 34.4 CH, C-18; 1.31 m, 1.10 m, H_2_-19; 29.6 CH_2_, C-19; 0.84 t, *J* = 6.6 Hz, H_3_-20; 11.4 CH_3_, C-20; 0.83 d, *J* = 6.2 Hz, H_3_-21; 19.2 CH_3_, C-21), while **16** showed signals matching an isopropyl chain end (δ 1.13 m, H_2_-18; 39.0 CH_2_, C-18; 1.50 m, H-19; 27.9 CH, C-19; 0.85 d, *J* = 6.5 Hz, H_3_-20 and 21; 22.6 2 × CH_3_, C-20 and 21), similar to that of **19**. The chain length (20 carbons) of **15** and **16** were deduced from a molecular formula based on the results of the HRMS measurements, thus establishing the structure of **15** as *rac*-18-methylicos-(3*Z*)-en-1-yn-5-ol and that of **16** as *rac*-19-methylicos-(3*Z*)-en-1-yn-5-ol.

14-Methyldocos-(3*Z*)-en-1-yn-5,6-diol (**17**) was isolated as an amorphous white solid presenting an EIMS molecular ion, M+, at *m/z* 350 corresponding to a molecular formula of C_23_H_42_O_2_ and three degrees of unsaturation. Its NMR data resembled those of **14** except for an extra-oxygenated methine (δ 3.82 dt, *J* = 6.5, 3.0 Hz, H-6; 74.1 CH, C-6). COSY, HSQC and HMBC correlations (Table S17) established fragments *e* (C-1 to C-8), *f* (C-12 to C-16) and *i* (C-20 to C-22) but failed to demonstrate their connection. The chain length (22 carbons) and the position where the doublet methyl (CH_3_-23) is attached to the chain (C-14) were deduced from the molecular formula and the EIMS fragmentation pattern *m/z* 219 and 191, and 201 and 173 (Figure S107) of **17**, respectively [20]. The absolute configuration of C-5 and C-6 was not determined. Based on these arguments the structure of **17** was assigned as 14-methyldocos-(3*Z*)-en-1-yn-5,6-diol.

We have previously demonstrated specific cytotoxic activity for two compounds, *scal*-**18** and **23** in multiple NSCLC cell lines, and other tumor cell lines, e.g., Ovcar and SCLC as well as in diploid lung fibroblasts WI-38 [17]. Here, the NSCLC cell line U-1810 was used alongside WI-38 to reveal the selective cytotoxicity of some of the novel compounds **1**, **6–8**, **10**, **19**, **20** and **22** (Table 1, Figure S188). Results showed that the chain length and point of alkylation or additional double-bonds in the chain did not to any large extent affect the activity, while the structure around the acetylene end of the molecule had profound effects on the specific cytotoxic activity in the tested NSCLC cell line.

Compounds **1**, **7**, **8**, **18** (multiple derivatives tested), **19**, **20**, **22**, and **23** that contained fragments *a* and *c* (Figure 2) were equally active in NSCLC U-1810 cells, while those containing fragment *b*, **6** and **10**, were not active and those containing fragments *d* and *e*, **13**-**16** and **17**, were not tested, however, compounds containing fragment *d* have been previously shown to be similarly active to those containing fragment *a* [20].

**Scheme 1:**
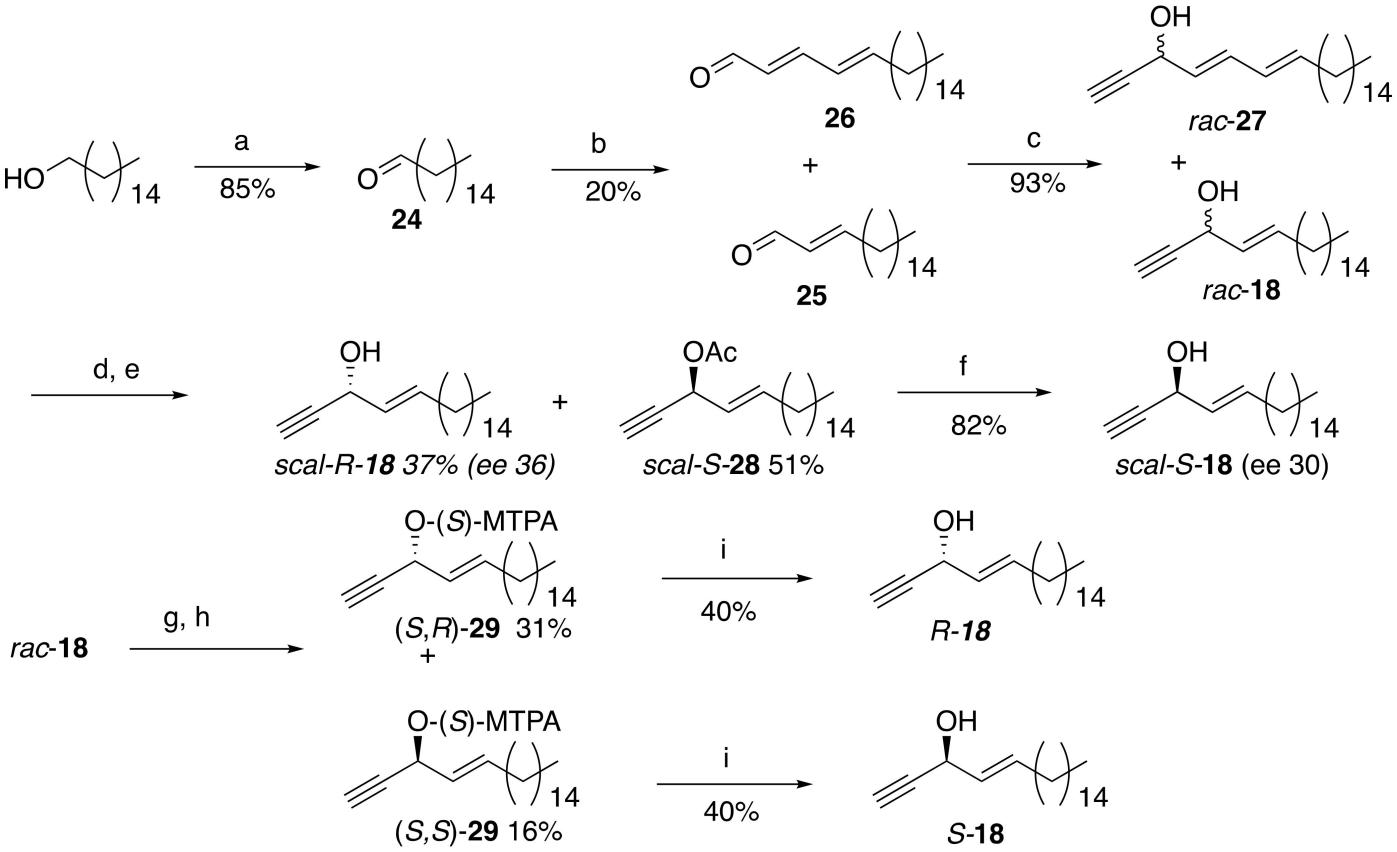
Synthetic scheme of compounds *R*-**18** and *S*-**18**. Reagents: (a) PCC, CH_2_Cl_2_, MS 4Å; (b) Ph_3_P=CHCHO, CH_2_Cl_2_; (c) HCCMgBr, THF; (d) Lipase, Vinyl acetate, Petroleum ether; (e) Chromatography; (f) K_2_CO_3_, MeOH; (g) (*R*)-MTPACl, DMAP, CH_2_Cl_2_; (h) HPLC; (i) NH_3_, MeOH, 90 °C, 3 days.

**Scheme 2:**
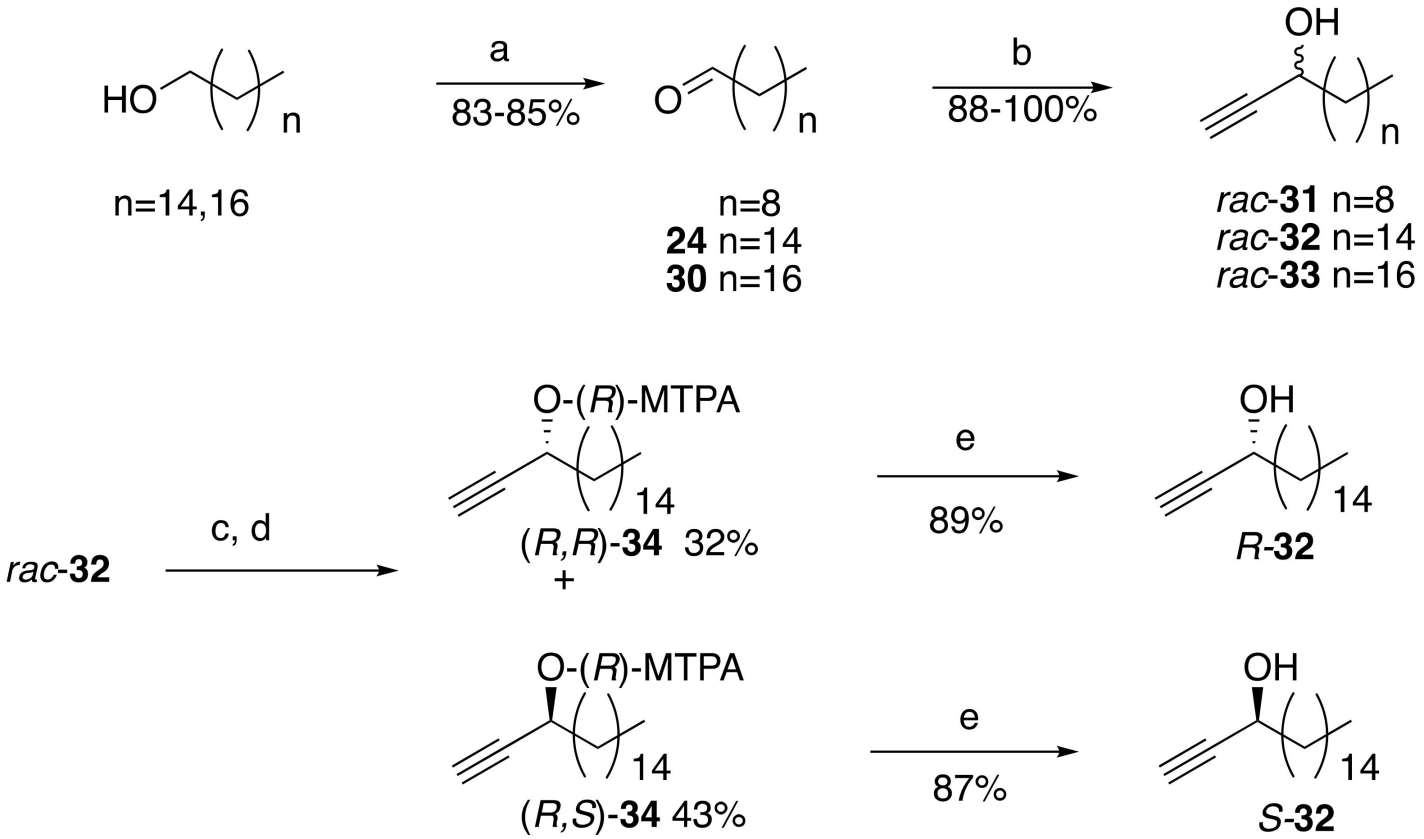
Synthetic scheme of compounds *rac*-**29**, *rac*-**30**, *R*-**30**, *S*-**30**, and *rac*-**31**. Reagents: (a) PCC, CH_2_Cl_2_, MS 4Å; (b) HCCMgBr, THF; (c) (*S*)-MTPACl, DMAP, CH_2_Cl_2_; (d) HPLC; (e) NH_3_, MeOH, 90 °C, 3 days.

In order to further analyze the cytotoxic activity and selectivity of this group of compounds we synthesized multi-gram quantities of racemic icos-(4*E*)-en-1-yn-3-ol (*rac*-**18**), (4*E*,6*E*)-docosa-4,6-dien-1-yn-3-ol (*rac*-**27**) (Scheme 1), dodec-1-yn-3-ol (*rac-***31**), octadec-1-yn-3-ol (*rac-***32**) and icos-1-yn-3-ol (*rac-***33**) (Scheme 2) by a published procedure from Gung et al. [23]. Applying enzymatic resolution to *rac-***18**, afforded scalemic mixtures of *scal-R*-**18** (ee 30) and *scal-S*-**18** (ee 36) [23]. In order to obtain the pure *S* and *R* enantiomers for the SAR analyses in tumor cells and fibroblasts, *rac*-**18** and *rac*-**32** were reacted with Mosher reagent [19] to afford the corresponding diasteromeric MTPA-esters, which in turn were separated by HPLC to yield the pure esters, (*S,R*)-**29** and (*S,S*)-**29** from *rac*-**18**, and (*R,R*)-**34** and (*R,S*)-**34** from *rac*-**32**. Hydrolysis of (*S,R*)- and (*S,S*)-**29** and (*R,R*)- and (*R,S*)-**34** afforded the enantiomeric pure *R*-**18** acetylene-3-ol lipids and *R*-**32** and *S*-**32**, respectively. We did not succeed in purifying *S*-**18** and thus, it was not tested for its cytotox- icity in NSCLC cells.

Cytotoxicity analyses of the latter derivatives established (Table 1, Figure S188) that the compound with a short chain, *rac*-**31** presents much lower toxicity to the NSCLC U-1810 cell line while, *rac*-**32**, *rac*-**33** and especially *R*-**32** were the most potent and tumor selective derivatives. Based on these results, we set to prepare some derivatives of *rac*-**32** for SAR studies. Interestingly, the byproduct, *rac*-**27**, which contains an extra ethylene moiety relative to *rac*-**18**, presented cytotoxicity similar to that of *rac*-**18** in the NSCLC U-1810 cell line, but its selective cytotoxic activity was much better as *rac*-**27** did not affect the viability of the normal lung fibroblasts WI-38 even when very high concentrations were applied (Table 1, Figure S188).

**Scheme 3:**
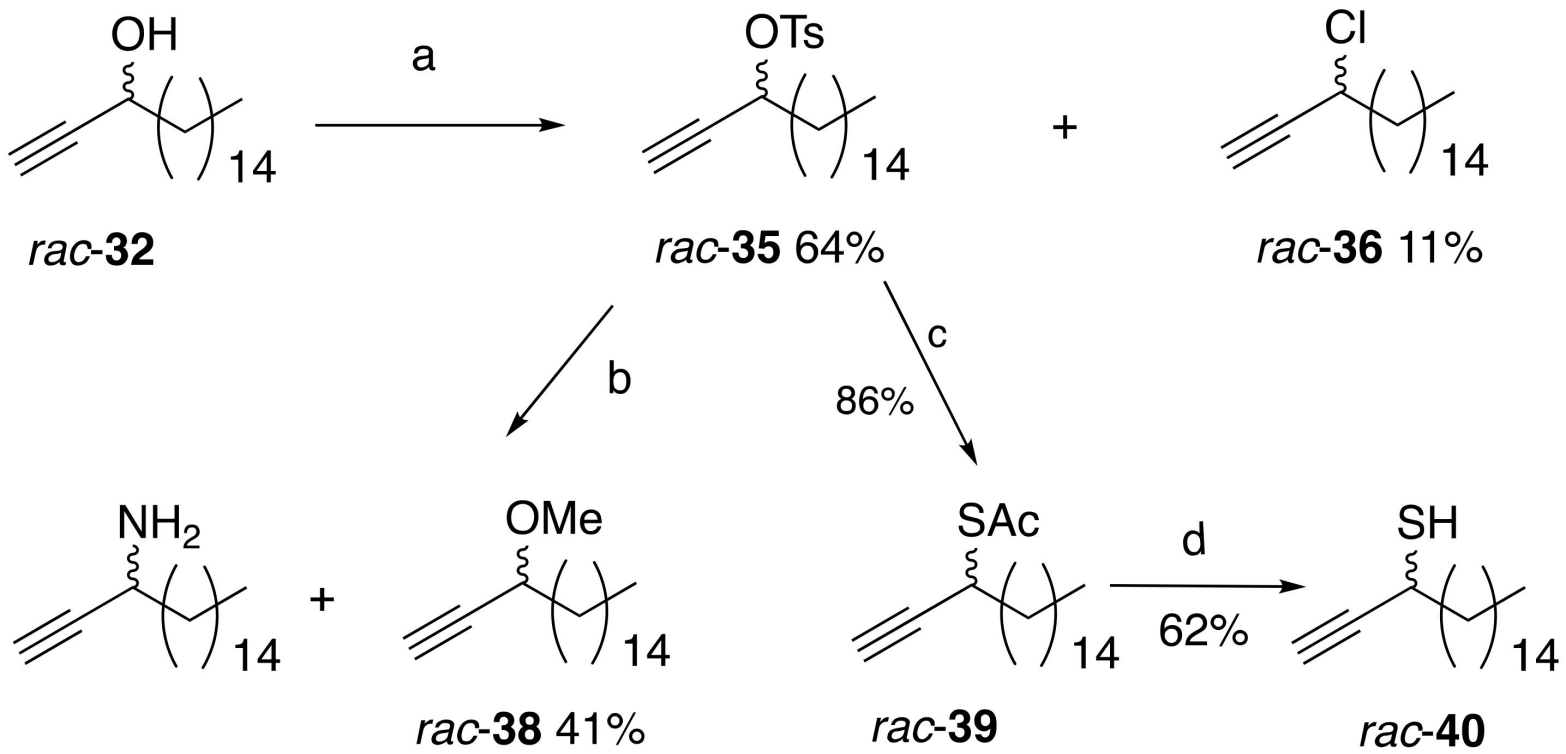
Synthetic scheme of the preparation of compounds *rac*-**35**-*rac*-**40**. Reagents: (a) TsCl, CH_2_Cl_2_, triethylamine, 3 days; (b) NH_3_, MeOH, 60^o^C, 3 h; (c) AcSH, triethylamine; (d) HCl, MeOH.

To facilitate the preparation of the amino and thiol derivatives of *rac*-**32**, it was reacted with tosylsulfonyl chloride to afford the tosylate derivative, *rac*-**35**, in moderate yield, along with the chloride byproduct, *rac*-**36** (Scheme 3) [24]. Treatment of *rac*-**35** with ammonia in methanol afforded the desired amine derivative *rac*-**37** and the methoxyl-derivative *rac*-**38**, both in moderate yields (Scheme 3). The acetylthiol derivative, *rac*-**39**, was finally produced by reaction with thioacetic acid [25] and acid hydrolysis produced the relatively unstable thiol derivative *rac*-**40**. When compounds *rac-***35**-*rac-***40** were studied for cytotoxic effects in NSCLC cells (U-1810) and lung fibroblasts (WI-38), none of these synthetic analogs inhibited NSCLC cells at concentrations comparable with those of *rac*-**32** (Table 1). In fact, some of them (*rac-***36** *rac-***38**, *rac-***39** and *rac-***40**) did not display any specific cytotoxicity (Table 1).

**Scheme 4:**
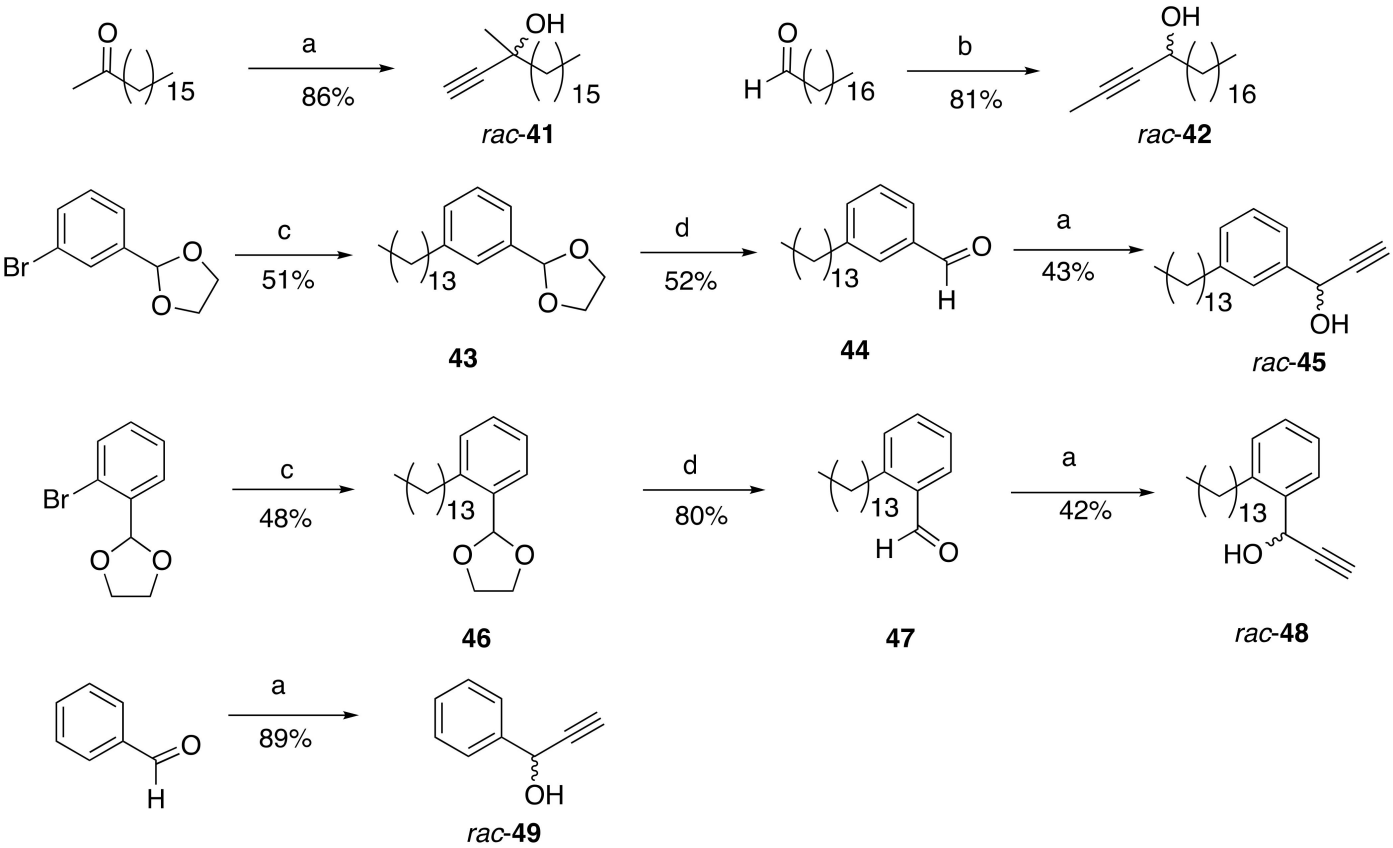
Synthetic scheme of the preparation of compounds *rac*-**41**-*rac*-**49**. Reagents: (a) HCCMgBr, THF; (b) MeCCMgBr, THF; (c) *i* Mg, THF; *ii* 1-bromotetradecane, HMPA, CuBr, THF; (d) TFA, CH_2_Cl_2_, 12 h.

Five additional derivatives, *rac-***41**, *rac-***42**, *rac-***45**, *rac-***48** and *rac-***49** were synthesized (Scheme 4) to study the influence of steric hindrance and restricted rotation next to the acetylene moiety, on the activity of the alkyl acetylene alcohols. The reaction of acetylene magnesium bromide with 2-oxo-hexadecane afforded in good yield the tertiary alcohol *rac*-**41** [23]. Palmitaldehyde was reacted with propylene magnesium bromide to produce *rac*-**42** in good yield. Coupling reaction of 2-(3-phenylmagnesiumbromide)-1,3-dioxolane and 2-(2-phenylmagnesium-bromide)-1,3-dioxolane with 1-bromotetradecane afforded the corresponding alkylphenyl dioxolanes **43** and **46**, respectively, which upon acidic hydrolysis afforded the corresponding aldehydes, **44** and **47**. Reactions of these aldehydes with ethynyl magnesium bromide resulted in the corresponding *meta*- and *ortho*-alkylphenyl propargylic alcohol derivatives *rac*-**45** and *rac*-**48** (Scheme 4). Finally, benzaldehyde was reacted with acetylene magnesium bromide in good yield to afford *rac*-**49**. When *rac-***41**, *rac-***42**, *rac-***45**, *rac-***48** and *rac-***49**, were assayed for anti-tumor effect in NSCLC U-1810 cells lower cytotoxicity was evident, of one to two orders of magnitudes, relative to *rac*-**18** and *rac*-**32** and thus these compounds were not tested further in diploid fibroblasts (Table 1). The latter results demonstrated that substitution of the proton of the carbinol methine-3, in *rac*-**32**, with a methyl group resulted in a product, *rac*-**41**, which presented two orders of magnitude less cytotoxic towards NSCLC U-1810 cells. Similar results were obtained for the substitution of the acetylene proton at position 1, in *rac*-**32**, with a methyl group, in *rac*-**42** (Table 1). Restricting the rotation further along the chain, by insertion of a phenyl ring in positions 4–6, *rac-***45**, or 4–5, *rac-***48**, similarly resulted in an order of magnitude less potent cytotoxic activity in the tested NSCLC U-1810 cells. Exclusion of the alkyl substituent from the phenyl ring, *rac**-*****49**, generated a product that did not display any anti-tumor effect in the NSCLC cells tested. A comparison of the cytotoxic activity of *rac-***27**, *rac-***45** and *rac-***48**, (Table 1) reveals that the restricted rotation imposed by the phenyl ring negatively influenced the anti-tumor potency of the resulting compounds. 

## 3. Materials and Methods

### 3.1. General Experimental Procedures 

Optical rotation values were obtained on a Jasco P-1010 polarimeter at the sodium D line (589 nm). UV spectra were recorded on an Agilent 8453 spectrophotometer. IR spectra were recorded on a Bruker Tensor 27 FT-IR instrument. NMR spectra were recorded on a Bruker Avance III Spectrometer at 500.13 MHz for ^1^H and 125.76 MHz for ^13^C and a Bruker Avance III 400 Spectrometer at 400.13 MHz for ^1^H, 100.62 MHz for ^13^C NMR, chemical shifts were referenced to TMS ^δ^H and ^δ^C = 0 ppm. DEPT, COSY-45, gTOCSY, gROESY, gHSQC, gHMQC, and gHMBC, spectra were recorded using standard Bruker pulse sequences. Low resolution mass spectra were recorded on a Waters MaldiSynapt instrument (ESI and APPI), a Waters Xevo TQD instrument (ESI), an Aviv Analytical 5975-SMB instrument (GCMS with cold EI) and Agilent Technologies GCMS5977A MDS with 7890B GC system. High resolution mass spectra were recorded on a Waters MaldiSynapt instrument (ESI and APPI) and Bruker Maxis Impact QTOF APGC instrument equipped with a Bruker SCION456 GC. HPLC separations were performed on a Merck Hitachi HPLC system (L-6200 Intelligent pump and L-4200 UV-VIS detector), a JASCO P4-2080 plus HPLC system with a Multiwavelength detector, and an Agilent 1100 Series HPLC system.

### 3.2. Biological Material

*Cribrochalina vasculum* samples (M01232-M01239), were collected in “Conch Reef Wall” Florida Keys (24°56.996′ N 80°27.223′ W) at 15 m depth. The sponge was identified as *Cribrochalina vasculum* (Lamarck, 1814) by J.R. Pawlik and M. Ilan, based on external morphology, types of spicules, and internal skeletal arrangement, which fit the species recorded descriptions [26,27]. Vouchers are deposited at the Steinhardt Museum of Natural History and National Research Center, Tel Aviv University (M1232, M1233, from 22°36.56′ N 73°38.38′ W; M1236, M1237, from 22°05.28′ N 74°32.15′ W; M1239 from 21°40.40′ N 73°50.31′ W). The cell mass was frozen and lyophilized.

### 3.3. Isolation Procedure

The freeze-dried sample (206 g) was extracted with a 45:45:10 mixture of EtOAc/MeOH/H_2_O at room temperature three times. The crude extract (43 g) was evaporated to dryness and separated, in 11 portions, on an ODS (YMC-GEL, 120A, 4.4 × 6.4 cm) flash column with increasing amounts of MeOH in water giving 11 fractions. The cytotoxic (revealed by screening NSCLC U-1810 cells) fraction 10 (9:1 MeOH/H_2_O) was further separated twice on Sephadex LH-20 column eluted with 1:1 chloroform/methanol solution. The resulting fractions were separated on HPLC column (YMC- Pack C8, 5 µm, 250 × 20 mm, 40% MeCN: 51% MeOH, 9% H_2_O) resulting in 14 fractions, two of which, fractions 3 and 10 were found to be pure **18** that eluted from the column at 40.9 min (70.1 mg, 0.034% yield of dry sponge weight) and **23** that eluted at 65.9 min (109.0 mg, 0.053% yield), respectively. The rest of the fractions were further separated on a YMC Pack ODS-A HPLC column (250 × 20 mm) eluted with different mixtures of MeCN, MeOH and H_2_O. Fraction 2 was eluted from the column with 9:1 MeCN/MeOH to give at 39.3 min, **1** (4.6 mg, 0.0022% yield), at 63.8 min, **2** (3.8 mg, 0.0018% yield), at 47.4 min **6** (0.6 mg, 0.00029% yield), at 58.0 min, **7** (9.8 mg, 0.0048% yield), at 36.9 min, **8** (4.9 mg, 0.0024% yield), at 54.1 min, **9** (4.7 mg, 0.0023% yield), at 53.3 min, **10** (6.4 mg, 0.0031% yield), at 51.8 min, **11** (2.9 mg, 0.0014% yield), at 41.5 min, **13** (0.7 mg, 0.00034% yield), at 57.1 min, **20** (5.8 mg, 0.0028% yield), at 42.6 min, **21** (5.5 mg, 0.0027% yield), at 51.0 min, **22** (10.7 mg, 0.0052% yield). Fraction 4 was eluted from the column with 94:6 MeOH/water to give at 76.9 min, **3** (22.9 mg, 0.011% yield), at 61.1 min, **5** (23.3 mg, 0.011% yield) and at 64.0 min, **19** (18.5 mg, 0.0090% yield). Fraction 5 was eluted from the column with 93:7 MeOH/H_2_O solution to give at 50.0 min, **4** (6.1 mg, 0.0030% yield), at 48.1 min, **14** (9.9 mg, 0.0048% yield), and at 52.3 min, mixture of **15** and **16** (4.0 mg, 0.0019% yield). Fraction 9 was eluted from the column with 96:4 MeOH/H_2_O to give at 57.9 min, **12** (3.2 mg, 0.0016% yield). Fraction 2 was eluted from the column with 95:5 MeOH/water to give at 34.5 min, **17** (2.0 mg, 0.00097% yield).

### 3.4. Physical Data of the Compounds

*(3R)-18-Methylnonadec-(4E)-en-1-yn-3-ol* (**1**): Amorphous white solid; [α]^25^_D_ -3.8 (*c* 0.46, CHCl_3_); UV (MeOH) λ_max_ (log ε) 204 (3.35) nm; IR (ATR Diamond) *ν*_max_ 3310, 2920, 2852, 2100, 1651 cm^−1^; ^1^H NMR (500 MHz, CDCl_3_) δ 5.90 (1H, dt, *J* = 15.2, 7.0 Hz, H-5), 5.59 (1H, dd, *J* = 15.2, 6.0 Hz, H-4), 4.82 (1H, d, *J* = 6.0 Hz, H-3), 2.55 (1H, d, *J* = 2.1 Hz, H-1), 2.05 (2H, q, *J* = 7.0 Hz, H_2_-6), 1.50 (1H, qqt, *J* = 6.5, 6.5, 6.5 Hz, H-18), 1.38 (2H, m, H_2_-7), 1.22-1.31 (18H, brm), 1.13 (2H, m, H_2_-17), 0.85 (6H, d, *J* = 6.0 Hz, H_3_-19, H_3_-20); ^13^C NMR (125 MHz, CDCl_3_) δ 134.5 (CH, C-5), 128.3 (CH, C-4), 83.3 (C, C-2), 73.8 (CH, C-1), 62.7 (CH, C-3), 39.0 (CH_2_, C-17), 31.9 (CH_2_, C-6), 29.6 (8 × CH_2_, C-8-15), 28.8 (CH_2_, C-7), 27.9 (CH, C-18), 27.4 (CH_2_, C-16), 22.6 (2 × CH_3_, C-19 and 20); EIGCMS *m/z* 292 [M]^+^ (10), 274 (8), 211 (14), 209 (6), 207 (89), 175 (6), 161 (8), 151 (9), 149 (10), 147 (12), 137 (13), 135 (18), 133 (20), 123 (20), 121 (29), 109 (41), 107 (22), 105 (22), 95 (100), 93 (30), 91 (59), 81 (93), 67 (49); HRAPGCMS *m/z* 293.2848 [M + H]^+^ (calcd for C_20_H_37_O 293.2839).

*(3R)-14-Methylnonadec-(4E)-en-1-yn-3-ol* (**2**): Amorphous white solid; [α]^25^_D_ -0.5 (*c* 0.38, CHCl_3_); UV (MeOH) λ_max_ (log ε) 203 (3.27) nm; IR (ATR Diamond) *ν*_max_ 3304, 2923, 2850, 2098, 1649 cm^−1^; ^1^H NMR (500 MHz, CDCl_3_) δ 5.90 (1H, dt, *J* = 15.0, 7.0 Hz, H-5), 5.60 (1H, dd, *J* = 15.0, 5.7 Hz, H-4), 4.82 (1H, d, *J* = 5.7 Hz, H-3), 2.55 (1H, d, *J* = 2.0 Hz, H-1), 2.06 (2H, q, *J* = 7.0 Hz, H_2_-6), 1.37 (1H, m, H-14), 1.36 (2H, m, H_2_-7), 1.21–1.31 (18H, brm), 1.06 (2H, m, H-13b, H-15b), 0.87 (3H, t, *J* = 7.0 Hz, H_3_-19), 0.83 (3H, d, *J* = 7.0 Hz, H_3_-20); ^13^C NMR (125 MHz, CDCl_3_) δ 134.6 (CH, C-5), 128.3 (CH, C-4), 83.4 (C, C-2), 73.9 (CH, C-1), 62.8 (CH, C-3), 37.0 (2 × CH_2_, C-13, C-15), 32.7 (CH, C-14), 32.2 (CH_2_, C-17), 31.9 (CH_2_, C-6), 29.6 (4 × CH_2_, C-8-11), 28.8 (CH_2_, C-7), 27.0 (CH_2_, C-12), 26.7 (CH_2_, C-16), 22.7 (CH_2_, C-18), 19.7 (CH_3_, C-20), 14.1 (CH_3_, C-19); SMBEIGCMS *m/z* 292 [M]^+^ (23), 277 (11), 263 (12), 249 (26), 235 (30), 221 (38), 207 (8), 193 (15), 179 (14), 175 (21), 163 (20), 149 (42), 137 (63), 121 (76), 109 (100), 95 (79), 81 (83), 107 (22); HRAPGCMS *m/z* 293.2862 [M + H]^+^ (calcd for C_20_H_37_O 293.2839).

*14-Methylnonadec-(4E)-en-1-yn-3-one* (**3**): Colorless oil; UV (MeOH) λ_max_ (log ε) 225 (3.48), 244 (3.81) nm; IR (ATR Diamond) *ν*_max_ 3306, 3247, 2922, 2853, 2098, 1650 cm^−1^; ^1^H NMR (500 MHz, CDCl_3_) δ 7.23 (1H, dt, *J* = 16.0, 7.0 Hz, H-5), 6.17 (1H, d, *J* = 16.0 Hz, H-4), 3.20 (1H, s, H-1), 2.30 (2H, q, *J* = 7.0 Hz, H_2_-6), 1.50 (2H, qi, *J* = 7.0 Hz, H_2_-7), 1.37 (1H, m, H-14), 1.23-1.31 (18H, brm), 1.07 (2H, m, H-13b, H-15b), 0.88 (3H, t, *J* = 7.0 Hz, H_3_-19), 0.83 (3H, d, *J* = 7.0 Hz, H_3_-20); ^13^C NMR (125 MHz, CDCl_3_) δ 177.9 (C, C-3), 156.0 (CH, C-5), 131.9 (CH, C-4), 79.8 (C, C-2), 78.8 (CH, C-1), 37.0 (2 × CH_2_, C-13 & 15), 32.7 (CH_2_, CH, C-6, C-14), 32.2 (CH_2_, C-17), 29.6 (3 × CH_2_, C-9–11), 29.2 (CH_2_, C-8), 27.8 (CH_2_, C-7), 27.0 (CH_2_, C-12), 26.7 (CH_2_, C-16), 22.7 (CH_2_, C-18), 19.7 (CH_3_, C-20), 14.1 (CH_3_, C-19); SMBEIGCMS *m/z* 290 [M]^+^ (61), 275 (18), 261 (27), 247 (30), 233 (53), 220 (49), 219 (47), 205 (18), 191 (33), 177 (44), 163 (87), 149 (99), 135 (93), 121 (96), 109 (91), 95 (100), 81 (99); HRAPGCMS *m/z* 291.2680 [M + H]^+^ (calcd for C_20_H_35_O 291.2682).

*(3R)-13,18-Dimethylnonadec-(4E)-en-1-yn-3-ol* (**4**): Colorless oil; [α]^25^_D_ -8.9 (*c* 0.61, CHCl_3_); UV (MeOH) λ_max_ (log ε) 204 (3.29) nm; IR (ATR Diamond) *ν*_max_ 3309, 2922, 2850, 2099, 1650 cm^−1^; ^1^H NMR (500 MHz, CDCl_3_) δ 5.90 (1H, dt, *J* = 15.0, 7.5 Hz, H-5), 5.59 (1H, dd, *J* = 15.0, 6.0 Hz, H-4), 4.82 (1H, d, *J* = 6.0 Hz, H-3), 2.56 (1H, d, *J* = 2.5 Hz, H-1), 2.05 (2H, q, *J* = 7.5 Hz, H_2_-6), 1.51 (1H, m, H-18), 1.40 (2H, m, H_2_-7), 1.35 (1H, m, H-13), 1.15–1.26 (16H, brm), 1.07 (2H, m, H-12b, H-14b), 0.86 (6H, d, *J* = 7.0 Hz, H_3_-19, H_3_-20), 0.82 (3H, d, *J* = 6.5 Hz, H_3_-21); ^13^C NMR (125 MHz, CDCl_3_) δ 134.5 (CH, C-5), 128.3 (CH, C-4), 83.3 (C, C-2), 73.8 (CH, C-1), 62.7 (CH, C-3), 39.3 (CH_2_, C-17), 37.0 (2 × CH_2_, C-12 C-14), 32.7 (CH, C-13), 31.9 (CH_2_, C-6), 29.6 (3 × CH_2_, C-8, C-9, C-10), 28.8 (CH_2_, C-7), 27.9 (CH, C-17), 27.7 (CH_2_, C-16), 27.0 (2 × CH_2_, C-11, C-15), 22.6 (2 × CH_3_, C-19, C-20), 19.7 (CH_3_, C-21); EIGCMS *m/z* 306 [M]^+^ (14), 291 (3), 288 (8), 273 (3), 263 (2), 249 (1), 245 (3), 235 (1), 221 (2), 209 (10), 207 (8), 191 (3), 189 (5), 179 (5), 175 (6), 165 (4), 163 (6), 161 (9), 151 (8), 149 (12), 147 (11), 137 (11), 135 (18), 133 (21), 123 (20), 121 (28), 119 (25), 93 (45), 109 (41), 107 (30), 105 (30), 95 (100), 93 (31), 91 (82), 81 (94), 67 (43); HRAPGCMS *m/z* 307.3003 [M + H]^+^ (calcd for C_21_H_39_O 307.2995).

*(3R)-14-Methylicos-(4E)-en-1-yn-3-ol* (**5**): Amorphous white solid; [α]^25^_D_ -6.3 (*c* 1.62, CHCl_3_); UV (MeOH) λ_max_ (log ε) 204 (3.30) nm; IR (ATR Diamond) *ν*_max_ 3306, 2922, 2849, 2099 and 1649 cm^−1^; ^1^H NMR (500 MHz, CDCl_3_) δ 5.85 (1H, dt, *J* = 15.0, 6.8 Hz, H-5), 5.56 (1H, dd, *J* = 15.0, 6.0 Hz, H-4), 4.79 (1H, d, *J* = 6.0 Hz, H-3), 2.50 (1H, d, *J* = 2.1 Hz, H-1), 2.02 (2H, q, *J* = 6.8 Hz, H_2_-6), 1.36 (2H, m, H_2_-7), 1.34 (1H, m, H-14), 1.21–1.29 (20H, brm), 1.08 (2H, m, H-13b, H-15b), 0.87 (3H, t, *J* = 6.6 Hz, H_3_-20), 0.83 (3H, d, *J* = 6.6 Hz, H_3_-21); ^13^C NMR (125 MHz, CDCl_3_) δ 134.3 (CH, C-5), 128.4 (CH, C-4), 83.4 (C, C-2), 73.8 (CH, C-1), 62.6 (CH, C-3), 37.0 (2 × CH_2_, C-13, C-15), 32.7 (CH, C-14), 31.9 (2 × CH_2_, C-6, C-18), 29.6 (5 × CH_2_, C-8, C-9, C-10, C-11, C-17), 28.8 (CH_2_, C-7), 27.0 (2 × CH_2_, C-12, C-16), 22.6 (CH_2_, C-19), 19.6 (CH_3_, C-21), 14.0 (CH_3_, C-20); SMBEIGCMS *m/z* 306 [M]^+^ (10), 305 (11), 291 (4), 277 (8), 263 (12), 249 (10), 235 (14), 221 (22), 207 (4), 193 (10), 179 (11), 165 (21), 151 (32), 137 (59), 123 (61), 109 (100), 95 (69), 81 (73); HRAPGCMS *m/z* 307.3027 [M + H]^+^ (calcd for C_21_H_39_O 307.2995).

*14-Methylicos-(4E)-en-1-yn-3-one* (**6**): Colorless oil; UV (MeOH) λ_max_ (log ε) 225 (3.51), 244 (3.86) nm; IR (ATR Diamond) *ν*_max_ 3303, 3247, 2923, 2853, 2098, 1650 cm^−1^; ^1^H NMR (500 MHz, CDCl_3_) δ 7.24 (1H, dt, *J* = 16.0, 7.0 Hz, H-5), 6.17 (1H, d, *J* = 16.0 Hz, H-4), 3.21 (1H, s, H-1), 2.30 (2H, q, *J* = 7.0 Hz, H_2_-6), 1.50 (2H, qi, *J* = 7.0 Hz, H_2_-7), 1.37 (1H, m, H-14), 1.23–1.31 (20H, brm), 1.07 (2H, m, H-13b, H-15b), 0.88 (3H, t, *J* = 6.5 Hz, H_3_-20), 0.83 (3H, d, *J* = 6.5 Hz, H_3_-21); ^13^C NMR (125 MHz, CDCl_3_) δ 177.8 (C, C-3), 155.9 (CH, C-5), 131.9 (CH, C-4), 79.8 (C, C-2), 78.8 (CH, C-1), 37.1 (2 × CH_2_, C-13, C-15), 32.7 (2 × CH, C-6, C-14), 31.9 (CH_2_, C-18), 29.9 (CH_2_, C-17), 29.6 (3 × CH_2_, C-9, C-10, C-11), 29.2 (CH_2_, C-8), 27.8 (CH_2_, C-7), 27.0 (2 × CH_2_, C-12, C-16), 22.7 (CH_2_, C-19), 19.7 (CH_3_, C-20), 14.1 (CH_3_, C-21); SMBEIGCMS *m/z* 304 [M]^+^ (78), 289 (13), 275 (15), 261 (29), 247 (29), 233 (49), 220 (52), 219 (44), 205 (19), 191 (48), 177 (40), 163 (66), 149 (86), 135 (87), 121 (92), 107 (90), 95 (100), 81 (98); HRAPGCMS *m/z* 305.2870 [M + H]^+^ (calcd for C_21_H_37_O 305.2839).

*(3R)-14-Methylicos-1-yn-3-ol* (**7**): Amorphous white solid; [α]^25^_D_ -1.2 (*c* 0.98, CHCl_3_); UV (MeOH) λ_max_ (log ε) 201 (2.02) nm; IR (ATR Diamond) *ν*_max_ 3291, 2918, 2849, 1468 cm^−1^; ^1^H NMR (500 MHz, CDCl_3_) δ 4.36 (1H, td, *J* = 6.5, 2.0 Hz, H-3), 2.46 (1H, d, *J* = 2.0 Hz, H-1), 1.71 (2H, m, H-4), 1.45 (2H, m, H_2_-5), 1.34 (1H, m, H-14), 1.22–1.31 (24H, brm), 1.07 (2H, m, H-13b, H-15b), 0.87 (3H, t, *J* = 7.0 Hz, H_3_-20), 0.83 (3H, d, *J* = 7.0 Hz, H_3_-21); ^13^C NMR (125 MHz, CDCl_3_) δ 85.0 (C, C-2), 72.6 (CH, C-1), 62.2 (CH, C-3), 37.6 (CH_2_, C-4), 37.0 (2 × CH_2_, C-13, C-15), 32.6 (CH, C-14), 31.9 (CH_2_, C-18), 30.0 (CH_2_, C-17), 29.6 (5 × CH_2_, C-7 – C-11), 29.2 (CH_2_, C-6), 27.0 (2 × CH_2_, C-12, C-16), 25.0 (CH_2_, C-5), 22.6 (CH_2_, C-19), 20.1 (CH_3_, C-21), 14.1 (CH_3_, C-20); SMBEIGCMS *m/z* 308 [M]^+^ (1), 307 (2), 290 [M-H_2_O]^+^ (47), 279 (11), 275 (11), 261 (20), 247 (26), 233 (46), 219 (48), 205 (45), 191 (20), 177 (36), 163 (42), 149 (64), 135 (97), 121 (83), 107 (67), 95 (96), 81 (79), 67 (70), 57 (100); HRAPGCMS *m/z* 309.3155 [M + H]^+^ (calcd for C_21_H_41_O 309.3152).

*(3R,E)-12-cis-(2-Hexylcyclopropyl)dodec-4-en-1-yn-3-ol* (**8**): Colorless oil; [α]^25^_D_ -7.2 (*c* 0.32, CHCl3); UV (MeOH) λ_max_ (log ε) 203 (3.24) nm; IR (ATR Diamond) *ν*_max_ 3311, 2920, 2849, 2101, 1650 cm^−1^; ^1^H NMR (500 MHz, CDCl_3_) δ 5.91 (1H, dt, *J* = 15.2, 7.0 Hz, H-5), 5.60 (1H, dd, *J* = 15.2, 5.7 Hz, H-4), 4.83 (1H, d, *J* = 5.7 Hz, H-3), 2.56 (1H, d, *J* = 2.1 Hz, H-1), 2.06 (2H, q, *J* = 7.0 Hz, H_2_-6), 1.36 (2H, m, H_2_-7), 1.13-1.35 (20H, brm), 0.88 (3H, t, *J* = 6.8 Hz, H_3_-20), 0.64 (2H, m), 0.55 (1H, td, *J* = 8.2, 4.0 Hz, H-21a), -0.33 (1H, td, *J* = 4.9, 4.0 Hz, H-21b); ^13^C NMR (125 MHz, CDCl_3_) δ 134.6 (CH, C-5), 128.3 (CH, C-4), 83.3 (C, C-2), 73.9 (CH, C-1), 62.8 (CH, C-3), 31.9 (2 × CH_2_, C-6, C-18), 30.2 (2 × CH_2_), 29.6 (4 × CH_2_, C-8, C-17 and 2 other), 28.8 (CH_2_, C-7), 28.7 (2 × CH_2_), 22.7 (CH_2_, C-19), 15.7 (2 × CH), 14.1 (CH_3_, C-20), 10.9 (CH_2_, C-21); EIGCMS *m/z* 304 [M]^+^ (14), 286 (1), 281 (2) 275 (1), 271 (2), 261 (4), 257 (3), 247 (2), 243 (4), 233 (3), 229 (4), 219 (4), 215 (11), 205 (14), 201 (16), 191 (4), 189 (5), 187 (9), 177 (6), 175 (9), 173 (11), 163 (9), 161 (10), 159 (15), 149 (18), 147 (18), 145 (30), 135 (27), 133 (31), 131 (51), 121 (33), 119 (42), 117 (45), 109 (26), 107 (32), 105 (50), 95 (100), 93 (44), 91 (87), 81 (71), 79 (62), 67 (73), 65 (41), 55 (78), 53 (28); HR-APGC-MS *m/z* 305.2863 [M + H]^+^ (calcd for C_21_H_37_O 305.2839).

*(3R)-13-Methylhenicos-(4E)-en-1-yn-3-ol* (**9**): Colorless oil; [α]^25^_D_ -2.9 (*c* 0.47, CHCl_3_); UV (MeOH) λ_max_ (log ε) 203 (3.29) nm; IR (ATR Diamond) *ν*_max_ 3304, 2925, 2851, 2100, 1649 cm^−1^; ^1^H NMR (500 MHz, CDCl_3_) δ 5.91 (1H, dt, *J* = 15.0, 7.5 Hz, H-5), 5.60 (1H, dd, *J* = 15.0, 6.0 Hz, H-4), 4.82 (1H, d, *J* = 6.0 Hz, H-3), 2.55 (1H, d, *J* = 2.5 Hz, H-1), 2.05 (2H, q, *J* = 7.5 Hz, H_2_-6), 1.37 (1H, m), 1.36 (2H, m, H_2_-7), 1.21–1.30 (22H, brm), 1.07 (2H, m), 0.87 (3H, t, *J* = 7.0 Hz, H_3_-21), 0.83 (3H, d, *J* = 7.0 Hz, H_3_-22); ^13^C NMR (125 MHz, CDCl_3_) δ 134.6 (CH, C-5), 128.3 (CH, C-4), 83.3 (C, C-2), 73.9 (CH, C-1), 62.8 (CH, C-3), 37.1 (2 × CH_2_), 32.7 (CH), 31.9 (2 × CH_2_, C-6, C-19), 29.6 (8 × CH_2_, C-8, C-18 and 6 other), 28.8 (CH_2_, C-7), 27.0 (2 × CH_2_), 22.7 (CH_2_, C-20), 19.7 (CH_3_, C-22), 14.1 (CH_3_, C-21); EIGCMS *m/z* 320 [M]^+^ (21), 302 (8), 179 (5), 165 (5), 163 (8), 161 (9), 151 (10), 149 (18), 147 (18), 137 (14), 135 (20), 133 (21), 123 (19), 121 (22), 119 (23), 109 (32), 107 (24), 105 (30), 95 (100), 93 (29), 91 (61), 81 (51), 78 (42), 67 (39); HRAPGCMS *m/z* 321.3122 [M + H]^+^ (calcd for C22H41O 321.3252).

*Docos-(4E,15Z)-dien-1-yn-3-one (***10***)*: Colorless oil; UV (MeOH) λ_max_ (log ε) 225 (3.48), 244 (3.81) nm; IR (ATR Diamond) *ν*_max_ 3306, 3247, 2922, 2853, 2098, 1650 cm^−1^; ^1^H NMR (500 MHz, CDCl_3_) δ 7.23 (1H, dt, *J* = 16.0, 7.0 Hz, H-5), 6.17 (1H, d, *J* = 16.0 Hz, H-4), 5.35 (2H, bs, H-15 & 16), 3.20 (1H, s, H-1), 2.30 (2H, q, *J* = 7.0 Hz, H_2_-6), 2.02 (4H, q, *J* = 5.5 Hz, H-14 & 17), 1.50 (2H, qi, *J* = 7.0 Hz, H_2_-7), 1.23–1.35 (20H, brm), 0.88 (3H, t, *J* = 6.7 Hz, H_3_-22); ^13^C NMR (125 MHz, CDCl_3_) δ 177.9 (C, C-3), 155.9 (CH, C-5), 131.9 (CH, C-4), 129.9 (CH, C-16), 129.8 (CH, C-15), 79.8 (C, C-2), 78.8 (CH, C-1), 32.7 (CH_2_, C-6), 31.8 (CH_2_, C-20), 29.6 (7 × CH_2_, C-9 to C-13, C-18, C-19), 29.2 (CH_2_, C-8), 27.8 (CH_2_, C-7), 27.2 (2 × CH_2_, C-14, C-17), 22.6 (CH_2_, C-21), 14.1 (CH_3_, C-22); EIGCMS *m/z* 316 [M]^+^ (14, C_22_H_36_O), 273 (6), 259 (19), 245 (32), 231 (16), 234 (13), 217 (13), 211 (12), 209 (33), 207 (24), 203 (11), 191 (27), 177 (18), 163 (20), 161 (20), 149 (22), 147 (48), 135 (40), 133 (77), 121 (61), 119 (29), 107 (95), 95 (71), 93 (50), 91 (41), 81 (96), 79 (63), 69 (42), 67 (84), 55 (100).

*(3R)-Docos-(15Z)-en-1-yn-3-ol (***11***)*: Colorless oil; [α]^25^_D_ -1.0 (*c* 0.29, CHCl_3_); UV (MeOH) λ_max_ (log ε) 201 (2.13) nm; IR (ATR Diamond) *ν*_max_ 3290, 2922, 2850, 1466 cm^−1^; ^1^H NMR (500 MHz, CDCl_3_) δ 5.34 (2H, m, H-15, H-16), 4.36 (1H, td, *J* = 6.5, 2.0 Hz, H-3), 2.45 (1H, d, *J* = 2.0 Hz, H-1), 2.01 (4H, m, H_2_-14, H_2_-17), 1.70 (2H, m, H_2_-4), 1.41 (2H, m, H_2_-5), 1.22–1.33 (24H, brm), 0.87 (3H, t, *J* = 7.0 Hz, H_3_-22); ^13^C NMR (125 MHz, CDCl_3_) δ 129.9 (CH, C-15), 129.9 (CH, C-16), 85.0 (C, C-2), 72.6 (CH, C-1), 62.3 (CH, C-3), 37.6 (CH_2_, C-4), 31.8 (CH_2_, C-20), 29.2–29.7 (9 × CH_2_, C-7 to C-13, C-18, C-19), 29.0 (CH_2_, C-6), 27.2 (2 × CH_2_, C-14, C-17), 25.0 (CH_2_, C-5), 22.6 (CH_2_, C-21), 14.1 (CH_3_, C-22); EIGCMS *m/z* 320 [M]^+^ (2, C_22_H_40_O), 302 (2), 273 (3), 263 (3), 259 (5), 245 (7), 231 (8), 217 (5), 209 (7), 207 (8), 189 (6), 175 (7), 161 (10), 149 (16), 147 (16), 135 (22), 133 (24), 121 (41), 119 (36), 109 (47), 107 (53), 105 (30), 95 (100).

*rac-Tetracos-(4E,15Z)-dien-1-yn-3-ol (***12***)*: Colorless oil; [α]^25^_D_ ~0 (*c* 0.32, CHCl_3_); UV (MeOH) λ_max_ (log ε) 204 (3.30) nm; IR (ATR Diamond) *ν*_max_ 3311, 2923, 2851, 2100, 1650 cm^−1^; ^1^H NMR (500 MHz, CDCl_3_) δ 5.92 (1H, dt, *J* = 15.3, 7.1 Hz, H-5), 5.60 (1H, dd, *J* = 15.3, 6.0 Hz, H-4), 5.34 (2H, t, *J* = 5.0 Hz, H-15, H-16), 4.83 (1H, d, *J* = 6.0 Hz, H-3), 2.56 (1H, bs, H-1), 2.06 (2H, q, *J* = 7.1 Hz, H_2_-6), 2.01 (4H, q, *J* = 5.0 Hz, H_2_-14, H_2_-17), 1.38 (2H, m, H_2_-7), 1.22–1.32 (24H, brm), 0.88 (3H, t, *J* = 6.7 Hz, H_3_-24); ^13^C NMR (125 MHz, CDCl_3_) δ 134.6 (CH, C-5), 129.9 (2 × CH, C-15, C-16), 128.3 (CH, C-4), 83.4 (C, C-2), 73.9 (CH, C-1), 62.8 (CH, C-3), 31.9 (2 × CH_2_, C-6, C-20), 29.6 (10 × CH_2_, C-8 to C-13, C-18 to C-21), 28.8 (CH_2_, C-7), 27.2 (2 × CH_2_, C-14, C-17), 22.7 (CH_2_, C-23), 14.1 (CH_3_, C-24); SMBEIGCMS *m/z* 346 [M]^+^ (22, C_24_H_42_O), 328 (34), 299 (6), 285 (19), 271 (26), 257 (33), 243 (67), 233 (59), 229 (100), 217 (33), 215 (54), 203 (29), 199 (22), 173 (18), 159 (22), 149 (29), 145 (39), 135 (39), 133 (60), 131 (75), 117 (67), 109 (31), 107 (51), 105 (54), 95 (77), 91 (59), 83 (38), 81 (100).

*rac-Icos-(3Z)-en-1-yn-5-ol (***13***)*: Colorless oil; [α]^25^_D_ ~0 (*c* 0.07, CHCl_3_); UV (MeOH) λ_max_ (log ε) 222 (3.38) nm; IR (ATR Diamond) *ν*_max_ 3314, 2923, 2850, 2361, 1712 cm^−1^; ^1^H NMR (500 MHz, CDCl_3_) δ 5.98 (1H, dd, *J* = 11.0, 8.9 Hz, H-4), 5.52 (1H, dd, *J* = 11.0, 1.5 Hz, H-3), 4.67 (1H, q, *J* = 8.5 Hz, H-5), 3.13 (1H, d, *J* = 1.5 Hz, H-1), 1.61 (1H, m, H-6a), 1.52 (1H, m, H-6b), 1.41 (H, m, H-7a), 1.32 (H, m, H-7b), 1.23–1.35 (24H, brm), 0.88 (3H, t, *J* = 7.0 Hz, H_3_-20); ^13^C NMR (125 MHz, CDCl_3_) δ 147.5 (CH, C-4), 108.8 (CH, C-3), 82.7 (CH, C-1), 79.5 (C, C-2), 70.0 (CH, C-5), 36.5 (CH_2_, C-6), 31.9 (CH_2_, C-18), 29.6 (10 × CH_2_, C-8 to C-17), 25.1 (CH_2_, C-7), 22.7 (CH_2_, C-19), 14.1 (CH_3_, C-20); EIGCMS *m/z* [M]^+^ 229.0 (21), 249 (1), 234 (5), 209 (2), 193 (1), 179 (1), 165 (2), 151 (2), 137 (5), 121 (3), 109 (8), 95 (100), 81 (5); HRAPGCMS *m/z* 293.2831 [M + H]^+^ (calcd for C_20_H_37_O 293.2839).

*rac*-*14-Methylicos-(3Z)-en-1-yn-5-ol* (**14***)*: Colorless oil; [α]^25^_D_ ~0 (*c* 0.99, CHCl_3_); UV (MeOH) λ_max_ (log ε) 222 (3.42) nm; IR (ATR Diamond) *ν*_max_ 3312, 2922, 2853, 2359, 1711 cm^−1^; ^1^H NMR (500 MHz, CDCl_3_) δ 5.98 (1H, ddd, *J* = 11.0, 8.0, 1.0 Hz, H-4), 5.53 (1H, ddd, *J* = 11.0, 3.5, 1.0 Hz, H-3), 4.67 (1H, qd, *J* = 8.0, 1.0 Hz, H-5), 3.13 (1H, dd, *J* = 3.5, 1.0 Hz, H-1), 1.62 (1H, m, H-6a), 1.52 (1H, m, H-6b), 1.41 (H, m, H-7a), 1.35 (1H, m, H-14), 1.32 (H, m, H-7b), 1.21–1.33 (20H, brm), 1.07 (2H, m, H-13b/15b), 0.88 (3H, t, *J* = 7.0 Hz, H_3_-20), 0.83 (3H, d, *J* = 6.5 Hz, H_3_-21); ^13^C NMR (500 MHz, CDCl_3_) δ 147.5 (CH, C-4), 108.8 (CH, C-3), 82.7 (CH, C-1), 79.5 (C, C-2), 70.0 (CH, C-5), 37.1 (2 × CH_2_, C-13 & 15), 36.5 (CH_2_, C-6), 32.7 (CH, C-14), 32.3 (CH_2_, C-18), 29.6 (5 × CH_2_, C-8 – 11 & 17), 27.1 (2 × CH_2_, C-12 & 16), 25.1 (CH_2_, C-7), 22.7 (CH_2_, C-19), 19.7 (CH_3_, C-21), 14.1 (CH_3_, C-20); EIGCMS *m/z* 306 [M]^+^ (23), 291 (4), 288 (11), 277 (6), 273 (7), 263 (6), 259 (7), 249 (5), 245 (7), 235 (7), 231 (9), 221 (2), 217 (8), 203 (13), 189 (4), 179 (4), 175 (9), 165 (9), 163 (9), 161 (6), 152 (8), 151 (8), 149 (8), 147 (6), 137 (15), 135 (12), 133 (10), 123 (16), 109 (32), 95 (100), 81 (79); HRAPGCMS *m/z* 307.3017 [M + H]^+^ (calcd for C_21_H_39_O 307.2995).

*rac-18-Methylicos-(3Z)-en-1-yn-5-ol (***15***)*: Colorless oil; [α]^25^_D_ ~0 (*c* 0.40, CHCl_3_); UV (MeOH) λ_max_ (log ε) 223 (3.44) nm; IR (ATR Diamond) *ν*_max_ 3310, 2920, 2853, 2359, 1710 cm^−1^; ^1^H NMR (500 MHz, CDCl_3_) δ 5.97 (1H, dd, *J* = 10.0, 8.0 Hz, H-4), 5.52 (1H, dd, *J* = 11.0, 2.0 Hz, H-3), 4.65 (1H, q, *J* = 8.0 Hz, H-5), 3.13 (1H, d, *J* = 2.0 Hz, H-1), 1.62 (1H, m, H-6a), 1.52 (1H, m, H-6b), 1.39 (H, m, H-7a), 1.32 (H, m, H-7b), 1.22–1.31 (22H, brm), 1.10 (H, m, H-19b), 1.06 (H, m, H-17b), 0.84 (3H, m, H_3_-20), 0.83 (3H, t, *J* = 6.2 Hz, H_3_-21); ^13^C NMR (125 MHz, CDCl_3_) δ 147.5 (CH, C-4), 108.7 (CH, C-3), 82.6 (CH, C-1), 79.6 (C, C-2), 70.0 (CH, C-5), 36.6 (CH_2_, C-17), 36.5 (CH_2_, C-6), 34.4 (CH, C-18), 29.6 (9 × CH_2_, C-8 to C-15, C-19), 27.1 (CH_2_, C-16), 25.0 (CH_2_, C-7), 19.2 (CH_3_, C-21), 11.4 (CH_3_, C-20); EIGCMS *m/z* 306 [M]^+^ (17), 291 (2), 277 (2), 263 (1), 249 (3), 248 (5), 209 (3), 207 (2), 193 (1), 179 (1), 165 (2), 151 (3), 137 (6), 123 (3), 121 (4), 109 (9), 95 (100), 81 (6); HRAPGCMS *m/z* 307.3026 [M + H]^+^ (calcd for C_21_H_39_O 307.2995).

*rac-19-Methylicos-(3Z)-en-1-yn-5-ol (***16***)*: Colorless oil; [α]^25^_D_ ~0 (*c* 0.40, CHCl_3_); UV (MeOH) λ_max_ (log ε) 223 (3.44) nm; IR (ATR Diamond) *ν*_max_ 3310, 2920, 2853, 2359, 1710 cm^−1^; ^1^H NMR (500 MHz, CDCl_3_) δ 5.97 (1H, dd, *J* = 10.5, 8.0 Hz, H-4), 5.52 (1H, dd, *J* = 10.5, 2.0 Hz, H-3), 4.65 (1H, q, *J* = 8.0 Hz, H-5), 3.13 (1H, d, *J* = 2.0 Hz, H-1), 1.62 (1H, m, H-6a), 1.52 (1H, m, H-6b), 1.50 (1H, m, H-19), 1.39 (H, m, H-7a), 1.32 (H, m, H-7b), 1.22–1.30 (20H, brm), 1.13 (2H, m, H_2_-18), 0.85 (6H, t, *J* = 6.5 Hz, H_3_-20, H_3_-21); ^13^C NMR (125 MHz, CDCl_3_) δ 147.5 (CH, C-4), 108.7 (CH, C-3), 82.6 (CH, C-1), 79.6 (C, C-2), 70.0 (CH, C-5), 39.0 (CH_2_, C-18), 36.5 (CH_2_, C-6), 29.6 (9 × CH_2_, C-8 - 16), 27.9 (CH, C-19), 27.4 (CH_2_, C-17), 25.0 (CH_2_, C-7), 22.6 (2 × CH_3_, C-20, C-21); EIGCMS *m/z* 306 [M]^+^ (16), 291 (2), 281 (5), 263 (2), 249 (3), 248 (5), 225 (3), 209 (5), 207 (3), 193 (1), 179 (2), 165 (3), 151 (3), 137 (6); 123 (4), 121 (5), 109 (9), 95 (100), 81 (6); HRAPGCMS *m/z* 307.3029 [M + H]^+^ (calcd for C_21_H_39_O 307.2995).

*14-Methyldocos-(3Z)-en-1-yn-5,6-diol (***17***)*: Amorphous white solid; [α]^25^_D_ -8.0 (*c* 0.20, MeOH); UV (MeOH) λ_max_ (log ε) 204 (3.36), 222 (3.18) nm; IR (ATR Diamond) *ν*_max_ 3311, 2921, 2850, 2101, 1650 cm^−1^; ^1^H NMR (500 MHz, CDCl_3_) δ 6.12 (1H, dd, *J* = 11.0, 9.0 Hz, H-4), 5.68 (1H, dd, *J* = 11.0, 1.5 Hz, H-3), 4.68 (1H, dd, *J* = 9.0, 3.0 Hz, H-5), 3.82 (1H, dt, *J* = 6.5, 3.0 Hz, H-5), 3.16 (1H, d, *J* = 1.5 Hz, H-6), 1.49 (1H, m, H-8a), 1.43 (1H, q, *J* = 6.5 Hz, H-7), 1.34 (H, m, H-8b), 1.35 (1H, m, H-14), 1.24–1.32 (24H, brm), 1.06 (2H, m, H-13b, H-15b), 0.88 (3H, t, *J* = 6.7 Hz, H3-22), 0.83 (3H, d, *J* = 6.5 Hz, H3-23); ^13^C NMR (125 MHz, CDCl_3_) δ 142.2 (CH, C-4), 111.4 (CH, C-3), 83.0 (CH, C-1), 79.3 (C, C-2), 74.1 (CH, C-6), 72.7 (CH, C-5), 37.1 (2 × CH_2_, C-13 & 15), 32.7 (CH, C-14), 31.9 (2 × CH_2_, C-7, C-20), 29.6 (5 × CH_2_, C-10, C-11, C-17 to C-19), 29.4 (CH_2_, C-9), 27.1 (2 × CH_2_, C-12, C-16), 25.8 (CH_2_, C-8), 22.7 (CH_2_, C-21), 19.7 (CH_3_, C-23), 14.1 (CH_3_, C-22); SMBEIGCMS *m/z* 350 [M]^+^ (28, C_23_H_42_O_2_), 332 (16), 317 (1.9), 314 (1.4), 306 (20), 303 (1), 299 (1.3), 289 (1), 285 (1), 275 (1), 271 (1), 269 (11), 257 (1), 261 (1), 249 (3), 247 (1), 243 (1.2), 233 (1.4), 229 (1.4), 219 (1.4), 215 (1.9), 205 (0.9), 201 (1.8), 191 (1.6), 187 (1.1), 177 (1.9), 173 (1.8), 163 (2.4), 159 (1.6), 149 (3.5), 145 (2.2), 139 (6), 135 (5), 131 (3), 125 (7), 121 (7), 117 (2.1), 97 (30), 95 (31), 82 (100), 57 (39).

*(3R)-Icos-(4E)-en-1-yn-3-ol (***18***)*: Amorphous white solid; [α]^25^_D_ -15.2 (*c* 3.50, CHCl_3_); UV (MeOH) λ_max_ (log ε) 204 (3.26) nm; IR (ATR Diamond) *ν*_max_ 3310, 2921, 2851, 2100, 1650 cm^−1^; ^1^H NMR (500 MHz, CDCl_3_) δ 5.87 (1H, dt, *J* = 15.5, 7.0 Hz, H-5), 5.56 (1H, dd, *J* = 15.5, 6.0 Hz, H-4), 4.82 (1H, d, *J* = 6.0 Hz, H-3), 2.53 (1H, d, *J* = 2.0 Hz, H-1), 2.03 (2H, q, *J* = 7.0 Hz, H_2_-6), 1.36 (2H, m, H_2_-7), 1.21–1.36 (24H, brm), 0.85 (3H, t, *J* = 7.0 Hz, H_3_-20; ^13^C NMR (125 MHz, CDCl_3_) δ 134.2 (CH, C-5), 128.4 (CH, C-4), 83.5 (C, C-2), 73.6 (CH, C-1), 62.5 (CH, C-3), 31.9 (CH_2_, C-6), 31.8 (CH_2_, C-18), 29.6 (10 × CH_2_, C-8 to C-17), 28.8 (CH_2_, C-7), 22.6 (CH_2_, C-19), 14.0 (CH_3_, C-20); SMB-EI-GC-MS *m/z* 292 [M]^+^ (10), 291 (13), 263 (13), 249 (18), 235 (8), 221 (7), 207 (5), 193 (8), 179 (9), 165 (14), 151 (30), 137 (57), 123 (59), 109 (100), 95 (59), 81 (78); HRAPGCMS *m/z* 293.2854 [M + H]^+^ (calcd for C_20_H_37_O 293.2839).

*(3R)-19-Methylicos-(4E)-en-1-yn-3-ol (***19***)*: Amorphous white solid; [α]^25^_D_ -1.2 (*c* 1.85, CHCl_3_); UV (MeOH) λ_max_ (log ε) 204 (3.31) nm; IR (ATR Diamond) *ν*_max_ 3304, 2922, 2852, 2098, 1650 cm^−1^; ^1^H NMR (500 MHz, CDCl_3_) δ 5.90 (1H, dt, *J* = 15.0, 7.0 Hz, H-5), 5.59 (1H, dd, *J* = 15.0, 6.0 Hz, H-4), 4.82 (1H, d, *J* = 6.0 Hz, H-3), 2.55 (1H, d, *J* = 2.0 Hz, H-1), 2.05 (2H, q, *J* = 7.0 Hz, H_2_-6), 1.50 (1H, qqt, *J* = 6.0, 6.0, 6.0 Hz, H-19), 1.38 (2H, m, H_2_-7), 1.22–1.31 (20H, brm), 1.14(2H, m, H_2_-18), 0.85 (6H, d, *J* = 6.0 Hz, H_3_-20, H_3_-21); ^13^C NMR (125 MHz, CDCl_3_) δ 134.6 (CH, C-5), 128.3 (CH, C-4), 83.4 (C, C-2), 73.9 (CH, C-1), 62.8 (CH, C-3), 39.0 (CH_2_, C-18), 31.9 (CH_2_, C-6), 29.6 (9 × CH_2_, C-8 to C-16), 28.8 (CH_2_, C-7), 27.9 (CH, C-19), 27.4 (CH_2_, C-17), 22.6 (2 × CH_3_, C-20, C-21); SMBEIGCMS *m/z* 306 [M]^+^ (29), 291 (14), 263 (28), 249 (10), 235 (11), 221 (6), 207 (5), 193 (6), 179 (9), 165 (19), 151 (33), 137 (54), 123 (67), 109 (100), 95 (82), 81 (94), 67 (32); HRAPGCMS *m/z* 307.3013 [M + H]^+^ (calcd for C_21_H_39_O 307.2995).

*(3R)-Henicos-(4E)-en-1-yn-3-ol (***20***)*: Amorphous white solid; [α]^25^_D_ -8.3 (*c* 0.58, CHCl_3_); UV (MeOH) λ_max_ (log ε) 204 (3.29) nm; IR (ATR Diamond) *ν*_max_ 3292, 2917, 2850, 1649 cm^−1^; ^1^H NMR (500 MHz, CDCl_3_) δ 5.92 (1H, dt, *J* = 15.0, 7.0 Hz, H-5), 5.61 (1H, dd, *J* = 15.0, 6.0 Hz, H-4), 4.83 (1H, d, *J* = 6.0 Hz, H-3), 2.56 (1H, s, H-1), 2.07 (2H, q, *J* = 7.0 Hz, H_2_-6), 1.39 (2H, m, H_2_-7), 1.23–1.39 (26H, brm), 0.88 (3H, t, *J* = 6.5 Hz, H_3_-21); EIMS *m/z* [M]^+^ 306 (7), 305 (8), 277 (6), 263 (9), 249 (5), 235 (4), 221 (4), 207 (5), 193 (4), 179 (5), 165 (9), 151 (13), 137 (29), 123 (38), 109 (63), 95 (63), 81 (100); HRAPGCMS *m/z* 307.3027 [M + H]^+^ (calcd for C_21_H_39_O 307.2995).

*(3R)-Docos-(4E,15Z)-dien-1-yn-3-ol (***21***)*: Colorless oil; [α]^25^_D_ -0.5 (*c* 0.55, CHCl_3_); UV (MeOH) λ_max_ (log ε) 204 (3.32) nm; IR (ATR Diamond) *ν*_max_ 3303, 2921, 2852, 2099, 1651 cm^−1^; ^1^H NMR (500 MHz, CDCl_3_) δ 5.90 (1H, dt, *J* = 15.3, 7.0 Hz, H-5), 5.60 (1H, dd, *J* = 15.3, 5.5 Hz, H-4), 5.34 (2H, t, *J* = 5.5 Hz, H-15, H-16), 4.82 (1H, d, *J* = 5.5 Hz, H-3), 2.55 (1H, d, *J* = 2.0 Hz, H-1), 2.06 (2H, q, *J* = 7.0 Hz, H_2_-6), 2.01 (4H, q, *J* = 5.5 Hz, H_2_-14, H_2_-17), 1.38 (2H, m, H_2_-7), 1.22–1.32 (20H, brm), 0.88 (3H, t, *J* = 6.7 Hz, H_3_-22); ^13^C NMR (125 MHz, CDCl_3_) δ 134.5 (CH, C-5), 129.9 (CH, C-16), 129.8 (CH, C-15), 128.3 (CH, C-4), 83.3 (C, C-2), 73.9 (CH, C-1), 62.7 (CH, C-3), 31.9 (CH_2_, C-6), 31.7 (CH_2_, C-20), 29.6 (8 × CH_2_, C-8 to C-13, C-18, C-19), 28.8 (CH_2_, C-7), 27.2 (2 × CH_2_, C-14, C-17), 22.6 (CH_2_, C-21), 14.1 (CH_3_, C-22); EIGCMS *m/z* 318 [M]^+^ (12, C_22_H_38_O), 300 (7), 275 (4), 271 (4), 257 (8), 243 (16), 229 (21), 215 (25), 201 (13), 187 (13), 173 (13), 161 (13), 159 (19), 149 (17), 147 (29), 145 (37), 135 (30), 133 (49), 131 (63), 121 (41), 119 (53), 117 (52), 109 (29), 107 (43), 105 (60), 95 (95), 93 (45), 91 (100), 81 (74), 79 (50).

*(3R)-21-Methyldocos-(4E,15Z)-dien-1-yn-3-ol (***22***)*: Amorphous white solid; [α]^25^_D_ -3.9 (*c* 1.07, CHCl_3_); UV (MeOH) λ_max_ (log ε) 204 (3.33) nm; IR (ATR Diamond) *ν*_max_ 3310, 2924, 2853, 2097, 1650 cm^−1^; ^1^H NMR (500 MHz, CDCl_3_) δ 5.92 (1H, dt, *J* = 15.0, 7.0 Hz, H-5), 5.61 (1H, dd, *J* = 15.0, 6.0 Hz, H-4), 5.34 (2H, t, *J* = 4.0 Hz, H-15, H-16), 4.83 (1H, d, *J* = 6.0 Hz, H-3), 2.56 (1H, s, H-1), 2.06 (2H, q, *J* = 7.0 Hz, H_2_-6), 2.01 (4H, m, H_2_-14, H_2_-17), 1.39 (2H, m, H_2_-7), 1.50 (1H, qqt, *J* = 6.5, 6.5, 6.5 Hz, H-21), 1.17–1.32 (18H, brm), 0.85 (6H, d, *J* = 6.5 Hz, H_3_-22, H_3_-23); ^13^C NMR (125 MHz, CDCl_3_) δ 134.6 (CH, C-5), 129.9 (2 × CH, C-15, C-16), 128.3 (CH, C-4), 83.3 (C, C-2), 73.9 (CH, C-1), 62.8 (CH, C-3), 39.3 (CH_2_, C-20), 31.9 (CH_2_, C-6), 30.0 (CH_2_, C-18), 29.6 (6 × CH_2_, C-8 to C-13), 28.8 (CH_2_, C-7), 28.0 (CH, C-21), 27.2 (2 × CH_2_, C-17, C-19), 27.1 (CH_2_, C-14), 22.6 (2 × CH_3_, C-22, C-23); EIMS *m/z* 332 [M]^+^ (7, C_23_H_40_O), 314 (9), 299 (5), 289 (4), 271 (9), 257 (10), 243 (19), 229 (24), 215 (20), 203 (10), 189 (9), 175 (10), 161 (11), 149 (13), 147 (15), 145 (15), 135 (21), 133 (27), 131 (25), 121 (30), 119 (29), 117 (26), 109 (43), 107 (41), 105 (32), 95 (75), 91 (39), 83 (48), 81 (96), 69 (63), 55 (100).

*(3R)-14-Methyldocos-(4E)-en-1-yn-3-ol (***23***)*: Amorphous white solid; [α]^25^_D_ -6.4 (*c* 2.82, CHCl_3_); UV (MeOH) λ_max_ (log ε) 204 (3.28) nm; IR (ATR Diamond) *ν*_max_ 3305, 2921, 2851, 2099, 1651 cm^−1^; ^1^H NMR (500 MHz, CDCl_3_) δ 5.90 (1H, dt, *J* = 15.3, 7.0 Hz, H-5), 5.60 (1H, dd, *J* = 15.3, 5.5 Hz, H-4), 4.82 (1H, d, *J* = 5.5 Hz, H-3), 2.55 (1H, d, *J* = 2.0 Hz, H-1), 2.06 (2H, q, *J* = 7.0 Hz, H_2_-6), 1.38 (2H, m, H_2_-7), 1.34 (1H, m, H-14), 1.23–1.31 (24H, brm), 1.06 (2H, m, H-13b, H-15b), 0.87 (3H, t, *J* = 6.7 Hz, H_3_-22), 0.83 (3H, d, *J* = 6.5 Hz, H_3_-23); ^13^C NMR (125 MHz, CDCl_3_) δ 134.5 (CH, C-5), 128.3 (CH, C-4), 83.4 (C, C-2), 73.9 (CH, C-1), 62.7 (CH, C-3), 37.1 (2 × CH_2_, C-13 & 15), 32.7 (CH, C-14), 31.9 (2 × CH_2_, C-6, C-20), 29.6 (7 × CH_2_, C-8 to C-11, C-17 to C-19), 28.8 (CH_2_, C-7), 27.1 (2 × CH_2_, C-12, C-16), 22.7 (CH_2_, C-21), 19.7 CH_3_, C-23), 14.1 (CH_3_, C-22); SMBEIGCMS *m/z* 334.4 [M]^+^ (14, C_23_H_42_O), 319 (5), 305 (8), 291 (11), 277 (9), 263 (10), 249 (14), 235 (23), 221 (29), 207 (6), 193 (15), 179 (13), 165 (26), 151 (37), 121 (37), 109 (100), 95 (80), 81 (89).

### 3.5. General Procedure for the Preparation of (S)- or (R)-MPTA Esters of Compounds **7**, **11**, **14**, **15**, **16**, **18**, **23**, **27** and **32**

A CH_2_Cl_2_ (0.5 mL) solution of each of the acetylenic alcohol (0.5 mg) was treated with (*S*)- or (*R*)-MTPA chloride (0.5 μL) and *N*,*N*-dimethylaminopyridine (1.0 mg). The reaction mixture was stirred at room temperature for 1 h. The solvent was evaporated; the crude reaction mixture was quenched with saturated aq. NH4Cl and extracted with EtOAc. The residue was purified on a silica open column eluted with a gradient of solvents from petroleum ether (PE) to 4:1 PE/EtOAc. 

*(S)-MTPA ester of***7**: Relevant ^1^H NMR (500 MHz, CDCl_3_) δ 5.51 (1H, td, *J* = 6.6, 1.8 Hz, H-3), 2.49 (1H, d, *J* = 2.0 Hz, H-1), 1.86 (2H, m, H-4).

*(R)-MTPA ester of***7**: Relevant ^1^H NMR (500 MHz, CDCl_3_) δ 5.54 (1H, td, *J* = 6.7, 2.0 Hz, H-3), 2.53 (1H, d, *J* = 2.0 Hz, H-1), 1.79 (2H, m, H-4).

*(S)-MTPA ester of***11**: Relevant ^1^H NMR (500 MHz, CDCl_3_) δ 5.51 (1H, td, *J* = 6.6, 1.8 Hz, H-3), 2.49 (1H, d, *J* = 2.0 Hz, H-1), 1.86 (2H, m, H-4).

*(R)-MTPA ester of***11**: Relevant ^1^H NMR (500 MHz, CDCl_3_) δ 5.54 (1H, td, *J* = 6.7,2.0 Hz, H-3), 2.53 (1H, d, *J* = 2.0 Hz, H-1), 1.79 (2H, m, H-4).

*(S)-MTPA ester of***14**: Relevant ^1^H NMR (500 MHz, CDCl_3_) δ 5.97 (1H, dd, *J* = 11.0, 8.5 Hz, H-4), 5.66 (1H, dd, *J* = 11.0, 2.5 Hz, H-3), 3.11 (1H, d, *J* = 2.0 Hz, H-1).

*(R)-MTPA ester of* **14**: Relevant ^1^H NMR (500 MHz, CDCl_3_) δ 5.82 (dd, *J* = 10.5, 8.5 Hz, 1H, H-4), 5.63 (1H, dd, *J* = 10.5, 2.5 Hz, 1H, H-3), 3.10 (1H, d, *J* = 2.0 Hz, H-1).

*(S)-MTPA ester of* **15**: Relevant ^1^H NMR (500 MHz, CDCl_3_) δ 5.97 (1H, dd, *J* = 11.0, 8.5 Hz, H-4), 5.66 (1H, dd, *J* = 11.0, 2.5 Hz, H-3), 3.11 (1H, d, *J* = 2.0 Hz, H-1).

*(R)-MTPA ester of* **15**: Relevant ^1^H NMR (500 MHz, CDCl_3_) δ 5.82 (dd, *J* = 10.5, 8.5 Hz, 1H, H-4), 5.63 (1H, dd, *J* = 10.5, 2.5 Hz, 1H, H-3), 3.10 (1H, d, *J* = 2.0 Hz, H-1).

*(S)-MTPA ester of* **16**: Relevant ^1^H NMR (500 MHz, CDCl_3_) δ 5.97 (1H, dd, *J* = 11.0, 8.5 Hz, H-4), 5.66 (1H, dd, *J* = 11.0, 2.5 Hz, H-3), 3.11 (1H, d, *J* = 2.0 Hz, H-1).

*(R)-MTPA ester of* **16**: Relevant ^1^H NMR (500 MHz, CDCl_3_) δ 5.82 (dd, *J* = 10.5, 8.5 Hz, 1H, H-4), 5.63 (1H, dd, *J* = 10.5, 2.5 Hz, 1H, H-3), 3.10 (1H, d, *J* = 2.0 Hz, H-1).

*(S)-MTPA ester of* **18**: Relevant ^1^H NMR (500 MHz, CDCl_3_) δ 6.08 (1H, dt, *J* = 15.0, 6.5 Hz, H-5), 6.00 (1H, d, *J* = 6.0 Hz, H-3), 5.60 (1H, dd, *J* = 15.0, 6.5 Hz, H-4), 2.58 (1H, d, *J* = 2.0 Hz, H-1), 2.08 (2H, q, *J* = 7.0 Hz, H_2_-6).

*(R)-MTPA ester of* **18**: Relevant ^1^H NMR (500 MHz, CDCl_3_) δ 6.03 (1H, d, *J* = 7.0 Hz, H-3), 6.01 (1H, m, H-5), 5.50 (1H, dd, *J* = 15.0, 6.5 Hz, H-4), 2.63 (1H, d, *J* = 2.0 Hz, H-1), 2.04 (2H, q, *J* = 7.0 Hz, H_2_-6).

*(S)-MTPA ester of* **23**: Relevant ^1^H NMR (500 MHz, CDCl_3_) δ 6.08 (1H, dt, *J* = 15.0, 6.5 Hz, H-5), 6.00 (1H, d, *J* = 6.0 Hz, H-3), 5.60 (1H, dd, *J* = 15.0, 6.5 Hz, H-4), 2.58 (1H, d, *J* = 2.0 Hz, H-1), 2.08 (2H, q, *J* = 7.0 Hz, H_2_-6).

*(R)-MTPA ester of* **23**: Relevant ^1^H NMR (500 MHz, CDCl_3_) δ 6.03 (1H, d, *J* = 7.0 Hz, H-3), 6.01 (1H, m, H-5), 5.50 (1H, dd, *J* = 15.0, 6.5 Hz, H-4), 2.63 (1H, d, *J* = 2.0 Hz, H-1), 2.04 (2H, q, *J* = 7.0 Hz, H_2_-6).

*(S)-((R)-icos-(4E)-en-1-yn-3-yl-3,3,3-trifluoro-2-methoxy-2-phenylpropanoate (S,R)-**29**:*

Amorphous white solid; [α]^25^_D_-31.4 (c 0.84, CHCl_3_); UV (MeOH) λ_max_ (log ε) 202 (3.63), 254 (3.29) nm; IR (ATR Diamond) *ν*_max_ 3311, 2924, 2854, 1751 cm^−1^; ^1^H NMR (500 MHz, CDCl_3_) δ 7.40, 7.54 (5H, m, Ph), 6.08 (1H, td, *J* = 15.0, 6.5 Hz, H-5), 6.00 (1H, d, *J* = 6.0 Hz, H-3), 5.60 (1H, dd, *J* = 6.5, 15.0 Hz, H-4), 3.56 (3H, s, -OCH_3_), 2.58 (1H, d, *J* = 2.0 Hz, H-1), 2.08 (2H, q, *J* = 7.0 Hz, H_2_-6), 1.39 (2H, m, H_2_-7), 1.25 (24H, bm), 0.88 (3H, t, *J* = 6.5 Hz, H_3_-20).

*(S)-((S)-icos-(4E)-en-1-yn-3-yl)-3,3,3-trifluoro-2-methoxy-2-phenylpropanoate (S,S)-**29**:*

Amorphous white solid; [α]^25^_D_ -21.6 (c 0.37, CHCl_3_); UV (MeOH) λ_max_ (log ε) 202 (3.61), 254 (3.24) nm; IR (ATR Diamond) *ν*_max_ 3310, 2924, 2853, 1750 cm^−1^; ^1^H NMR (500 MHz, CDCl_3_) δ 7.40, 7.53 (5H, m, Ph), 6.03 (1H, d, *J* = 7.0 Hz, H-3), 6.01 (1H, m, H-5), 5.50 (1H, dd, *J* = 6.5, 15.0 Hz, H-4), 3.60 (3H, s, -OCH_3_), 2.63 (1H, d, *J* = 2.0 Hz, H-1), 2.04 (2H, q, *J* = 7.0 Hz, H_2_-6), 1.36 (2H, m, H_2_-7), 1.25 24H, (bm), 0.88 (3H, t, *J* = 7.0 Hz, H_3_-20).

*(R)-((S)-octadec-1-yn-3-yl)-3,3,3-trifluoro-2-methoxy-2-phenylpropanoate* (*R,S*)-**34**:

Amorphous white solid; UV (MeOH) λ_max_ (log ε) 208 (3.53), 251 (3.24), 256 (3.26), 261 (3.23), 267 (3.20) nm; IR (ATR Diamond) *ν*_max_ 3295, 2917, 2850, 1746 cm^−1^; ^1^H NMR (500 MHz, CDCl_3_) δ 7.38, 7.41, 7.53 (5H, m, Ph), 5.51 (1H, dt, *J* = 1.8, 6.6 Hz, H-3), 3.55 (3H, s, -OCH_3_), 2.49 (1H, d, *J* = 2.0 Hz, H-1), 1.86 (2H, m, H_2_-4), 1.44 (2H, m, H_2_-5), 1.25 (24H, bm), 0.88 (3H, t, *J* = 6.5 Hz, H_3_-18); ^13^C NMR (125 MHz, CDCl_3_) 165.7 (C, -CO_2−_), 132.3 (C, Ph), 129.7, 128.4, 127.4 (5 CH, Ph), 79.7 (C, C-2), 74.6 (CH, C-1), 66.2 (CH, C-3), 55.5 (CH_3_, -OCH_3_), 34.3 (CH_2_, C-4), 32.0 (CH_2_, C-16), 29.7, 29.6, 29.5, 29.4 (9 × CH_2_, C-7-C-15), 28.9 (CH_2_, C-6), 24.9 (CH_2_, C-5), 22.7 (CH_2_, C-17), 14.2 (CH_3_, C-18). 

*(R)-((R)-octadec-1-yn-3-yl)-3,3,3-trifluoro-2-methoxy-2-phenylpropanoate* (*R,R*)-**34**:

Amorphous white solid; UV (MeOH) λ_max_ (log ε) 207 (3.55), 251 (3.19), 256 (3.22), 261 (3.22), 267 (3.17) nm; IR (ATR Diamond) *ν*_max_ 3295, 2917, 2850, 1746 cm^−1^; ^1^H NMR (500 MHz, CDCl_3_) δ 7.38, 7.41, 7.54 (5H, m, Ph), 5.54 (1H, dt, *J* = 2.0, 6.7 Hz, -C**H**(O(S)MTPA)-), 3.60 (3H, s, -OCH_3_), 2.53 (1H, d, *J* = 2.0 Hz, **H**-C≡C-), 1.79 (2H, m, -CH(O(S)MTPA)-CH_2_-), 1.31 (2H, m, -CH(O(S)MTPA)-CH_2_-CH_2_-), 1.25 (24H, bm, CH_2_), 0.88 (3H, t, *J* = 6.5 Hz, CH_3_); ^13^C NMR (125 MHz, CDCl_3_) δ 165.7 (C, -CO_2−_), 132.3 (C, Ph), 129.6, 128.4, 127.3 (5 CH, Ph), 80.0 (C, C-2), 74.7 (CH, C-1), 65.7 (CH, C-3), 55.5 (CH_3_, -OCH_3_), 34.3 (CH_2_, C-4), 31.9 (CH_2_, C-16), 29.7, 29.6, 29.4, 29.3 (9 × CH_2_, C-7 to C-15), 28.8 (CH_2_, C-6), 24.6 (CH_2_, C-5), 22.7 (CH_2_, C-17), 14.1 (CH_3_, C-18).

*General procedure for periodate-permanganate oxidation of compounds***10***,***12***,***21***, and***22** [28]: The t-BuOH solution (1 mL) of each of the acetylenic compounds (0.5 mg) was treated with 0.1 mL of water solutions of K2CO3 (0.1 M), KMnO_4_ (0.01 M) and NaIO4 (0.1 M) and stirred in room temperature for 24 h. The reaction was neutralized with a few drops of diluted H_2_SO_4_ (10%) water solution and extracted with petroleum ether. The solvent was evaporated and the precipitation was dissolved in methanol. The samples were analyzed by LCMS on an ACQUITY UPLC BEH C18 1.7 μm 2.1 × 50 mm column revealing the presence of undecadioic acid as a major product.

*General procedure for oxidation of hexadecanol and octadecanol to***24***and***30**: Hexadecanol (20 g, 82.6 mmol) was dissolved in 200 mL of anhydrous dichloromethane. The mixture was added to a stirred suspension consisting of pyridinium chlorochromate (PCC) (26.8 g, 124 mmol) and Celite (26.8 g) in 250 mL of anhydrous dichloromethane. The resulting suspension was stirred for 3 h at rt and the progress of the reaction was monitored by TLC (Silica Gel 60, 9:1 petroleum ether/ethyl acetate (PE/EtOAc). Upon the disappearance of the starting material, the suspension was filtered twice through a filter paper (Whatman 2). The solvent was evaporated and the residue dissolved in 500 mL of PE. The resulting suspension was filtered through a thin layer of silica, the filter cake was rinsed with an additional 200 mL of PE and the filtrates were combined. The solvent was removed under reduced pressure to afford hexadecanal (**24**) (17.08 g, 71.1 mmol, 85%) as an amorphous white solid. The same procedure was used for the synthesis of octadecanal (**30**), from octadecanol, in an 83% yield.

*Hexadecanal (***24***)*: Amorphous white solid; ^1^H NMR (400 MHz, CDCl_3_) δ 9.71 (1H, bs, CHO), 2.37 (2H, m, CH_2_CHO), 1.58 (2H, m, C**H**2CH_2_CHO), 1.23 (24 × H, bm), 0.84 (3H, t, *J* = 6.5 Hz, CH_3_).

*Octadecanal (***30***)*: Amorphous white solid; ^1^H NMR (500 MHz, CDCl_3_) δ 9.74 (1H, s, CHO), 2.39 (2H, t, *J* = 6.5 Hz, C**H**2CHO), 1.60 (2H, m, C**H**2CH_2_CHO), 1.25 (28 × H, bm), 0.85 (3H, t, *J* = 6.5 Hz, CH_3_).

*Preparation of 2-octadecenal (25) from hexadecanal* *(***24***)*: A pressure-resistant tube was charged with a stirring bar, hexadecanal (10.07 g, 42.0 mmol) and 50 mL of dichloromethane. After the addition of the Wittig reagent Ph3P=CHCHO (20.1 g, 66.0 mmol) the tube was flushed with nitrogen and sealed. The mixture was heated to 65 °C and the progress of the reaction was monitored by TLC (Silica Gel 60, 9:1 PE/EtOAc). After four days, most of the aldehyde was reacted and the mixture was diluted with 400 mL of PE and filtered through a thin layer of silica. The solvent was removed under reduced pressure to give the crude product, which was purified by column chromatography on silica gel, with 1% step gradient of eluents from 100% PE to 4:1 PE/EtOAc to afford pure 2-octadecenal (**25**) (2.19 g, 8.23 mmol, 20%) as an amorphous white solid. ^1^H NMR (400 MHz, CDCl_3_) δ 9.48 (1H, d, 7.6, C**H**O), 6.84 (1H, td, *J* = 6.8,15.6 Hz, -C**H**=CHCHO), 6.09 (1H, dd, *J* = 8.0,15.6 Hz, -CH=C**H**CHO), 2.31 (2H, q, *J* = 6.8 Hz, -C**H**_2_-CH=CHCHO), 1.49 (2H, m, C**H**_2_-CH_2_-CH=CH-), 1.24 (24 × H, bm), 0.86 (3H, t, *J* = 6.0 Hz, CH_3_).

*General procedure for production the alkylyncarbinols rac*-**18***, rac*-**27***, rac*-**31***, rac*-**32***, rac*-**33***, rac-***41**, *rac-***42**, *rac-***45**, *rac-***48***and rac-***49** [23]. The solution of 2-octadecenal (**25**) and (2*E*,4*E*)-icos-2,4-dien-1-al (**26**) (<1%), (2.19 g, 8.23 mmol) in 50 mL anhydrous THF was cooled to 0 °C. To this solution, ethynylmagnesium bromide (27.5 mL, 9.9 mmol, 0.5 M solution in THF) was added and the reaction mixture was stirred at 0 °C. The progress of the reaction was monitored by TLC (Silica gel 60, 9:1 PE/EtOAc). After 3 h the reaction was completed, and the mixture was quenched with 50 mL of sat. NH4Cl, and the organic layer extracted with diethyl ether (3 × 50 mL). The combined organic layers were neutralized with 50 mL saturated NaHCO3, separated and dried over anhydrous MgSO4. The solution was filtered and the solvent removed under reduced pressure to afford a crude product (2.41 g) that after separation on a Cosmosyl C-8 preparative HPLC column afforded (*E*)-icos-4-en-1-yn-3-ol (*rac*-**18**) (2.23 g, 7.65 mmol, 93%) and (4*E*,6*E*)-docosa-4,6-dien-1-yn-3-ol (*rac*-**27**) (3.8 mg, 1.2 μmol, 0.15%). The same procedure was used for the preparation of *rac*-**31** (9.5 mg, 52.1 μmol, 93%), *rac*-**32** (570 mg, 2.14 mmol, 93%), *rac-***33** (610 mg, 20.7 mmol, 93%), *rac-***41** (10.2 mg, 34.5 μmol, 86%), *rac-***42** (34.9 mg, 0.11 mmol, 81%), *rac-***45** (7.0 mg, 21.4 μmol, 42%), *rac-***48** (4.5 mg, 13.7 μmol, 43%) and *rac-***49** (95.8 mg, 0.83 mmol, 89%).

*rac-Icos-(4E)-en-1-yn-3-ol (rac*-**18***)*: All data except the absence of optical rotation were identical to those of **18**.

*(4E,6E)-docosa-4,6-dien-1-yn-3-ol (rac*-**27***)*: Amorphous white solid; UV (MeOH) λ_max_ (log ε) 231 (3.86) nm, 296 (2.48) nm; IR (ATR Diamond) *ν*_max_ 3386, 3294, 2954, 2917, 2850, 2360, 2338, 2098, 1716, 1625 cm^−1^; ^1^H NMR (500 MHz, CDCl_3_) δ 6.40 (1H, dd, *J* = 15.1, 10.5 Hz, H-5), 6.04 (1H, dd, *J* = 15.1, 10.5 Hz, H-6), 5.79 (1H, dt, *J* = 15.1, 7.5 Hz, H-7), 5.67 (1H, dd, *J* = 15.1, 6.0 Hz, H-4), 4.90 (1H, d, *J* = 6.0 Hz, H-3), 2.58 (1H, d, *J* = 2.1 Hz, H-1), 2.08 (2H, q, *J* = 7.5 Hz, H_2_-8), 1.37 (2H, m, H_2_-9), 1.21–1.30 (24H, bm), 0.88 (3H, t, *J* = 6.8 Hz, H_3_-22); ^13^C NMR (125 MHz, CDCl_3_) δ 137.8 (CH, C-7), 133.0 (CH, C-5), 128.6 (CH, C-6), 128.2 (CH, C-4), 83.0 (C, C-2), 74.2 (CH, C-1), 62.6 (CH, C-3), 32.7 (CH_2_, C-8), 31.9 (CH_2_, C-20), 29.6 (10 × CH_2_, C-8 to C-17), 29.1 (CH_2_, C-9), 22.7 (CH_2_, C-21), 14.2 (CH_3_, C-22); EIGCMS [M]^+^ *m/z* 318 (5), 250 (8), 207 (8), 191 (4), 177 (10), 163 (13), 149 (19), 135 (40), 121 (61), 107 (100), 94 (64), 79 (64), 67 (51), 55 (66), 43 (73); HREIMS *m/z* 318.2940 [M]^+^ (calcd for C_22_H_38_O 318.2923).

*Dodec-1-yn-3-ol (rac*-**31***)*: Amorphous white solid; UV (MeOH) λ_max_ (log ε) 206 (2.29) nm; IR (ATR Diamond) *ν*_max_ 3311, 2923, 2854, 2360, 2342, 1465 cm^−1^; ^1^H NMR (500 MHz, CDCl_3_) δ 4.36 (1H, td, *J* = 6.5, 2.0 Hz, H-3), 2.45 (1H, d, *J* = 2.5 Hz, H-1), 1.71 (2H, m, H2-4), 1.44 (2H, qi, *J* = 6.5 Hz, H_2_-5), 1.22–1.31 (12H, bm), 0.87 (3H, t, *J* = 6.5 Hz, H_3_-12); ^13^C NMR (125 MHz, CDCl_3_) δ 85.0 (C, C-2), 72.8 (CH, C-1), 62.3 (CH, C-3), 37.6 (CH_2_, C-4), 31.9 (CH_2_, C-10), 29.5, 29.3 (3 × CH_2_, C-7 to C-9), 29.2 (CH_2_, C-6), 25.0 (CH_2_, C-5), 22.6 (CH_2_, C-11), 14.1 (CH_3_, C-12); HRAPGCMS [M + H]^+^, *m/z* 183.1754 (calcd for C_12_H_23_O, 183.1743).

*Octadec-1-yn-3-ol (rac*-**32***)*: Amorphous white solid; UV (MeOH) λ_max_ (log ε) 203 (2.20) nm; IR (ATR Diamond) *ν*_max_ 3287, 2916, 2849, 1466 cm^−1^; ^1^H NMR (500 MHz, CDCl_3_) δ 4.36 (1H, td, *J* = 8.5, 2.5 Hz, H-3), 2.46 (1H, d, *J* = 2.5 Hz, H-1), 1.69 (2H, m, H_2_-4), 1.44 (2H, m, H_2_-5), 1.22–1.31 (24H, bm), 0.87 (3H, t, *J* = 7.0 Hz, H_3_-18); ^13^C NMR (125 MHz, CDCl_3_) δ 85.1 (C, C-2), 72.8 (CH, C-1), 62.2 (CH, C-3), 37.7 (CH_2_, C-4), 31.9 (CH_2_, C-16), 29.7, 29.6, 29.5, 29.3 (9 × CH_2_, C-7 to C-15), 29.2 (CH_2_, C-6), 25.0 (CH_2_, C-5), 22.8 CH_2_, C-17), 14.1 (CH_3_, C-18); HRAPGCMS [M + H]^+^, *m/z* 267.2706 (calcd for C_18_H_35_O, 267.2682).

*Icos-1-yn-3-ol (rac*-**33***)*: Amorphous white solid; UV (MeOH) λ_max_ (log ε) 204 (2.18) nm; IR (ATR Diamond) *ν*_max_ 3287, 2916, 2849, 1466 cm^−1^; ^1^H NMR (500 MHz, CDCl_3_) δ 4.35 (1H, t, *J* = 7.0 Hz, H-3), 2.44 (1H, d, *J* = 2.0 Hz, H-1), 1.69 (2H, q, *J* = 7.0 Hz, H_2_-4), 1.44 (2H, qi, *J* = 7.0 Hz, H_2_-5), 1.22–1.31 (28H, bm), 0.87 (3H, t, *J* = 7.0 Hz, H_3_-20); ^13^C NMR (125 MHz, CDCl_3_) δ 85.1 (C, C-2), 72.7 (CH, C-1), 62.2 (CH, C-3), 37.6 (CH_3_, C-4), 31.9 (CH_2_, C-18), 29.8, 29.7, 29.6, 29.4 (11 × CH_2_, C-7 to C-17), 29.2 (CH_2_, C-6), 25.1 (CH_2_, C-5), 22.8 (CH_2_, C-19), 14.1 (CH_3_, C-20); HRAPGCMS [M + H]^+^, *m/z* 295.3019 (calcd for C_20_H_39_O, 295.2995).

*3-Methylnonadec-1-yn-3-ol (rac*-**41***)*: Amorphous white solid; UV (MeOH) λ_max_ (log ε) 205 (2.01) nm; IR (ATR Diamond) *ν*_max_ 3306, 2916, 2849, 2360, 2341, 1468 cm^−1^; ^1^H NMR (500 MHz, CDCl_3_) δ 2.43 (1H, s, H-1), 1.81 (1H, bs, -CMe(O**H**)-), 1.65 (2H, q, *J* = 6.5 Hz, H_2_-4), 1.48 (3H, s, -CC**H**_3_(OH)-), 1.22–1.35 (28H, bm), 0.88 (3H, t, *J* = 7.0 Hz, H_3_-19); ^13^C NMR (125 MHz, CDCl_3_) δ 87.8 (C, C-2), 71.2 (CH, C-1), 68.1 (C, C-3), 43.5 (CH_2_, C-4), 31.9 (CH_2_, C-17), 29.7, 29.6, 29.5, 29.4 (11 × CH_2_, C-6 to C-16, and CH_3_, -C**Me**(OH)-), 24.5 (CH_2_, C-5), 22.7 (CH_2_, C-18), 14.1 (CH_2_, C-19); HRAPGCMS [M + H]^+^, *m/z* 295.3021 (calcd for C_20_H_39_O, 295.2995).

*Henicos-2-yn-4-ol (rac*-**42***)*: Amorphous white solid; UV (MeOH) λ_max_ (log ε) 203 (2.29) nm; IR (ATR Diamond) *ν*_max_ 3325, 2957, 2914, 2849, 2360, 2341, 1469 cm^−1^; ^1^H NMR (500 MHz, CDCl_3_) δ 4.31 (1H, tq, *J* = 7.0, 2.0 Hz, H-4), 1.84 (3H, d, *J* = 2.0 Hz, H-1), 1.75 (1H, bs, -CH(O**H**)-), 1.65 (2H, m, H-5), 1.41 (2H, m, H_2_-6), 1.22–1.31 (28H, bm), 0.87 (3H, t, *J* = 6.5 Hz, H_3_-21); ^13^C NMR (125 MHz, CDCl_3_) δ 80.8 (C, C-3), 80.5 (C, C-2), 62.7 (CH, C-4), 38.1 (CH_2_, C-5), 31.9 (CH_2_, C-19), 29.7, 29.6, 29.5, 29.4, 29.3 (12 × CH_2_, C-7 to C-18), 25.2 (CH_2_, C-6), 22.7 (CH_2_, C-20), 14.1 (CH_3_, C-21), 3.5 (CH_3_, C-1); HRAPGCMS [M + H]^+^, *m/z* 309.3178 (calcd for C_21_H_41_O, 309.3152).

*1-Phenylprop-2-yn-1-ol (rac*-**49***)*: Amorphous white solid; UV (MeOH) λ_max_ (log ε) 209 (3.45) nm; IR (ATR Diamond) *ν*_max_ 3288, 2962, 2854, 2116, 1493, 1458 cm^−1^; ^1^H NMR (500 MHz, CDCl_3_) δ 7.55 (2H, d, *J* = 7.5 Hz, Ph), 7.40(2H, t, *J* = 7.0 Hz, Ph), 7.34 (1H, t, J = 7.0 Hz, Ph), 5.45 (1H, d, *J* = 1.5 Hz, -CH(OH)-), 2.67 (1H, d, *J* = 2.0 Hz, **H**-C≡C-); ^13^C NMR (125 MHz, CDCl_3_) δ 140.0 (C, Ph), 128.6, 128.4, 126.6 (5 × CH, Ph), 83.5 (C, C-2), 74.8 (CH, C-1), 64.2 (CH, C-3); HRAPCIMS [M − H_2_O + H]^+^, *m/z* 115.0545 (calcd for C_9_H_7_, 115.0548).

*Kinetic resolution ofrac-18 to produce (R,E)-icos-4-en-1-yn-3-ol***(***scal-R*-**18***) and (S,E)-icos-4-en-1-yn-3-yl acetate (***28***)* [23]: (*E*)-icos-4-en-1-yn-3-ol (*rac*-**18**) (2.3 g, 7.84 mmol) was dissolved in PE (100 mL) and treated with lipase AK “Amano” (1.0 g), molecular sieves (1.0 g) and vinyl acetate (3.1 mL, 33.7 mmol). The reaction mixture was stirred at room temperature for 6 hr. The progress of the reaction was monitored by TLC (Silica Gel 60, 9:1 PE/EtOAc), and ^1^H NMR. When the reaction reached 60% conversion, the mixture was filtered through Celite. The filter cake was washed with 100 mL PE and the solvent was removed under reduced pressure. The products were separated by column chromatography on silica gel, with 1% step gradient, from 100% PE to 4:1 PE/EtOAc, affording (*R*,E)-icos-4-en-1-yn-3-ol (*scal-R*-**18**) [0.85 g, 2.9 mmol, 37%, ee 30, [α]^25^_D_ -4.8 (c 1.50, CHCl_3_)] as an amorphous white solid, and (*S*,*E*)-icos-4-en-1-yn-3-yl acetate (*scal-S-***28**) (1.35 g, 4.0 mmol, 51%, ee 36) as an amorphous white solid. The enantiomeric excess of the compounds was determined by the integration of the methoxy signal in the ^1^H-NMR spectra of the corresponding Mosher esters. All other physiochemical data of *scal-R*-**18** were identical to those of *rac*-**18**.

*(S,E)-icos-4-en-1-yn-3-yl acetate (scal-S*-**28***)*: Amorphous white solid; UV (MeOH) λ_max_ (log ε) 204 (3.44) nm; IR *ν*_max_ 3313, 2918, 2850, 1730, 1688 cm^−1^; ^1^H NMR (400 MHz, CDCl_3_) δ 6.02 (1H, dt, *J* = 15.2, 7.6 Hz, H-5), 5.83 (1H, d, *J* = 5.2 Hz, H-3), 5.55 (1H, dd, *J* = 15.2, 6.4 Hz, H-4), 2.55 (1H, d, *J* = 2.0 Hz, H-1), 2.09 (2H, m, H_2_-6), 2.09 (s, 3H, CO-CH_3_), 1.52 (2H, m, H_2_-7), 1.21–1.33 (24 × H, bm), 0.87 (3H, t, *J* = 6.0 Hz, H_3_-20).

*Hydrolysis of scal-S-****28****to produce scal-S-****18*** [14]: The solution of (*S*,*E*)-icos-4-en-1-yn-3-yl acetate (*scal-S*-**28**) (1.35 g, 4.0 mmol) in 30 mL methanol was treated with K2CO3 (20 mg). The reaction was allowed to stand at rt and its progress was monitored by TLC (Silica Gel 60, 9:1 PE/EtOAc). After 3 h, the starting material disappeared, and the reaction was quenched with 10 mL HCl (1M) and the organic layer was extracted three times with diethyl ether. The organic solvent was removed under reduced pressure to give the crude product. The crude extract was purified by column chromatography on silica gel, with 2% step gradient from 100% PE to 4:1 PE/EA, affording (*S*)-icos-(4*E*)-en-1-yn-3-ol (*scal-S*-**18**) [0.95 g, 3.26 mmol, 82%, ee 30, [α]^25^_D_ +4.2 (c 1.88, CHCl_3_)] as an amorphous white solid. The enantiomeric excess of the compounds was determined by the integration of the methoxy signal in the ^1^H NMR spectra of the corresponding Mosher esters. All other physiochemical data were identical to those of *rac*-**18**.

*General procedure for hydrolysis of MTPA esters, preparation of pure acetylenes R*-**18,***R*-**32***and S*-**32**: The appropriate MTPA ester, (*S,R*)-**29**, (*R,R*)-**34** or (*R,S*)-**34** were treated with NH3 in methanol in a sealed pressure resistant tube. The reaction was heated to 90 °C and the progress was monitored by TLC (Silica Gel 60, 9:1 PE/EtOAc). After two days the solvent was removed under reduced pressure and the residue was extracted with PE. The crude extract was purified by column chromatography on silica gel, with 1% step gradient from PE to 4:1 PE/EtOAc, affording (*R*,*E*)-icos-4-en-1-yn-3-ol (*R*-**18**) (1.5 mg, 0.0051 mmol, 40%, ee 99), (*S*)-octadec-1-yn-3-ol (*S*-**32**) (8.3 mg, 0.031 mmol, 87%, ee 99) or (*R*)-octadec-1-yn-3-ol (*R*-**32**) (5.9 mg, 0.022 mmol, 89%, ee 99), as amorphous white solids. 

*(R,E)-icos-4-en-1-yn-3-ol* (*R*-**18**): an amorphous white solid. [α]^25^_D_ 16.5 (c 0.63, CHCl_3_). 

*(S)-octadec-1-yn-3-ol* (*S*-**32**): an amorphous white solid. [α]^25^_D_ -3.4 (c 0.83, CHCl_3_). 

*(R)-octadec-1-yn-3-ol* (*R*-**32**): an amorphous white solid. [α]^25^_D_ 6.1 (c 0.59, CHCl_3_). 

The enantiomeric excess of these compounds was determined by the integration of the methoxyl signal in the ^1^H-NMR spectra of the corresponding Mosher esters. All other physiochemical data were identical to those of *rac*-**18** and *rac*-**32**.

*Preparation of octadec-1-yn-3-yl 4-methylbenzenesulfonate (rac*-**35***) and 3-Chlorooctadec-1-yne (rac*-**36***)* [24]. A solution of octadec-1-yn-3-ol (*rac*-**32**) (25.2 mg, 0.095 mmol) in dichloromethane (DCM, 1 mL) was treated with p-toluenesulfonyl chloride (20 mg, 0.105 mmol). The reaction mixture was stirred for 3h at rt, and then water (1mL) was added. The organic layer was separated and the solvent was removed under reduced pressure. The crude reaction mixture was separated by column chromatography on silica gel using a 2% step gradient from PE to 4:1 PE/EtOAc to afford octadec-1-yn-3-yl 4-methylbenzenesulfonate (*rac*-**35**) (25.6 mg, 0.061 mmol, 64%) and 3-Chlorooctadec-1-yne (*rac*-**36**) (3.0 mg, 0.010, 11%). 

*Octadec-1-yn-3-yl 4-methylbenzenesulfonate (rac*-**35***)*: Amorphous white solid; IR (ATR Diamond) *ν*_max_ 3275, 2922, 2851, 1597, 1464 cm^−1^; ^1^H NMR (500 MHz, CDCl_3_) δ 7.82 (2H, d, *J* = 8.0 Hz, Ts), 7.33 (2H, d, *J* = 8.0 Hz, Ts), 5.05 (1H, td, *J* = 7.0, 2.0 Hz, H-3), 2.45 (3H, s, C**H**3-Ar), 2.39 (1H, d, *J* = 2.0 Hz, H-1), 1.81 (2H, m, H_2_-4), 1.41 (2H, m, H_2_-5), 1.22–1.32 (24H, bm), 0.88 (3H, t, *J* = 7.0 Hz, H_3_-18); ^13^C NMR (125 MHz, CDCl_3_) δ 144.8, 134.0 (2 x C, Ts), 129.6, 128.1 (2 x CH, Ts), 79.2 (C, C-2), 76.0 (CH, C-1), 71.2 (CH, C-3), 35.7 (CH_2_, C-4), 31.9 (CH_2_, C-16), 29.9, 29.8, 29.7, 29.6, 29.5, 29.4 (9 × CH_2_, C-7 to C-15), 28.8 (CH_2_, C-6), 24.5 (CH_2_, C-5), 22.7 (CH_2_, C-17), 21.7 (CH_3_, Ts), 14.1 (CH_3_, C-18); HRESIMS [M + Na]^+^, *m/z* 443.2598 (calcd for C25H40O3SNa, 443.2596).

*3-chlorooctadec-1-yne (rac*-**36***)*: Amorphous white solid; UV (MeOH) λ_max_ (log ε) 202 (2.81) nm; IR (ATR Diamond) *ν*_max_ 3311, 2922, 2853, 2361, 2341, 1458 cm^−1^; ^1^H NMR (500 MHz, CDCl_3_) δ 4.50 (1H, td, *J* = 6.8, 2.3 Hz, H-3), 2.59 (1H, d, *J* = 2.3 Hz, H-1), 1.94 (2H, q, *J* = 6.8 Hz, H_2_-4), 1.52 (2H, qi, *J* = 7.6 Hz, H_2_-5), 1.22–1.32 (24H, bm), 0.88 (3H, t, *J* = 6.7 Hz, H_3_-18); ^13^C NMR (125 MHz, CDCl_3_) δ 82.1 (C, C-2), 74.1 (CH, C-1), 47.9 (CH, C-3), 39.0 (CH_2_, C-4), 31.9 (CH_2_, C-16), 29.5, 29.4, 29.3, 29.2, 29.1 (9 × CH_2_, C-7 to C-15), 28.8 (CH_2_, C-6), 26.1 (CH_2_, C-5), 22.7 (CH_2_, C-17), 14.1 (CH_3_, C-18); SMBEIGCMS *m/z* 286/284 (1:3) ([M]^+^, <1), 249 ([M-Cl.]^+^, 5), 219 (3), 205 (3), 191 (3), 177 (5), 163 (8), 149 (17), 135 (49), 123 (68), 121 (62), 109 (100), 107 (71), 102 (55); HREIMS [M-Cl.]^+^, *m/z* 249.2596 (calcd for C_18_H_33_, 249.2582).

*Preparation of octadec-1-yn-3-amine (rac*-**37***) and 3-methoxyoctadec-1-yne (rac*-**38***)*. The octadec-1-yn-3-yl-4-methyl-benzenesulfonate (*rac*-**35**) (16.8 mg, 0.039 mmol) was reacted with NH_3_ in methanol in a pressure resistant vial. The reaction was stirred at 60 °C and its progress was monitored by TLC (9:1 PE/EA). After 3 h the solvent was removed under reduced pressure. The residue was purified by column chromatography on silica gel, with a 2% step gradient from PE to 9:1 PE/EtOAc, affording first, 3-methoxyoctadec-1-yne (rac-**38**) (4.4 mg, 0.016 mmol, 41%) as an amorphous white solid, and then octadec-1-yn-3-amine (rac-**37**) (5.9 mg, 0.022 mmol, 56%) as a colorless oil. 

*Octadec-1-yn-3-amine (rac*-**37***)*: Colorless oil; UV (MeOH) λ_max_ (log ε) 202 (2.70) nm; IR (ATR Diamond) *ν*_max_ 3174, 2955, 2916, 2850, 2360, 2341, 1577, 1470 cm^−1^; ^1^H NMR (500 MHz, CD3OD) δ 3.49 (1H, t, *J* = 8.0 Hz, H-3), 2.60 (1H, s, H-1), 1.46 (2H, q, *J* = 7.5 Hz, H_2_-4), 1.44 (2H, m, H_2_-5), 1.23–1.34 (24 × H, bm), 0.88 (3H, t, *J* = 7.5 Hz, H_3_-18); ^13^C NMR (125 MHz, CD3OD) δ 79.7 (C, C-2), 77.1 (CH, C-1), 43.7 (CH, C-3), 34.5 (CH_2_, C-4), 32.7 (CH_2_, C-16), 30.5, 30.4, 30.3, 30.1, 29.7 (9 × CH_2_, C-7 to C-15), 26.1 (CH_2_, C-6), 23.4 (CH_2_, C-5), 21.0 (CH_2_, C-17), 14.1 (CH_3_, C-18); HRESIMS [M + H]^+^, *m/z* 266.2847, (calcd for C_18_H_36_N, 266.2848).

*3-Methoxyoctadec-1-yne (rac*-**38***)*: Amorphous white solid; UV (MeOH) λ_max_ (log ε) 201 (2.32) nm; IR (ATR Diamond) *ν*_max_ 3310, 2921, 2852, 2360, 2341, 1735, 1464 cm^−1^; ^1^H NMR (500 MHz, CDCl_3_) δ 3.92 (1H, td, *J* = 6.6, 2.0 Hz, H-3), 3.41 (3H, s, -CH(C**H**3)-), 2.43 (1H, d, *J* = 2.0 Hz, H-1), 1.71 (2H, m, H_2_-4), 1.44 (2H, qi, *J* = 6.5 Hz, H_2_-5), 1.22–1.31 (24 × H, bm), 0.88 (3H, t, *J* = 6.7 Hz, H_3_-18); ^13^C NMR (125 MHz, CDCl_3_) δ 79.8.1 (C, C-2), 73.6 (CH, C-1), 71.5 (CH, C-3), 56.4 (CH_3_, -OMe) 35.5 (CH_2_, C-4), 31.9 (CH_2_, C-16), 29.7, 29.6, 29.5, 29.4 (9 × CH_2_, C-7 to C-15), 29.3 (CH_2_, C-6), 25.1 (CH_2_, C-5), 22.7 (CH_2_, C-17), 14.1 (CH_3_, C-18), SMBEIGCMS *m/z* 280 ([M]^+^, 10), 265 (25), 251 (28), 249 (13), 219 (21), 205 (17), 191 (9), 177 (11), 163 (23), 149 (50), 135 (75), 121 (62), 108 (75), 96 (85), 84 (100), 80 (90), 69 (93), 55 (68). 

*Preparation of S-octadec-1-yn-3-yl ethanethioate (rac*-**39***)* [25]. The solution of octadec-1-yn-3-yl 4-methylbenzenesulfonate (*rac*-**35**) (32.0 mg, 0.074 mmol) and one drop of triethylamine were reacted with thioacetic acid (9 μL, 0.13 mmol) in DCM (1 mL). The reaction mixture was stirred for 12 h at rt and then water (1 mL) was added. The organic layer was separated, removed under reduced pressure and the residue dissolved PE. The crude reaction mixture was purified by column chromatography on silica gel, with a 2% step gradient from PE to 4:1 PE/EtOAc to afford *S*-octadec-1-yn-3-yl ethanethioate (*rac*-**39**) (20.7 mg, 0.064 mmol, 86%) as an amorphous white solid. 

*S-Octadec-1-yn-3-yl ethanethioate (rac-**39**):* Amorphous white solid; UV (MeOH) λ_max_ (log ε) 202 (2.74) nm; IR (ATR Diamond) *ν*_max_ 3312, 2923, 2853, 1698, 1459 cm^−1^; ^1^H NMR (500 MHz, CDCl_3_) δ 4.25 (1H, td, *J* = 6.9, 2.4 Hz, H-3), 2.34 (3H, s, -CH(SCOC**H**_3_)-), 2.28 (1H, d, *J* = 2.4 Hz, H-1), 1.75 (2H, q, *J* = 7.1 Hz, H_2_-4), 1.49 (2H, qi, *J* = 7.4 Hz, H_2_-5), 1.22–1.31 (24 × H, bm), 0.88 (3H, t, *J* = 6.7 Hz, H_3_-18); ^13^C NMR (125 MHz, CDCl_3_) δ 194.0 (C, -SCOMe) 82.7 (C, C-2), 71.3 (CH, C-1), 35.4 (CH, C-3), 33.6 (CH_2_, C-4), 31.9 (CH_2_, C-16), 30.3 (CH_3__,_ -SCO**C**H_3_), 29.7, 29.6, 29.5, 29.4, 29.3 (9 × CH_2_, C-7 to C-15), 29.0 (CH_2_, C-6), 26.9 (CH_2_, C-5), 22.7 (CH_2_, C-17), 14.1 (CH_3_, C-18); ESIMS: [M + H]^+^, *m/z* 325.3.

*Hydrolysis of thioacetate to prepare Octadec-1-yne-3-thiol (rac*-**40***)*. The solution of *S*-octadec-1-yn-3-yl ethanethioate (*rac*-**39**) (11 mg, 0.034 mmol) in methanol was treated with aq. HCl (1M, 1 mL). The reaction was stirred at 60 °C and its progress was monitored by TLC (9:1 PE/EtOAc). The solvent was removed under reduced pressure and the residue was extracted with PE. Final purification of the crude reaction mixture was achieved by column chromatography on silica gel, with a 2% step gradient from PE to 4:1 PE/EtOAc to afford octadec-1-yne-3-thiol (*rac*-**40**) (6.0 mg, 0.021 mmol, 62%) as an amorphous white solid. 

*Octadec-1-yne-3-thiol (rac*-**40***)*: Amorphous white solid; UV (MeOH) λ_max_ (log ε) 202 (2.63) nm; IR (ATR Diamond) *ν*_max_ 3311, 2923, 2853, 2361, 2341, 1490 cm^−1^; ^1^H NMR (400 MHz, CDCl_3_) δ 3.56(1H, q, *J* = 8.5 Hz, H-3), 2.35 (1H, s, H-1), 2.15 (1H, d, *J* = 8.5 Hz, -CH(S**H**)-), 1.75 (2H, m, H_2_-4), 1.50 (2H, qi, *J* = 8.5 Hz, H_2_-5), 1.23–1.33 (24 × H, bm), 0.88 (3H, t, *J* = 7.0 Hz, H_3_-18); ^13^C NMR (125 MHz, CDCl_3_ + CD3OD) δ 85.5, 71.3, 39.2, 32.0, 29.7, 29.6, 29.5, 29.0, 28.5, 27.1, 22.7, 14.1.

*General procedure for production the alkylaryl-dioxolanes***43***, and***46**. Excess magnesium was crushed in a glovebox and transferred into a reactor containing 2 mL of dry THF. Then 19 µL (0.126 mmol) of 2-(3-bromophenyl)-1,3-dioxolane was added and the reactor was sealed under a nitrogen atmosphere. The reaction was stirred for 2h at 65 °C until the colorless reaction mixture changed color to yellow (indicating the formation of the magnesium-bromide reagent). The reaction was cooled to room temperature and the yellow solution was transferred to another reactor containing 34 µL (0.112 mmol) of 1-bromotetradecane, 35 µL of HMPA and 3 mg of CuBr. The reactor was sealed under a nitrogen atmosphere and stirred for 12 h at 65 °C and then 1 mL of methanol was added. The solvent was removed under reduced pressure and the residue was partitioned between petroleum ether and water. The petroleum ether fraction was evaporated and the residue was purified by column chromatography on silica gel, with a 1% step gradient from PE to 9:1 PE/EA, affording 2-(3-tetradecylphenyl)-1,3-dioxolane (**43**) as colorless liquid 22.3 mg (0.064 mmol, 51%). The same procedure was used for the preparation of 2-(2-tetradecylphenyl)-1,3-dioxolane (**46**), as colorless liquid 21.0 mg (0.061 mmol, 48%), from 2-(2-bromophenyl)-1,3-dioxolane.

*2-(3-tetradecylphenyl)-1,3-dioxolane (***43***)*: Colorless liquid; ^1^H NMR (400 MHz, CDCl_3_) δ 7.28 (2H, m), 7.18 (2H, m), 5.79 (1H, s, -C**H**(OCH_2_CH_2_O)), 4.05–4.18 (4H, m, -CH(OC**H**_2_C**H**_2_O)), 2.61 (2H, t, *J* = 7.5 Hz, -C**H**2Ph-), 1.60 (2H, m, -C**H**2CH_2_Ph-), 1.22–1.35 (22H, bm), 0.88 (3H, t, *J* = 6.8 Hz, -CH_3_). 

*2-(2-tetradecylphenyl)-1,3-dioxolane (***46*)***: Colorless liquid; ^1^H NMR (500 MHz, CDCl_3_) δ 7.56 (1H, d, *J* = 7.3 Hz), 7.28 (1H, t, *J* = 7.3 Hz), 7.20 (2H, m), 6.01 (1H, s, -C**H**(OCH_2_CH_2_O)), 4.03–4.18 (4H, m, -CH(OC**H**_2_C**H**_2_O)), 2.72 (2H, t, *J* = 7.8 Hz, -C**H**_2_Ph-), 1.60 (2H, m, -C**H**_2_CH_2_Ph-), 1.22–1.39 (22H, bm), 0.89 (3H, t, *J* = 7.0 Hz, CH_3_); ^13^C NMR (125 MHz, CDCl_3_) δ 141.6 (C), 134.9 (C), 129.6 (CH), 129.0 (CH), 126.0 (CH), 125.8 (CH), 101.5 (CH, -**C**H(OCH_2_CH_2_O)), 65.3 (2 × CH_2_, -CH(O**C**H_2_**C**H_2_O)), 32.3 (CH_2_, -**C**H_2_Ph-), 31.9 (CH_2_, -**C**H_2_CH_2_CH_3_), 31.7 (CH_2_, -**C**H_2_CH_2_Ph-), 29.6 (9 × CH_2_), 22.7 (CH_2_, -**C**H_2_CH_3_), 14.1 (CH_3_, -CH_3_). 

*General procedure for the hydrolysis of the alkylaryl-dioxolanes 43, and 46 to the corresponding aldehydes***44***and***47**. 2-(3-tetradecylphenyl)-1,3-dioxolane (**43**) 22.3 mg (0.064 mmol) and 2-(2-tetradecylphenyl)-1,3-dioxolane **(46)** 21.0 mg (0.061 mmol) were dissolved, each, in 1 mL of dichlorometane and 0.4 mL of TFA in sealed reactors and allowed to stir at rt for 12 h. The solvent was removed under reduced pressure to afford 3-tetradecylbenzaldehyde (**44**) (9.6 mg, 0.032 mmol, 52%) and 2-tetradecylbenzaldehyde (**47**) (15.5 mg, 0.051 mmol, 80%).

*3-tetradecylbenzaldehyde (***44***)*: Amorphous white solid; ^1^H NMR (500 MHz, CDCl_3_) δ 10.00 (1H, s, -C**H**O), 7.70 (2H, m), 7.45 (2H, m), 2.67 (2H, t, *J* = 7.5 Hz, -C**H**_2_Ph-), 1.60 (2H, m, - C**H**_2_CH_2_Ph-), 1.22–1.35 (22H, bm), 0.88 (3H, t, *J* = 6.6 Hz, -CH_3_); ^13^C NMR (125 MHz, CDCl_3_) δ 192.7 (CH, -**C**HO), 144.0 (C), 136.5 (C), 134.7 (CH), 129.4 (CH), 128.9 (CH), 127.5 (CH), 35.7 (CH_2_, -**C**H2Ph-), 31.9 (CH_2_, -**C**H_2_CH_2_CH_3_), 31.2 (CH_2_, -**C**H_2_CH_2_Ph-), 29.6 (9 × CH_2_), 22.7 (CH_2_, -**C**H_2_CH_3_), 14.1 (CH_3_, -CH_3_). 

*2-tetradecylbenzaldehyde (***47***)*: Amorphous white solid; ^1^H NMR (500 MHz, CDCl_3_) δ 10.30 (1H, s, -C**H**O), 7.83 (1H, d, *J* = 7.2 Hz), 7.49 (1H, t, *J* = 7.2 Hz), 7.34 (1H, t, *J* = 7.2 Hz), 7.26 (1H, d, *J* = 7.2 Hz), 3.02 (2H, t, *J* = 7.5 Hz, -C**H**_2_Ph-), 1.59 (2H, qi, *J* = 7.5 Hz, -C**H**_2_CH_2_Ph-), 1.22–1.41 (22H, bm), 0.88 (3H, t, *J* = 7.0 Hz, -CH_3_); ^13^C NMR (125 MHz, CDCl_3_) δ 192.3 (CH, -**C**HO), 145.9 (C), 133.8 (CH), 133.7 (C), 131.2 (CH), 131.0 (CH), 126.4 (CH), 32.5 (CH_2_, -**C**H_2_Ph-), 32.5 (CH_2_, -**C**H_2_CH_2_Ph-), 31.9 (CH_2_, -**C**H_2_CH_2_CH_3_), 29.6 (9 × CH_2_), 22.7 (CH_2_, -**C**H_2_CH_3_), 14.1 (CH_3_, -CH_3_).

*1-(3-tetradecylphenyl)prop-2-yn-1-ol (rac-***45***)*: Amorphous white solid; UV (MeOH) λ_max_ (log ε) 203 (3.92) nm, 256 (2.73) nm, 285 (1.55) nm; IR (ATR Diamond) *ν*_max_ 3310, 2954, 2922, 2852, 2360, 2341, 1608, 1465 cm^−1^; ^1^H NMR (400 MHz, CDCl_3_) δ 7.37 (1H, m), 7.36 (1H, m), 7.30 (1H, *J* = 7.6 Hz), 7.16 (1H, d, *J* = 7.6 Hz), 5.45 (1H, dd, *J* = 6.4, 2.0 Hz, -C**H**(OH)-), 2.67 (1H, d, *J* = 2.0 Hz, **H**-C≡C-), 2.62 (2H, t, *J* = 7.0 Hz, -C**H**_2_Ph-), 2.17 (1H, d, *J* = 6.4 Hz, -O**H**), 1.60 (2H, qi, *J* = 7.8 Hz, -C**H**_2_CH_2_Ph-), 1.21–1.36 (22H, bm), 0.88 (3H, t, *J* = 7.2 Hz, -CH_3_); ^13^C NMR (100 MHz, CDCl_3_) δ 140.6 (C), 137.6 (C), 129.7 (CH), 128.7 (CH), 126.8 (CH), 126.3 (CH), 83.8 (C, HC≡**C**-), 74.7 (CH, H**C**≡C-), 64.6 (CH, -**C**H(OH)-), 35.9 (CH_2_, -**C**H_2_Ph-), 31.9 (CH_2_, -**C**H_2_CH_2_CH_3_), 31.5 (CH_2_, -**C**H_2_CH_2_Ph-), 29.6 (9 × CH_2_), 22.7 (CH_2_, -**C**H_2_CH_3_), 14.1 (CH_3_, -CH_3_); EIGCMS *m/z* 328 (11), 281 (7), 253 (7), 207 (21), 145 (24), 131 (100), 115 (13), 91 (28), 73 (11), 55 (13), 43 (18); HREIMS *m/z* 328.2763 M^+^ (calcd for C_23_H_36_O 328.2766).

*1-(2-tetradecylphenyl)prop-2-yn-1-ol (rac-***48***)*: Amorphous white solid; UV (MeOH) λ_max_ (log ε) 203 (3.97) nm, 264 (2.61) nm, 272 (2.56) nm; IR (ATR Diamond) *ν*_max_ 3309, 2954, 2921, 2852, 2360, 2341, 1465 cm^−1^; ^1^H NMR (400 MHz, CDCl_3_) δ 7.71 (1H, dd, *J* = 6.4, 2.0 Hz), 7.19–7.30 (3H, m), 5.68 (1H, s, -CH(OH)-), 2.72 (2H, m, -C**H**_2_Ph-), 2.62 (1H, d, *J* = 2.4 Hz, **H**-C≡C-), 2.13 (1H, bs, -O**H**), 1.62 (2H, qi, *J* = 8.0 Hz, -C**H**_2_CH_2_Ph-), 1.23–1.43 (22H, bm), 0.88 (3H, t, *J* = 6.4 Hz, -CH_3_); ^13^C NMR (125 MHz, CDCl_3_) δ 143.6 (C), 139.9 (C), 128.7 (CH), 128.6 (CH), 126.6 (CH), 123.9 (CH), 83.6 (C, HC≡**C**-), 74.6 (CH, H**C**≡C-), 61.6 (CH, -**C**H(OH)-), 32.3 (CH_2_, -**C**H_2_Ph-), 31.9 (CH_2_, -**C**H_2_CH_2_CH_3_), 31.5 (CH_2_, -**C**H_2_CH_2_Ph-), 29.6 (9 × CH_2_), 22.7 (CH_2_, -**C**H_2_CH_3_), 14.1 (CH_3_, -CH_3_); EIGCMS *m/z* 328 (1), 310 (12), 183 (12), 169 (28), 155 (32), 142 (100), 128 (77), 115 (38), 91 (30), 55 (14), 43 (23); HREIMS *m/z* 328.2769 M^+^ (calcd for C_23_H_36_O 328.2766).

*Cytotoxicity structure-activity relationship analyses of compounds in NSCLC tumor cells and fibroblasts*. Stock solutions (10 mg/mL) of the compounds to be tested were made in DMSO and further diluted with cell culture media prior to structure-activity relationship (SAR) screening as earlier described [17]. To assess anti-tumor activity, the large cell lung carcinoma cell line NSCLC U-1810 (kind gift from Uppsala University) [29] and diploid lung fibroblasts WI-38 [30] were used. U-1810 cells were propagated in RPMI-1640 medium (Sigma-Aldrich, St. Louis, MI, USA) supplemented with FBS (10% HyClone) and l-glutamine (2 mmol/L, Invitrogen) while WI-38 fibroblasts were cultured in Eagle’s Minimum Essential Medium (Sigma-Aldrich) with 15% FBS and l-glutamine. For SAR assessment the cytotoxic assay 3-(4,5-dimethylthiazol-2-yl)-2,5 diphenyltetrazolium bromide (MTT) was applied [8]. Profiling of U-1810 cells was carried out at 80% confluency (obtained by seeding 5000 cells/well in a 96-well plate for 24 h prior to treatment) and of WI-38 at 100% confluency (obtained by seeding 18,000 cells/well in a 96-well plate for 24 h prior to compound addition), the later to mimic the behavior of fibroblasts in the human body where they are non-dividing. For SAR evaluation, different concentrations of the compounds were added to fresh media while untreated cells were treated with DMSO corresponding to the amount applied when testing the highest concentration of the different compounds. The cytotoxicity of the compounds was assessed at 72 h post addition to cells by adding MTT solution (0.5 mg/mL, Sigma-Aldrich, 4 h at 37 °C) and dissolving the resulting formazan crystals in an SDS-containing solution (10% SDS and 0.01 mol/L HCl). Absorbance was quantified at 595 nm and the compound-induced cytotoxicity was calculated relative to that observed in DMSO-treated cells. The IC_50_ values were deduced from survival plots drawn in Graph Pad PRISM software vers.6 and for some of the compounds extrapolated as indicated in footnotes to Table 1. The cell survival curves for some of the compounds are shown in Figure S188. 

## 4. Conclusions

The alkyl-4*E*-ene-3-ol-1-ynes that were isolated from the extract of the sponge were composed of different mixtures of 3*R- and 3*S-isomers, where the 3*R*-isomers dominated. The fact that the carbocation at position-3 is rather stabilized by surrounding double- and triple-bonds, and that the 3-ol-1-ynes isolated in this study were composed solely of the 3*R*-isomer, suggests that the compounds are biosynthesized by the sponge as the 3*R*-isomers and were racemized along the isolation process. Furthermore, the alkyl-3*Z*-en-5-ol-1-ynes isolated in this study were all racemic. This group of compounds is believed to be derived from the migration of the carbocation formed at position-3, to position-5, and its quenching by water to give the racemic 5-alcohol. This suggestion is in line with the finding in this study that the 3*R*-isomers are more active than the 3*S*-isomers. The results of the cytotoxic activity of the natural and synthetic compounds revealed that the terminal acetylene and the alcohol at position 3 (with the 3*R*-configuration) are essential for the potency of these alkylynols and resulted in two orders of magnitude selectivity toward the NSCLC cell line over normal fibroblasts, i.e., **7**, *R*-**32**, *rac*-**32** and *rac*-**33**. The length of the alkyl chain seems to influence the activity where a short alkyl chain (*rac*-**31**), or the absence of a chain (*rac-***49**) presented reduced cytotoxicity toward the NSCLC cell line relative to *rac*-**32** and *rac*-**33**. The addition of an *E*-4,5-double bond to the alkylynol skeleton results in equally potent but less selective derivatives **1**, **8**, *rac*-**18**, *R*-**18**, **19**, **20**, **22** and **23**, toward the tumor cell line. Any substitution of the 3-OH with another electronegative substituent resulted in much less potent cytotoxicity in the tested NSCLC cell line, i.e., *rac-***35** to *rac-***40**. Substitution of the acetylene (at C-1) or the carbinol (at C-3) by a methyl group results in essentially non-active products (**41** and **42**). The presence of a 4*E*-double bond, in *rac*-**18**, results in equipotent products but with less selective cytotoxicity towards the tested tumor cell line, relative to the presence of 4*E*,6*E-*diene, in *rac*-**27**, or saturated chain, in *rac*-**32**. However, the introduction of a less flexible moiety, a phenyl, to 4,5-bond (*rac-***45** and *rac-***48**) resulted in one order of magnitude less potent cytotoxicity in the examined NSCLC cell line.

## Figures and Tables

**Table 1:** Cytotoxicity analyses of the natural products and synthetic compounds. The IC_50_ values were estimated from the cell viability curve and is given in µM. ^a^ The selectivity of compound in NSCLC cells (U-1810) vs. diploid fibroblasts (WI-38) was calculated at IC_50_ if not otherwise indicated.

**Compound Number**	**Compound Structure**	**U-1810 IC** **_50_**	**WI-38 IC** **_50_**	**Selectivity**
**1**	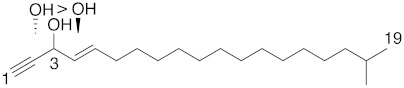	1	12	12
**6**	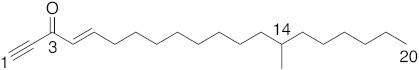	65	73	1.1
**7**	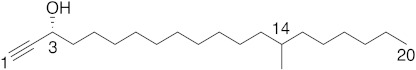	3	>163 ^b^	54 ^b^
**8**	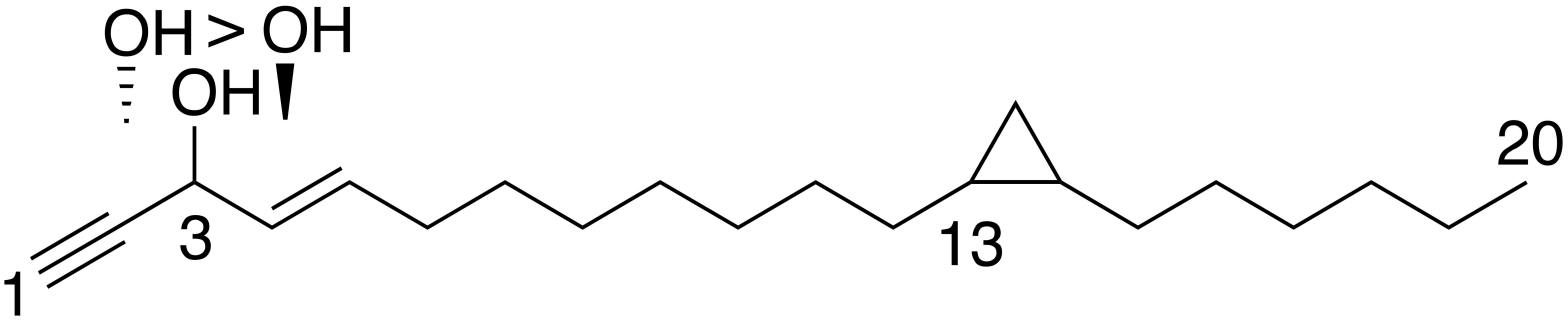	1	10	10
**10**	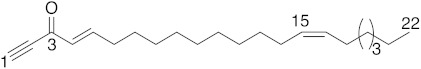	~80	30	~0.4
*scal*-**18**	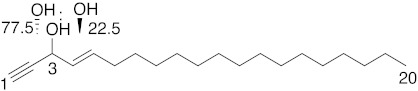	0.5^c^	7.3 ^d^	14.6
*rac*-**18**	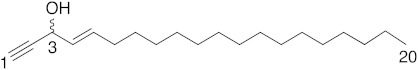	1.2	8.3	6.9
*R*-**18**	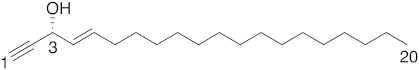	0.9	3.4	3.8
*scal-R*-**18**	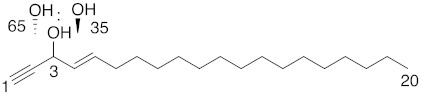	1	6.8	6.8
**Compound number**	**Compound structure**	**U-1810 IC** **_50_**	**WI-38 IC** **_50_**	**Selectivity**
*scal-S*-**18**	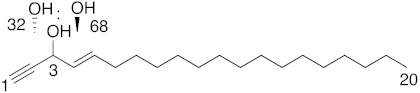	1	8.5	8.5
**19**	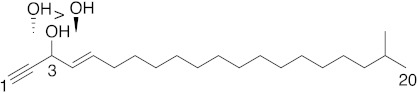	2	13	6.5
**20**	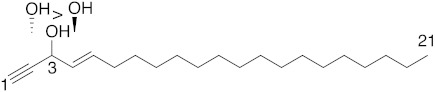	1.4	14	10
**22**	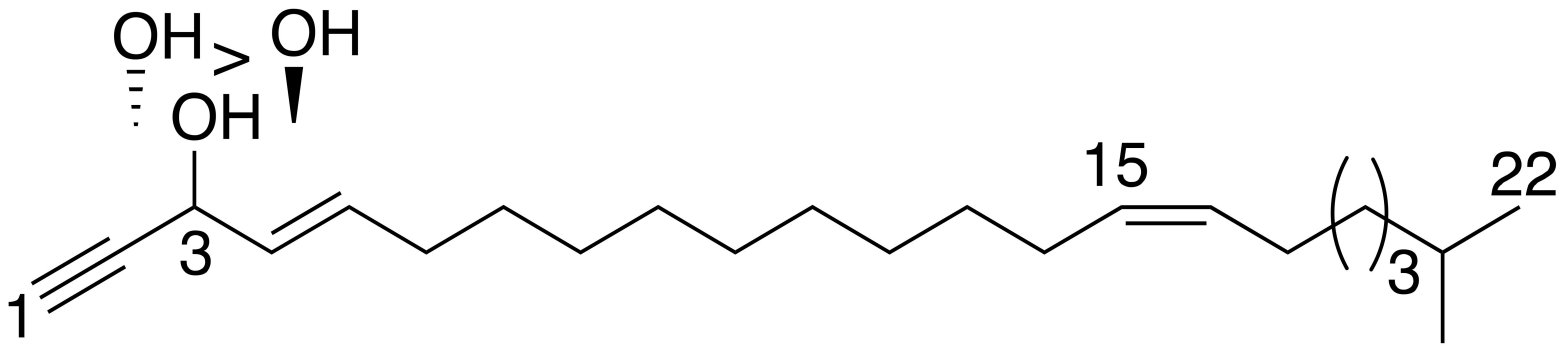	1.2	13	10.8
**23**	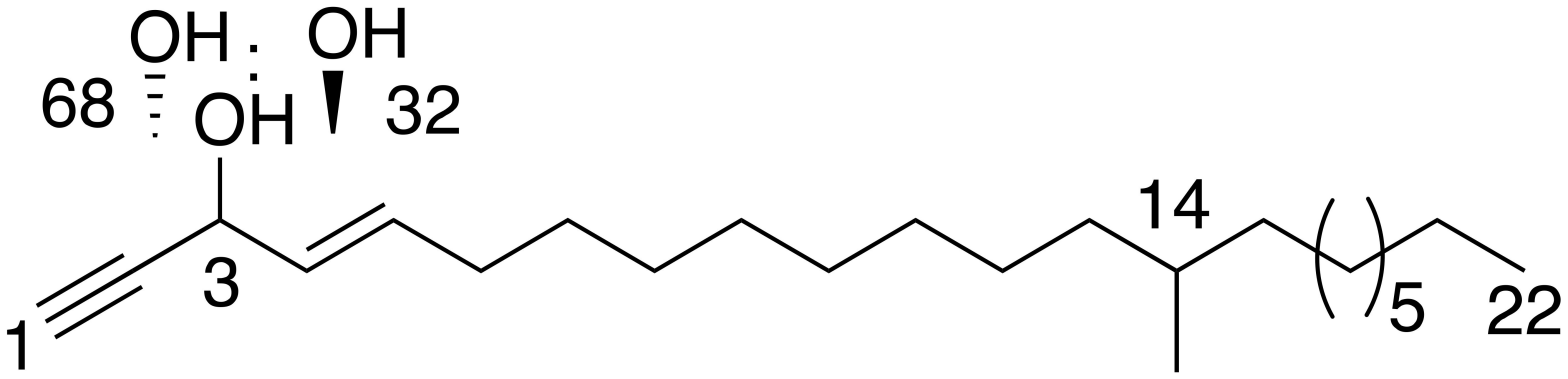	0.8 ^e^	9.7 ^f^	12.1
*rac*-**27**	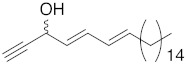	~2.4	70	29.2
*rac*-**31**	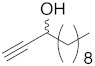	15	ND ^g^	ND ^g^
*rac*-**32**	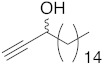	3	75	25
*R*-**32**	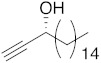	1.4	>188 ^h^	134 ^i^
*S*-**32**	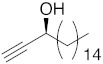	140	>188 ^j^	1.3 ^k^
*rac*-**33**	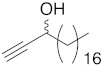	2.8	>170 ^l^	67 ^m^
*R,R*-**34**	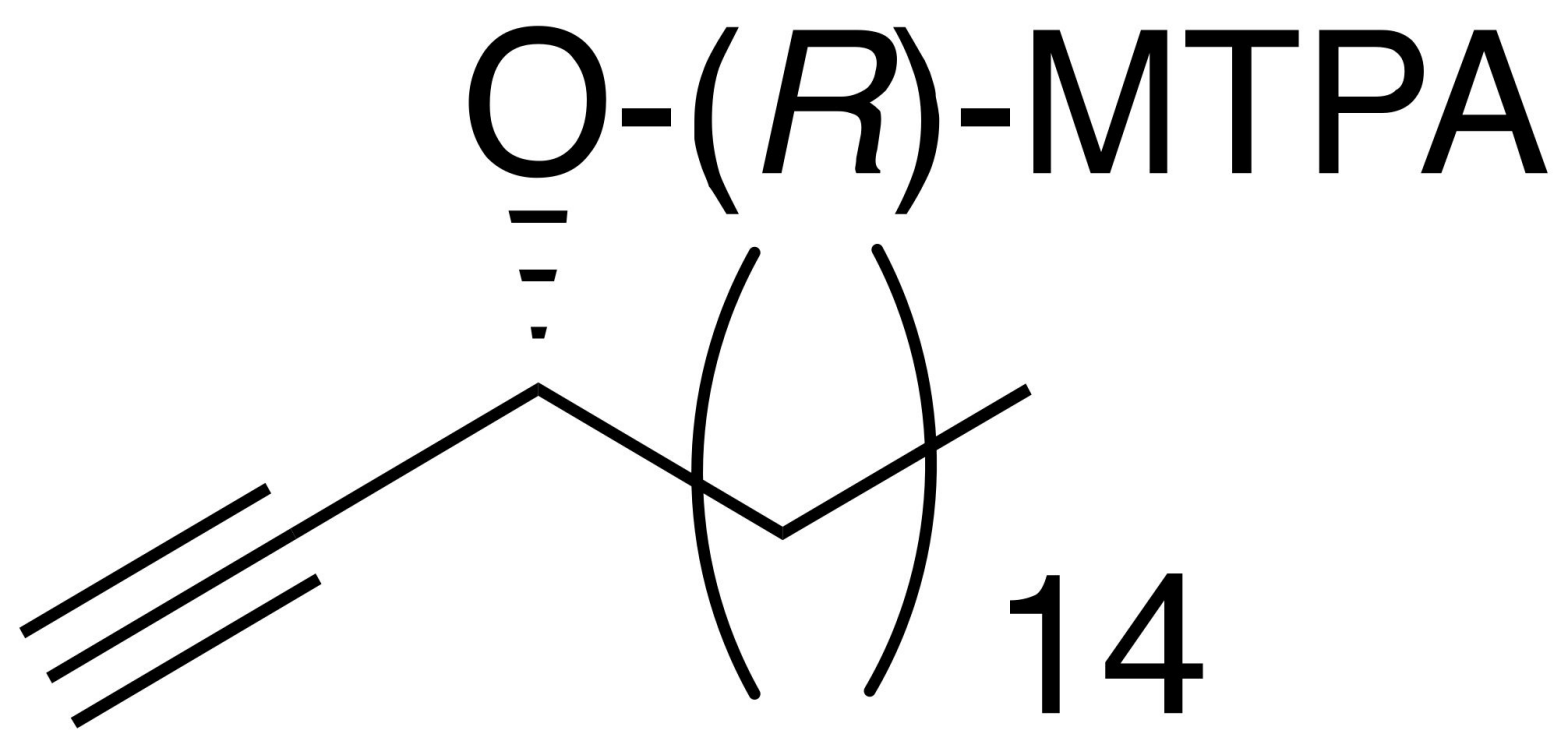	>113 ^n^	>113 ^o^	ND ^g^
*S,R*-**34**	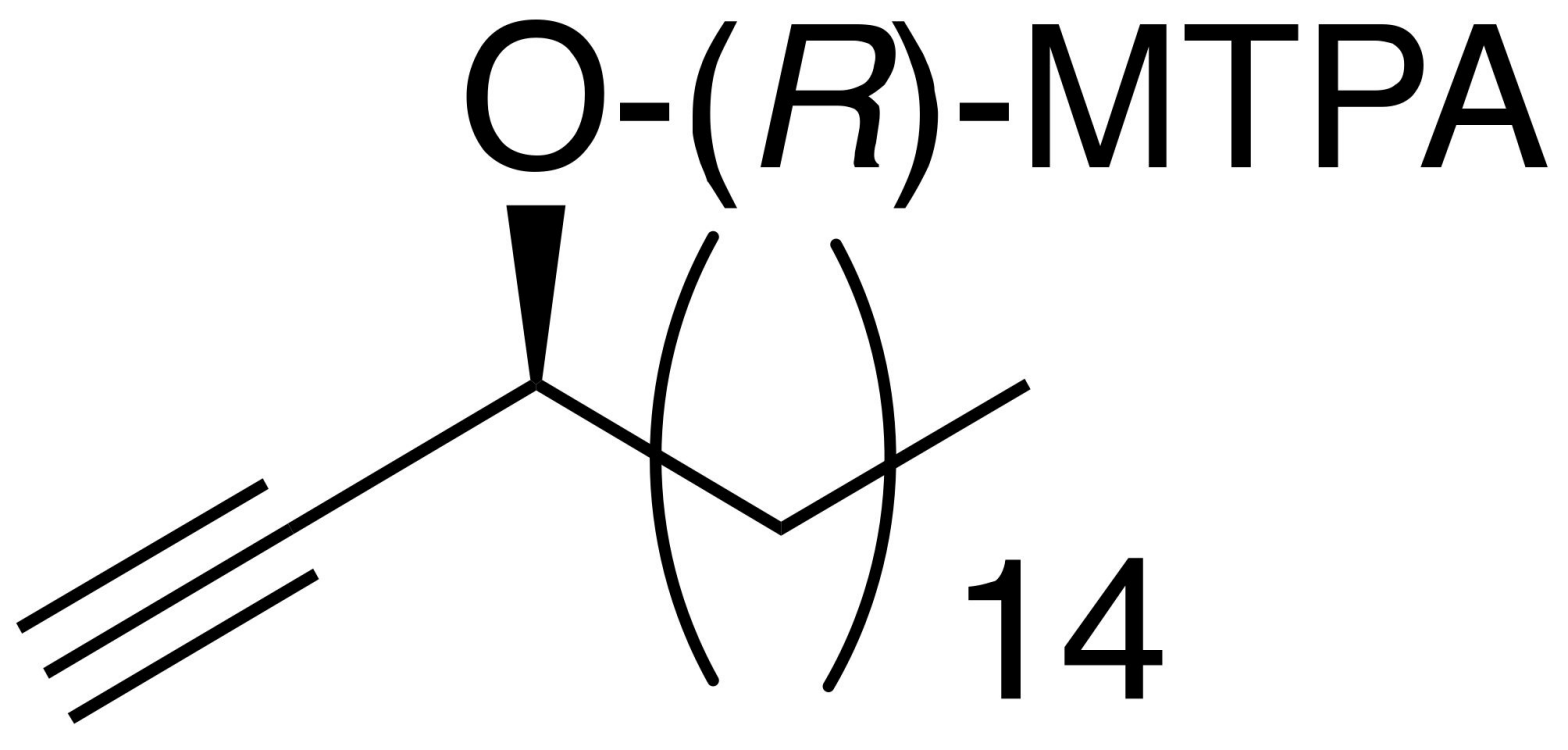	>113 ^p^	>113 ^q^	ND ^g^
*rac*-**35**	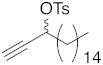	23	>119 ^r^	>5.2 ^s^
**Compound number**	**Compound structure**	**U-1810 IC** **_50_**	**WI-38 IC** **_50_**	**Selectivity**
*rac*-**36**	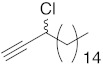	140	>175 ^t^	>1.3 ^u^
*rac*-**37**	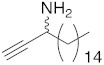	29	34	1.2
*rac*-**38**	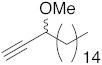	>179 ^v^	>179 ^x^	ND ^g^
*rac*-**39**	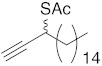	60	>154 ^y^	2.6 ^z^
*rac*-**40**	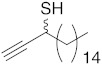	>177 ^aa^	>177 ^aa^	ND ^g^
*rac*-**41**	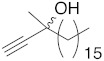	>162 ^ab^	ND ^ac^	NA
*rac*-**42**	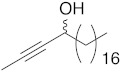	>162 ^ad^	ND ^ac^	NA
*rac*-**45**	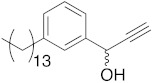	~152 ^ae^	ND ^ac^	NA
*rac*-**48**	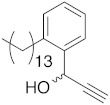	~50 ^af^	ND ^ac^	NA
*rac*-**49**	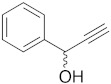	>379 ^ag^	ND ^ac^	NA

^a^ The cytotoxicity was evaluated on the human non-small cell lung carcinoma (NSCLC) cell line U-1810 and on WI-38, a diploid fibroblast cell line established from the lung. IC_50_ doses were estimated from cell viability survival curves over a range of doses of each compound. The entire dose response curves of compounds in the two cell types are presented in Figure S188. ^b^ IC_50_ was not reached. The highest dose tested did not give any cytotoxicity. The selectivity was calculated at IC_50_ of U-1810 and the highest dose applied for WI-38. ^c^ Data taken from reference [17]: IC_50_ 1.5 μM (24 h), 1.1 μM (48 h), 0.5 μM (72 h); ^d^ Data taken from reference [17]: IC_50_ 10.0 μM (24 h), 6.0 μM (48 h), 7.3 μM (72 h); ^e^ Data taken from reference [17]: IC_50_ 15.1 μM (24 h), 2.4 μM (48 h), 0.8 μM (72 h); ^f^ Data taken from reference [17]: IC_50_ 31.5 μM (24 h), 21.2 μM (48 h), 9.7 μM (72 h); ^g^ Not determined; ^h^ IC_50_ not reached at 188 µM, cell viability ~60%; ^i^ Selectivity calculated at IC_50_ of U-1810 and with data from ^h^; ^j^ IC_50_ not reached at 188 µM, cell viability ~80%; ^k^ Selectivity calculated at IC_50_ of U-1810 and with data from ^j^; ^l^ IC_50_ not reached at 170 µM, cell viability ~100%; ^m^ Selectivity calculated at IC_50_ of U-1810 and with data from ^l^; ^n,o^ IC_50_ not reached at 113 µM, cell viability~100%; ^p^ IC_50_ not reached at 113 µM, cell viability~96%; ^q^ IC_50_ not reached at 113 µM, cell viability ~100%; ^r^ IC_50_ not reached at 119 µM, cell viability ~85%; ^s^ Selectivity was calculated at IC_50_ of U-1810 and the highest dose applied in WI-38. ^t^ IC_50_ not reached at 175 µM, cell viability ~75%; ^u^ Selectivity was calculated at IC_50_ of U-1810 and the highest dose applied in WI-38. ^v^ IC_50_ not reached at 179 µM, cell viability ~71%; ^x^ IC_50_ not reached at 179 µM, cell viability ~77%; ^y^ IC_50_ not reached at 154 µM, cell viability ~85%; ^z^ Selectivity was calculated as in ^s^; ^aa^ IC_50_ not reached at 177 µM, cell viability ~100%; ^ab^ IC_50_ not reached at 162 µM, cell viability~54%; ^ac^ Effect on WI-38 cell viability was not determined (ND); ^ad^ IC_50_ not reached at 162 µM, cell viability ~90%; ^ae^ Estimated from one experiment with triplicate samples for some of the doses; at 76 µM cell viability was about 70%; ^af^ Estimated from one experiment with triplicate samples; ^ag^ IC_50_ not reached at 379 µM, cell viability ~80%.

## Data Availability

Not applicable.

## Supplementary Materials

The following supporting information can be downloaded at: https://www.mdpi.com/article/10.3390/md20040265/s1, ^1^D (^1^H, ^13^C) and 2D NMR (HSQC, HMBC, COSY, ROESY) spectra and HR MS data of compounds **1**–**17**, tables of full NMR data of **1**–**17** and two figures with the structures of the known metabolites isolated in this study.
