# Supplementary material for: Cytotoxic Alkylynols of the Sponge Cribrochalina vasculum: Structure, Synthetic Analogs and SAR Studies
Source: Mar Drugs. 2022 Apr 13;20(4):265. doi: 10.3390/md20040265 (PMC9032987; doi:10.3390/md20040265)
Supplement: Supplementary file 1 [file marinedrugs-20-00265-s001.zip › marinedrugs-1626032-supplementary.pdf]

# Supplementary Material

# Cytotoxic Alkylynols of the Sponge *Cribrochalina vasculum*: Structure, Synthetic Analogs and SAR Studies

**Dimitry Kovalerchik [1], Ana Zovko [2], Petra Hååg [2], Adam Sierakowiak [2], Kristina Viktorsson [2], Rolf Lewensohn [2,3] and Micha Ilan [4], Shmuel Carmeli [1,*]**

[1] Raymond and Beverly Sackler Faculty of Exact Sciences, School of Chemistry, Tel Aviv University,
Tel Aviv, 69978, Israel; kovaler310@gmail.com
[2] Department of Oncology-Pathology, Karolinska Institutet, SE-171 64, Solna, Sweden; zovko.ana@gmail.com (A.Z.); petra.haag@ki.se (P.H.); adam.sie.se@gmail.com (A.S.); kristina.viktorsson@ki.se (K.V.); rolf.lewensohn@ki.se (R.L.)
[3] Theme Cancer, Medical Unit Head and Neck, Lung and Skin Tumors, Thoracic Oncology Center, Karolinska University Hospital, SE-171 64, Solna, Sweden
[4] Department of Zoology, George S. Wise Faculty of Life Sciences, Tel Aviv University, Tel Aviv, 69978, Israel
* Correspondence: carmeli@tauex.tau.ac.il; Tel.: +972-3-6408550

## Table of Content


| Page | Caption |
|---|---|
| 8 | Figure S1. $^1$H NMR spectrum of (3*R*)-18-methylnonadec-(4*E*)-en-1-yn-3-ol (**1**) in $CDCl_3$ |
| | Figure S2. $^{13}$C NMR spectrum of (3*R*)-18-methylnonadec-(4*E*)-en-1-yn-3-ol (**1**) in $CDCl_3$ |
| 9 | Figure S3. HSQC spectrum of (3*R*)-18-methylnonadec-(4*E*)-en-1-yn-3-ol (**1**) in $CDCl_3$ |
| | Figure S4. HMBC spectrum of (3*R*)-18-methylnonadec-(4*E*)-en-1-yn-3-ol (**1**) in $CDCl_3$ |
| 10 | Figure S5. COSY spectrum of (3*R*)-18-methylnonadec-(4*E*)-en-1-yn-3-ol (**1**) in $CDCl_3$ |
| | Figure S6. DEPT spectrum of (3*R*)-18-methylnonadec-(4*E*)-en-1-yn-3-ol (**1**) in $CDCl_3$ |
| 11 | Table S1. NMR data of (3*R*)-18-methylnonadec-(4*E*)-en-1-yn-3-ol (**1**) in $CDCl_3$. |
| 12 | Figure S7. GCMS spectrum of (3*R*)-18-methylnonadec-(4*E*)-en-1-yn-3-ol (**1**) |
| | Figure S8. HRMS spectrum of (3*R*)-18-methylnonadec-(4*E*)-en-1-yn-3-ol (**1**) |
| 13 | Figure S9. $^1$H NMR spectrum of (3*R*)-14-methylnonadec-(4*E*)-en-1-yn-3-ol (**2**) in $CDCl_3$ |
| | Figure S10. $^{13}$C NMR spectrum of (3*R*)-14-methylnonadec-(4*E*)-en-1-yn-3-ol (**2**) in $CDCl_3$ |
| 14 | Figure S11. HSQC spectrum of (3*R*)-14-methylnonadec-(4*E*)-en-1-yn-3-ol (**2**) in $CDCl_3$ |
| | Figure S12. HMBC spectrum of (3*R*)-14-methylnonadec-(4*E*)-en-1-yn-3-ol (**2**) in $CDCl_3$ |
| 15 | Figure S13. COSY spectrum of (3*R*)-14-methylnonadec-(4*E*)-en-1-yn-3-ol (**2**) in $CDCl_3$ |
| 16 | Table S2. NMR data of (3*R*)-14-methylnonadec-(4*E*)-en-1-yn-3-ol (**2**) in $CDCl_3$ |
| 17 | Figure S14. GCMS spectrum of (3*R*)-14-methylnonadec-(4*E*)-en-1-yn-3-ol (**2**) |
| 18 | Figure S15. HRMS spectrum of (3*R*)-14-methylnonadec-(4*E*)-en-1-yn-3-ol (**2**) |
| 19 | Figure S16. $^1$H NMR spectrum of 14-methylnonadec-(4*E*)-en-1-yn-3-one (**3**) in $CDCl_3$ |
| | Figure S17. $^{13}$C NMR spectrum of 14-methylnonadec-(4*E*)-en-1-yn-3-one (**3**) in $CDCl_3$ |
| 20 | Table S3. NMR data of 14-methylnonadec-(4*E*)-en-1-yn-3-one (**3**) in $CDCl_3$ |
| 21 | Figure S18. EIMS and fragmentation pattern of 14-methylnonadec-(4*E*)-en-1-yn-3-one (**3**) |

| | |
|---|---|
| 22 | Figure S19. HRCIMS and fragmentation pattern of 14-methylnonadec-(4*E*)-en-1-yn-3-one (**3**) |
| 23 | Figure S20. $^{1}$H NMR spectrum of (3*R*)-13,18-dimethylnonadec-(4*E*)-en-1-yn-3-ol (**4**) in $CDCl_3$ |
| | Figure S21. $^{13}$C NMR spectrum of (3*R*)-13,18-dimethylnonadec-(4*E*)-en-1-yn-3-ol (**4**) in $CDCl_3$ |
| 24 | Table S4. NMR data of (3*R*)-13,18-dimethylnonadec-(4*E*)-en-1-yn-3-ol (**4**) in $CDCl_3$ |
| 25 | Figure S22. Measured and calculated $^{13}$C NMR data of (3*R*)-13,18-dimethylnonadec-(4*E*)-en-1-yn-3-ol (**4**) |
| 26 | Figure S23. EIMS and fragmentation pattern of (3*R*)-13,18-dimethylnonadec-(4*E*)-en-1-yn-3-ol (**4**) |
| 27 | Figure S24. HRMS and fragmentation pattern of (3*R*)-13,18-dimethylnonadec-(4*E*)-en-1-yn-3-ol (**4**) |
| 28 | Figure S25. $^{1}$H NMR spectrum of (3*R*)-14-methylicos-(4*E*)-en-1-yn-3-ol (**5**) in $CDCl_3$ |
| | Figure S26. $^{13}$C NMR spectrum of (3*R*)-14-methylicos-(4*E*)-en-1-yn-3-ol (**5**) in $CDCl_3$ |
| 29 | Figure S27. HSQC spectrum of (3*R*)-14-methylicos-(4*E*)-en-1-yn-3-ol (**5**) in $CDCl_3$ |
| | Figure S28. HMBC spectrum of (3*R*)-14-methylicos-(4*E*)-en-1-yn-3-ol (**5**) in $CDCl_3$ |
| 30 | Figure S29. COSY spectrum of (3*R*)-14-methylicos-(4*E*)-en-1-yn-3-ol (**5**) in $CDCl_3$ |
| 31 | Table S5. NMR data of (3*R*)-14-methylicos-(4*E*)-en-1-yn-3-ol (**5**) in $CDCl_3$ |
| 32 | Figure S30. EIMS and fragmentation pattern of (3*R*)-14-methylicos-(4*E*)-en-1-yn-3-ol (**5**) |
| 33 | Figure S31. HRMS and fragmentation pattern of (3*R*)-14-methylicos-(4*E*)-en-1-yn-3-ol (**5**) |
| 34 | Figure S32. $^{1}$H NMR spectrum of 14-methylicos-(4*E*)-en-1-yn-3-one (**6**) in $CDCl_3$ |
| | Figure S33. $^{13}$C NMR spectrum of 14-methylicos-(4*E*)-en-1-yn-3-one (**6**) in $CDCl_3$ |
| 35 | Table S6. NMR data of 14-methylicos-(4*E*)-en-1-yn-3-one (**6**) in $CDCl_3$ |
| 36 | Figure S34. EIMS and fragmentation pattern of 14-methylicos-(4*E*)-en-1-yn-3-one (**6**) |
| 37 | Figure S35. HRCIMS and fragmentation pattern of 14-methylicos-(4*E*)-en-1-yn-3-one (**6**) |
| 38 | Figure S36. $^{1}$H NMR spectrum of (3*R*)-14-methylicos-1-yn-3-ol (**7**) in $CDCl_3$ |
| | Figure S37. $^{13}$C NMR spectrum of (3*R*)-14-methylicos-1-yn-3-ol (**7**) in $CDCl_3$ |
| 39 | Figure S38. HSQC spectrum of (3*R*)-14-methylicos-1-yn-3-ol (**7**) in $CDCl_3$ |
| | Figure S39. HMBC spectrum of (3*R*)-14-methylicos-1-yn-3-ol (**7**) in $CDCl_3$ |
| 40 | Figure S40. COSY spectrum of (3*R*)-14-methylicos-1-yn-3-ol (**7**) in $CDCl_3$ |
| | Figure S41. DEPT spectrum of (3*R*)-14-methylicos-1-yn-3-ol (**7**) in $CDCl_3$ |
| 41 | Table S7. NMR data of (3*R*)-14-methylicos-1-yn-3-ol (**7**) in $CDCl_3$ |
| 42 | Figure S42. EIMS and fragmentation pattern of (3*R*)-14-methylicos-1-yn-3-ol (**7**) |
| | Figure S43. HRMS and fragmentation pattern of (3*R*)-14-methylicos-1-yn-3-ol (**7**) |
| 43 | Figure S44. $^{1}$H NMR spectrum of (3*R*,*E*)-12-*cis*-(2-hexylcyclopropyl)dodec-4-en-1-yn-3-ol (**8**) in $CDCl_3$ |
| | Figure S45. $^{13}$C NMR spectrum of (3*R*,*E*)-12-*cis*-(2-hexylcyclopropyl)dodec-4-en-1-yn-3-ol (**8**) in $CDCl_3$ |
| 44 | Figure S46. HSQC spectrum of (3*R*,*E*)-12-*cis*-(2-hexylcyclopropyl)dodec-4-en-1-yn-3-ol (**8**) in $CDCl_3$ |
| | Figure S47. HMBC spectrum of (3*R*,*E*)-12-*cis*-(2-hexylcyclopropyl)dodec-4-en-1-yn-3-ol (**8**) in $CDCl_3$ |
| 45 | Figure S48. COSY spectrum of (3*R*,*E*)-12-*cis*-(2-hexylcyclopropyl)dodec-4-en-1-yn-3-ol (**8**) in $CDCl_3$ |
| | Figure S49. DEPT spectrum of (3*R*,*E*)-12-*cis*-(2-hexylcyclopropyl)dodec-4-en-1-yn-3-ol (**8**) in $CDCl_3$ |
| 46 | Table S8. NMR data of (3*R*,*E*)-12-*cis*-(2-hexylcyclopropyl)dodec-4-en-1-yn-3-ol (**8**) in $CDCl_3$ |
| 47 | Figure S50. Measured and calculated $^{13}$C NMR data of (3*R*,*E*)-12-*cis*-(2-hexylcyclopropyl)dodec-4-en-1-yn-3-ol (**8**) |
| 48 | Figure S51. EIMS of (3*R*,*E*)-12-*cis*-(2-hexylcyclopropyl)dodec-4-en-1-yn-3-ol (**8**) |
| | Figure S52. HRMS of (3*R*,*E*)-12-*cis*-(2-hexylcyclopropyl)dodec-4-en-1-yn-3-ol (**8**) |

| | |
|---|---|
| 49 | Figure S53a. Proposed fragmentation pattern of the EIMS parent ion of (3*R*,*E*)-12-*cis*-(2-hexylcyclopropyl)dodec-4-en-1-yn-3-ol (**8**) |
| | Figure S53b. Proposed fragmentation pattern of the EIMS water elimination product ion of (3*R*,*E*)-12-*cis*-(2-hexylcyclopropyl)dodec-4-en-1-yn-3-ol (**8**) |
| 50 | Figure S54. $^1$H NMR spectrum of (3*R*)-13-methylhenicos-(4*E*)-en-1-yn-3-ol (**9**) in $CDCl_3$ |
| | Figure S55. $^{13}$C NMR spectrum of (3*R*)-13-methylhenicos-(4*E*)-en-1-yn-3-ol (**9**) in $CDCl_3$ |
| 51 | Table S9. NMR data of (3*R*)-13-methylhenicos-(4*E*)-en-1-yn-3-ol (**9**) in $CDCl_3$ |
| 52 | Figure S56. EIMS and fragmentation pattern of (3*R*)-13-methylhenicos-(4*E*)-en-1-yn-3-ol (**9**) |
| | Figure S57. HRMS of (3*R*)-13-methylhenicos-(4*E*)-en-1-yn-3-ol (**9**) |
| 53 | Figure S58. $^1$H NMR spectrum of docos-(4*E*,15*Z*)-dien-1-yn-3-one (**10**) in $CDCl_3$ |
| | Figure S59. $^{13}$C NMR spectrum of docos-(4*E*,15*Z*)-dien-1-yn-3-one (**10**) in $CDCl_3$ |
| 54 | Figure S60. HSQC spectrum of docos-(4*E*,15*Z*)-dien-1-yn-3-one (**10**) in $CDCl_3$ |
| | Figure S61. HMBC spectrum of docos-(4*E*,15*Z*)-dien-1-yn-3-one (**10**) in $CDCl_3$ |
| 55 | Figure S62. COSY spectrum of docos-(4*E*,15*Z*)-dien-1-yn-3-one (**10**) in $CDCl_3$ |
| 56 | Table S10. NMR data of docos-(4*E*,15*Z*)-dien-1-yn-3-one (**10**) in $CDCl_3$ |
| 57 | Figure S63. EIMS and fragmentation pattern of docos-(4*E*,15*Z*)-dien-1-yn-3-one (**10**) |
| 58 | Figure S64. LCMS chromatogram and mass spectrum of the periodate-permanganate oxidation products of docos-(4*E*,15*Z*)-dien-1-yn-3-one (**10**). |
| 59 | Figure S65. $^1$H NMR spectrum of (3*R*)-docos-(15*Z*)- en-1-yn-3-ol (**11**) in $CDCl_3$ |
| | Figure S66. $^{13}$C NMR spectrum of (3*R*)-docos-(15*Z*)- en-1-yn-3-ol (**11**) in $CDCl_3$ |
| 60 | Table S11. NMR data of (3*R*)-docos-(15*Z*)- en-1-yn-3-ol (**11**) in $CDCl_3$ |
| 61 | Figure S67. EIMS of (3*R*)-docos-(15*Z*)- en-1-yn-3-ol (**11**) |
| 62 | Figure S68. $^1$H NMR spectrum of (3*R*)-tetracos-(4*E*,15*Z*)-dien-1-yn-3-ol (**12**) in $CDCl_3$ |
| | Figure S69. $^{13}$C NMR spectrum of (3*R*)-tetracos-(4*E*,15*Z*)-dien-1-yn-3-ol (**12**) in $CDCl_3$ |
| 63 | Figure S70. HSQC spectrum of (3*R*)-tetracos-(4*E*,15*Z*)-dien-1-yn-3-ol (**12**) in $CDCl_3$ |
| | Figure S71. HMBC spectrum of (3*R*)-tetracos-(4*E*,15*Z*)-dien-1-yn-3-ol (**12**) in $CDCl_3$ |
| 64 | Figure S72. COSY spectrum of (3*R*)-tetracos-(4*E*,15*Z*)-dien-1-yn-3-ol (**12**) in $CDCl_3$ |
| | Figure S73. DEPT spectrum of (3*R*)-tetracos-(4*E*,15*Z*)-dien-1-yn-3-ol (**12**) in $CDCl_3$ |
| 65 | Table S12. NMR data of (3*R*)-tetracos-(4*E*,15*Z*)-dien-1-yn-3-ol (**12**) in $CDCl_3$ |
| 66 | Figure S74. EIGCMS spectrum of (3*R*)-tetracos-(4*E*,15*Z*)-dien-1-yn-3-ol (**12**) |
| 67 | Figure S75. LCMS chromatogram and mass spectrum of the periodate-permanganate oxidation products of (3*R*)-tetracos-(4*E*,15*Z*)-dien-1-yn-3-ol (**12**) |
| 68 | Figure S76. $^1$H NMR spectrum of (5*S*)-icos-(3*Z*)-en-1-yn-5-ol (**13**) in $CDCl_3$ |
| | Figure S77. $^{13}$C NMR spectrum of (5*S*)-icos-(3*Z*)-en-1-yn-5-ol (**13**) in $CDCl_3$ |
| 69 | Figure S78. HSQC spectrum of (5*S*)-icos-(3*Z*)-en-1-yn-5-ol (**13**) in $CDCl_3$ |
| | Figure S79. HMBC spectrum of (5*S*)-icos-(3*Z*)-en-1-yn-5-ol (**13**) in $CDCl_3$ |
| 70 | Figure S80. COSY spectrum of (5*S*)-icos-(3*Z*)-en-1-yn-5-ol (**13**) in $CDCl_3$ |
| | Figure S81. DEPT spectrum of (5*S*)-icos-(3*Z*)-en-1-yn-5-ol (**13**) in $CDCl_3$ |
| 71 | Table S13. NMR data of (5*S*)-icos-(3*Z*)-en-1-yn-5-ol (**13**) in $CDCl_3$ |
| | Figure S82. EIGCMS spectrum of (5*S*)-icos-(3*Z*)-en-1-yn-5-ol (**13**) |
| 72 | Figure S83. HRCIMS spectrum of (5*S*)-icos-(3*Z*)-en-1-yn-5-ol (**13**) |
| 73 | Figure S84. $^1$H NMR spectrum of (5*S*)-14-methylicos-(3*Z*)-en-1-yn-5-ol (**14**) in $CDCl_3$ |
| | Figure S85. $^{13}$C NMR spectrum of (5*S*)-14-methylicos-(3*Z*)-en-1-yn-5-ol (**14**) in $CDCl_3$ |
| 74 | Figure S86. HSQC spectrum of (5*S*)-14-methylicos-(3*Z*)-en-1-yn-5-ol (**14**) in $CDCl_3$ |
| | Figure S87. HMBC spectrum of (5*S*)-14-methylicos-(3*Z*)-en-1-yn-5-ol (**14**) in $CDCl_3$ |
| 75 | Figure S88. COSY spectrum of (5*S*)-14-methylicos-(3*Z*)-en-1-yn-5-ol (**14**) in $CDCl_3$ |

| | |
|---|---|
| | Figure S89. DEPT spectrum of (5*S*)-14-methylicos-(3*Z*)-en-1-yn-5-ol (**14**) in $CDCl_3$ |
| 76 | Table S14. NMR data of (5*S*)-14-methylicos-(3*Z*)-en-1-yn-5-ol (**14**) in $CDCl_3$ |
| 77 | Figure S90. EIGCMS spectrum and fragmentation of (5*S*)-14-methylicos-(3*Z*)-en-1-yn-5-ol (**14**) |
| 78 | Figure S91. HRCIMS spectrum of (5*S*)-14-methylicos-(3*Z*)-en-1-yn-5-ol (**14**) |
| 79 | Figure S92. $^{1}$H NMR spectrum of (5*S*)-18-methylicos-(3*Z*)-en-1-yn-5-ol (**15**) and (5*S*)-19-methylicos-(3*Z*)-en-1-yn-5-ol (**16**) in $CDCl_3$ |
| | Figure S93. $^{13}$C NMR spectrum of (5*S*)-18-methylicos-(3*Z*)-en-1-yn-5-ol (**15**) and (5*S*)-19-methylicos-(3*Z*)-en-1-yn-5-ol (**16**) in $CDCl_3$ |
| 80 | Figure S94. HSQC spectrum of (5*S*)-18-methylicos-(3*Z*)-en-1-yn-5-ol (**15**) and (5*S*)-19-methylicos-(3*Z*)-en-1-yn-5-ol (**16**) in $CDCl_3$ |
| | Figure S95. HMBC spectrum of (5*S*)-18-methylicos-(3*Z*)-en-1-yn-5-ol (**15**) and (5*S*)-19-methylicos-(3*Z*)-en-1-yn-5-ol (**16**) in $CDCl_3$ |
| 81 | Figure S96. COSY spectrum of (5*S*)-18-methylicos-(3*Z*)-en-1-yn-5-ol (**15**) and (5*S*)-19-methylicos-(3*Z*)-en-1-yn-5-ol (**16**) in $CDCl_3$ |
| | Figure S97. DEPT spectrum of (5*S*)-18-methylicos-(3*Z*)-en-1-yn-5-ol (**15**) and (5*S*)-19-methylicos-(3*Z*)-en-1-yn-5-ol (**16**) in $CDCl_3$ |
| 82 | Table S15. NMR data of (5*S*)-18-methylicos-(3*Z*)-en-1-yn-5-ol (**15**) in $CDCl_3$ |
| | Table S16. NMR data of (5*S*)-19-methylicos-(3*Z*)-en-1-yn-5-ol (**16**) in $CDCl_3$ |
| 83 | Figure S98. EIGCMS spectrum of (5*S*)-18-methylicos-(3*Z*)-en-1-yn-5-ol (**15**) |
| | Figure S99. EIGCMS spectrum of (5*S*)-19-methylicos-(3*Z*)-en-1-yn-5-ol (**16**) |
| 84 | Figure S100. HRCIMS spectrum of (5*S*)-18-methylicos-(3*Z*)-en-1-yn-5-ol (**15**) |
| 85 | Figure S100. HRCIMS spectrum of (5*S*)-19-methylicos-(3*Z*)-en-1-yn-5-ol (**16**) |
| 86 | Figure S102. $^{1}$H NMR spectrum of 14-methyldocos-(3*Z*)-en-1-yn-5,6-diol (**17**) in $CDCl_3$ |
| | Figure S103. $^{13}$C NMR spectrum of 14-methyldocos-(3*Z*)-en-1-yn-5,6-diol (**17**) in $CDCl_3$ |
| 87 | Figure S104. HSQC spectrum of 14-methyldocos-(3*Z*)-en-1-yn-5,6-diol (**17**) in $CDCl_3$ |
| | Figure S105. HMBC spectrum of 14-methyldocos-(3*Z*)-en-1-yn-5,6-diol (**17**) in $CDCl_3$ |
| 88 | Figure S106. COSY spectrum of 14-methyldocos-(3*Z*)-en-1-yn-5,6-diol (**17**) in $CDCl_3$ |
| 89 | Table S17. NMR data of 14-methyldocos-(3*Z*)-en-1-yn-5,6-diol (**17**) in $CDCl_3$ |
| 90 | Figure S107. EIMS and fragmentation pattern of 14-methyldocos-(3*Z*)-en-1-yn-5,6-diol (**17**) |
| 91 | Figure S108. $^{1}$H NMR spectrum of(3*R*)-icos-(4*E*)-en-1-yn-3-ol (**18**) in $CDCl_3$ |
| | Figure S109. $^{13}$C NMR spectrum of(3*R*)-icos-(4*E*)-en-1-yn-3-ol (**18**) in $CDCl_3$ |
| 92 | Figure S110. HSQC spectrum of(3*R*)-icos-(4*E*)-en-1-yn-3-ol (**18**) in $CDCl_3$ |
| | Figure S111. HMBC spectrum of(3*R*)-icos-(4*E*)-en-1-yn-3-ol (**18**) in $CDCl_3$ |
| 93 | Figure S112. COSY spectrum of(3*R*)-icos-(4*E*)-en-1-yn-3-ol (**18**) in $CDCl_3$ |
| | Figure S113. DEPT spectrum of(3*R*)-icos-(4*E*)-en-1-yn-3-ol (**18**) in $CDCl_3$ |
| 94 | Table S18. NMR data of (3*R*)-icos-(4*E*)-en-1-yn-3-ol (**18**) in $CDCl_3$ |
| 95 | Figure S114. EIGCMS spectrum of (3*R*)-icos-(4*E*)-en-1-yn-3-ol (**18**) |
| | Figure S115. HRCIMS spectrum of (3*R*)-icos-(4*E*)-en-1-yn-3-ol (**18**) |
| 96 | Figure S116. $^{1}$H NMR spectrum of (3*R*)-19-methylicos-(4*E*)-en-1-yn-3-ol (**19**) in $CDCl_3$ |
| | Figure S117. $^{13}$C NMR spectrum of (3*R*)-19-methylicos-(4*E*)-en-1-yn-3-ol (**19**) in $CDCl_3$ |
| 97 | Table S19. NMR data of (3*R*)-19-methylicos-(4*E*)-en-1-yn-3-ol (**19**) in $CDCl_3$ |
| | Figure S118. HRCIMS spectrum of (3*R*)-19-methylicos-(4*E*)-en-1-yn-3-ol (**19**) |
| 98 | Figure S119. $^{1}$H NMR spectrum of (3*R*)-henicos-(4*E*)-en-1-yn-3-ol (**20**) in $CDCl_3$ |
| | Table S20. NMR data of (3*R*)-henicos-(4*E*)-en-1-yn-3-ol (**20**) in $CDCl_3$ |
| 99 | Figure S120. CIGCMS spectrum of (3*R*)-henicos-(4*E*)-en-1-yn-3-ol (**20**) |
| | Figure S121. HRCIMS spectrum of (3*R*)-henicos-(4*E*)-en-1-yn-3-ol (**20**) |

| | |
|---|---|
| 100 | Figure S122. $^{1}$H NMR spectrum of (3*R*)-docos-(4*E*,15*Z*)-dien-1-yn-3-ol (**21**) in $CDCl_3$ |
| | Figure S123. $^{13}$C NMR spectrum of (3*R*)-docos-(4*E*,15*Z*)-dien-1-yn-3-ol (**21**) in $CDCl_3$ |
| 101 | Table S21. NMR data of (3*R*)-docos-(4*E*,15*Z*)-dien-1-yn-3-ol (**21**) in $CDCl_3$ |
| | Figure S124. EIGCMS spectrum of (3*R*)-docos-(4*E*,15*Z*)-dien-1-yn-3-ol (**21**) |
| 102 | Figure S125. $^{1}$H NMR spectrum of (3*R*)-21-methyldocos-(4*E*,15*Z*)-dien-1-yn-3-ol (**22**) in $CDCl_3$ |
| | Figure S126. $^{13}$C NMR spectrum of (3*R*)-21-methyldocos-(4*E*,15*Z*)-dien-1-yn-3-ol (**22**) in $CDCl_3$ |
| 103 | Table S22. NMR data of (3*R*)-21-methyldocos-(4*E*,15*Z*)-dien-1-yn-3-ol (**22**) in $CDCl_3$ |
| | Figure S127. EIMS spectrum of (3*R*)-21-methyldocos-(4*E*,15*Z*)-dien-1-yn-3-ol (**22**) |
| 104 | Figure S128. $^{1}$H NMR spectrum of (3*R*)-14-methyldocos-1-yn-3-ol (**23**) in $CDCl_3$ |
| | Figure S129. $^{13}$C NMR spectrum of (3*R*)-14-methyldocos-1-yn-3-ol (**23**) in $CDCl_3$ |
| 105 | Table S23. NMR data of (3*R*)-14-methyldocos-1-yn-3-ol (**23**) in $CDCl_3$ |
| 106 | Figure S130. EIMS of (3*R*)-14-methyldocos-1-yn-3-ol (**23**) |
| 107 | Figure S131. $^{1}$H NMR spectrum of (4*E*,6*E*)-docosa-4,6-dien-1-yn-3-ol (***rac*-27**) in $CDCl_3$ |
| | Figure S132. $^{13}$C NMR spectrum of (4*E*,6*E*)-docosa-4,6-dien-1-yn-3-ol (***rac*-27**) in $CDCl_3$ |
| 108 | Figure S133. HREIMS of (4*E*,6*E*)-docosa-4,6-dien-1-yn-3-ol (***rac*-27**) |
| 109 | Figure S134. $^{1}$H NMR spectrum of (*S*)-((*R*)-icos-(4*E*)-en-1-yn-3-yl-3,3,3-trifluoro-2-methoxy-2-phenylpropanoate (**(*S,R*)-29**) in $CDCl_3$ |
| | Figure S135. $^{1}$H NMR spectrum of (*S*)-((*S*)-icos-(4*E*)-en-1-yn-3-yl-3,3,3-trifluoro-2-methoxy-2-phenylpropanoate (**(*S,S*)-29**) in $CDCl_3$ |
| 110 | Figure S136. $^{1}$H NMR spectrum of dodec-1-yn-3-ol (***rac*-31**) in $CDCl_3$ |
| | Figure S137. $^{13}$C NMR spectrum of dodec-1-yn-3-ol (***rac*-31**) in $CDCl_3$ |
| 111 | Figure S138. HRCIMS of dodec-1-yn-3-ol (***rac*-31**) |
| 112 | Figure S139. $^{1}$H NMR spectrum of octadec-1-yn-3-ol (***rac*-32**) in $CDCl_3$ |
| | Figure S140. $^{13}$C NMR spectrum of octadec-1-yn-3-ol (***rac*-32**) in $CDCl_3$ |
| 113 | Figure S141. CIGCMS of octadec-1-yn-3-ol (***rac*-32**) |
| | Figure S142. HRCIMS of octadec-1-yn-3-ol (***rac*-32**) |
| 114 | Figure S143. $^{1}$H NMR spectrum of octadec-1-yn-3-ol (***R*-32**) in $CDCl_3$ |
| | Figure S144. $^{1}$H NMR spectrum of octadec-1-yn-3-ol (***S*-32**) in $CDCl_3$ |
| 115 | Figure S145. $^{1}$H NMR spectrum of icos-1-yn-3-ol (***rac*-33**) in $CDCl_3$ |
| | Figure S146. $^{13}$C NMR spectrum of icos-1-yn-3-ol (***rac*-33**) in $CDCl_3$ |
| 116 | Figure S147. CIGCMS of icos-1-yn-3-ol (***rac*-33**) |
| | Figure S148. HRCIMS of icos-1-yn-3-ol (***rac*-33**) |
| 117 | Figure S149. $^{1}$H NMR spectrum of (*R*)-((*S*)-octadec-1-yn-3-yl)-3,3,3-trifluoro-2-methoxy-2-phenylpropanoate (**(*R,S*)-34**) in $CDCl_3$ |
| | Figure S150. $^{1}$H NMR spectrum of (*R*)-((*S*)-octadec-1-yn-3-yl)-3,3,3-trifluoro-2-methoxy-2-phenylpropanoate (**(*R,S*)-34**) in $CDCl_3$ |
| 118 | Figure S151. $^{1}$H NMR spectrum of (*R*)-((*R*)-octadec-1-yn-3-yl)-3,3,3-trifluoro-2-methoxy-2-phenylpropanoate (**(*R,R*)-34**) in $CDCl_3$ |
| | Figure S152. $^{13}$C NMR spectrum of (*R*)-((*R*)-octadec-1-yn-3-yl)-3,3,3-trifluoro-2-methoxy-2-phenylpropanoate (**(*R,R*)-34**) in $CDCl_3$ |
| 119 | Figure S153. $^{1}$H NMR spectrum of octadec-1-yn-3-yl 4-methylbenzenesulfonate (***rac*-35**) in $CDCl_3$ |
| | Figure S154. $^{13}$C NMR spectrum of octadec-1-yn-3-yl 4-methylbenzenesulfonate (***rac*-35**) in $CDCl_3$ |
| 120 | Figure S155. ESIMS of octadec-1-yn-3-yl 4-methylbenzenesulfonate (***rac*-35**) |
| | Figure S156. HRESIMS of octadec-1-yn-3-yl 4-methylbenzenesulfonate (***rac*-35**) |
| 121 | Figure S157. $^{1}$H NMR spectrum of 3-chlorooctadec-1-yne (***rac*-36**) in $CDCl_3$ |

| | |
|---|---|
| | Figure S158. $^{13}$C NMR spectrum of 3-chlorooctadec-1-yne (***rac*-36**) in CDCl$_3$ |
| 122 | Figure S159. SMBEIMS of 3-chlorooctadec-1-yne (***rac*-36**) |
| | Figure S160. HRESIMS of 3-chlorooctadec-1-yne (***rac*-36**) |
| 123 | Figure S161. $^{1}$H NMR spectrum of octadec-1-yn-3-amine (***rac*-37**) in CDCl$_3$ |
| | Figure S162. $^{13}$C NMR spectrum of octadec-1-yn-3-amine (***rac*-37**) in CDCl$_3$ |
| 124 | Figure S163. ESIMS of octadec-1-yn-3-amine (***rac*-37**) |
| | Figure S164. HRESIMS of octadec-1-yn-3-amine (***rac*-37**) |
| 125 | Figure S165. $^{1}$H NMR spectrum of 3-methoxyoctadec-1-yne (***rac*-38**) in CDCl$_3$ |
| | Figure S166. $^{13}$C NMR spectrum of 3-methoxyoctadec-1-yne (***rac*-38**) in CDCl$_3$ |
| 126 | Figure S167. EIGCMS of 3-methoxyoctadec-1-yne (***rac*-38**) |
| 127 | Figure S168. $^{1}$H NMR spectrum of *S*-octadec-1-yn-3-yl ethanethioate (***rac*-39**) in CDCl$_3$ |
| | Figure S169. $^{13}$C NMR spectrum of *S*-octadec-1-yn-3-yl ethanethioate (***rac*-39**) in CDCl$_3$ |
| 128 | Figure S170. ESIMS of *S*-octadec-1-yn-3-yl ethanethioate (***rac*-39**) |
| 129 | Figure S171. $^{1}$H NMR spectrum of octadec-1-yn-3-thiol (***rac*-40**) in CDCl$_3$ |
| | Figure S172. $^{13}$C NMR spectrum of octadec-1-yn-3-thiol (***rac*-40**) in CDCl$_3$ |
| 130 | Figure S173. $^{1}$H NMR spectrum of 3-methylnonadec-1-yn-3-ol (***rac*-41**) in CDCl$_3$ |
| | Figure S174. $^{13}$C NMR spectrum of 3-methylnonadec-1-yn-3-ol (***rac*-41**) in CDCl$_3$ |
| 131 | Figure S175. HRCIMS 3-methylnonadec-1-yn-3-ol (***rac*-41**) |
| 132 | Figure S176. $^{1}$H NMR spectrum of henicos-2-yn-4-ol (***rac*-42**) in CDCl$_3$ |
| | Figure S177. $^{13}$C NMR spectrum of henicos-2-yn-4-ol (***rac*-42**) in CDCl$_3$ |
| 133 | Figure S178. HRCIMS henicos-2-yn-4-ol (***rac*-42**) |
| 134 | Figure S179. $^{1}$H NMR spectrum of 1-(3-tetradecylphenyl)prop-2-yn-1-ol (***rac*-45**) in CDCl$_3$ |
| | Figure S180. $^{13}$C NMR spectrum of 1-(3-tetradecylphenyl)prop-2-yn-1-ol (***rac*-45**) in CDCl$_3$ |
| 135 | Figure S181. HREIMS of 1-(3-tetradecylphenyl)prop-2-yn-1-ol (***rac*-45**) |
| 136 | Figure S182. $^{1}$H NMR spectrum of 1-(2-tetradecylphenyl)prop-2-yn-1-ol (***rac*-48**) in CDCl$_3$ |
| | Figure S183. $^{13}$C NMR spectrum of 1-(2-tetradecylphenyl)prop-2-yn-1-ol (***rac*-48**) in CDCl$_3$ |
| 137 | Figure S184. HREIMS of 1-(2-tetradecylphenyl)prop-2-yn-1-ol (***rac*-48**) |
| 138 | Figure S185. $^{1}$H NMR spectrum of 1-phenylprop-2-yn-1-ol (***rac*-49**) in CDCl$_3$ |
| | Figure S186. $^{13}$C NMR spectrum of 1-phenylprop-2-yn-1-ol (rac-49) in CDCl$_3$ |
| 139 | Figure S187. HREIMS of 1-phenylprop-2-yn-1-ol (***rac*-49**) |
| 140 | Figure S188. Dose response curves of the compounds described in Table 1 obtained from screening of NSCLC U-1810 cells or diploid fibroblast WI-38 cells are presented. The $IC_{50}$ values were deduced from the cell viability curves. |

Figure S1. $^1$H NMR spectrum of (3*R*)-18-methylnonadec-(4*E*)-en-1-yn-3-ol (**1**) in $CDCl_3$.

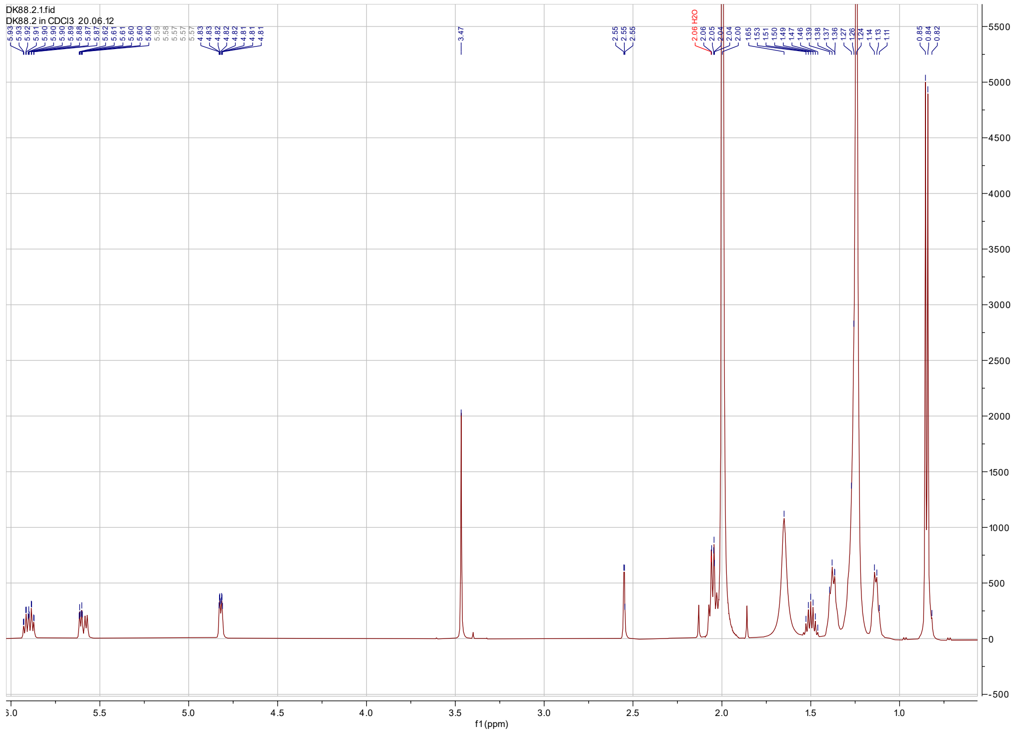

Figure S2. $^{13}$C NMR spectrum of (3*R*)-18-methylnonadec-(4*E*)-en-1-yn-3-ol (**1**) in $CDCl_3$.

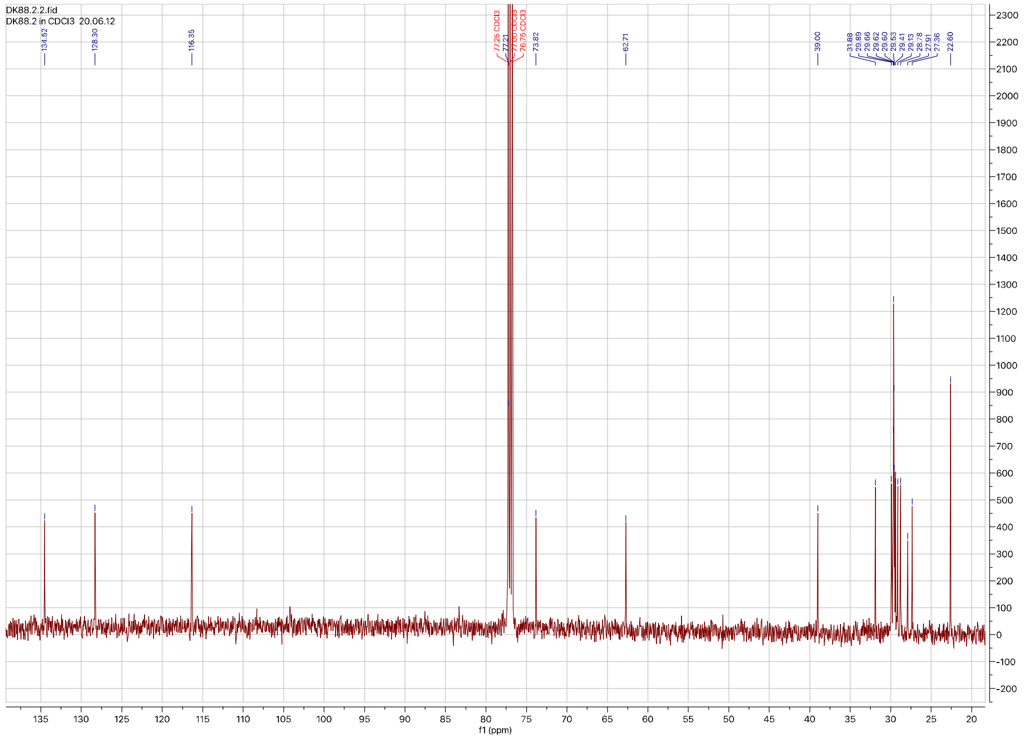

Figure S3. HSQC spectrum of (3*R*)-18-methylnonadec-(4*E*)-en-1-yn-3-ol (**1**) in $CDCl_3$.

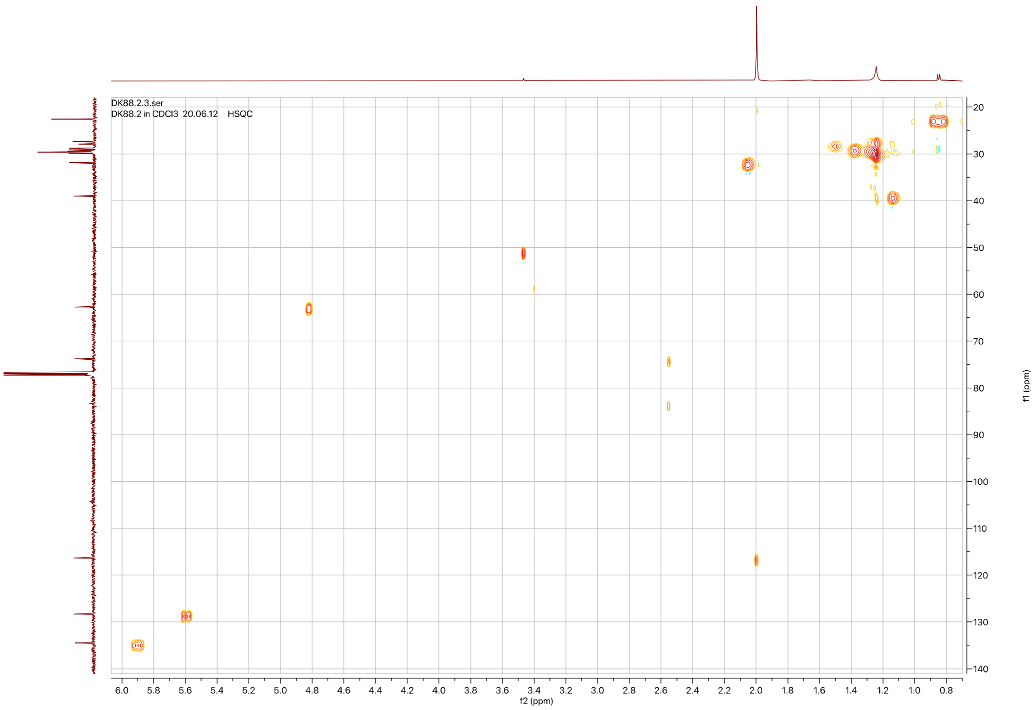


Figure S4. HMBC spectrum of (3*R*)-18-methylnonadec-(4*E*)-en-1-yn-3-ol (**1**) in $CDCl_3$.

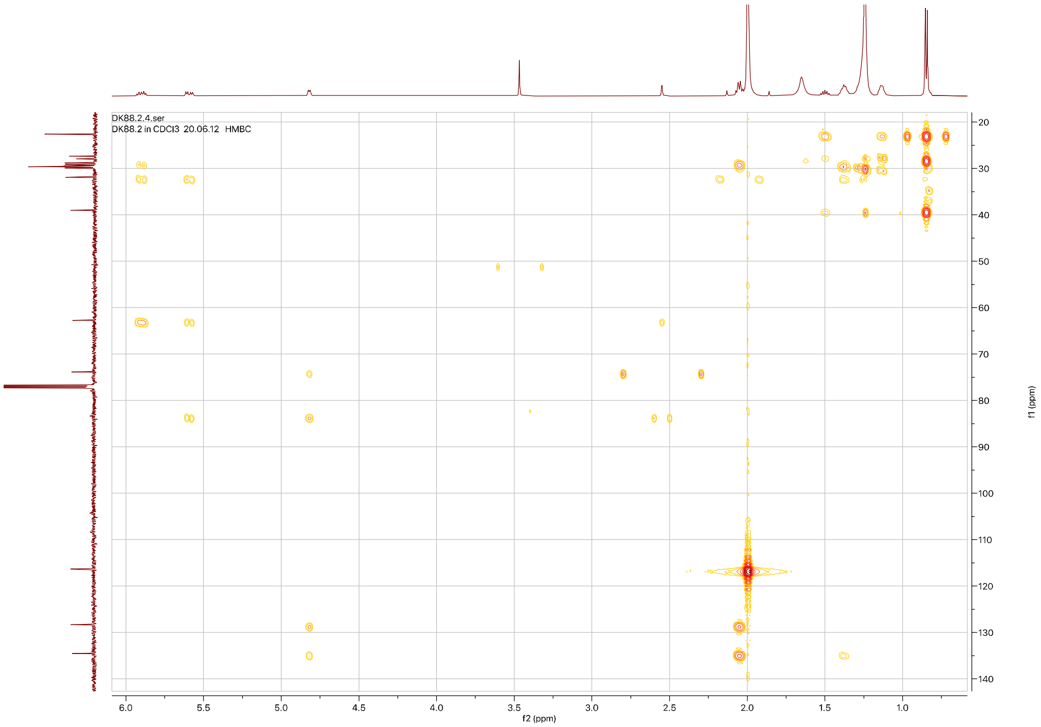

Figure S5. COSY spectrum of (3*R*)-18-methylnonadec-(4*E*)-en-1-yn-3-ol (**1**) in $CDCl_3$.

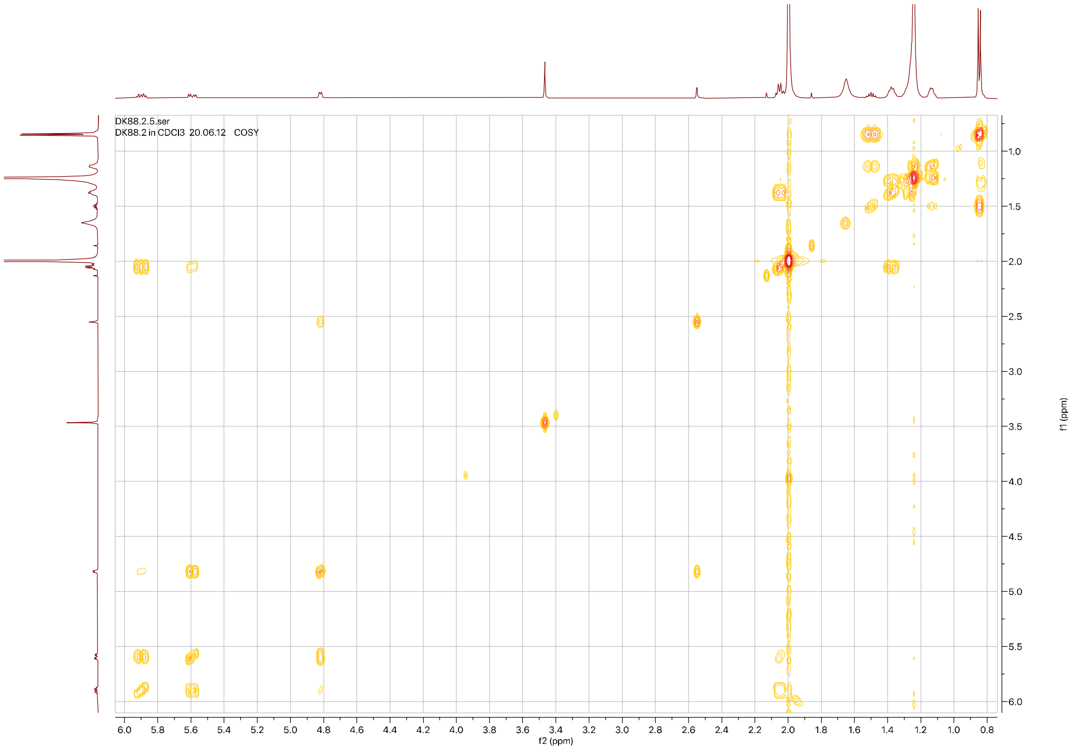


Figure S6. DEPT spectrum of (3*R*)-18-methylnonadec-(4*E*)-en-1-yn-3-ol (**1**) in $CDCl_3$.

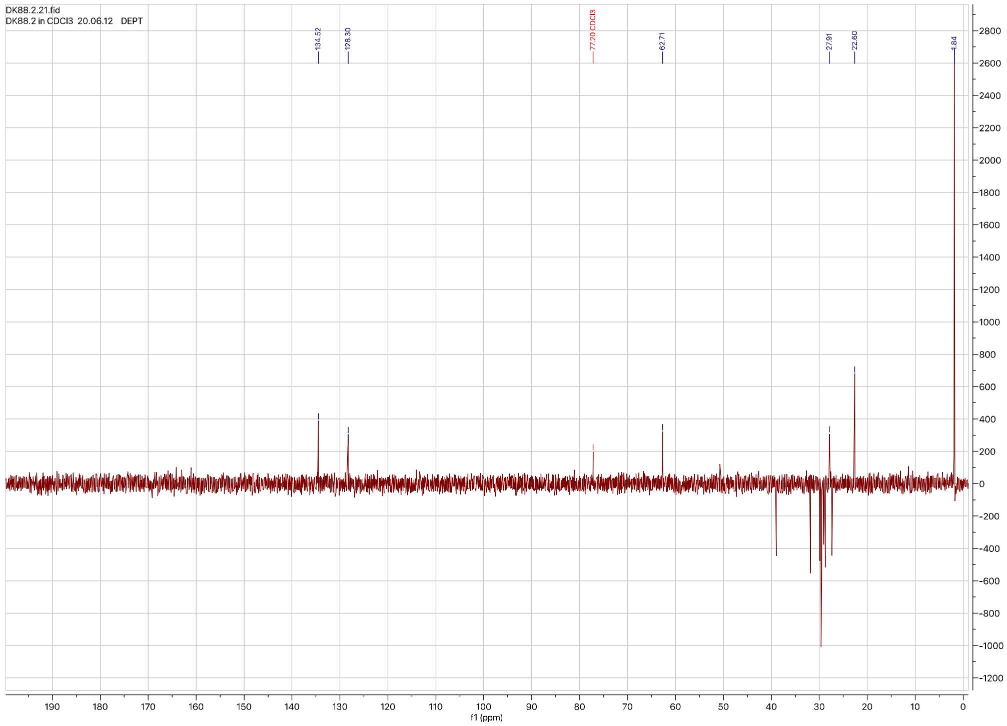

Table S1. NMR data of (3*R*)-18-methylnonadec-(4*E*)-en-1-yn-3-ol (**1**) in $CDCl_3$.[a]

| Position | $\delta_C$, mult.[b] | $\delta_H$, mult. *J* (Hz) | LR H-C Correlations[c] |
|---|---|---|---|
| 1 | 73.8[d] CH | 2.55 d (2.1) | 3 |
| 2 | 83.3[e] qC | - | 1, 3, 4 |
| 3 | 62.7 CH | 4.82 d (6.0) | 1, 4, 5 |
| 4 | 128.3 CH | 5.59 dd (15.2, 6.0) | 3, 6 |
| 5 | 134.5 CH | 5.90 dt (15.2, 7.0) | 3, 6, 7 |
| 6 | 31.9 $CH_2$ | 2.05 q (7.0) | 4, 5, 7, 8 |
| 7 | 28.8 $CH_2$ | 1.38 m | 5, 6, 8 |
| 8-15 | ~29.6[f] 8 × $CH_2$ | 1.22 – 1.26 brm | |
| 16 | 27.4 $CH_2$ | 1.24 m | 15, 17 |
| 17 | 39.0 $CH_2$ | 1.13 m | 16, 18, 19, 20 |
| 18 | 27.9 CH | 1.50 qqt (6.5, 6.5, 6.5) | 17, 19, 20 |
| 19 | 22.6 $CH_3$ | 0.85 d (6.5) | 17, 18, 20 |
| 20 | 22.6 $CH_3$ | 0.85 d (6.5) | 17, 18, 19 |

[a]500.13 MHz for $^1$H and 125.76 MHz for $^{13}$C; [b]Multiplicity and assignment from HSQC experiment; [c]Determined from HMBC experiment; [d] $^1J$ = 250.0 Hz; [e] $^2J$ = 49.0 Hz; [f]Exact $^{13}$C chemical shifts 29.13, 29.41, 29.62 (× 4), 29.66, 29.89 ppm.

Figure S7. GCMS spectrum of (3*R*)-18-methylnonadec-(4*E*)-en-1-yn-3-ol (**1**).

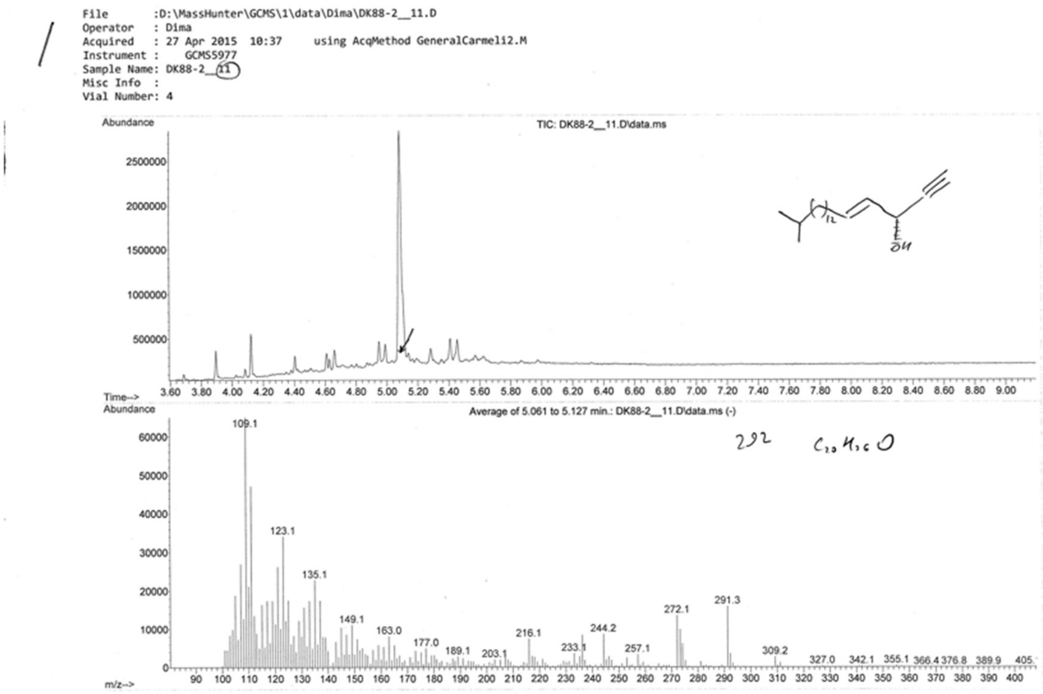


Figure S8. HRMS spectrum of (3*R*)-18-methylnonadec-(4*E*)-en-1-yn-3-ol (**1**).

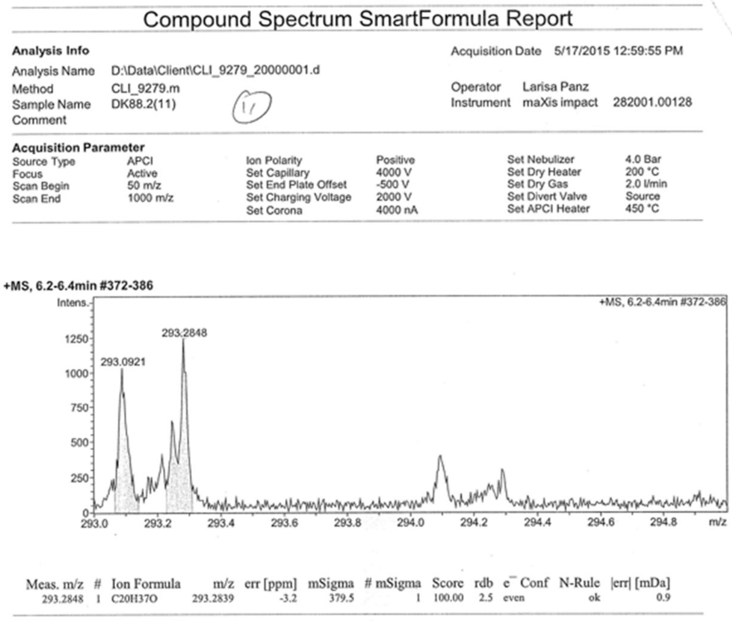

Figure S9. $^{1}$H NMR spectrum of (3*R*)-14-methylnonadec-(4*E*)-en-1-yn-3-ol (**2**) in $CDCl_3$

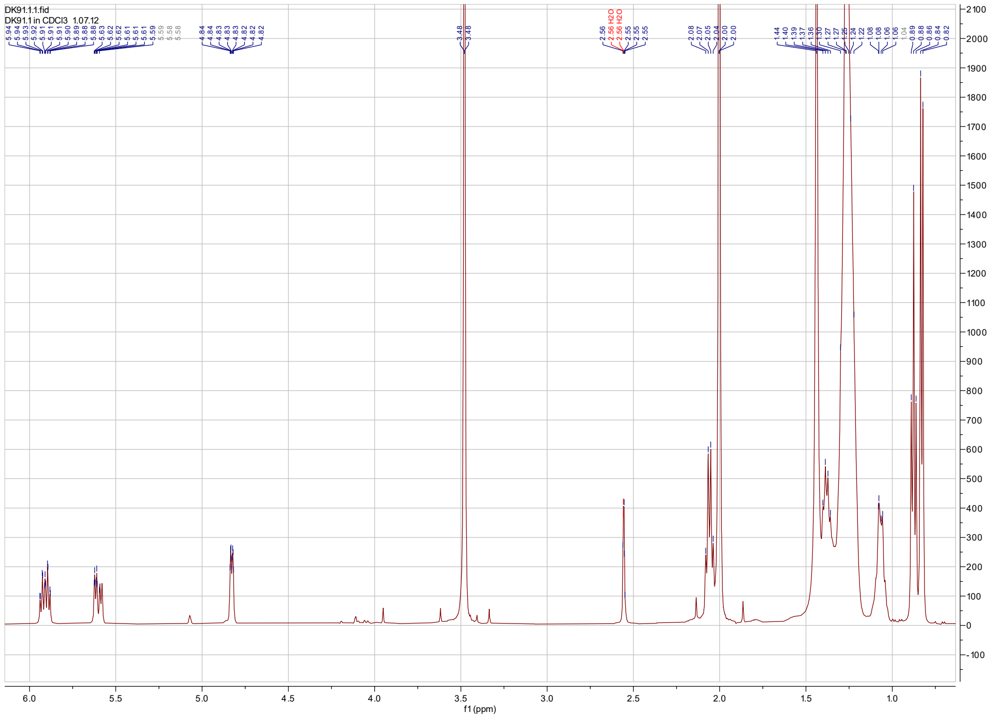


Figure S10. $^{13}$C NMR spectrum of (3*R*)-14-methylnonadec-(4*E*)-en-1-yn-3-ol (**2**) in $CDCl_3$

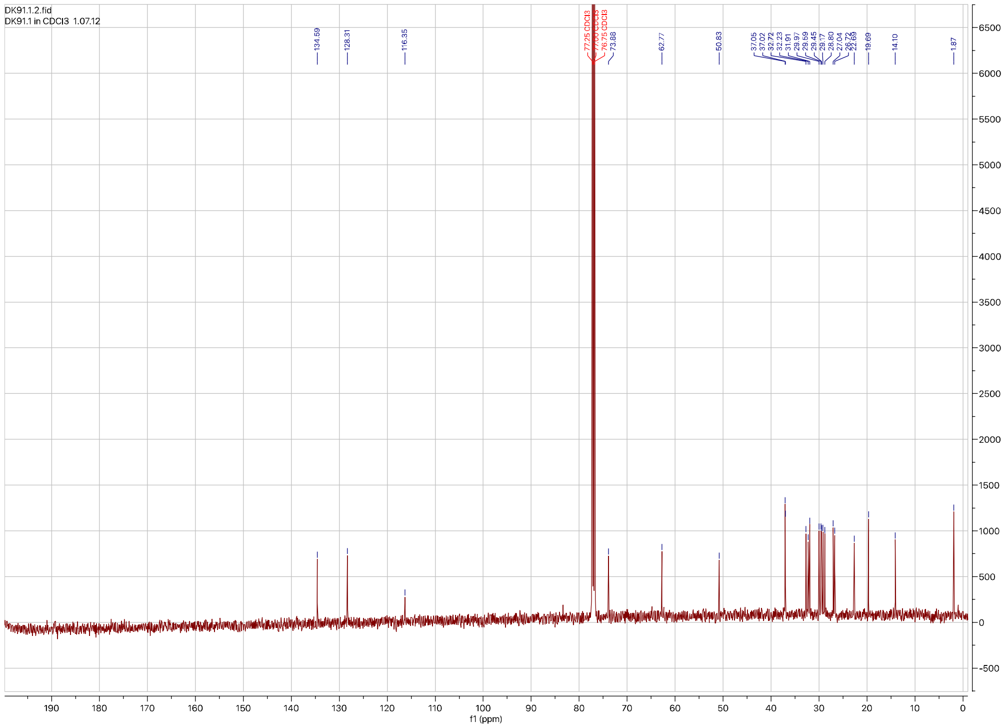

Figure S11. HSQC spectrum of (3*R*)-14-methylnonadec-(4*E*)-en-1-yn-3-ol (**2**) in $CDCl_3$

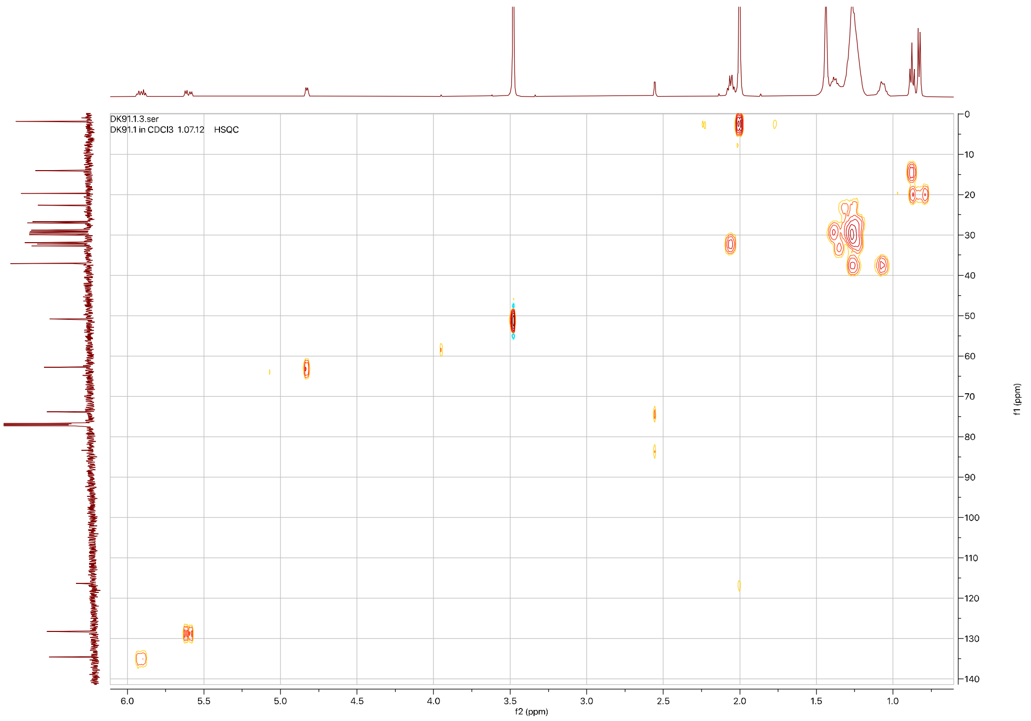


Figure S12. HMBC spectrum of (3*R*)-14-methylnonadec-(4*E*)-en-1-yn-3-ol (**2**) in $CDCl_3$

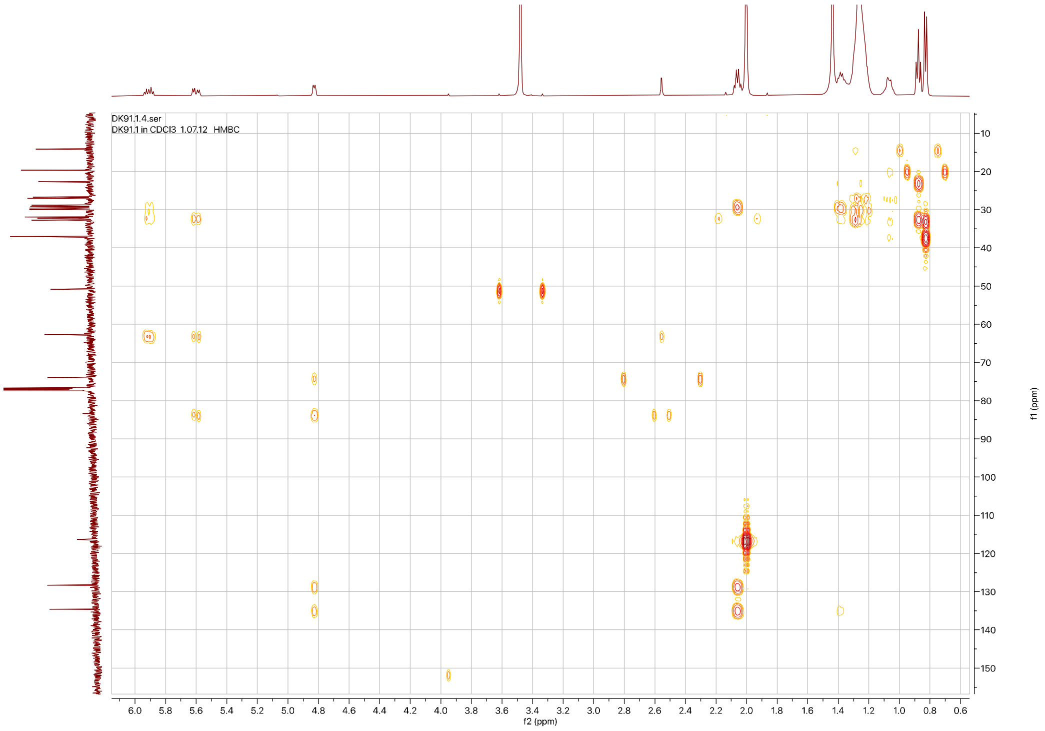

Figure S13. COSY spectrum of (3*R*)-14-methylnonadec-(4*E*)-en-1-yn-3-ol (**2**) in $CDCl_3$

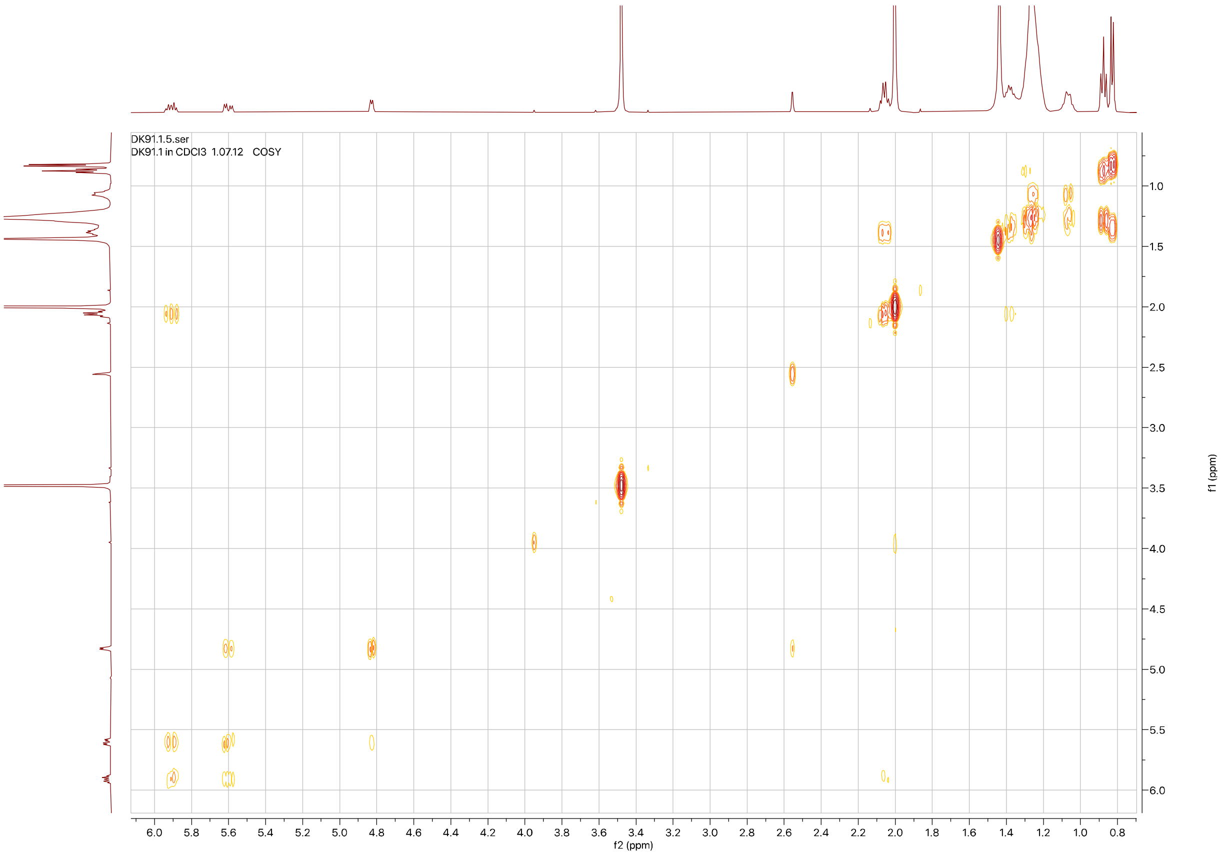

Table S2. NMR data of (3*R*)-14-methylnonadec-(4*E*)-en-1-yn-3-ol (**2**) in $CDCl_3$.[a]

| Position | $\delta_C$, mult.[b] | $\delta_H$, mult. *J* (Hz) | LR H-C Correlations[c] |
|---|---|---|---|
| 1 | 73.9[d] CH | 2.55 d (2.0) | 3 |
| 2 | 83.4[e] qC | - | 1, 3, 4 |
| 3 | 62.8 CH | 4.82 d (5.7) | 1, 4, 5 |
| 4 | 128.3 CH | 5.60 dd (15.0, 5.7) | 3, 6 |
| 5 | 134.5 CH | 5.90 dt (15.0, 7.0) | 3, 6, 7 |
| 6 | 31.9 $CH_2$ | 2.06 q (7.0) | 4, 5, 7, 8 |
| 7 | 28.8 $CH_2$ | 1.36 m | 5, 6, 8 |
| 8-11 | ~29.6[f] 4 × $CH_2$ | 1.21 – 1.31 brm | |
| 12 | 27.0 $CH_2$ | 1.23 m | 11, 13a, 13b |
| 13a | 37.0 $CH_2$ | 1.25 m | 12, 14, 15a, 15b, 21 |
| b | | 1.06 m | |
| 14 | 32.7 CH | 1.37 m | 13a, 13b, 15a, 15a, 21 |
| 15a | 37.0 $CH_2$ | 1.25 m | 13a, 13b, 14, 16, 21 |
| b | | 1.06 m | |
| 16 | 26.7 $CH_2$ | 1.24 m | 15a, 15b, 17 |
| 17 | 32.2 $CH_2$ | 1.24 m | 16, 18a, 18b, 19 |
| 18a | 22.7 $CH_2$ | 1.30 m | 17, 19 |
| b | | 1.24 m | |
| 19 | 14.1 $CH_3$ | 0.87 t (7.0) | 18a, 18b |
| 20 | 19.7 $CH_3$ | 0.83 d (7.0) | 13b, 15b |

[a]500.13 MHz for $^{1}$H and 125.76 MHz for $^{13}$C; [b]Multiplicity and assignment from HSQC experiment; [c]Determined from HMBC experiment; [d] $^{1}J$ = 250.0 Hz; [e] $^{2}J$ = 48.8 Hz; [f]Exact $^{13}$C chemical shifts 29.17, 29.45, 29.59, 29.97 ppm.

Figure S14. GCMS spectrum of (3*R*)-14-methylnonadec-(4*E*)-en-1-yn-3-ol (**2**)

File :C:\MSDCHEM\1\DATA\SMB DATA 7_11\Snapshot\AVIV1261.D
Operator :
Acquired : 12 Aug 2012 14:37 using AcqMethod SMB GC-MS Organics.M
Instrument : GC-MSD
Sample Name: Dima K 91.1
Misc Info : Dima K 91.1
Vial Number: 1

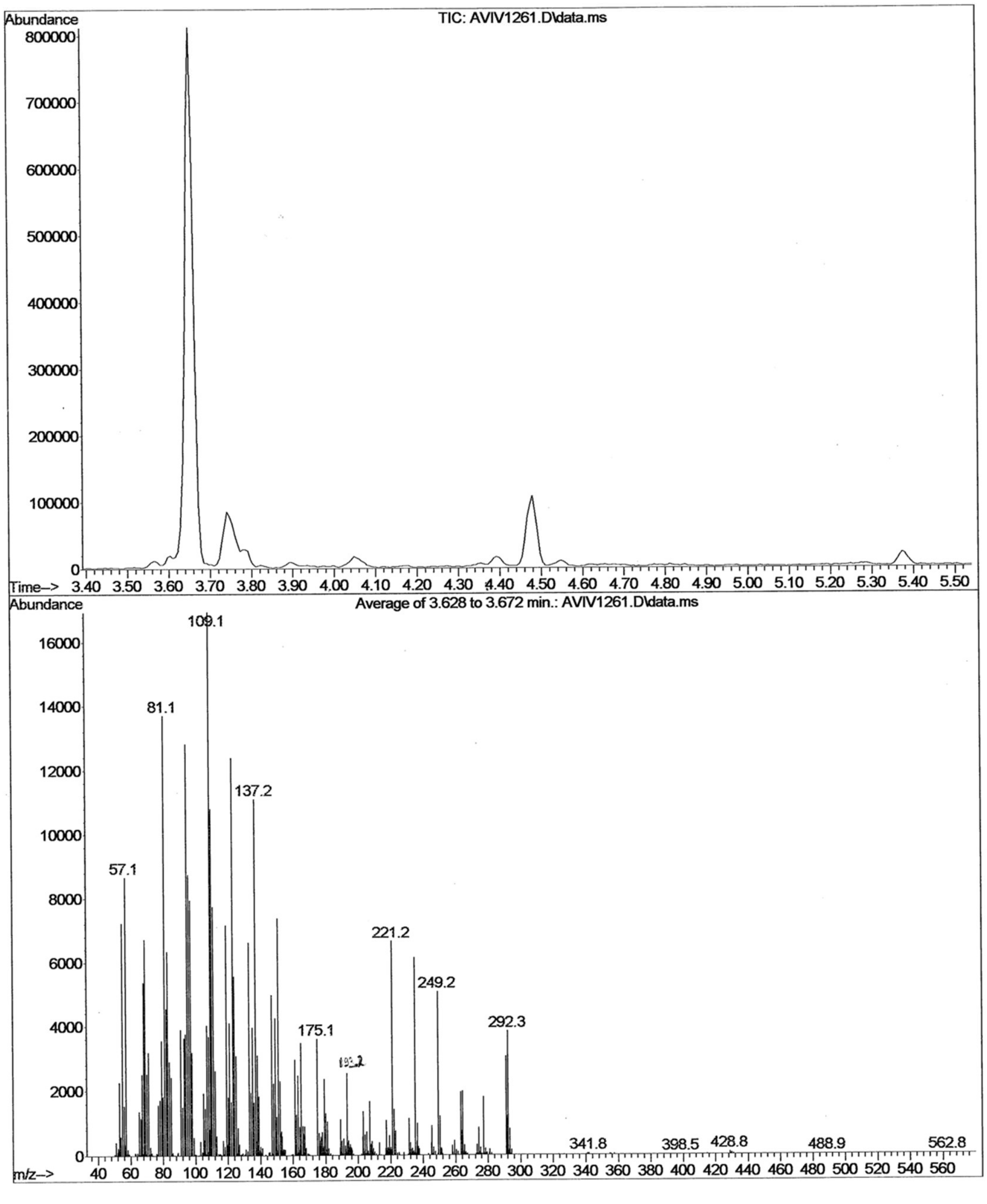

Figure S15. HRMS spectrum of (3*R*)-14-methylnonadec-(4*E*)-en-1-yn-3-ol (**2**)

## Compound Spectrum SmartFormula Report

**Analysis Info**

| | | | |
|---|---|---|---|
| Analysis Name | D:\Data\Client\CLI_9279_25000001.d | Acquisition Date | 5/17/2015 2:25:32 PM |
| Method | GC APCI_pos_cli_8051.m | Operator | Larisa Panz |
| Sample Name | DK91.1-12 | Instrument | maXis impact 282001.00128 |
| Comment | | | |

**Acquisition Parameter**

| | | | | | |
|---|---|---|---|---|---|
| Source Type | APCI | Ion Polarity | Positive | Set Nebulizer | 4.0 Bar |
| Focus | Active | Set Capillary | 4000 V | Set Dry Heater | 200 °C |
| Scan Begin | 50 m/z | Set End Plate Offset | -500 V | Set Dry Gas | 2.0 l/min |
| Scan End | 1000 m/z | Set Charging Voltage | 2000 V | Set Divert Valve | Source |
| | | Set Corona | 4000 nA | Set APCI Heater | 450 °C |

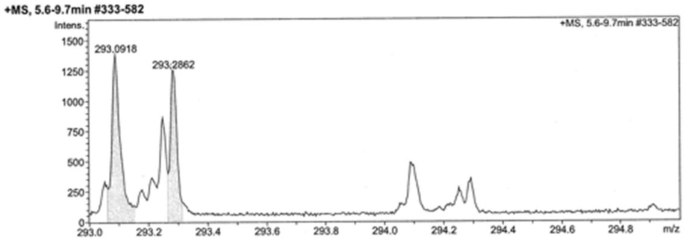


| Meas. m/z | # | Ion Formula | m/z | err [ppm] | mSigma | # mSigma | Score | rdb | e⁻ Conf | N-Rule | \|err\| [mDa] |
|---|---|---|---|---|---|---|---|---|---|---|---|
| 293.2862 | 1 | C20H37O | 293.2839 | -8.0 | 577.2 | 1 | 100.00 | 2.5 | even | ok | 2.4 |

CLI_9279_25000001.d
Bruker Compass DataAnalysis 4.2 printed: 5/17/2015 2:38:42 PM by: Larisa Panz Page 1 of 1

Figure S16. $^{1}$H NMR spectrum of 14-methylnonadec-(4*E*)-en-1-yn-3-one (**3**) in $CDCl_3$

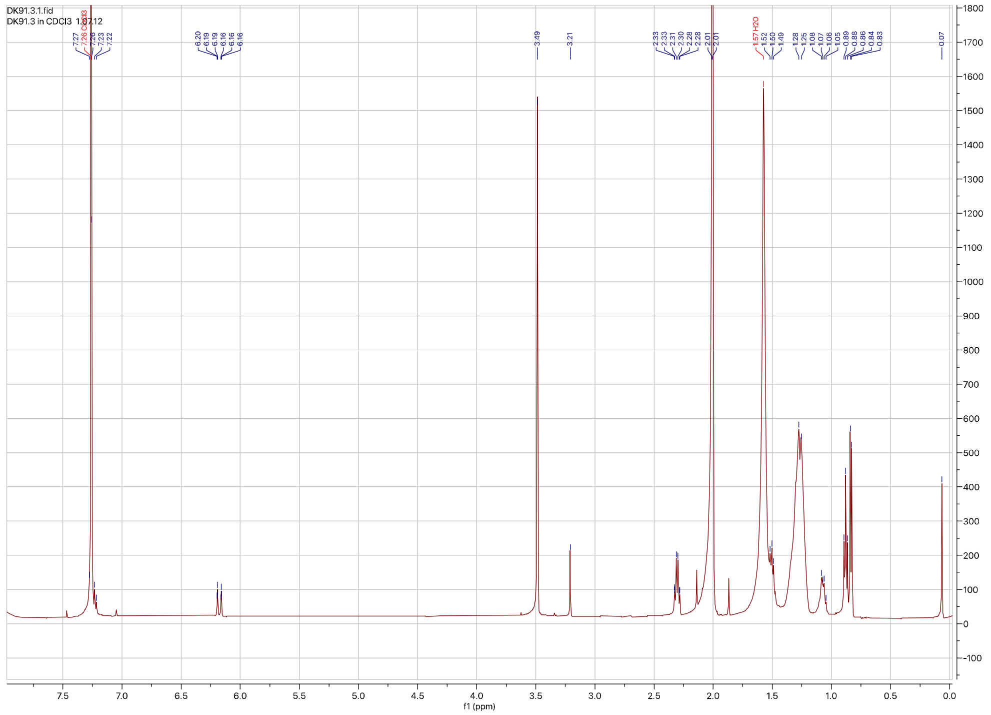


Figure S17. $^{13}$C NMR spectrum of 14-methylnonadec-(4*E*)-en-1-yn-3-one (**3**) in $CDCl_3$

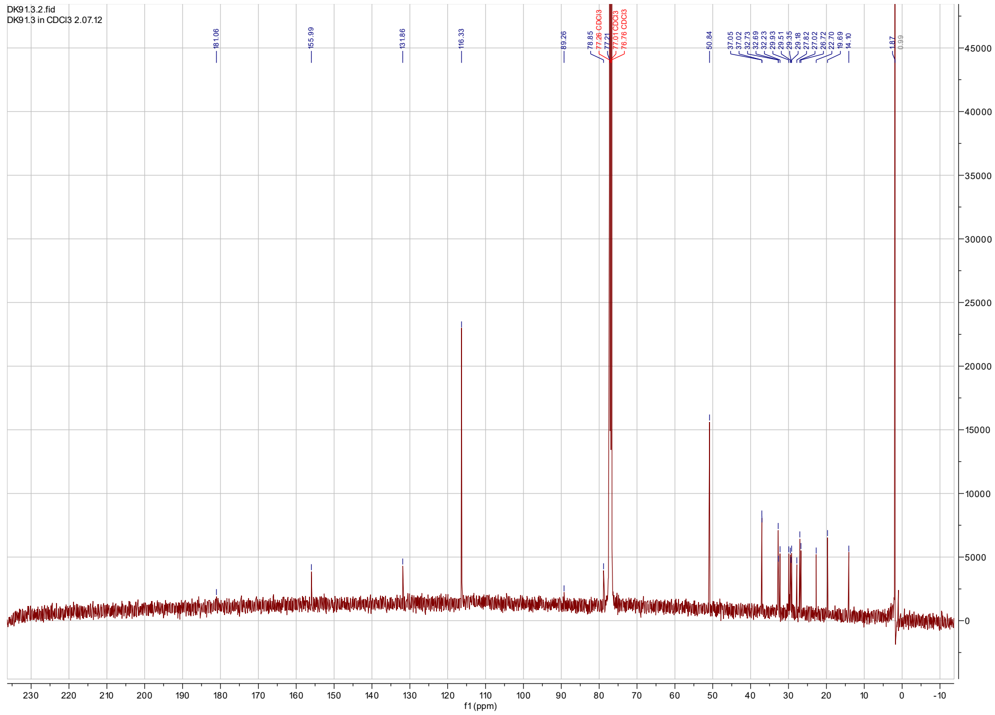

Table S3. NMR data of 14-methylnonadec-(4*E*)-en-1-yn-3-one (**3**) in $CDCl_3$.[a]

| Position | $\delta_C$, mult.[b] | $\delta_H$, mult. *J* (Hz) | LR H-C Correlations[c] |
|---|---|---|---|
| 1 | 78.8[d] CH | 3.20 s | - |
| 2 | 79.8[e] qC | - | 1, 4 |
| 3 | 177.9 qC | - | 1, 4, 5 |
| 4 | 131.9 CH | 6.17 d (16.0) | 6 |
| 5 | 156.0 CH | 7.23 dt (16.0, 7.0) | 6, 7 |
| 6 | 32.7 $CH_2$ | 2.30 q (7.0) | 4, 5, 7, 8 |
| 7 | 27.8 $CH_2$ | 1.50 tt (7.0, 7.0) | 5, 6, 8 |
| 8 | 29.2 $CH_2$ | 1.30 m | |
| 9-11 | ~29.6[f] 3 × $CH_2$ | 1.23 – 1.31 brm | |
| 12 | 27.0 $CH_2$ | 1.23 m | 11, 13a, 13b |
| 13a | 37.0 $CH_2$ | 1.26 m | 12, 14, 15a, 15b, 21 |
| b | | 1.07 m | |
| 14 | 32.7 CH | 1.37 m | 13a, 13b, 15a, 15a, 21 |
| 15a | 37.0 $CH_2$ | 1.26 m | 13a, 13b, 14, 16, 21 |
| b | | 1.07 m | |
| 16 | 26.7 $CH_2$ | 1.25 m | 15a, 15b, 17 |
| 17 | 32.2 $CH_2$ | 1.25 m | 16, 18a, 18b, 19 |
| 18a | 22.7 $CH_2$ | 1.31 m | 17, 19 |
| b | | 1.25 m | |
| 19 | 14.1 $CH_3$ | 0.88 t (7.0) | 18a, 18b |
| 20 | 19.7 $CH_3$ | 0.83 d (7.0) | 13b, 15b |

[a]500.13 MHz for $^1$H and 125.76 MHz for $^{13}$C; [b]Multiplicity and assignment from HSQC experiment; [c]Determined from HMBC experiment; [d] $^1J$ = 252.0 Hz [e] $^2J$ = 48.0 Hz; [f]Exact $^{13}$C chemical shifts 29.34, 29.51, 29.92 ppm.

Figure S18. EIMS and fragmentation pattern of 14-methylnonadec-(4*E*)-en-1-yn-3-one (**3**).

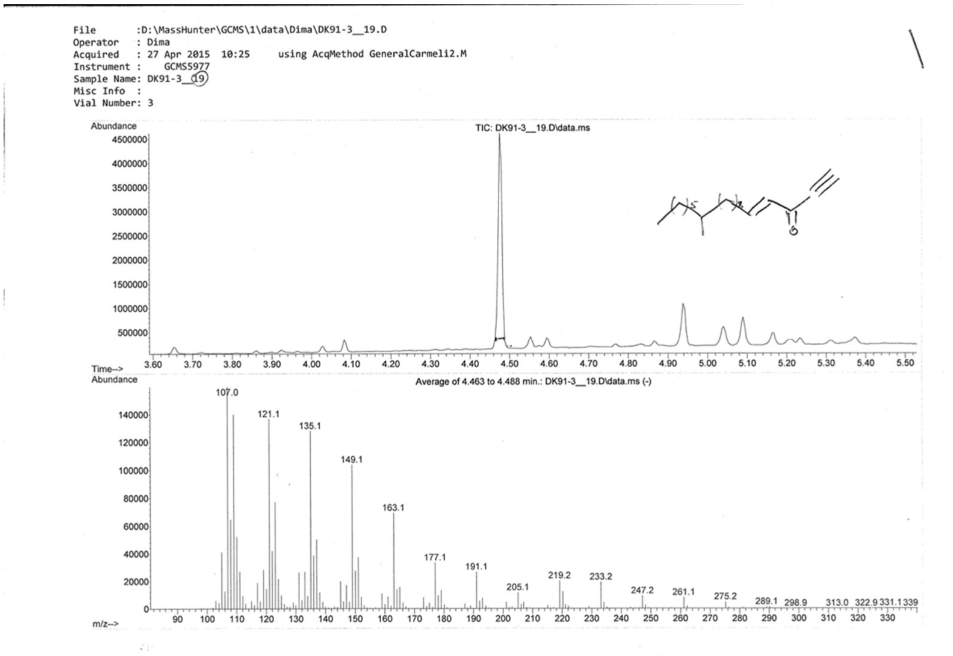
File :D:\MassHunter\GCMS\1\data\Dima\DK91-3__19.D
Operator : Dima
Acquired : 27 Apr 2015 10:25 using AcqMethod GeneralCarmeli2.M
Instrument : GCMS5977
Sample Name: DK91-3__19
Misc Info :
Vial Number: 3
Abundance
TIC: DK91-3__19.D\data.ms
Time-->
Abundance
Average of 4.463 to 4.488 min.: DK91-3__19.D\data.ms (-)
107.0
121.1
135.1
149.1
163.1
177.1
191.1
205.1
219.2
233.2
247.2
261.1
275.2
289.1
298.9
313.0
322.9
331.1
339
m/z-->

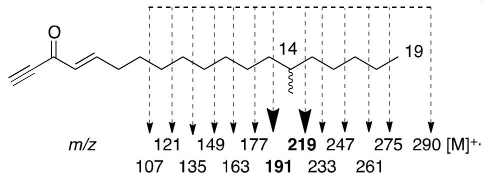
O
14
19
m/z
121 149 177 219 247 275 290 [M]+·
107 135 163 191 233 261

Figure S19. HRCIMS and fragmentation pattern of 14-methylnonadec-(4*E*)-en-1-yn-3-one (**3**)

## Compound Spectrum SmartFormula Report

**Analysis Info**

| | | | |
|---|---|---|---|
| Analysis Name | D:\Data\Client\CLI_9279_2.d | Acquisition Date | 5/17/2015 8:57:04 AM |
| Method | CLI_9279.m | Operator | Larisa Panz |
| Sample Name | DK-91-3(19) | Instrument | maXis impact 282001.00128 |
| Comment | | | |

**Acquisition Parameter**

| | | | | | |
|---|---|---|---|---|---|
| Source Type | APCI | Ion Polarity | Positive | Set Nebulizer | 4.0 Bar |
| Focus | Active | Set Capillary | 4000 V | Set Dry Heater | 200 °C |
| Scan Begin | 50 m/z | Set End Plate Offset | -500 V | Set Dry Gas | 2.0 l/min |
| Scan End | 1000 m/z | Set Charging Voltage | 2000 V | Set Divert Valve | Source |
| | | Set Corona | 4000 nA | Set APCI Heater | 450 °C |

**+MS, 6.1-6.4min #363-385**

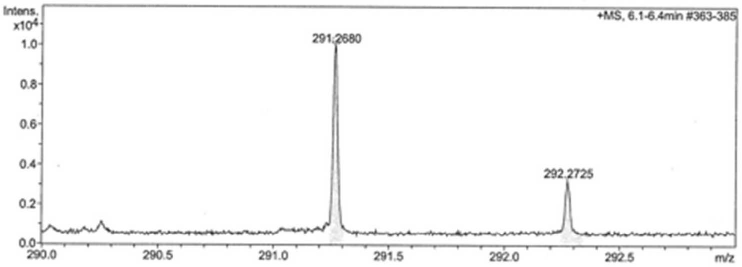


| Meas. m/z | # | Ion Formula | m/z | err [ppm] | mSigma | # mSigma | Score | rdb | e⁻ Conf | N-Rule | \|err\| [mDa] |
|---|---|---|---|---|---|---|---|---|---|---|---|
| 291.2680 | 1 | C20H35O | 291.2682 | 0.8 | 572.8 | 1 | 100.00 | 3.5 | even | ok | 0.2 |
| | 2 | C13H35N6O | 291.2867 | 64.2 | 580.9 | 2 | 0.00 | -0.5 | even | ok | 18.7 |

CLI_9279_2.d

Bruker Compass DataAnalysis 4.2 printed: 5/17/2015 9:06:36 AM by: Larisa Panz Page 1 of 1

Figure S20. $^{1}$H NMR spectrum of (3*R*)-13,18-dimethylnonadec-(4*E*)-en-1-yn-3-ol (**4**) in $CDCl_3$

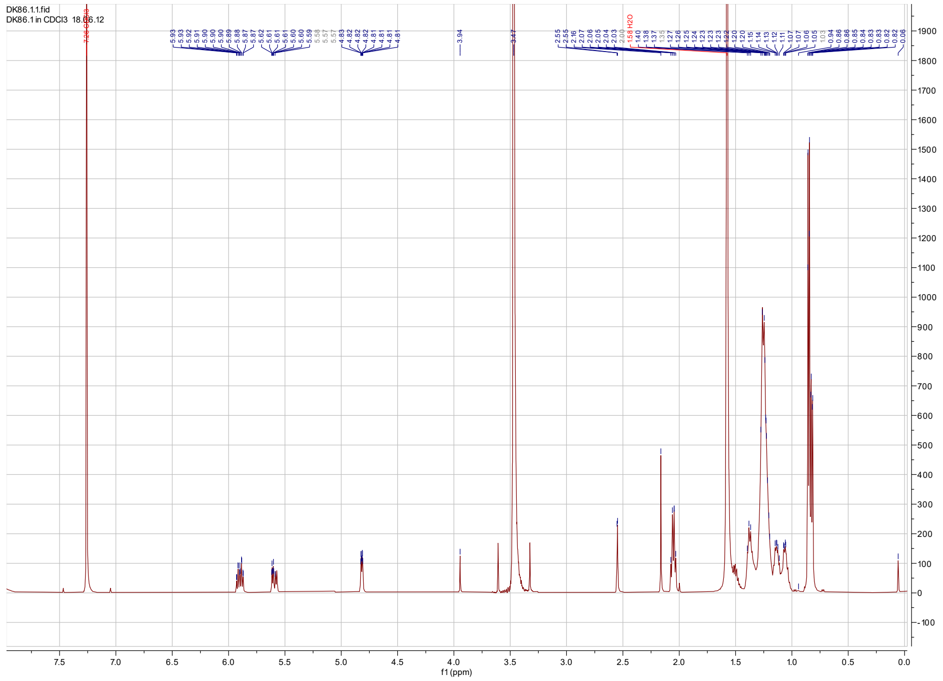

Figure S21. $^{13}$C NMR spectrum of (3*R*)-13,18-dimethylnonadec-(4*E*)-en-1-yn-3-ol (**4**) in $CDCl_3$

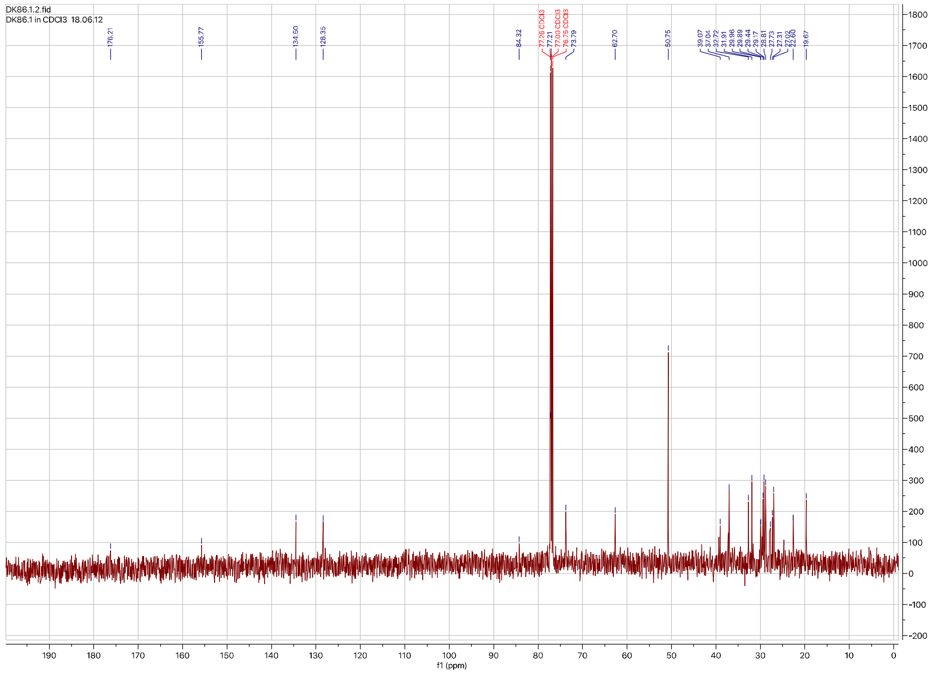

Table S4. NMR data of (3*R*)-13,18-dimethylnonadec-(4*E*)-en-1-yn-3-ol (**4**) in $CDCl_3$.[a]

| Position | $\delta_C$, mult.[b] | $\delta_H$, mult. *J* (Hz) | LR H-C Correlations[c] |
|---|---|---|---|
| 1 | 73.8[d] CH | 2.56 d (2.5) | 3 |
| 2 | 83.3[e] qC | - | 1, 3, 4 |
| 3 | 62.7 CH | 4.82 brd (6.0) | 1, 4, 5 |
| 4 | 128.3 CH | 5.59 dd (15.0, 6.0) | 3, 6 |
| 5 | 134.5 CH | 5.90 dt (15.0, 7.5) | 3, 6, 7 |
| 6 | 31.9 $CH_2$ | 2.05 q (7.0) | 4, 5, 7, 8 |
| 7 | 28.8 $CH_2$ | 1.40 m | 5, 6, 8 |
| 8-10 | ~29.6[f] 3 × $CH_2$ | 1.23 – 1.26 brm | |
| 11 | 27.0 $CH_2$ | 1.25 m | 10, 12a, 12b |
| 12a | 37.0 $CH_2$ | 1.26 m | 11, 13, 14a, 14b, 21 |
| b | | 1.07 m | |
| 13 | 32.7 CH | 1.35 m | 12a, 12b, 14a, 14b, 21 |
| 14a | 37.0 $CH_2$ | 1.26 m | 12a, 12b, 13, 15, 21 |
| b | | 1.07 m | |
| 15 | 27.0 $CH_2$ | 1.25 m | 14a, 14b, 16 |
| 16 | 27.7 $CH_2$ | 1.25 m | 15, 17 |
| 17 | 39.3 $CH_2$ | 1.15 m | 16, 18, 19, 20 |
| 18 | 27.9 CH | 1.51 m | 17, 19, 20 |
| 19 | 22.6 $CH_3$ | 0.86 d (7.0) | 17, 18, 20 |
| 20 | 22.6 $CH_3$ | 0.86 d (7.0) | 17, 18, 19 |
| 21 | 19.7 $CH_3$ | 0.82 d (6.5) | 12b, 14b |

[a]500.13 MHz for $^1H$ and 125.76 MHz for $^{13}C$; [b]Multiplicity and assignment from HSQC experiment; [c]Determined from HMBC experiment; [d] $^1J$ = 250.0 Hz; [e] $^2J$ = 49.0 Hz; [f]Exact $^{13}C$ chemical shifts 29.16, 29.44, 29.45 ppm.

Figure S22. Measured and calculated $^{13}C$ NMR data of (3*R*)-13,18-dimethylnonadec-(4*E*)-en-1-yn-3-ol (**4**).

Measured

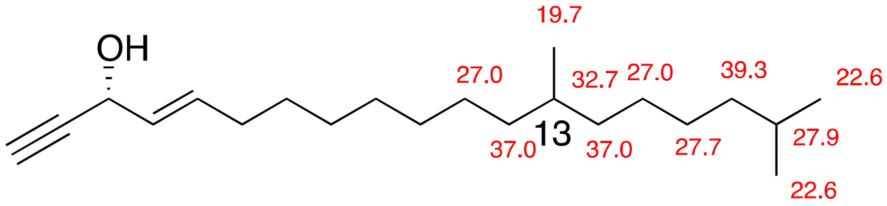


Calculated

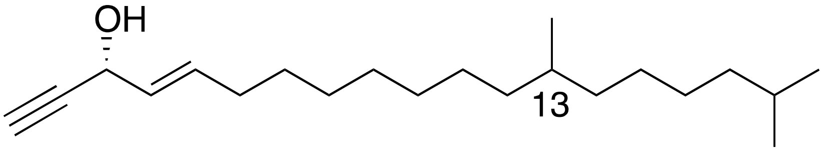


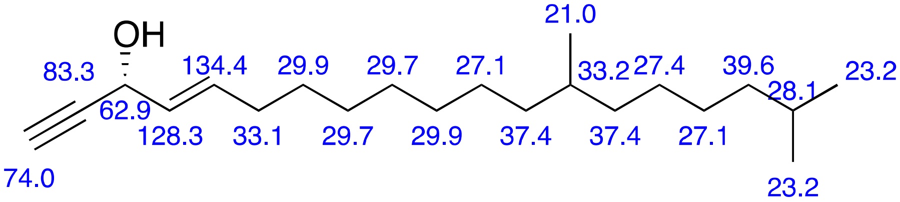


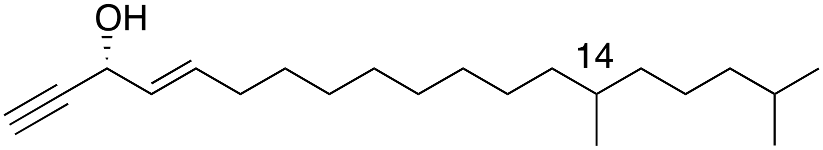


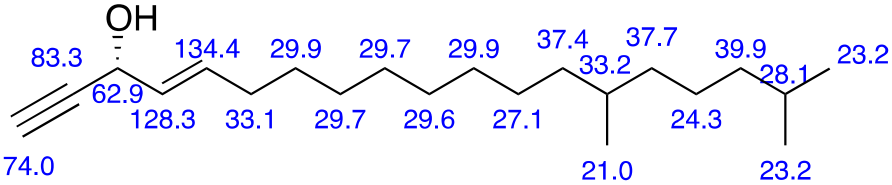

Figure S23. EIMS and fragmentation pattern of (3*R*)-13,18-dimethylnonadec-(4*E*)-en-1-yn-3-ol (**4**).

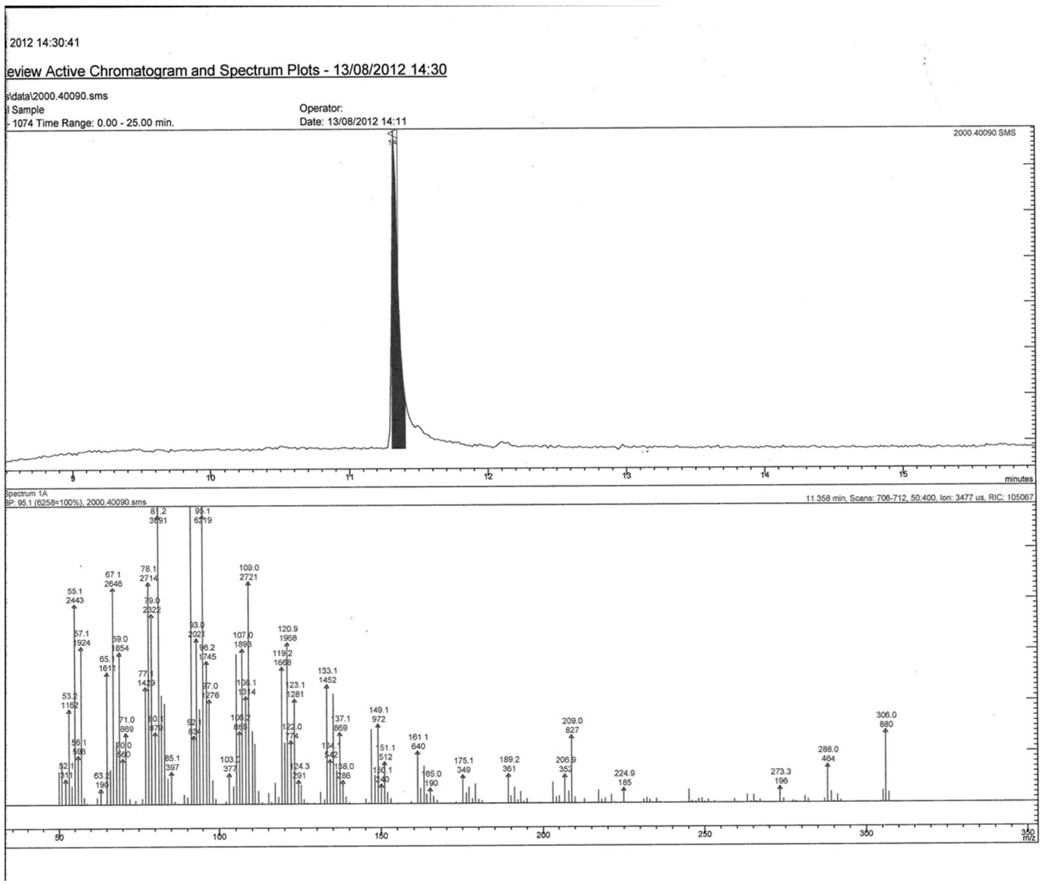


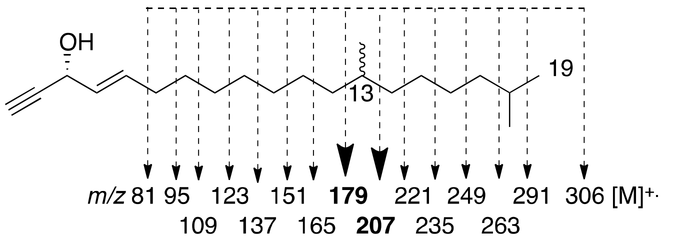

Figure S24. HRMS and fragmentation pattern of (3*R*)-13,18-dimethylnonadec-(4*E*)-en-1-yn-3-ol (**4**)

## Compound Spectrum SmartFormula Report

**Analysis Info**

| | | | |
|---|---|---|---|
| Analysis Name | D:\Data\Client\CLI_9279_6.d | Acquisition Date | 5/17/2015 10:20:26 AM |
| Method | CLI_9279.m | Operator | Larisa Panz |
| Sample Name | DK_86-1(9) | Instrument | maXis impact 282001.00128 |
| Comment | | | |

**Acquisition Parameter**

| | | | | | |
|---|---|---|---|---|---|
| Source Type | APCI | Ion Polarity | Positive | Set Nebulizer | 4.0 Bar |
| Focus | Active | Set Capillary | 4000 V | Set Dry Heater | 200 °C |
| Scan Begin | 50 m/z | Set End Plate Offset | -500 V | Set Dry Gas | 2.0 l/min |
| Scan End | 1000 m/z | Set Charging Voltage | 2000 V | Set Divert Valve | Source |
| | | Set Corona | 4000 nA | Set APCI Heater | 450 °C |

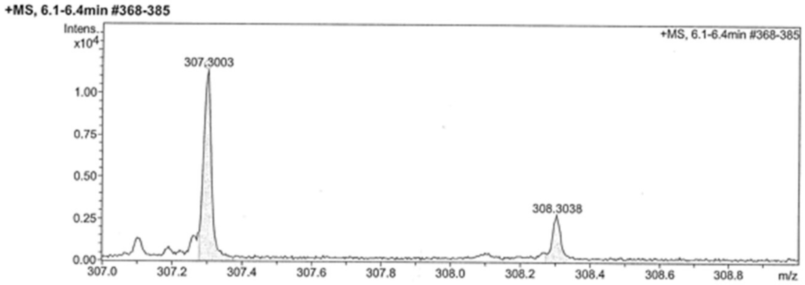


| Meas. m/z | # | Ion Formula | m/z | err [ppm] | mSigma | # mSigma | Score | rdb | e⁻ Conf | N-Rule | \|err\| [mDa] |
|---|---|---|---|---|---|---|---|---|---|---|---|
| 307.3003 | 1 | C21H39O | 307.2995 | -2.5 | 410.3 | 1 | 100.00 | 2.5 | even | ok | 0.8 |

Figure S25. $^{1}$H NMR spectrum of (3*R*)-14-methylicos-(4*E*)-en-1-yn-3-ol (**5**) in $CDCl_3$.

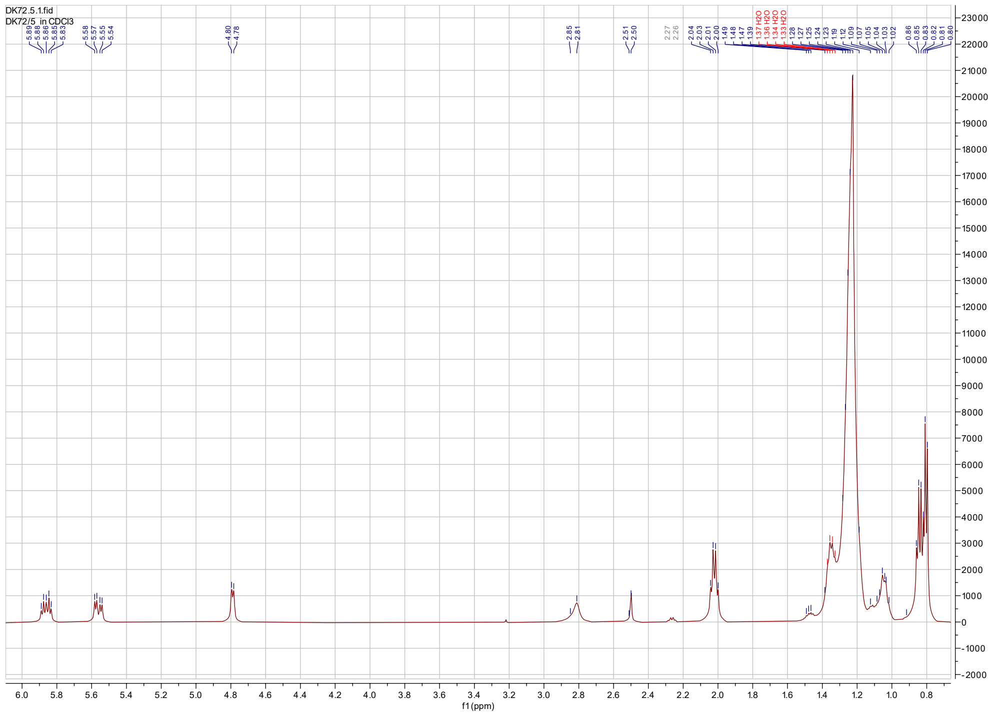


Figure S26. $^{13}$C NMR spectrum of (3*R*)-14-methylicos-(4*E*)-en-1-yn-3-ol (**5**) in $CDCl_3$.

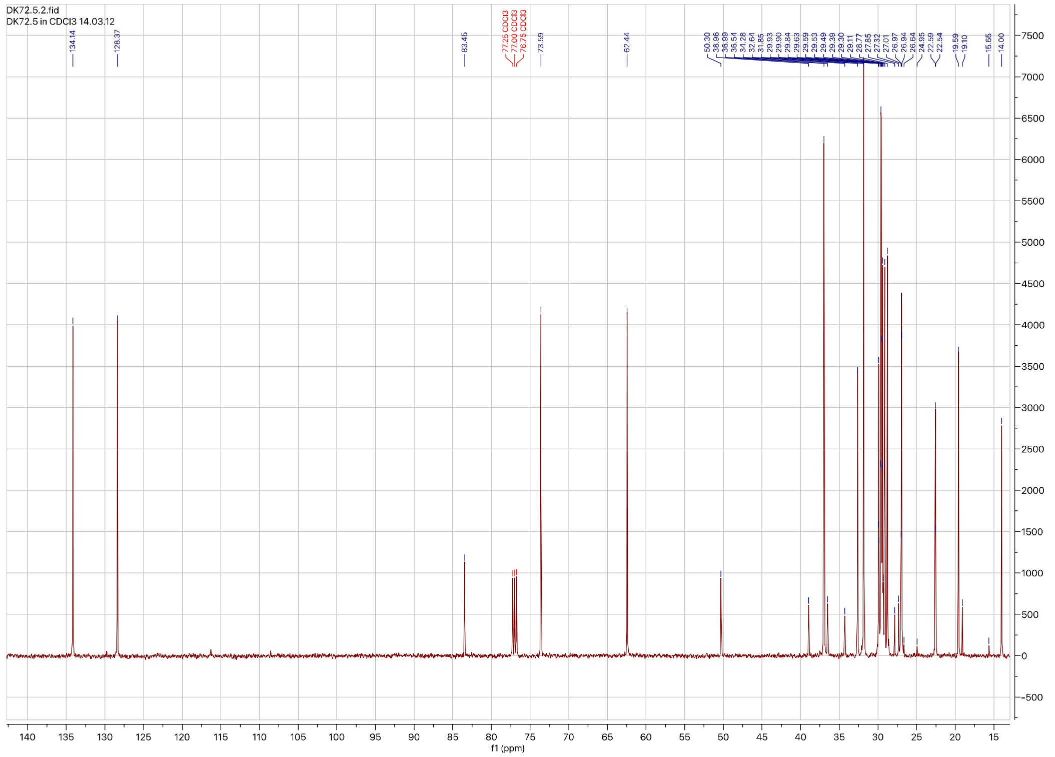

Figure S27. HSQC spectrum of (3*R*)-14-methylicos-(4*E*)-en-1-yn-3-ol (**5**) in $CDCl_3$.

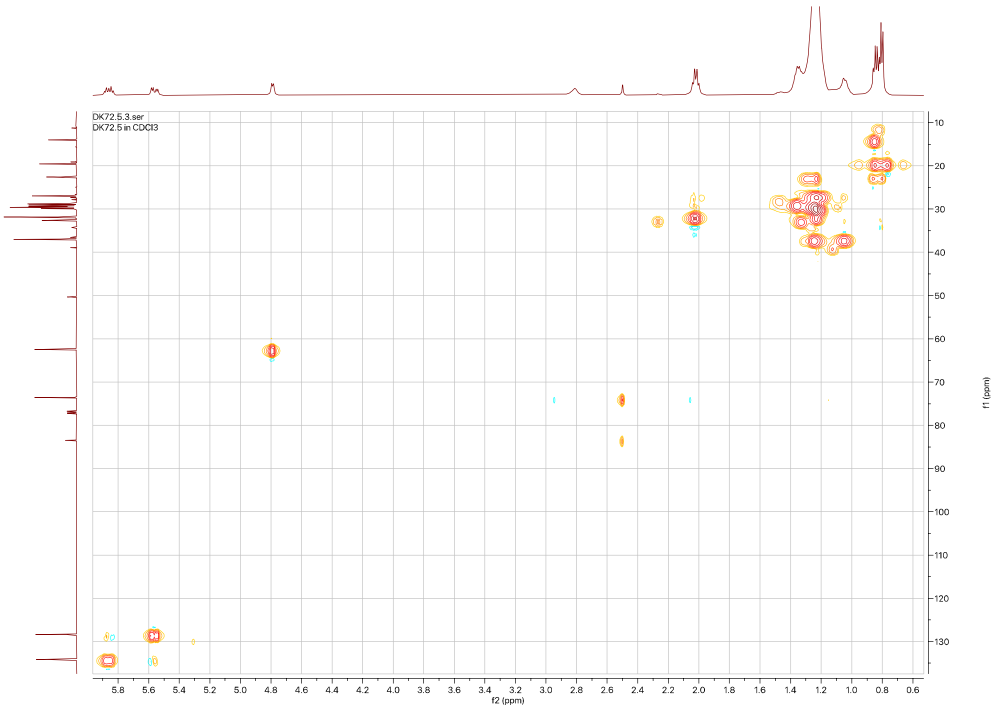


Figure S28. HMBC spectrum of (3*R*)-14-methylicos-(4*E*)-en-1-yn-3-ol (**5**) in $CDCl_3$.

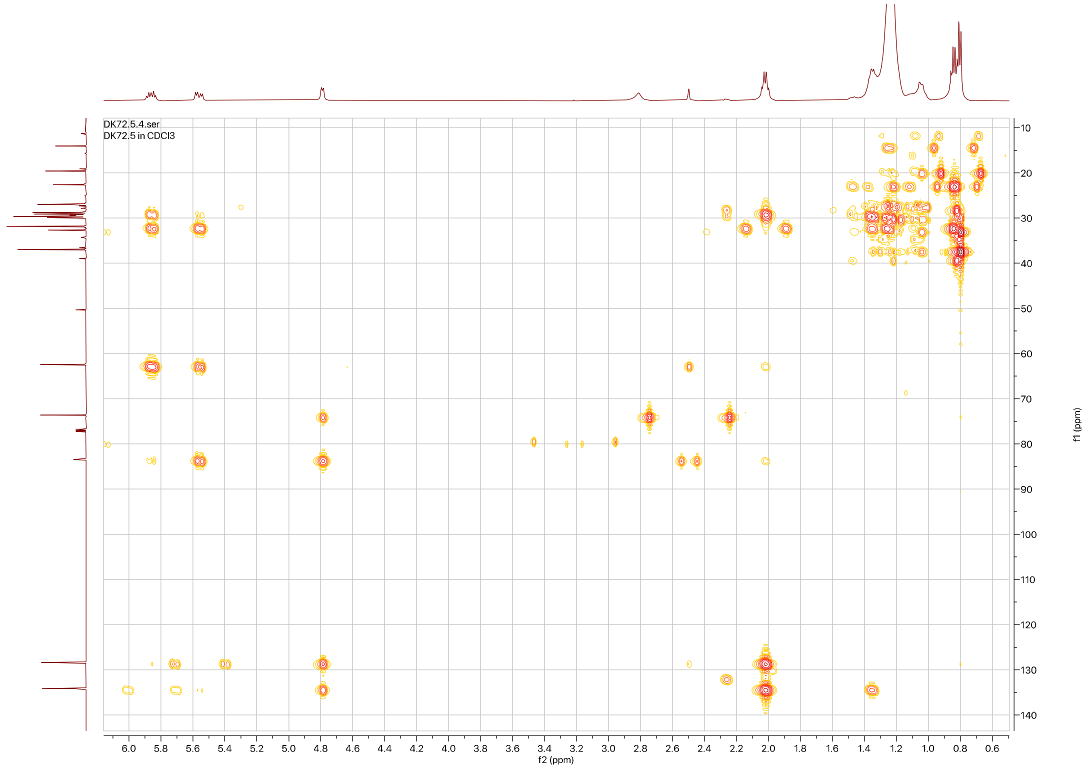

Figure S29. COSY spectrum of (3*R*)-14-methylicos-(4*E*)-en-1-yn-3-ol (**5**) in $CDCl_3$.

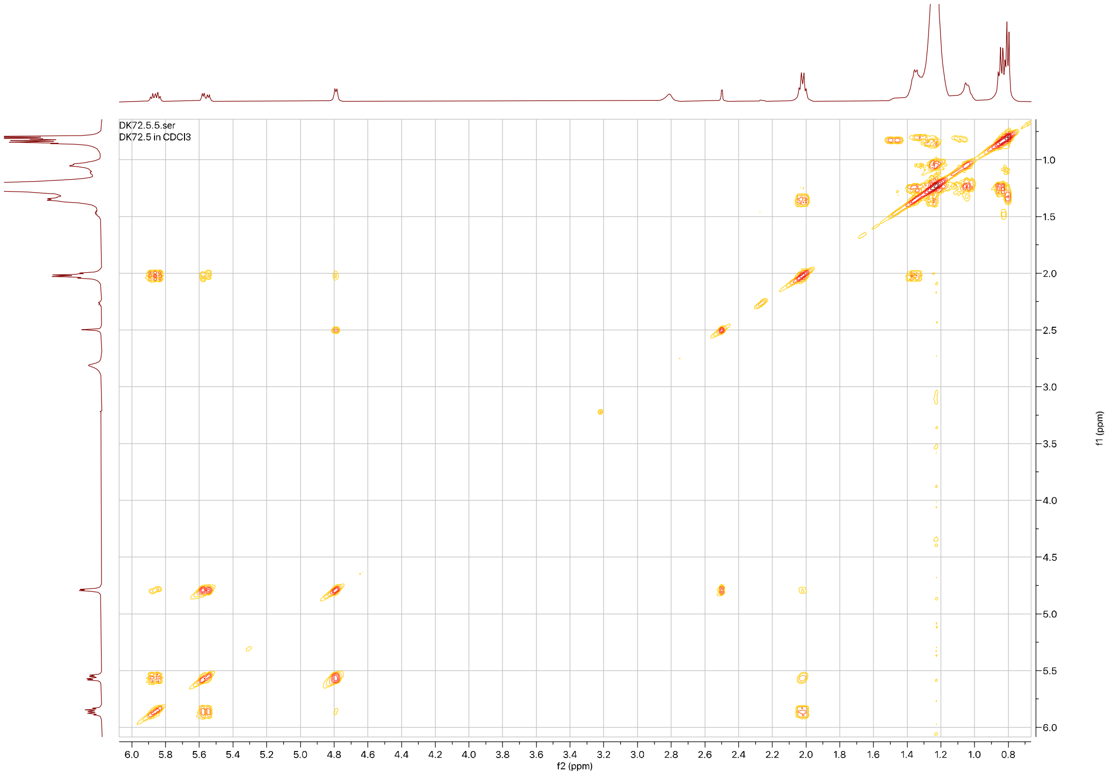

Table S5. NMR data of (3*R*)-14-methylicos-(4*E*)-en-1-yn-3-ol (**5**) in $CDCl_3$.[a]

| Position | $\delta_C$, mult.[b] | $\delta_H$, mult. *J* (Hz) | LR H-C Correlations[c] |
|---|---|---|---|
| 1 | 73.8[d] CH | 2.50 d (2.1) | 3 |
| 2 | 83.4[e] qC | - | 1, 3, 4 |
| 3 | 62.6 CH | 4.79 d (6.0) | 1, 4, 5 |
| 4 | 128.4 CH | 5.56 dd (15.0, 6.0) | 3, 6 |
| 5 | 134.3 CH | 5.85 dt (15.0, 6.8) | 3, 6, 7 |
| 6 | 31.9 $CH_2$ | 2.02 q (6.8) | 4, 5, 7, 8 |
| 7 | 28.8 $CH_2$ | 1.36 m | 5, 6, 8 |
| 8-11 | ~29.6[f] 4 × $CH_2$ | 1.21 – 1.26 brm | |
| 12 | 27.0 $CH_2$ | 1.23 m | 11, 13a, 13b |
| 13a | 37.0 $CH_2$ | 1.25 m | 12, 14, 15a, 15b, 21 |
| b | | 1.08 m | |
| 14 | 32.7 CH | 1.34 m | 13a, 13b, 15a, 15a, 21 |
| 15a | 37.0 $CH_2$ | 1.25 m | 13a, 13b, 14, 16, 21 |
| b | | 1.08 m | |
| 16 | 27.0 $CH_2$ | 1.23 m | 15a, 15b, 17 |
| 17 | ~29.6[f] $CH_2$ | 1.21 – 1.26 brm | |
| 18 | 31.9 $CH_2$ | 1.23 m | 17, 19a, 19b, 20 |
| 19a | 22.6 $CH_2$ | 1.29 m | 18, 20 |
| b | | 1.23 m | |
| 20 | 14.0 $CH_3$ | 0.87 t (6.6) | 19a, 19b |
| 21 | 19.6 $CH_3$ | 0.83 d (6.6) | 13b, 15b |

[a]500.13 MHz for $^{1}H$ and 125.76 MHz for $^{13}C$; [b]Multiplicity and assignment from HSQC experiment; [c]Determined from HMBC experiment; [d] $^{1}J$ = 250.0 Hz; [e] $^{2}J$ = 48.8 Hz; [f]Exact $^{13}C$ chemical shifts 29.15, 29.43, 29.57, 29.63, 29.95 ppm.

Figure S30. EIMS and fragmentation pattern of (3*R*)-14-methylicos-(4*E*)-en-1-yn-3-ol (**5**).

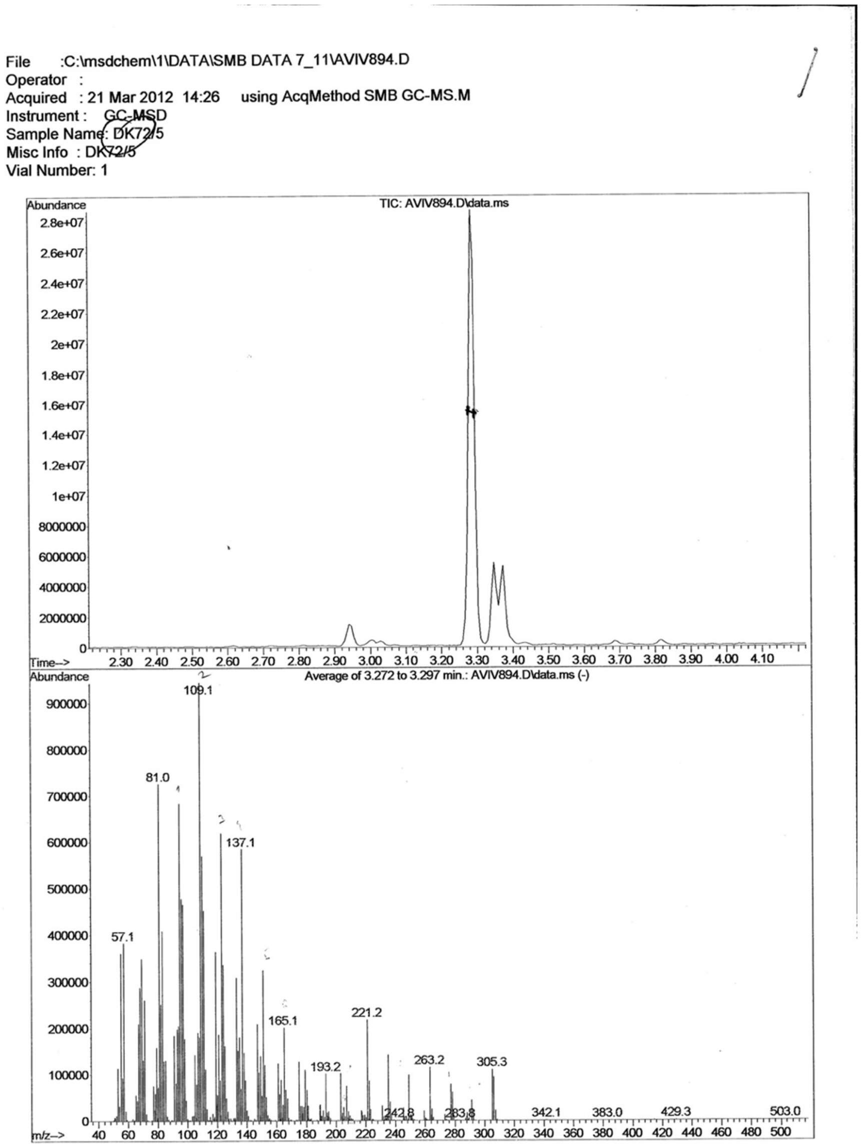


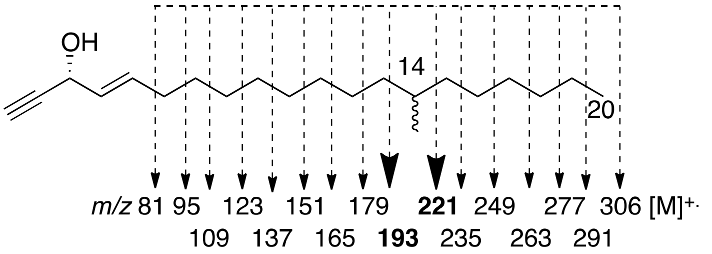

Figure S31. HRMS and fragmentation pattern of (3*R*)-14-methylicos-(4*E*)-en-1-yn-3-ol (**5**).

## Compound Spectrum SmartFormula Report

**Analysis Info**

Acquisition Date 5/17/2015 1:19:29 PM

Analysis Name D:\Data\Client\CLI_9279_21000001.d
Method GC APCI_pos_cli_8051.m
Sample Name DK107.1(7)
Comment

Operator Larisa Panz
Instrument maXis impact 282001.00128

**Acquisition Parameter**

| | | | | | |
|---|---|---|---|---|---|
| Source Type | APCI | Ion Polarity | Positive | Set Nebulizer | 4.0 Bar |
| Focus | Active | Set Capillary | 4000 V | Set Dry Heater | 200 °C |
| Scan Begin | 50 m/z | Set End Plate Offset | -500 V | Set Dry Gas | 2.0 l/min |
| Scan End | 1000 m/z | Set Charging Voltage | 2000 V | Set Divert Valve | Source |
| | | Set Corona | 4000 nA | Set APCI Heater | 450 °C |

**+MS, 5.4-5.8min #325-347**

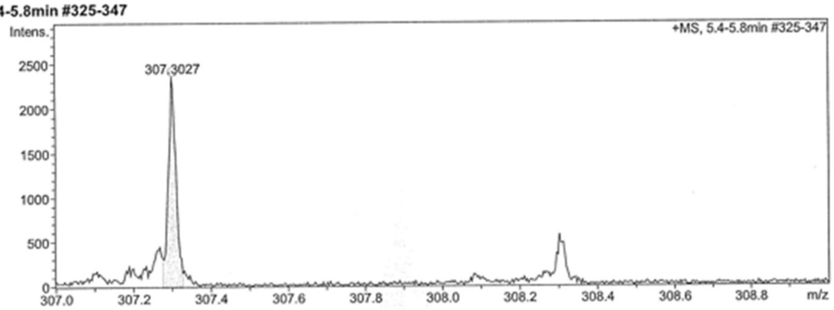


| Meas. m/z | # | Ion Formula | m/z | err [ppm] | mSigma | # mSigma | Score | rdb | e⁻ Conf | N-Rule | \|err\| [mDa] |
|---|---|---|---|---|---|---|---|---|---|---|---|
| 307.3027 | 1 | C21H39O | 307.2995 | -10.1 | 175.1 | 1 | 100.00 | 2.5 | even | ok | 3.1 |

Figure S32. $^{1}$H NMR spectrum of 14-methylicos-(4*E*)-en-1-yn-3-one (**6**) in $CDCl_3$.

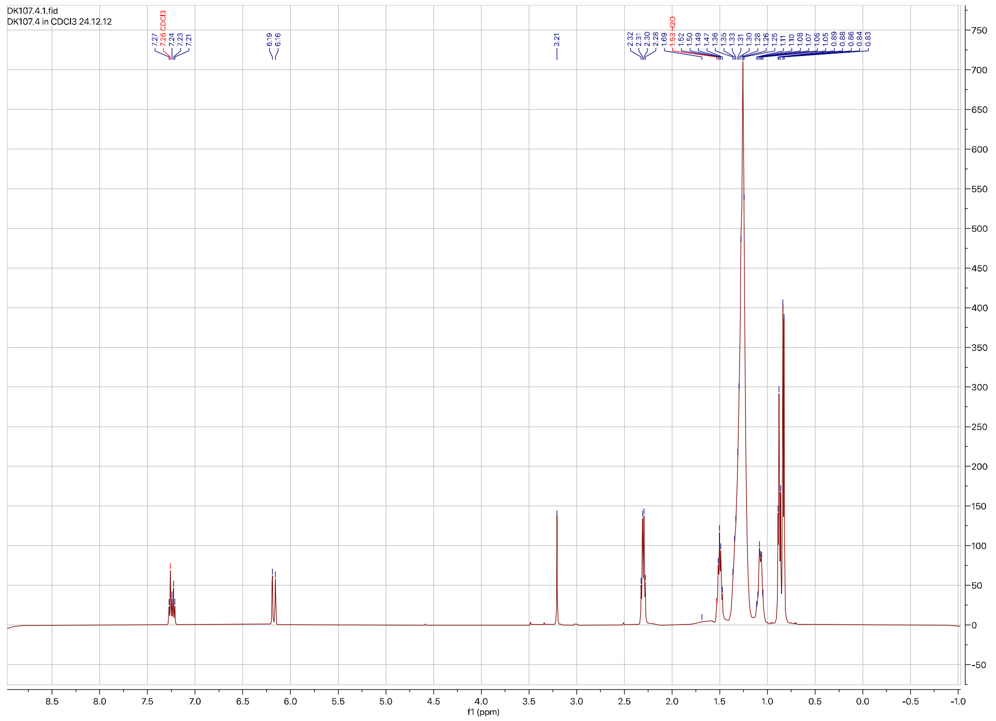


Figure S33. $^{13}$C NMR spectrum of 14-methylicos-(4*E*)-en-1-yn-3-one (**6**) in $CDCl_3$.

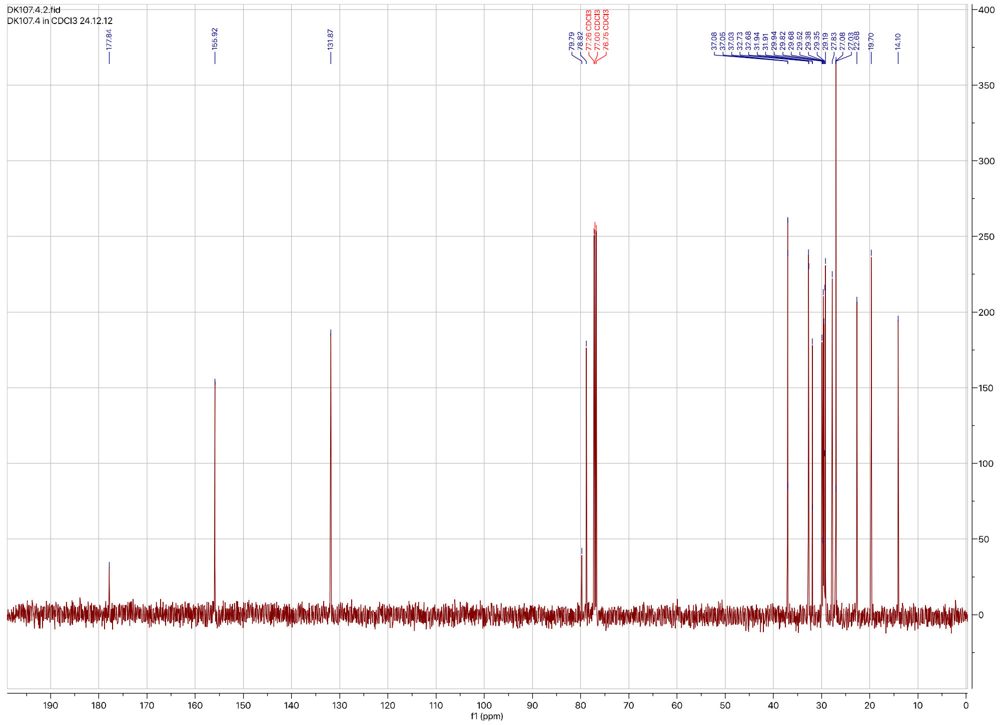

Table S6. NMR data of 14-methylicos-(4*E*)-en-1-yn-3-one (**6**) in $CDCl_3$.[a]

| Position | $\delta_C$, mult.[b] | $\delta_H$, mult. *J* (Hz) | LR H-C Correlations[c] |
|---|---|---|---|
| 1 | 78.8[d] CH | 3.21 s | - |
| 2 | 79.8[e] qC | - | 1, 4 |
| 3 | 177.8 qC | - | 1, 4, 5 |
| 4 | 131.9 CH | 6.17 d (16.0) | 6 |
| 5 | 155.9 CH | 7.24 dt (16.0, 7.0) | 6, 7 |
| 6 | 32.7 $CH_2$ | 2.30 q (7.0) | 4, 5, 7, 8 |
| 7 | 27.8 $CH_2$ | 1.50 tt (7.0, 7.0) | 5, 6, 8 |
| 8 | 29.2 $CH_2$ | 1.30 m | |
| 9-11 | ~29.6[f] 3 × $CH_2$ | 1.23 – 1.31 brm | |
| 12 | 27.0 $CH_2$ | 1.23 m | 11, 13a, 13b |
| 13a | 37.1 $CH_2$ | 1.26 m | 12, 14, 15a, 15b, 21 |
| b | | 1.07 m | |
| 14 | 32.7 CH | 1.37 m | 13a, 13b, 15a, 15a, 21 |
| 15a | 37.1 $CH_2$ | 1.26 m | 13a, 13b, 14, 16, 21 |
| b | | 1.07 m | |
| 16 | 27.0 $CH_2$ | 1.25 m | 15a, 15b, 17 |
| 17 | 29.9 $CH_2$ | 1.26 m | |
| 18 | 31.9 $CH_2$ | 1.25 m | 17, 19a, 19b, 20 |
| 19a | 22.7 $CH_2$ | 1.31 m | 18, 20 |
| b | | 1.25 m | |
| 20 | 14.1 $CH_3$ | 0.88 t (6.5) | 19a, 19b |
| 21 | 19.7 $CH_3$ | 0.83 d (6.5) | 13b, 15b |

[a]500.13 MHz for $^1H$ and 125.76 MHz for $^{13}C$; [b]Multiplicity and assignment from HSQC experiment; [c]Determined from HMBC experiment; [d] $^1J$ = 253.5 Hz; [e] $^2J$ = 48.0 Hz; [f]Exact $^{13}C$ chemical shifts 29.34, 29.51, 29.67ppm.

Figure S34. EIMS and fragmentation pattern of 14-methylicos-(4*E*)-en-1-yn-3-one (**6**)

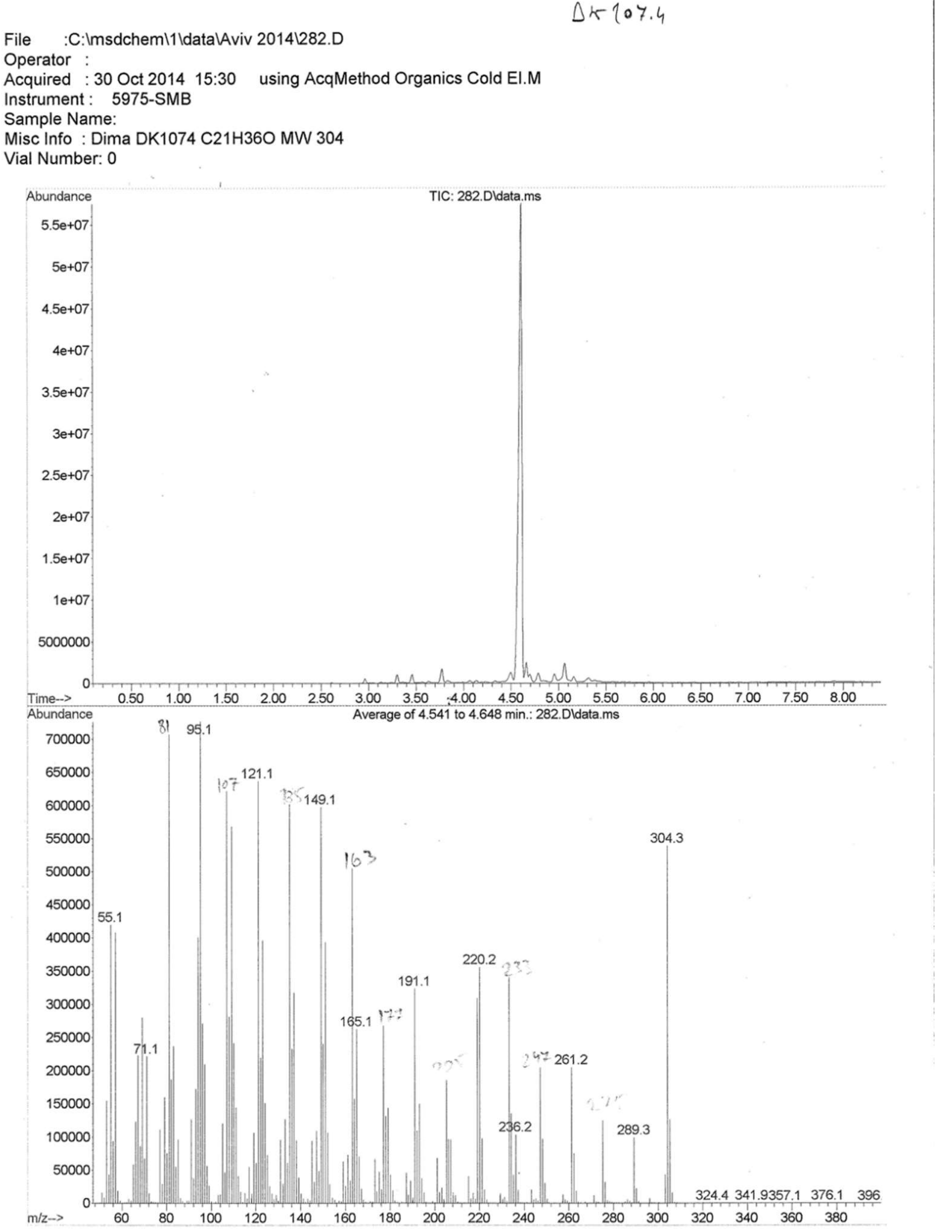


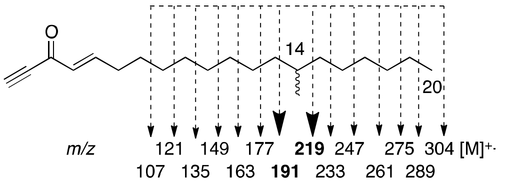

Figure S35. HRCIMS and fragmentation pattern of 14-methylicos-(4*E*)-en-1-yn-3-one (**6**)

## Compound Spectrum SmartFormula Report

**Analysis Info**

| | | | |
|---|---|---|---|
| Analysis Name | D:\Data\Client\CLI_9279_9.d | Acquisition Date | 5/17/2015 11:06:37 AM |
| Method | CLI_9279.m | Operator | Larisa Panz |
| Sample Name | DK_107.4(20) (70) | Instrument | maXis impact 282001.00128 |
| Comment | | | |

**Acquisition Parameter**

| | | | | | |
|---|---|---|---|---|---|
| Source Type | APCI | Ion Polarity | Positive | Set Nebulizer | 4.0 Bar |
| Focus | Active | Set Capillary | 4000 V | Set Dry Heater | 200 °C |
| Scan Begin | 50 m/z | Set End Plate Offset | -500 V | Set Dry Gas | 2.0 l/min |
| Scan End | 1000 m/z | Set Charging Voltage | 2000 V | Set Divert Valve | Source |
| | | Set Corona | 4000 nA | Set APCI Heater | 450 °C |

**+MS, 6.7-7.0min #402-421**

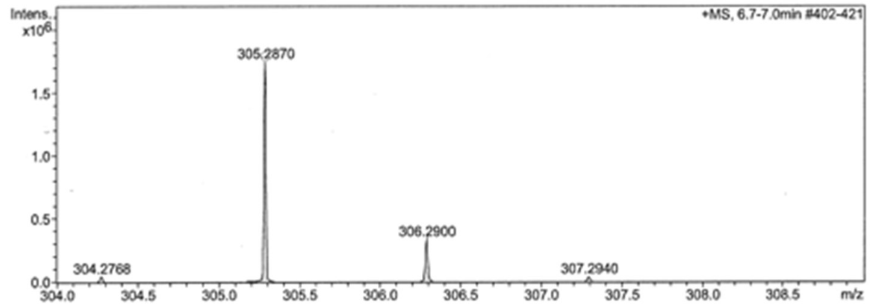


| Meas. m/z | # | Ion Formula | m/z | err [ppm] | mSigma | # mSigma | Score | rdb | e⁻ Conf | N-Rule | \|err\| [mDa] |
|---|---|---|---|---|---|---|---|---|---|---|---|
| 305.2870 | 1 | C14H37N6O | 305.3023 | 50.2 | 10.0 | 1 | 0.00 | -0.5 | even | ok | 15.3 |
| | 2 | C21H37O | 305.2839 | -10.2 | 22.2 | 2 | 100.00 | 3.5 | even | ok | 3.1 |

CLI_9279_9.d
Bruker Compass DataAnalysis 4.2 printed: 5/17/2015 11:16:57 AM by: Larisa Panz Page 1 of 1

Figure S36. $^{1}$H NMR spectrum of (3*R*)-14-methylicos-1-yn-3-ol (**7**) in $CDCl_3$

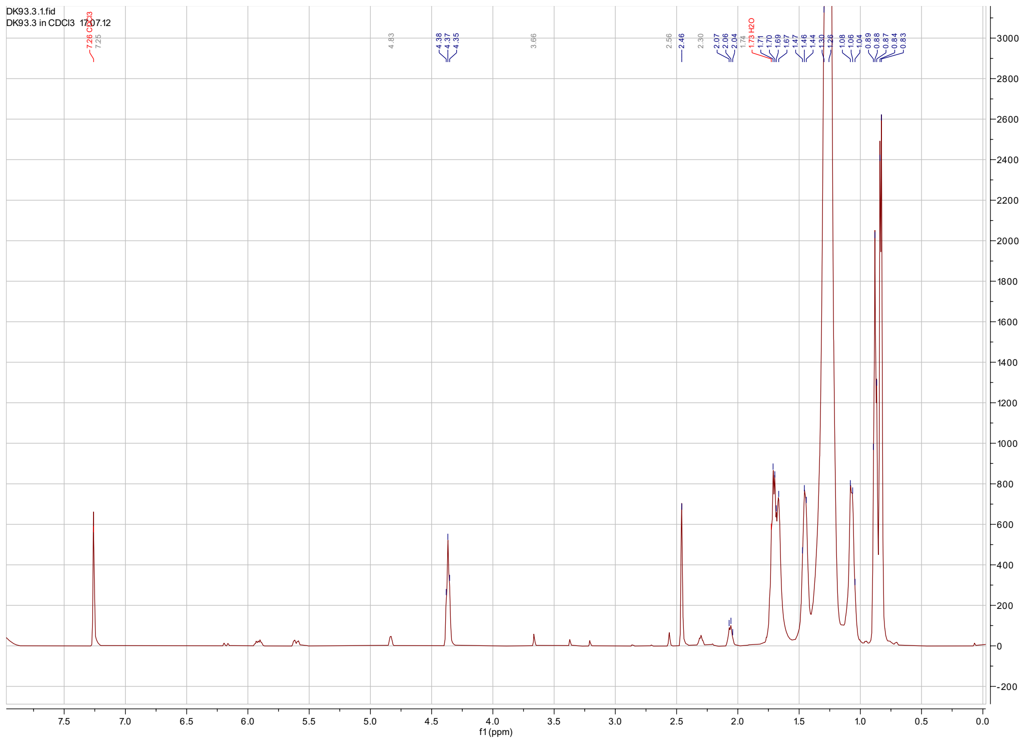

Figure S35. $^{13}$C NMR spectrum of (3*R*)-14-methylicos-1-yn-3-ol (**7**) in $CDCl_3$

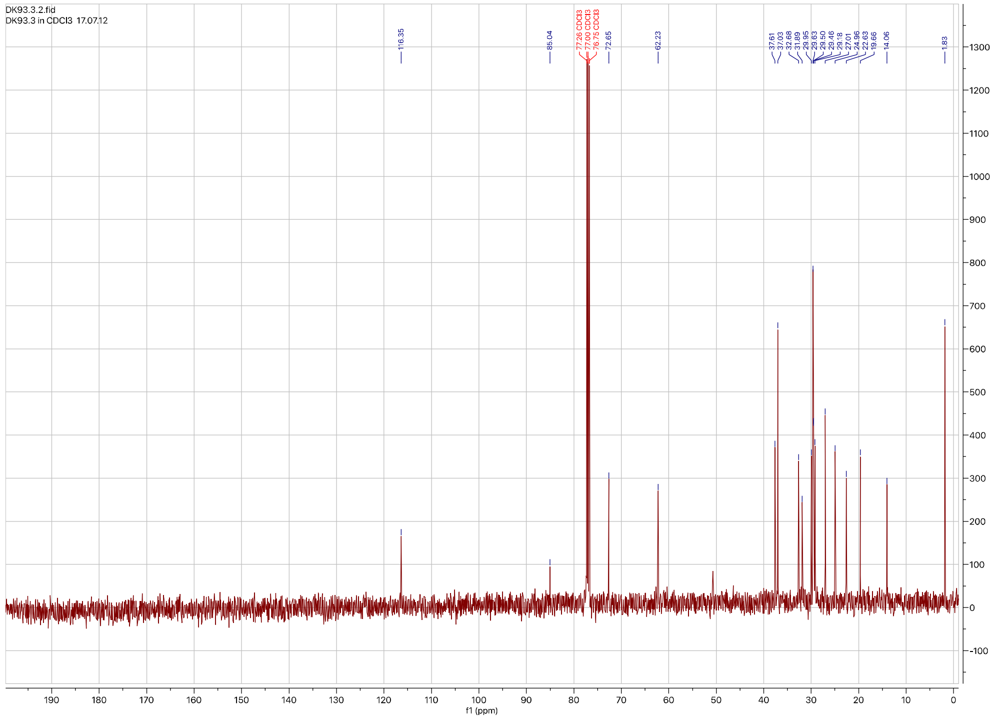

Figure S38. HSQC spectrum of (3*R*)-14-methylicos-1-yn-3-ol (**7**) in $CDCl_3$

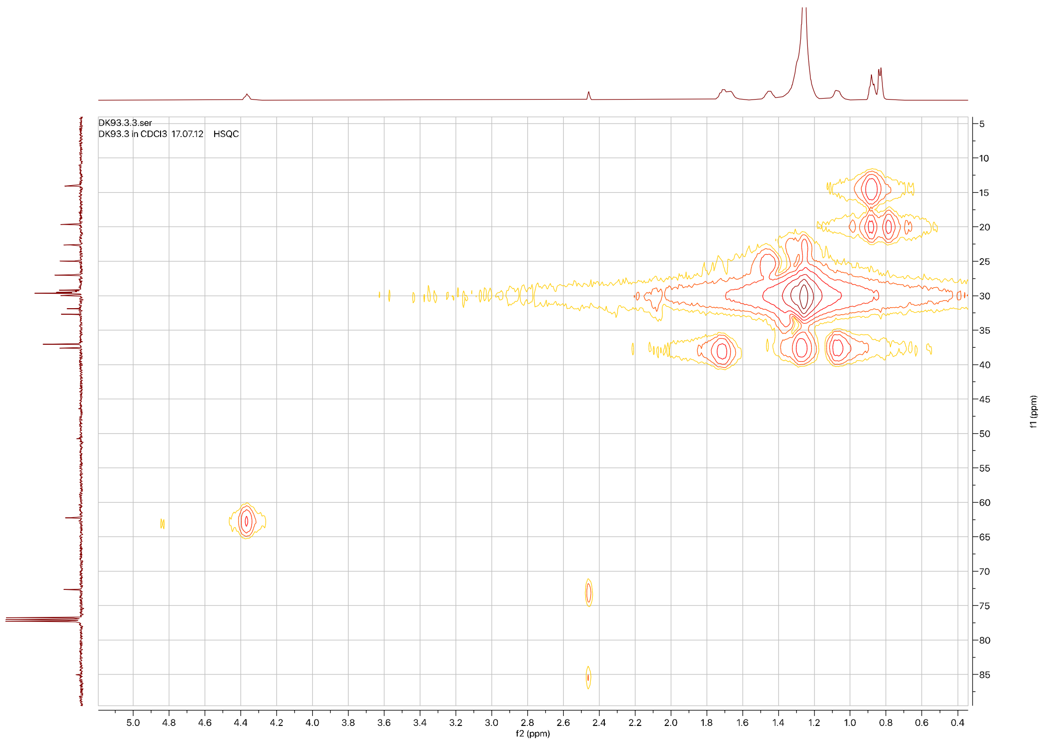


Figure S39. HMBC spectrum of (3*R*)-14-methylicos-1-yn-3-ol (**7**) in $CDCl_3$

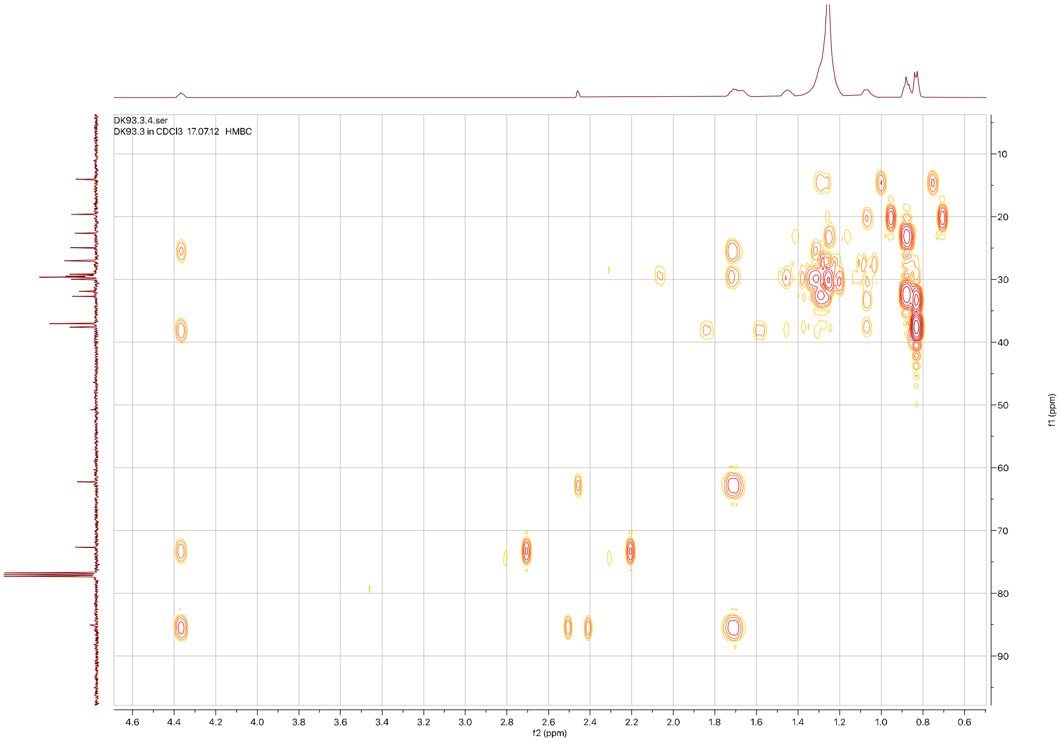

Figure S40. COSY spectrum of (3*R*)-14-methylicos-1-yn-3-ol (**7**) in $CDCl_3$

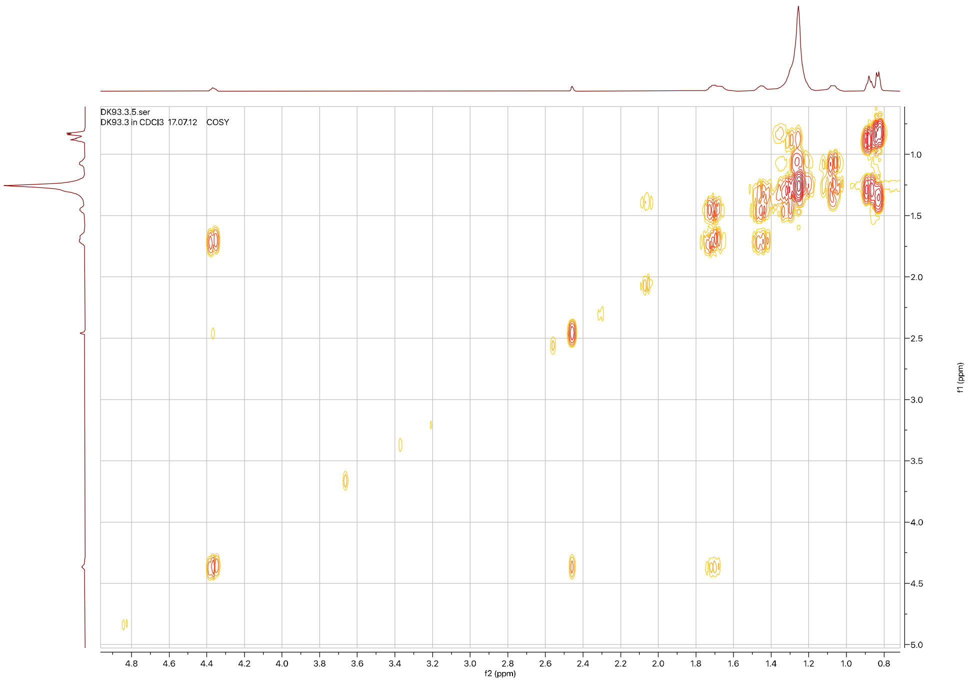


Figure S41. DEPT spectrum of (3*R*)-14-methylicos-1-yn-3-ol (**7**) in $CDCl_3$

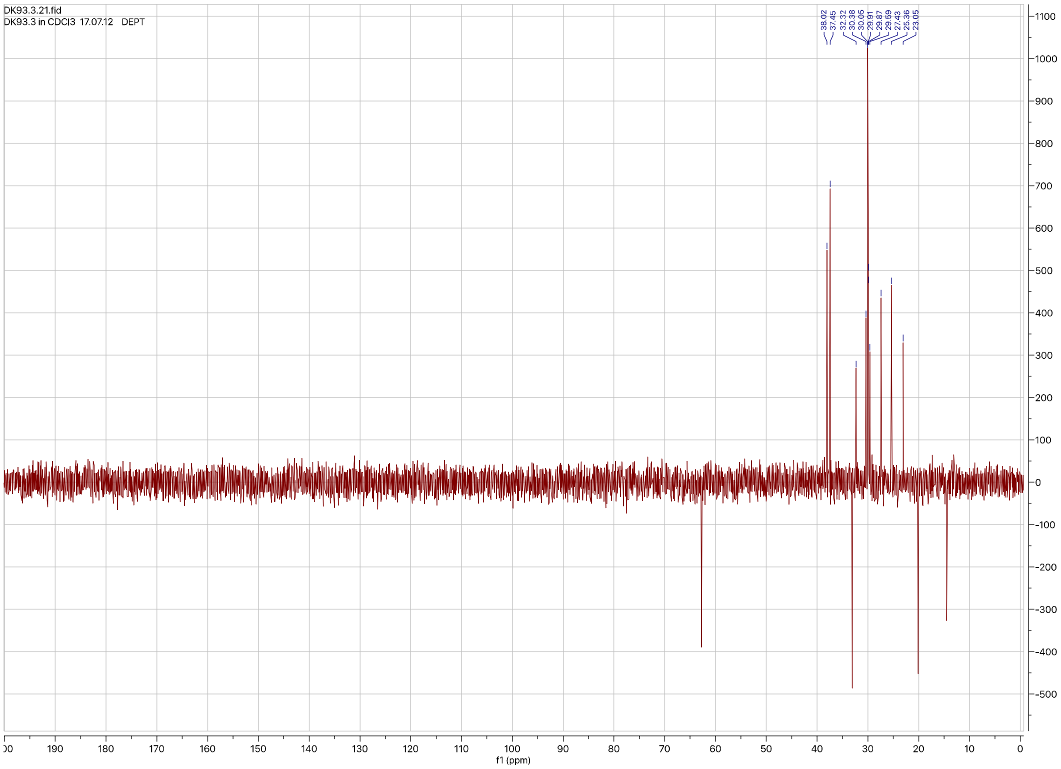

Table S7. NMR data of (3*R*)-14-methylicos-1-yn-3-ol (**7**) in $CDCl_3$.[a]

| Position | $\delta_C$, mult.[b] | $\delta_H$, mult. *J* (Hz) | LR H-C Correlations[c] |
|---|---|---|---|
| 1 | 72.6[d] CH | 2.46 d (2.0) | 3 |
| 2 | 85.0[e] qC | - | 1, 3, 4 |
| 3 | 62.2 CH | 4.36 td (6.5, 2.0) | 1, 4 |
| 4 | 37.6 $CH_2$ | 1.71 m | 3, 6 |
| 5 | 25.0 $CH_2$ | 1.45 m | 3, 4, 6, 7 |
| 6 | 29.2 $CH_2$ | 1.26 m | 4, 5 |
| 7-11 | ~29.5[f] 5 × $CH_2$ | 1.22 – 1.31 brm | |
| 12 | 27.0 $CH_2$ | 1.23 m | 11, 13a, 13b |
| 13a | 37.0 $CH_2$ | 1.25 m | 12, 14, 15a, 15b, 21 |
| b | | 1.07 m | |
| 14 | 32.6 CH | 1.34 m | 13a, 13b, 15a, 15a, 21 |
| 15a | 37.0 $CH_2$ | 1.25 m | 13a, 13b, 14, 16, 21 |
| b | | 1.07 m | |
| 16 | 27.0 $CH_2$ | 1.23 m | 15a, 15b, 17 |
| 17 | 30.0 $CH_2$ | 1.24 m | |
| 18 | 31.9 $CH_2$ | 1.23 m | 17, 19a, 19b, 20 |
| 19a | 22.6 $CH_2$ | 1.29 m | 18, 20 |
| b | | 1.23 m | |
| 20 | 14.1 $CH_3$ | 0.87 t (7.0) | 19a, 19b |
| 21 | 19.6 $CH_3$ | 0.83 d (7.0) | 13b, 15b |

[a]500.13 MHz for $^1H$ and 125.76 MHz for $^{13}C$; [b]Multiplicity and assignment from HSQC experiment; [c]Determined from HMBC experiment; [d] $^1J$ = 251.0 Hz; [e] $^2J$ = 46.0 Hz; [f]Exact $^{13}C$ chemical shifts 29.46, 29.50, 29.58, 29.63 (× 2) ppm.

Figure S42. EIMS and fragmentation pattern of (3*R*)-14-methylicos-1-yn-3-ol (**7**)

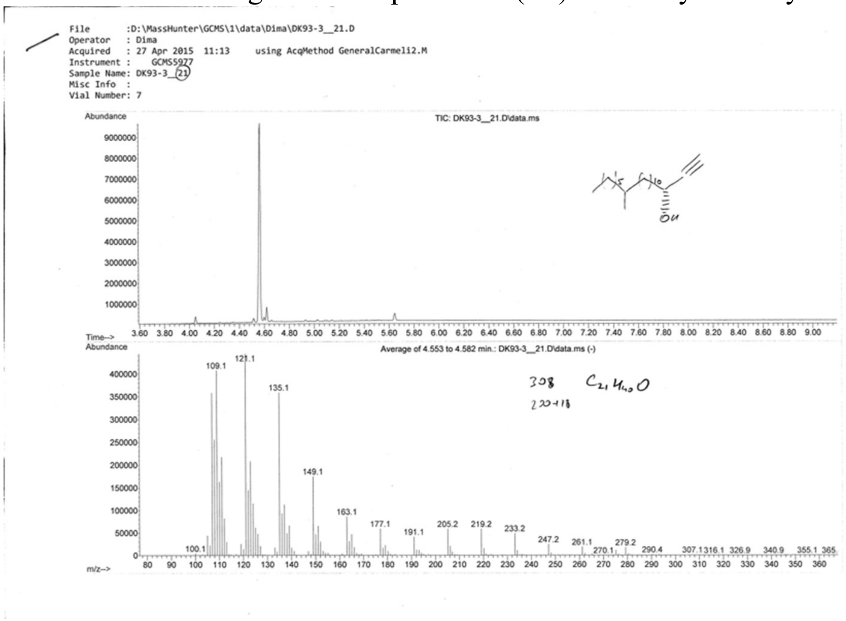


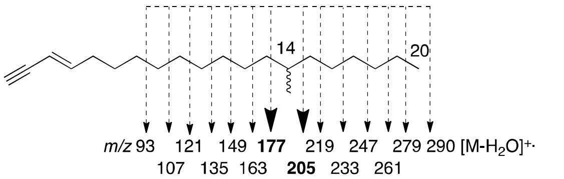


Figure S43. HRMS and fragmentation pattern of (3*R*)-14-methylicos-1-yn-3-ol (**7**)

## Compound Spectrum SmartFormula Report

**Analysis Info**

Analysis Name D:\Data\Client\CLI_9279_5B.d
Method CLI_9279.m
Sample Name DK_93-3(21)
Comment

Acquisition Date 5/17/2015 10:11:00 AM
Operator Larisa Panz
Instrument maXis impact 282001.00128

**Acquisition Parameter**

| | | | | | |
|---|---|---|---|---|---|
| Source Type | APCI | Ion Polarity | Positive | Set Nebulizer | 4.0 Bar |
| Focus | Active | Set Capillary | 4000 V | Set Dry Heater | 200 °C |
| Scan Begin | 50 m/z | Set End Plate Offset | -500 V | Set Dry Gas | 2.0 l/min |
| Scan End | 1000 m/z | Set Charging Voltage | 2000 V | Set Divert Valve | Source |
| | | Set Corona | 4000 nA | Set APCI Heater | 450 °C |

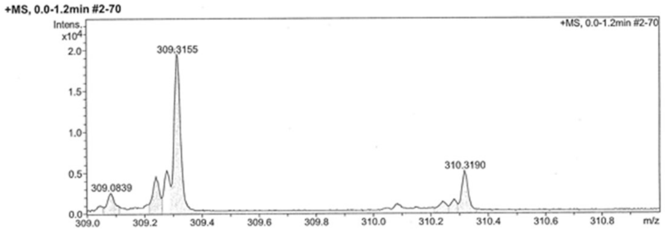


| Meas. m/z | # | Ion Formula | m/z | err [ppm] | mSigma | # mSigma | Score | rdb | e⁻ Conf | N-Rule | \|err\| [mDa] |
|---|---|---|---|---|---|---|---|---|---|---|---|
| 309.3155 | 1 | C21H41O | 309.3152 | -1.0 | 162.4 | 1 | 100.00 | 1.5 | even | ok | 0.3 |

Figure S44. $^{1}$H NMR spectrum of (3*R*,*E*)-12-*cis*-(2-hexylcyclopropyl)dodec-4-en-1-yn-3-ol (**8**) in $CDCl_3$.

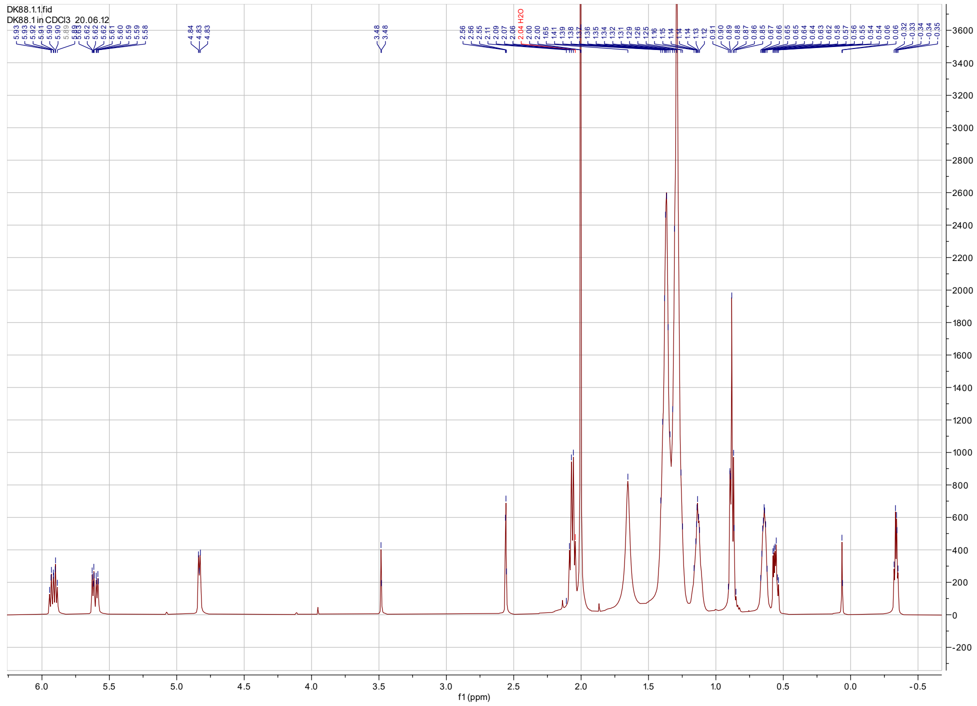

Figure S45. $^{13}$C NMR spectrum of (3*R*,*E*)-12-*cis*-(2-hexylcyclopropyl)dodec-4-en-1-yn-3-ol (**8**) in $CDCl_3$.

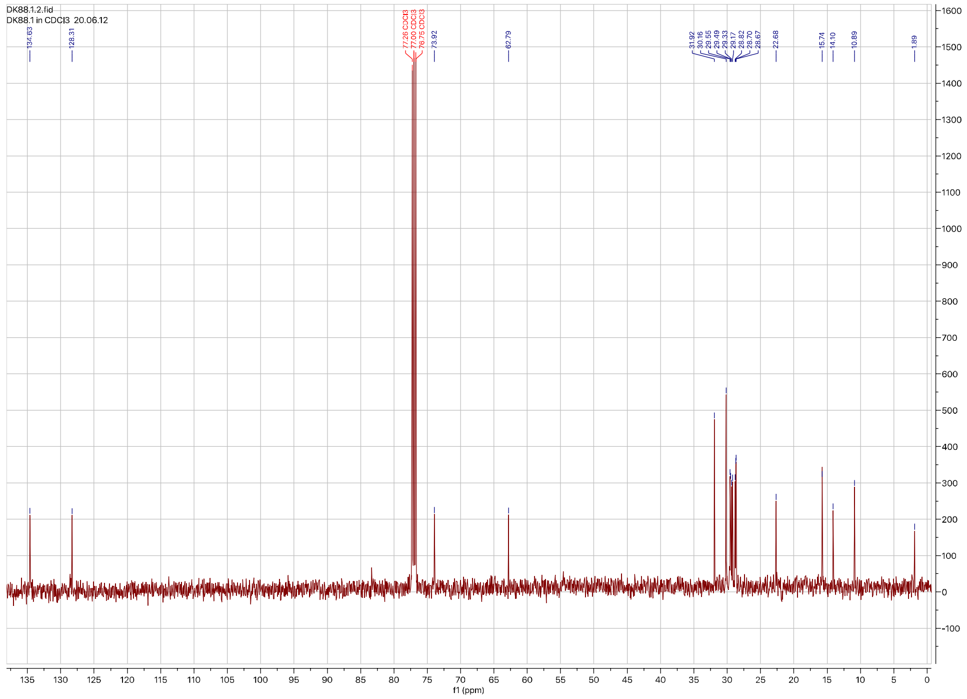

Figure S46. HSQC spectrum of (3*R*,*E*)-12-*cis*-(2-hexylcyclopropyl)dodec-4-en-1-yn-3-ol (**8**) in $CDCl_3$.

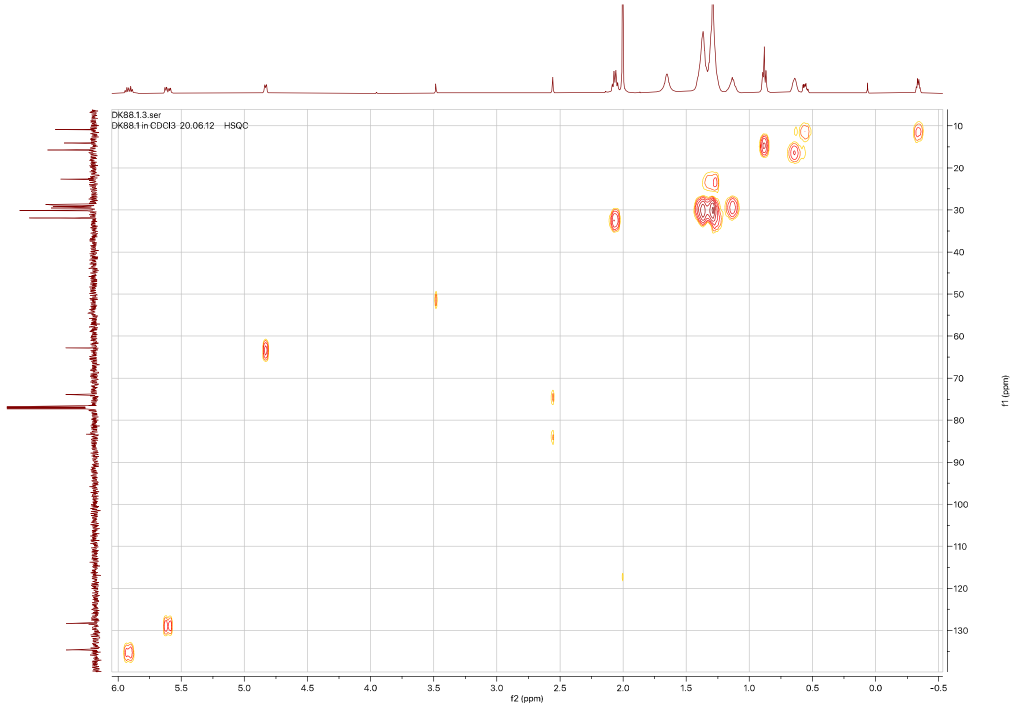


Figure S47. HMBC spectrum of (3*R*,*E*)-12-*cis*-(2-hexylcyclopropyl)dodec-4-en-1-yn-3-ol (**8**) in $CDCl_3$.

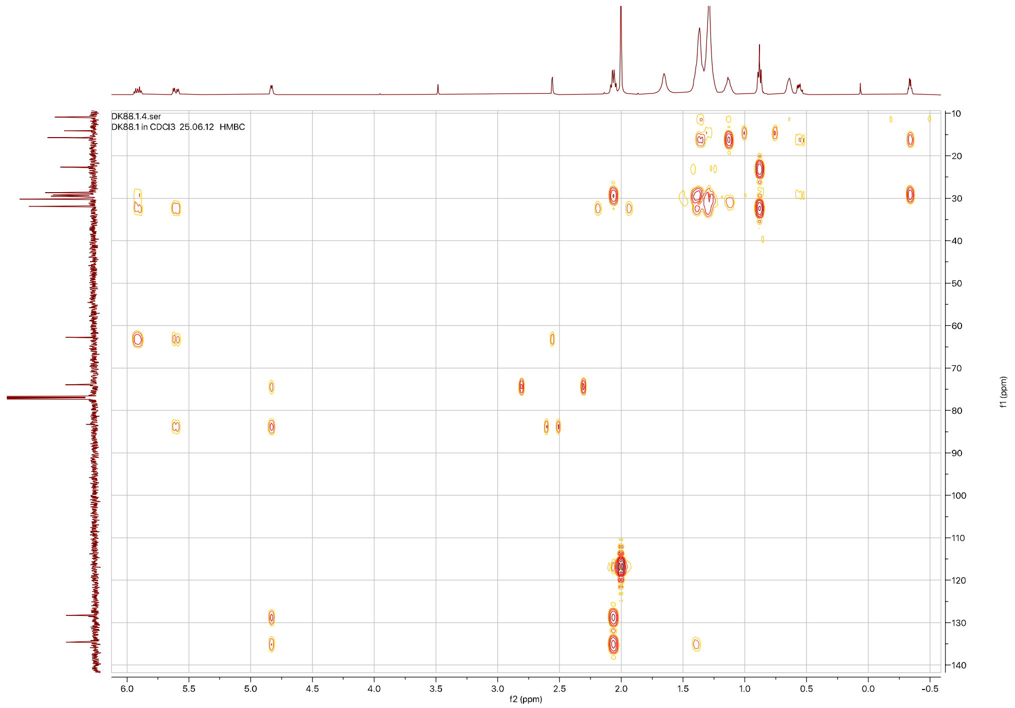

Figure S48. COSY spectrum of (3*R*,*E*)-12-*cis*-(2-hexylcyclopropyl)dodec-4-en-1-yn-3-ol (**8**) in $CDCl_3$.

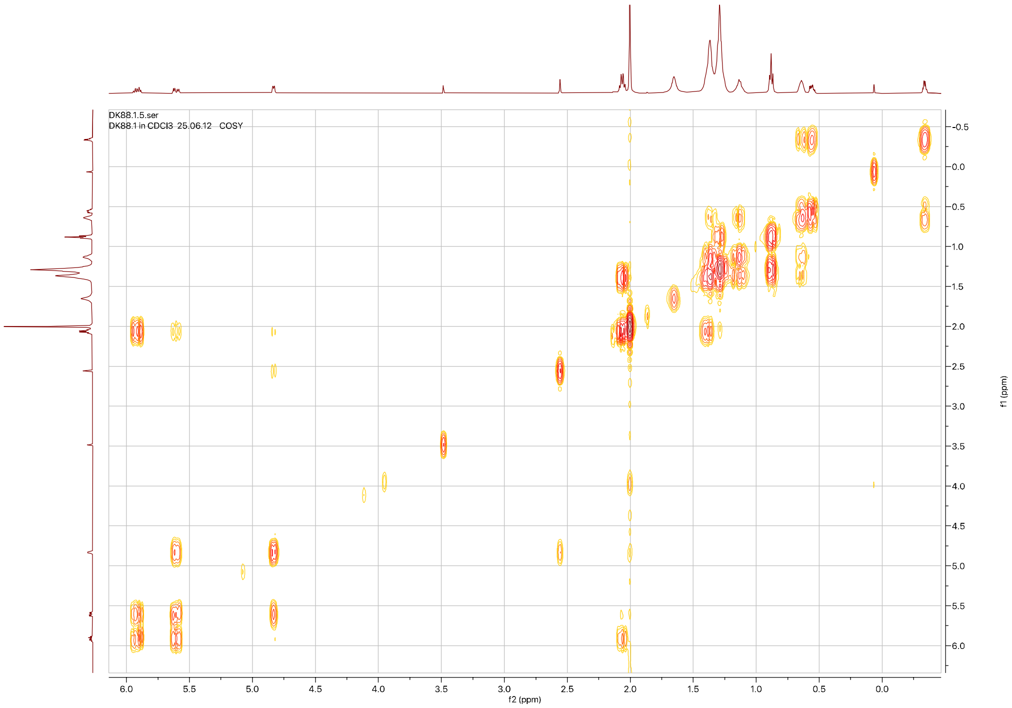

Figure S49. DEPT spectrum of (3*R*,*E*)-12-*cis*-(2-hexylcyclopropyl)dodec-4-en-1-yn-3-ol (**8**) in $CDCl_3$.

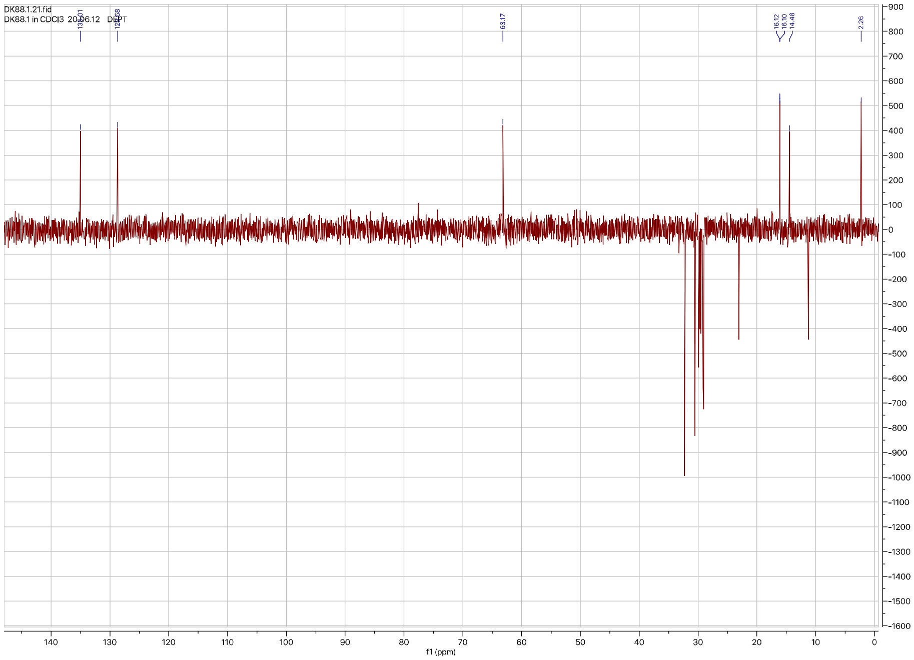

Table S8. NMR data of (3*R*,*E*)-12-*cis*-(2-hexylcyclopropyl)dodec-4-en-1-yn-3-ol (**8**) in $CDCl_3$.[a]

| Position | $\delta_C$, mult.[b] | $\delta_H$, mult. *J* (Hz) | LR H-C Correlations[c] |
|---|---|---|---|
| 1 | 73.9[d] CH | 2.56 d (2.1) | 3 |
| 2 | 83.3[e] qC | - | 1, 3, 4 |
| 3 | 62.8 CH | 4.83 brd (5.7) | 1, 4 |
| 4 | 128.3 CH | 5.60 dd (15.2, 5.7) | 3, 6 |
| 5 | 134.6 CH | 5.91 dt (15.2, 7.0) | 3, 4, 6, 7 |
| 6 | 31.9 $CH_2$ | 2.06 q (7.0) | 4, 5, 7, 8 |
| 7 | 28.8 $CH_2$ | 1.36 m | 5, 6, 8 |
| 8-10 | ~29.6[f] 3 × $CH_2$ | 1.24 – 1.32 brm | |
| 11 | 30.2 $CH_2$ | 1.35 m | 10, 12a, 12b, |
| 12a | 28.7 $CH_2$ | 1.35 m | 11, 13a, 13b |
| b | | 1.13 m | |
| 13 | 15.7 CH | 0.64 m | 11, 12a, 12b, 14, 15a, 15b, 21a, 21b |
| 14 | 15.7 CH | 0.64 m | 11, 12a, 12b, 14, 15a, 15b, 21a, 21b |
| 15a | 28.7 $CH_2$ | 1.35 m | 13a, 13b, 14, 16, 21a, 21b |
| b | | 1.13 m | |
| 16 | 30.2 $CH_2$ | 1.35 m | 15a, 15b, 17 |
| 17 | ~29.6[f] $CH_2$ | 1.24 – 1.32 brm | |
| 18 | 31.9 $CH_2$ | 1.26 m | 17, 19 |
| 19 | 22.7 $CH_2$ | 1.31 m | 18, 20 |
| 20 | 14.1 $CH_3$ | 0.88 t (6.8) | 18, 19 |
| 21a | 10.9 $CH_2$ | -0.55 td (8.2, 4.0) | 11, 12a, 12b, 13, 14, 15a, 15b, 16 |
| b | | -0.33 td (4.9, 4.0) | |

[a]500.13 MHz for $^1H$ and 125.76 MHz for $^{13}C$; [b]Multiplicity and assignment from HSQC experiment; [c]Determined from HMBC experiment; [d] $^1J$ = 251.3 Hz; [e] $^2J$ = 48.9 Hz; [f]Exact $^{13}C$ chemical shifts 29.17, 29.33, 29.49, 29.55 ppm.

Figure S50. Measured and calculated $^{13}C$ NMR data of (3*R*,*E*)-12-*cis*-(2-hexylcyclopropyl)dodec-4-en-1-yn-3-ol (**8**)

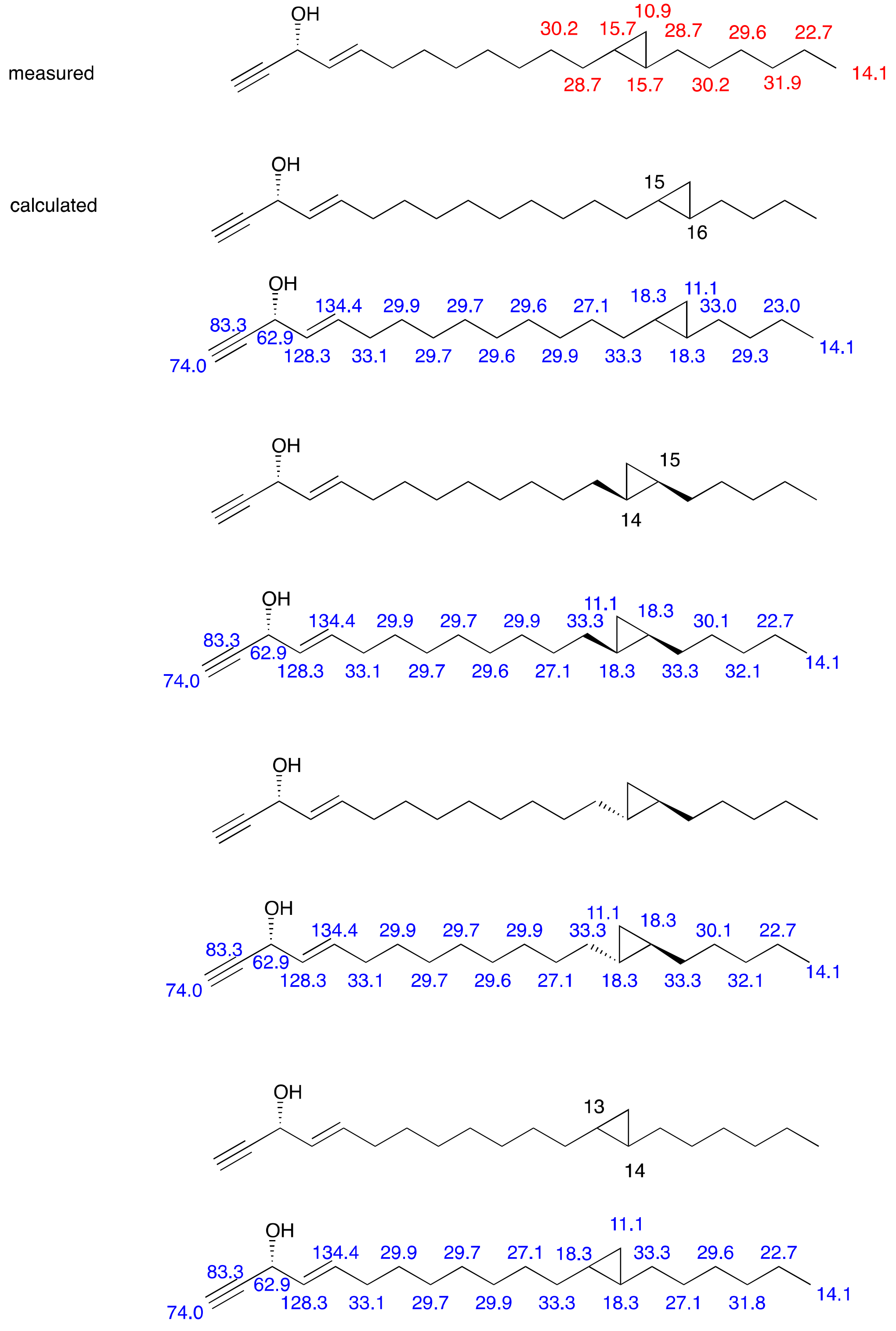

Figure S51. EIMS of (3*R*,*E*)-12-*cis*-(2-hexylcyclopropyl)dodec-4-en-1-yn-3-ol (**8**).

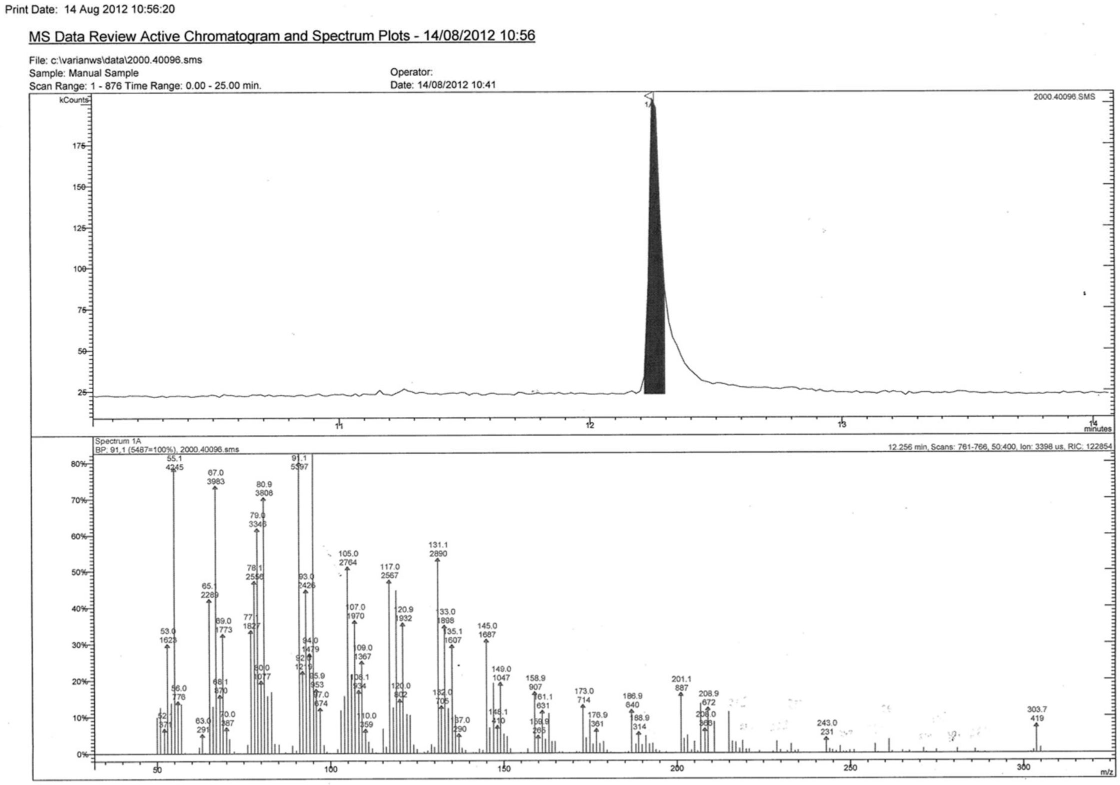


Figure S52. HRMS of (3*R*,*E*)-12-*cis*-(2-hexylcyclopropyl)dodec-4-en-1-yn-3-ol (**8**).

## Compound Spectrum SmartFormula Report

**Analysis Info**

| | | | |
|---|---|---|---|
| Analysis Name | D:\Data\Client\CLI_9279_9.d | Acquisition Date | 5/17/2015 11:06:37 AM |
| Method | CLI_9279.m | Operator | Larisa Panz |
| Sample Name | (10) DK 38.1 | Instrument | maXis impact 282001.00128 |
| Comment | | | |

**Acquisition Parameter**

| | | | | | |
|---|---|---|---|---|---|
| Source Type | APCI | Ion Polarity | Positive | Set Nebulizer | 4.0 Bar |
| Focus | Active | Set Capillary | 4000 V | Set Dry Heater | 200 °C |
| Scan Begin | 50 m/z | Set End Plate Offset | -500 V | Set Dry Gas | 2.0 l/min |
| Scan End | 1000 m/z | Set Charging Voltage | 2000 V | Set Divert Valve | Source |
| | | Set Corona | 4000 nA | Set APCI Heater | 450 °C |

**+MS, 8.0-8.1min #477-484**

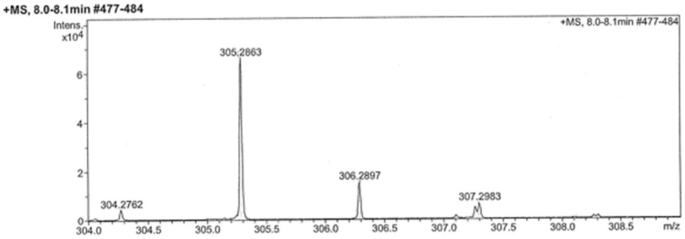


| Meas. m/z | # | Ion Formula | m/z | err [ppm] | mSigma | # mSigma | Score | rdb | e⁻ Conf | N-Rule | \|err\| [mDa] | \|err\| [mDa] |
|---|---|---|---|---|---|---|---|---|---|---|---|---|
| 305.2863 | 1 | C21H37O | 305.2839 | -7.8 | 42.7 | 1 | 100.00 | 3.5 | even | ok | 2.4 | 2.4 |

Figure S53a. Proposed fragmentation pattern of the EIMS parent ion of (3*R*,*E*)-12-*cis*-(2-hexylcyclopropyl)dodec-4-en-1-yn-3-ol (**8**).

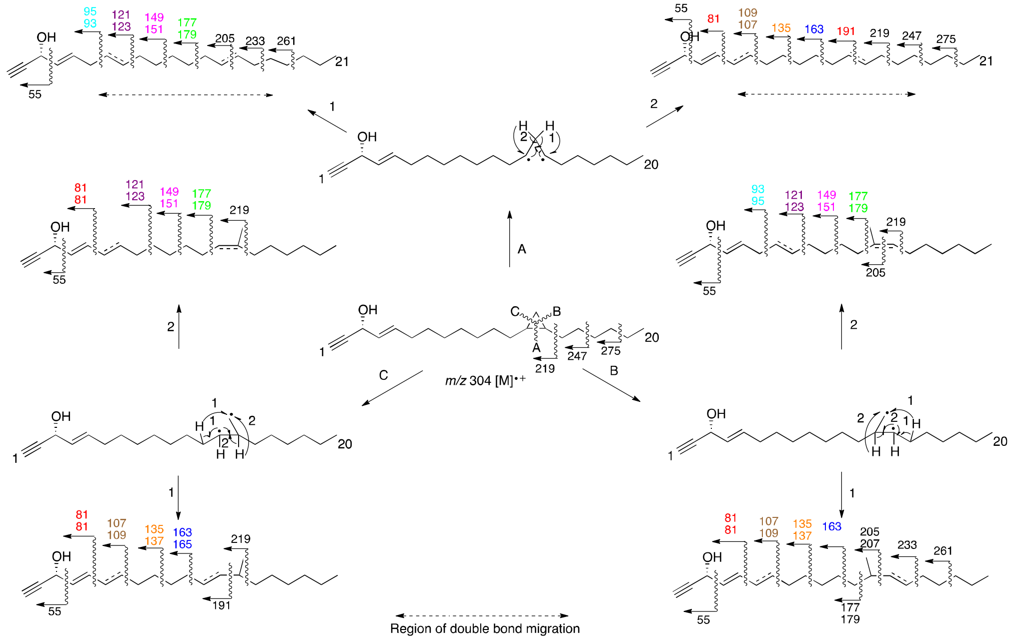


Figure S53b. Proposed fragmentation pattern of the EIMS water elimination product ion of (3*R*,*E*)-12-*cis*-(2-hexylcyclopropyl)dodec-4-en-1-yn-3-ol (**8**).

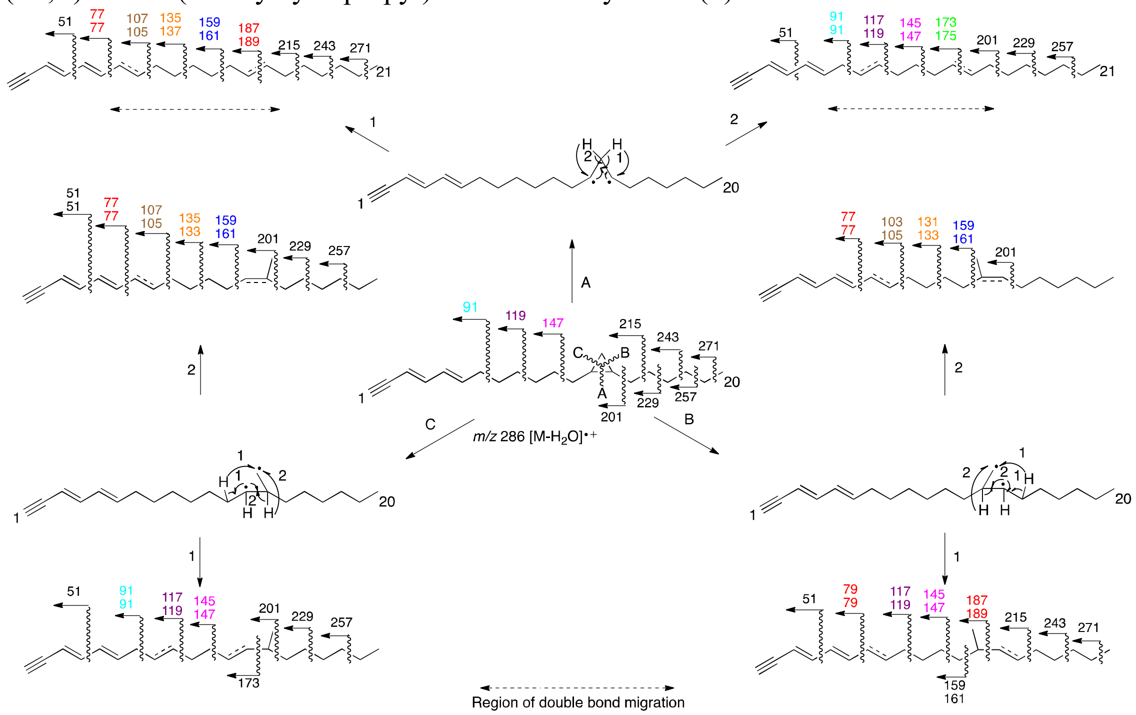

Figure S54. $^{1}$H NMR spectrum of (3*R*)-13-methylhenicos-(4*E*)-en-1-yn-3-ol (**9**) in $CDCl_3$

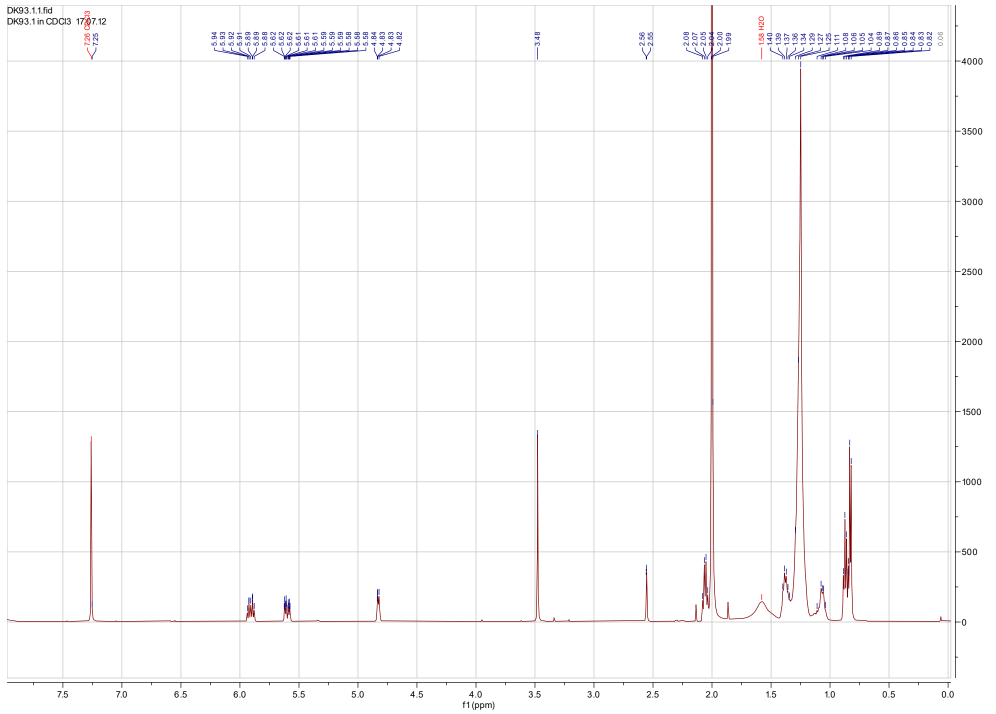


Figure S55. $^{13}$C NMR spectrum of (3*R*)-13-methylhenicos-(4*E*)-en-1-yn-3-ol (**9**) in $CDCl_3$

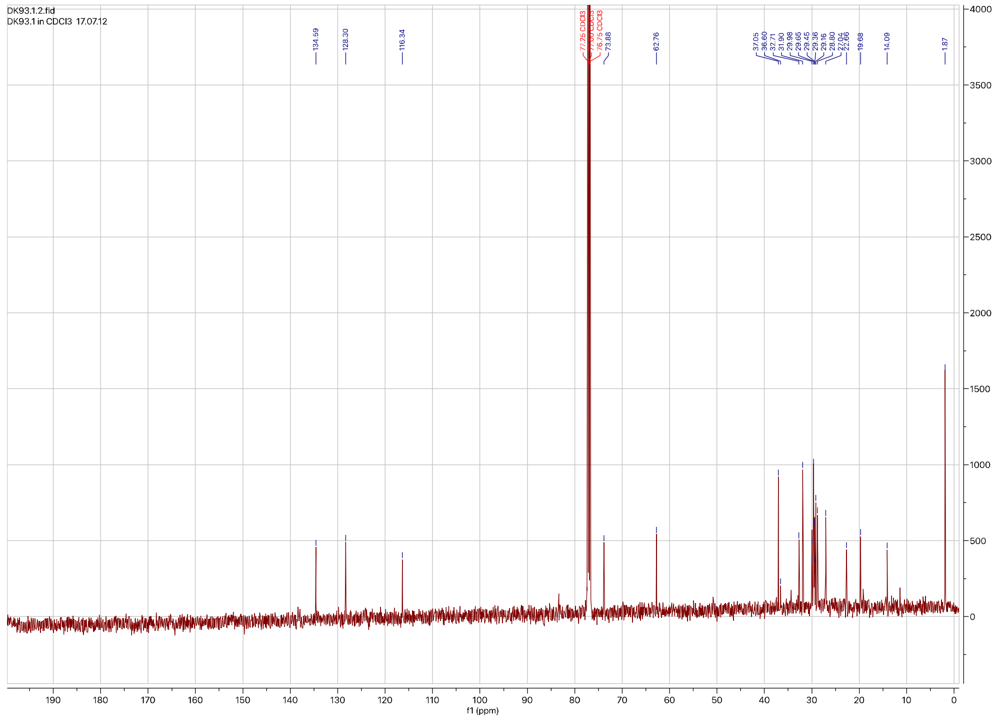

Table S9. NMR data of (3*R*)-13-methylhenicos-(4*E*)-en-1-yn-3-ol (**9**) in $CDCl_3$.[a]

| Position | $\delta_C$, mult.[b] | $\delta_H$, mult. *J* (Hz) | LR H-C Correlations[c] |
|---|---|---|---|
| 1 | 73.9[d] CH | 2.55 d (2.5) | 3 |
| 2 | 83.3[e] qC | - | 1, 3, 4 |
| 3 | 62.8 CH | 4.82 brd (6.0) | 1, 4, 5 |
| 4 | 128.3 CH | 5.60 dd (16.0, 6.0) | 6 |
| 5 | 134.6 CH | 5.91 dt (16.0, 7.5) | 3, 6, 7 |
| 6 | 31.9 $CH_2$ | 2.05 q (7.5) | 4, 5, 7, 8 |
| 7 | 28.8 $CH_2$ | 1.36 m | 5, 6, 8 |
| 8-11 | ~29.6[f] 4 × $CH_2$ | 1.21 – 1.30 brm | |
| 12 | 27.0 $CH_2$ | 1.23 m | 11, 13a, 13b |
| 13a | 37.1 $CH_2$ | 1.25 m | 12, 14, 15a, 15b, 21 |
| b | | 1.07 m | |
| 14 | 32.7 CH | 1.37 m | 13a, 13b, 15a, 15a, 22 |
| 15a | 37.1 $CH_2$ | 1.25 m | 13a, 13b, 14, 16, 22 |
| b | | 1.07 m | |
| 16 | 27.0 $CH_2$ | 1.23 m | 15a, 15b, 17 |
| 17-18 | ~29.6[f] 2 × $CH_2$ | 1.21 – 1.30 brm | |
| 19 | 31.9 $CH_2$ | 1.23 m | 18, 20a, 20b, 21 |
| 20a | 22.7 $CH_2$ | 1.29 m | 19, 21 |
| b | | 1.23 m | |
| 21 | 14.1 $CH_3$ | 0.87 t (6.5) | 19a, 19b |
| 22 | 19.7 $CH_3$ | 0.83 d (6.5) | 13b, 15b |

[a]500.13 MHz for $^1$H and 125.76 MHz for $^{13}$C; [b]Multiplicity and assignment from HSQC experiment; [c]Determined from HMBC experiment; [d] $^1J$ = 250.5 Hz; [e] $^2J$ = 48.8 Hz; [f]Exact $^{13}$C chemical shifts 29.16, 29.44, 29.66 (× 3), 29.96 ppm.

Figure S56. EIMS and fragmentation pattern of (3*R*)-13-methylhenicos-(4*E*)-en-1-yn-3-ol (**9**)

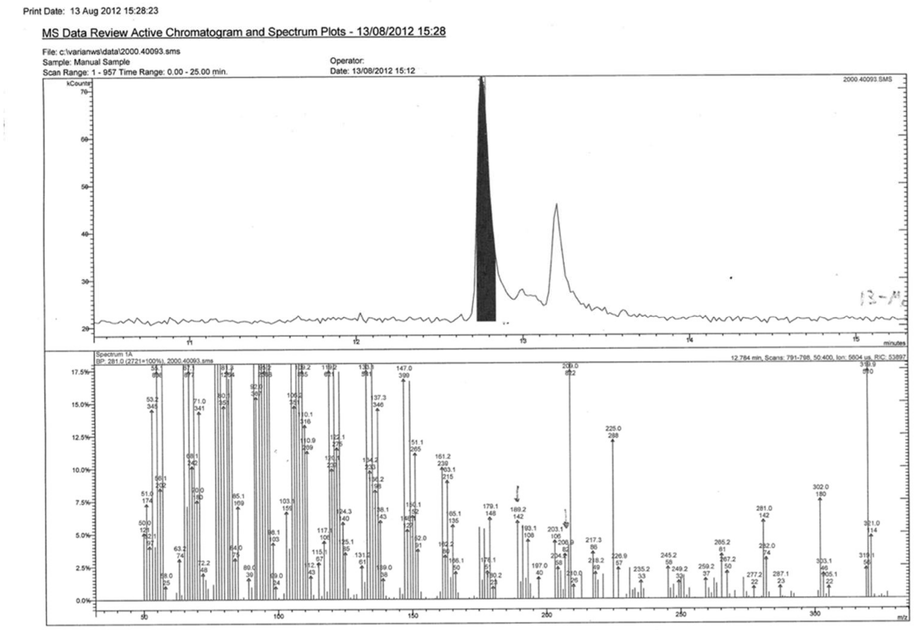


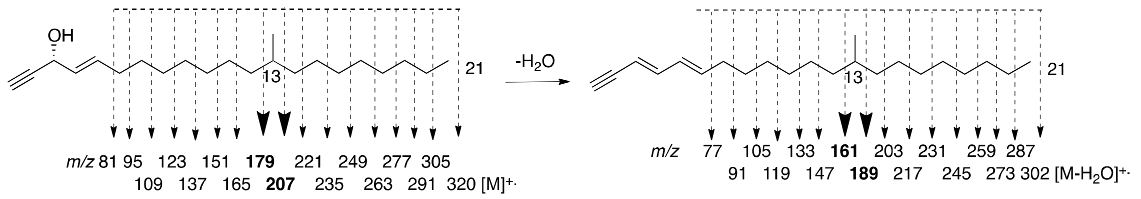


Figure S57. HRMS of (3*R*)-13-methylhenicos-(4*E*)-en-1-yn-3-ol (**9**)

**Compound Spectrum SmartFormula Report**

**Analysis Info**

| | | | |
|---|---|---|---|
| Analysis Name | D:\Data\Client\CLI_9279_7.d | Acquisition Date | 5/17/2015 10:36:18 AM |
| Method | CLI_9279.m | Operator | Larisa Panz |
| Sample Name | DK_93-1(13) | Instrument | maXis impact 282001.00128 |
| Comment | | | |

**Acquisition Parameter**

| | | | | | |
|---|---|---|---|---|---|
| Source Type | APCI | Ion Polarity | Positive | Set Nebulizer | 4.0 Bar |
| Focus | Active | Set Capillary | 4000 V | Set Dry Heater | 200 °C |
| Scan Begin | 50 m/z | Set End Plate Offset | -500 V | Set Dry Gas | 2.0 l/min |
| Scan End | 1000 m/z | Set Charging Voltage | 2000 V | Set Divert Valve | Source |
| | | Set Corona | 4000 nA | Set APCI Heater | 450 °C |

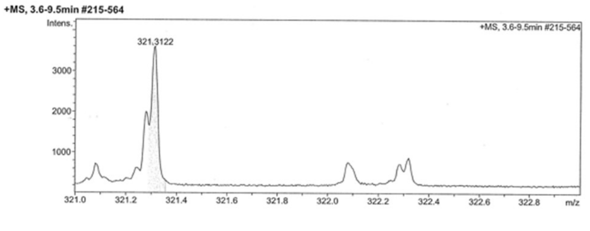


| Meas. m/z | # | Ion Formula | m/z | err [ppm] | mSigma | # mSigma | Score | rdb | e⁻ Conf | N-Rule | \|err\| [mDa] |
|---|---|---|---|---|---|---|---|---|---|---|---|
| 321.3122 | 1 | C22H41O | 321.3152 | 9.2 | 589.5 | 1 | 100.00 | 2.5 | even | ok | 2.9 |

Figure S58. $^{1}$H NMR spectrum of docos-(4$E$,15$Z$)-dien-1-yn-3-one (**10**) in $CDCl_3$

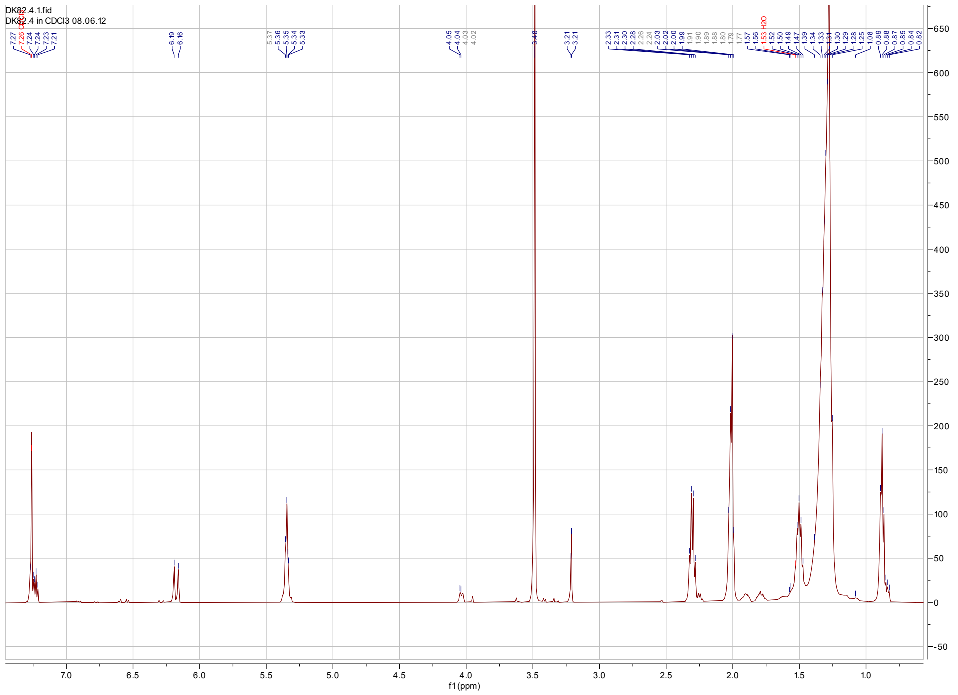


Figure S59. $^{13}$C NMR spectrum of docos-(4$E$,15$Z$)-dien-1-yn-3-one (**10**) in $CDCl_3$

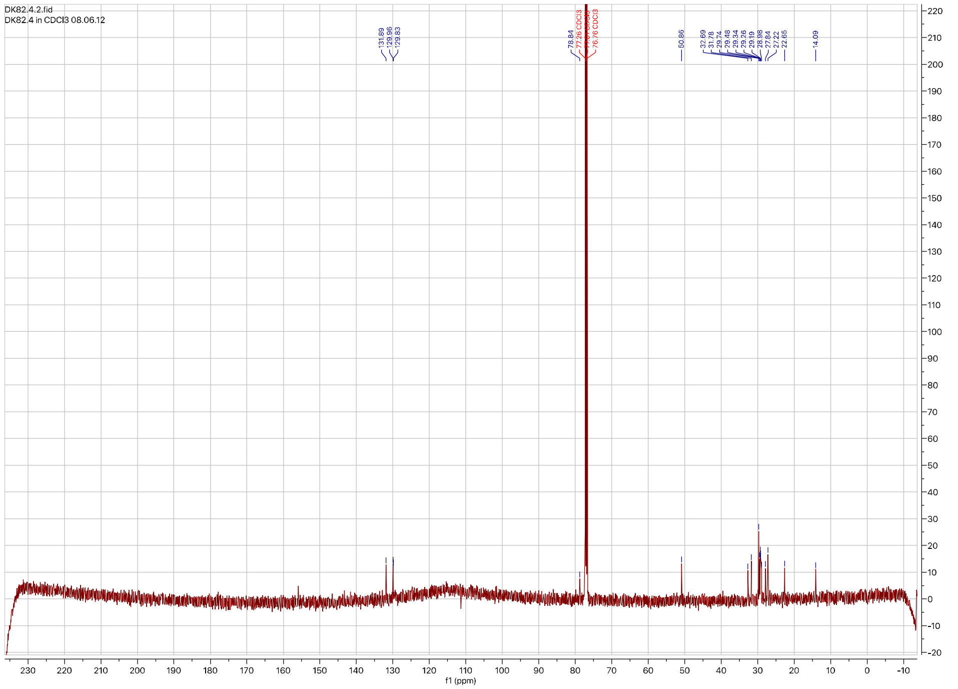

Figure S60. HSQC spectrum of docos-(4$E$,15$Z$)-dien-1-yn-3-one (**10**) in $CDCl_3$

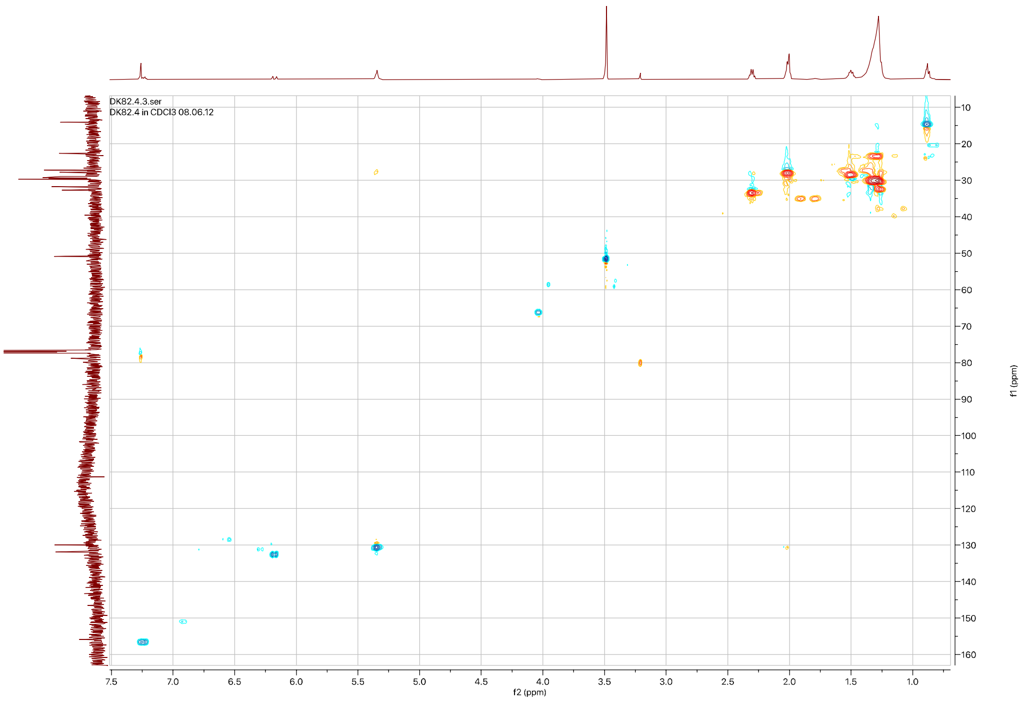


Figure S61. HMBC spectrum of docos-(4$E$,15$Z$)-dien-1-yn-3-one (**10**) in $CDCl_3$

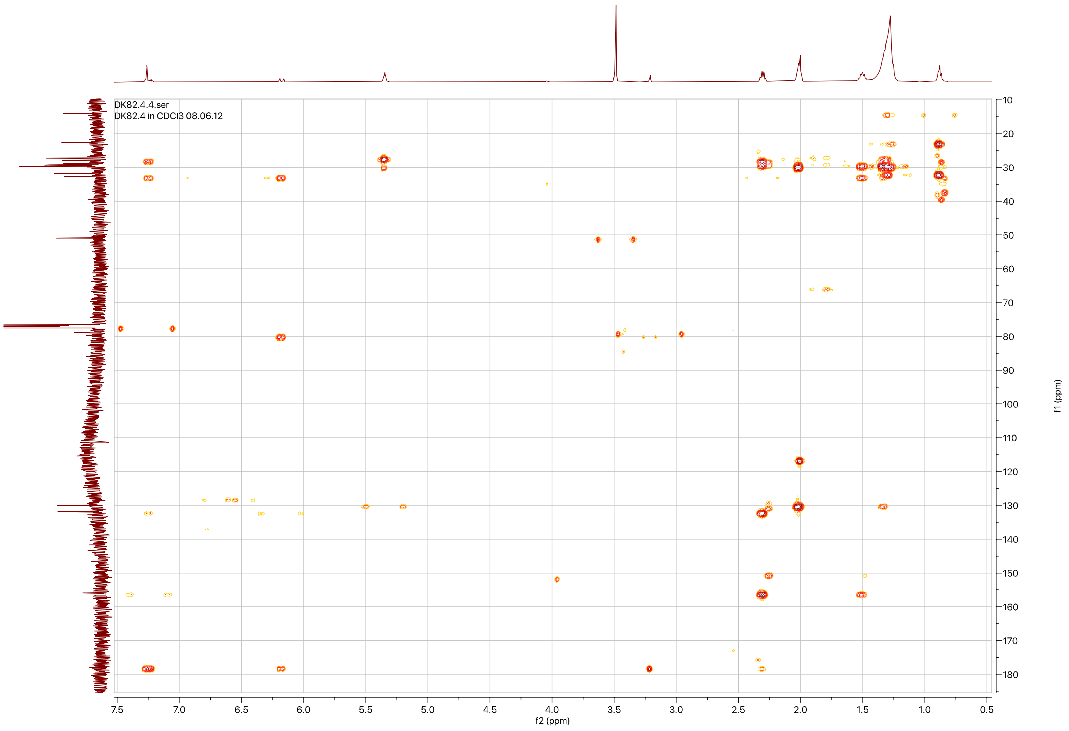

Figure S62. COSY spectrum of docos-(4*E*,15*Z*)-dien-1-yn-3-one (**10**) in $CDCl_3$

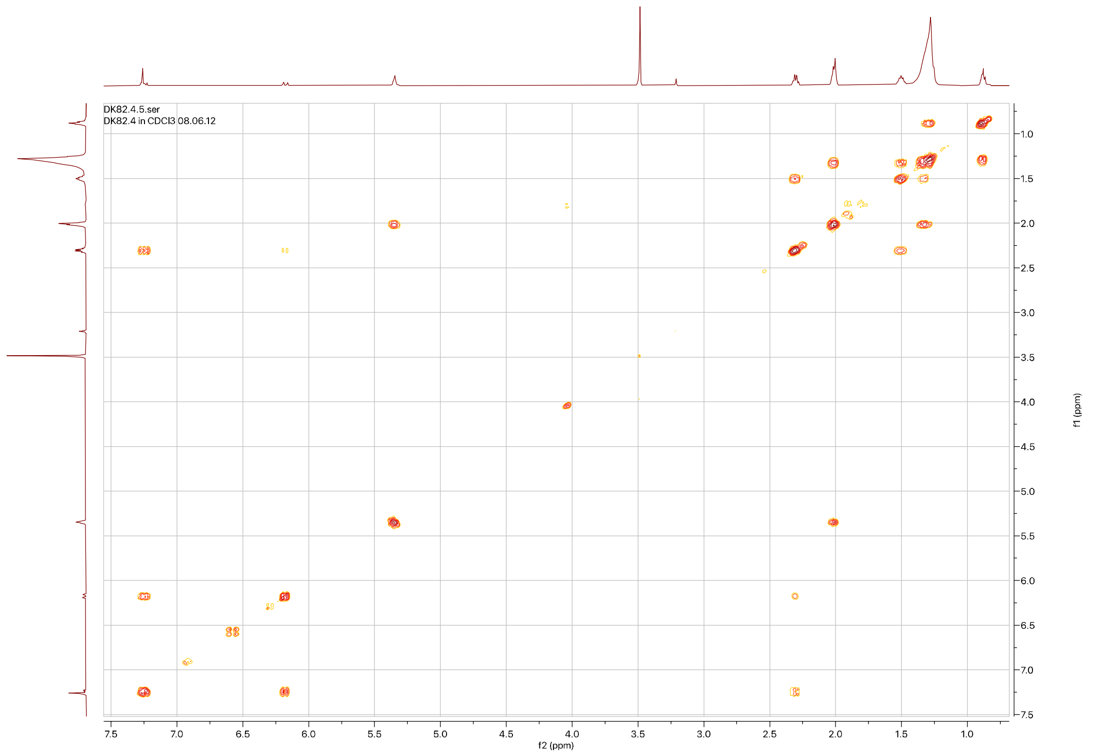

Table S10. NMR data of docos-(4*E*,15*Z*)-dien-1-yn-3-one (**10**) in $CDCl_3$.[a]

| Position | $\delta_C$, mult.[b] | $\delta_H$, mult. *J* (Hz) | LR H-C Correlations[c] |
|---|---|---|---|
| 1 | 78.8[d] CH | 3.20 s | - |
| 2 | 79.8[e] qC | - | 1, 4 |
| 3 | 177.9 qC | - | 1, 4, 5 |
| 4 | 131.9 CH | 6.17 d (16.0) | 6 |
| 5 | 155.9 CH | 7.23 dt (16.0, 7.0) | 6, 7 |
| 6 | 32.7 $CH_2$ | 2.30 q (7.0) | 4, 5, 7, 8 |
| 7 | 27.8 $CH_2$ | 1.50 tt (7.0, 7.0) | 5, 6, 8 |
| 8 | 29.2 $CH_2$ | 1.30 m | |
| 9-13 | ~29.6[f] 5 × $CH_2$ | 1.23 – 1.35 brm | |
| 14 | 27.2 $CH_2$ | 2.02 m | 13, 15 |
| 15 | 129.8 CH | 5.35 m | 13, 14 |
| 16 | 129.9 CH | 5.35 m | 17, 18 |
| 17 | 27.2 $CH_2$ | 2.02 m | 16, 18 |
| 18-19 | 29.6[f] 2 × $CH_2$ | 1.23 – 1.35 brm | |
| 20 | 31.8 $CH_2$ | 1.27 m | 19, 21a, 21b, 22 |
| 21a | 22.6 $CH_2$ | 1.31 m | 20, 22 |
| b | | 1.27 m | |
| 22 | 14.1 $CH_3$ | 0.88 t (6.7) | 20, 21a, 21b |

[a]500.13 MHz for $^1$H and 125.76 MHz for $^{13}$C; [b]Multiplicity and assignment from HSQC experiment; [c]Determined from HMBC experiment; [d] $^1J$ = 254.6 Hz; [e] $^2J$ = 47.6 Hz; [f]Exact $^{13}$C chemical shifts 29.25, 29.33, 29.47, 29.73 (× 4) ppm.

Figure S63. EIMS and fragmentation pattern of docos-(4*E*,15*Z*)-dien-1-yn-3-one (**10**)

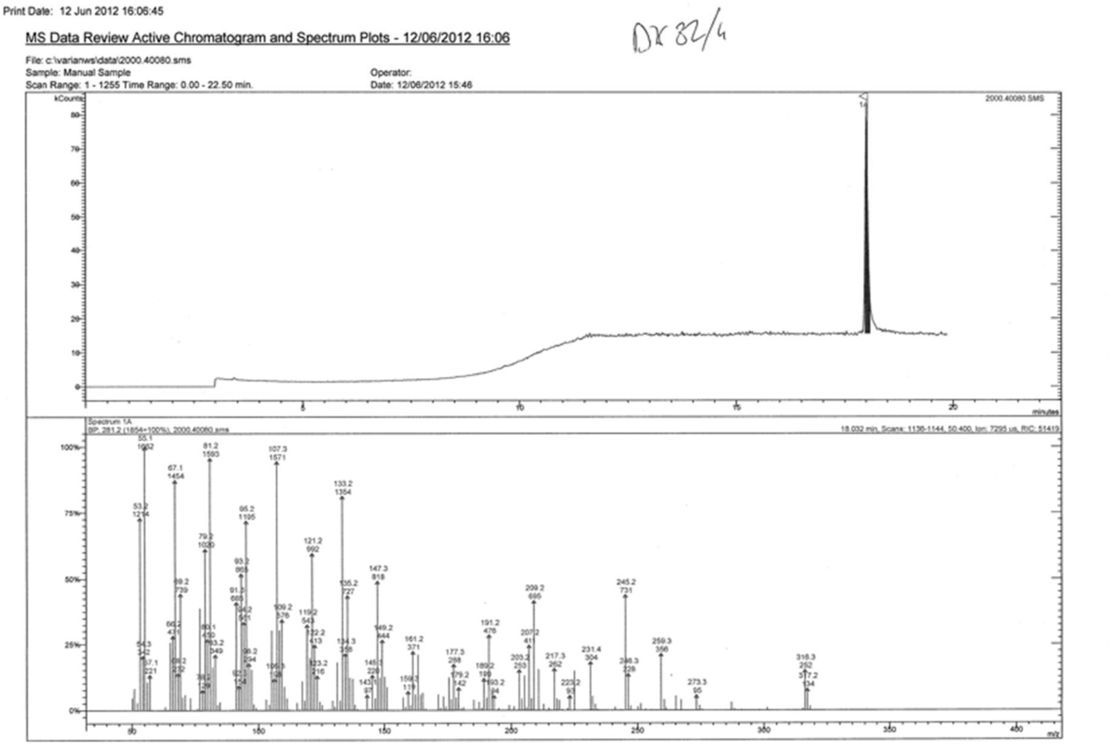

Figure S64. LCMS chromatogram and mass spectrum of the periodate-permanganate oxidation products of docos-(4*E*,15*Z*)-dien-1-yn-3-one (**10**). 2 Minutes gradient from 100% solvent A (95% $H_2O$, 5% ACN, 0.1% FA) to 100% of solvent B (99.9% ACN, 0.1% FA) and then isocratic elution in 100% of solvent B for 2 more minutes.

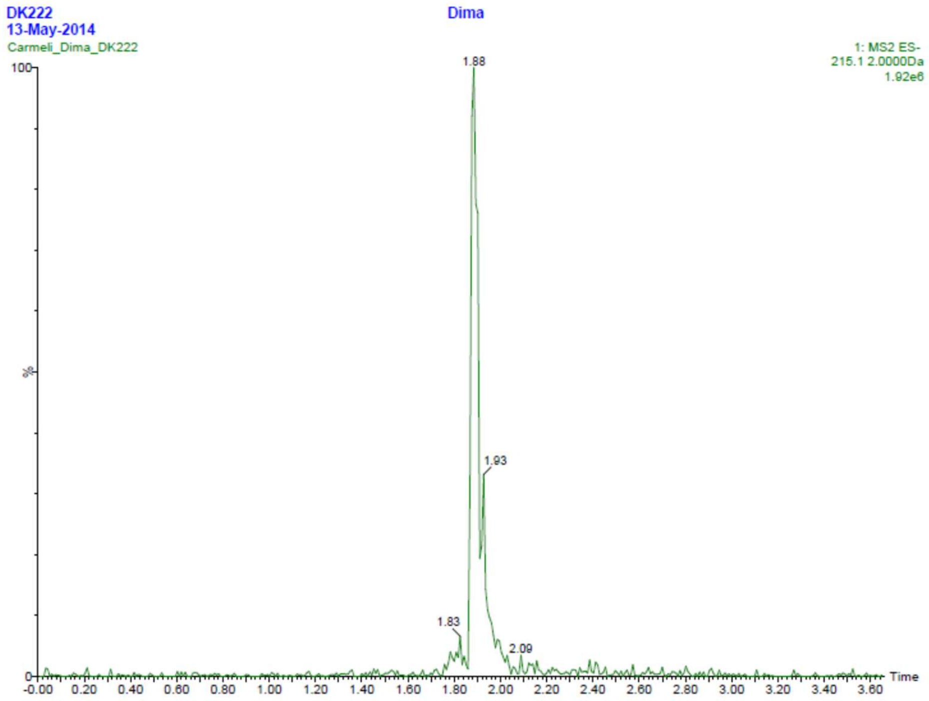


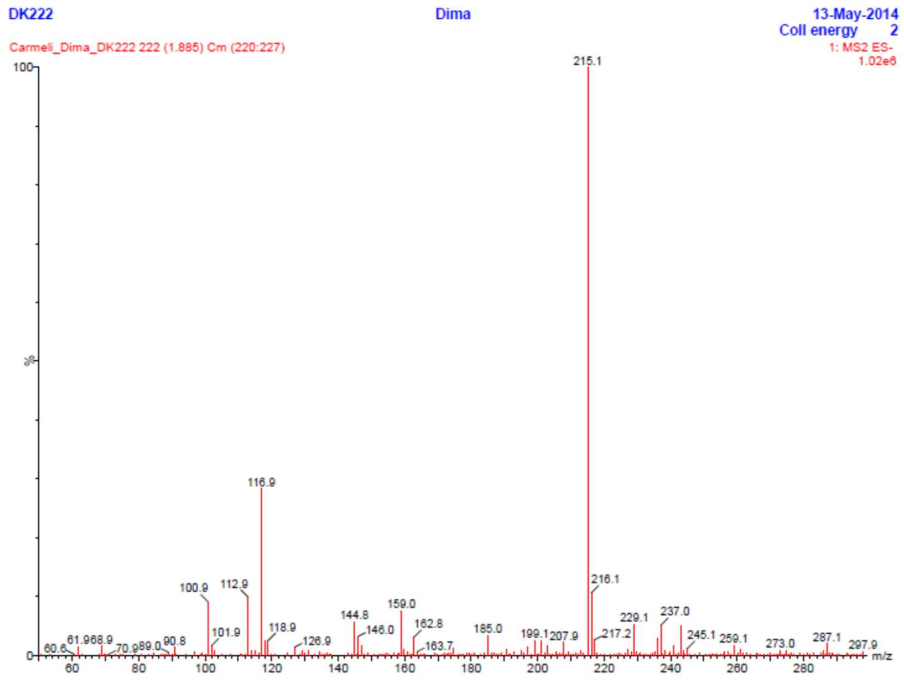

Figure S65. $^{1}$H NMR spectrum of (3*R*)-docos-(15*Z*)- en-1-yn-3-ol (**11**) in $CDCl_3$

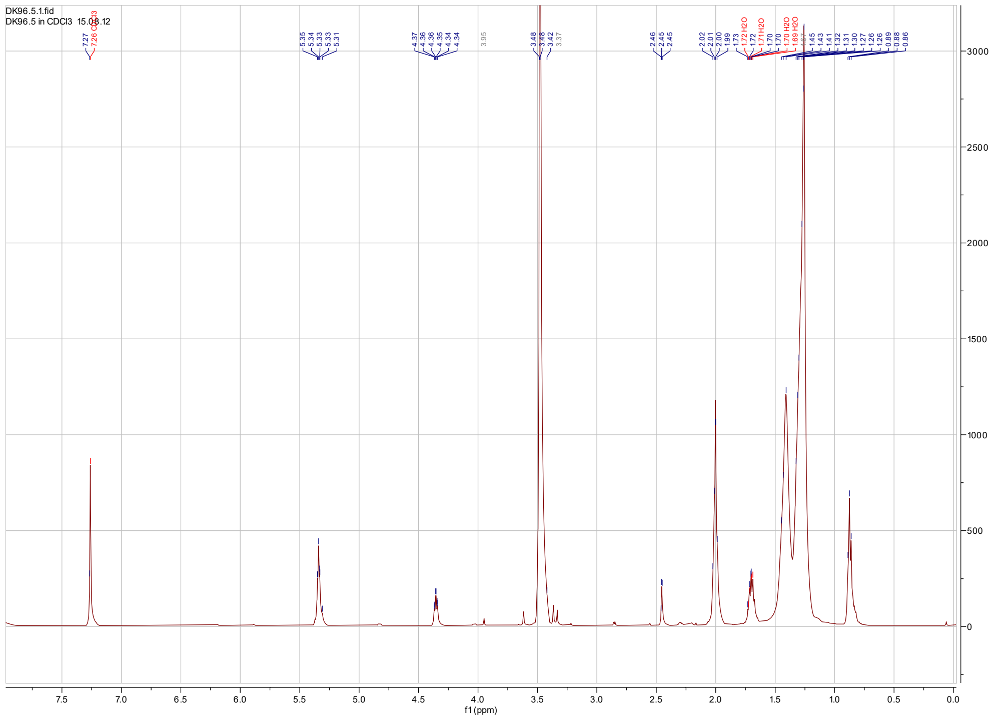

Figure S66. $^{13}$C NMR spectrum of (3*R*)-docos-(15*Z*)- en-1-yn-3-ol (**11**) in $CDCl_3$

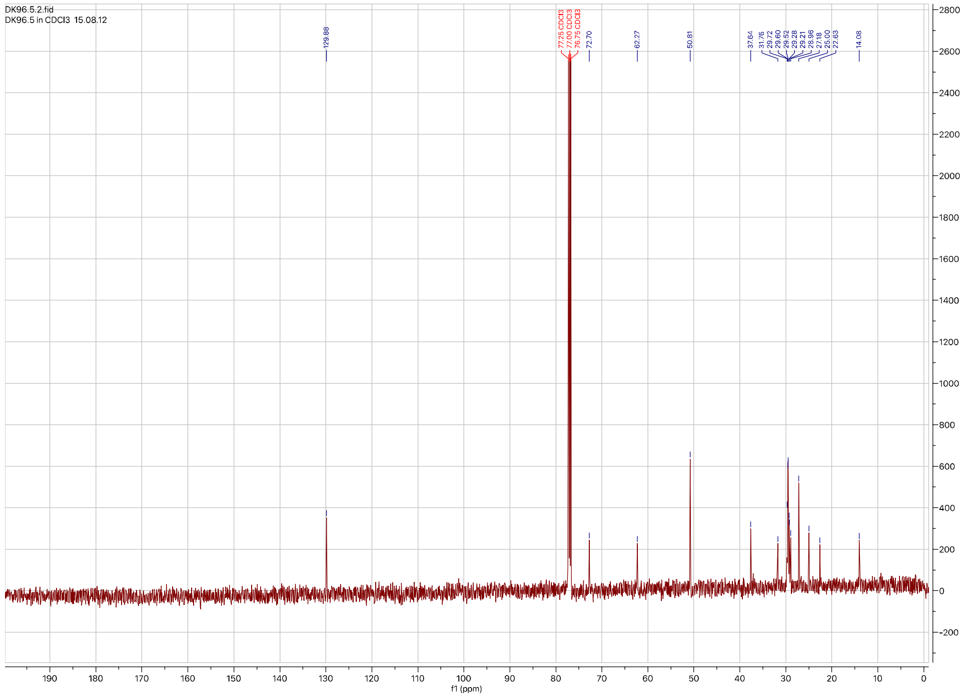

Table S11. NMR data of (3*R*)-docos-(15*Z*)- en-1-yn-3-ol (**11**) in $CDCl_3$.[a]

| Position | $\delta_C$, mult.[b] | $\delta_H$, mult. *J* (Hz) | LR H-C Correlations[c] |
|---|---|---|---|
| 1 | 72.6[d] CH | 2.45 d (2.0) | 3 |
| 2 | 85.0[e] qC | - | 1, 3, 4 |
| 3 | 62.3 CH | 4.36 td (6.5, 2.0) | 1, 4 |
| 4 | 37.6 $CH_2$ | 1.70 m | 3, 6 |
| 5 | 25.0 $CH_2$ | 1.41 m | 3, 4, 6 |
| 6 | 29.0 $CH_2$ | 1.26 m | 4, 5 |
| 7-13 | ~29.6[f] 7 × $CH_2$ | 1.22 – 1.31 brm | |
| 14 | 27.2 $CH_2$ | 2.01 m | 13, 15 |
| 15 | 129.9 CH | 5.34 t (4.5) | 13, 14 |
| 16 | 129.9 CH | 5.34 t (4.5) | 17, 18 |
| 17 | 27.2 $CH_2$ | 2.01 m | 16, 18 |
| 18-19 | 29.6[f] 2 × $CH_2$ | 1.22 – 1.31 brm | |
| 20 | 31.8 $CH_2$ | 1.25 m | 19, 21a, 21b, 22 |
| 21a | 22.6 $CH_2$ | 1.33 m | 20, 22 |
| b | | 1.28 m | |
| 22 | 14.1 $CH_3$ | 0.87 t (7.0) | 20, 21a, 21b |

[a]500.13 MHz for $^1H$ and 125.76 MHz for $^{13}C$; [b]Multiplicity and assignment from HSQC experiment; [c]Determined from HMBC experiment; [d] $^1J$ = 251.0 Hz; [e] $^2J$ = 46.0 Hz; [f]Exact $^{13}C$ chemical shifts 29.21, 29.49, 29.52 (× 3), 29.60 (× 2), 29.72, 29.74 ppm.

Figure S67. EIMS of (3*R*)-docos-(15*Z*)- en-1-yn-3-ol (**11**)

Print Date: 14 Aug 2012 11:41:52

Dk 96/5

MS Data Review Active Chromatogram and Spectrum Plots - 14/08/2012 11:41

File: c:\varianws\data\2000.40098.sms
Sample: Manual Sample
Scan Range: 1 - 1124 Time Range: 0.00 - 25.00 min.

Operator:
Date: 14/08/2012 11:22

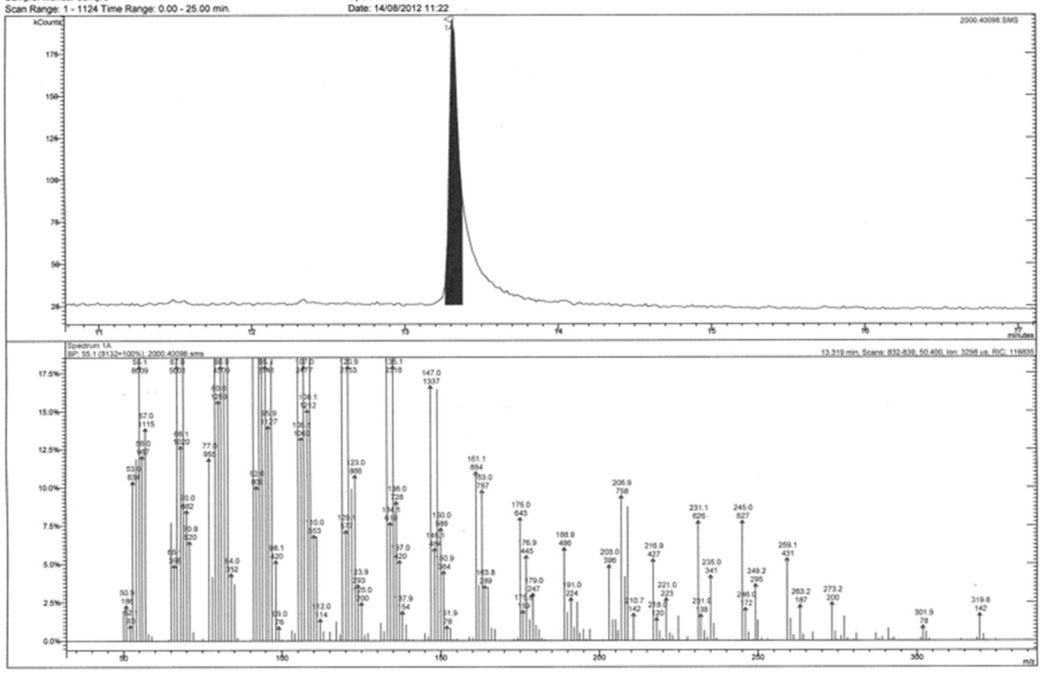

Figure S68. $^{1}$H NMR spectrum of (3*R*)-tetracos-(4*E*,15*Z*)-dien-1-yn-3-ol (**12**) in $CDCl_3$

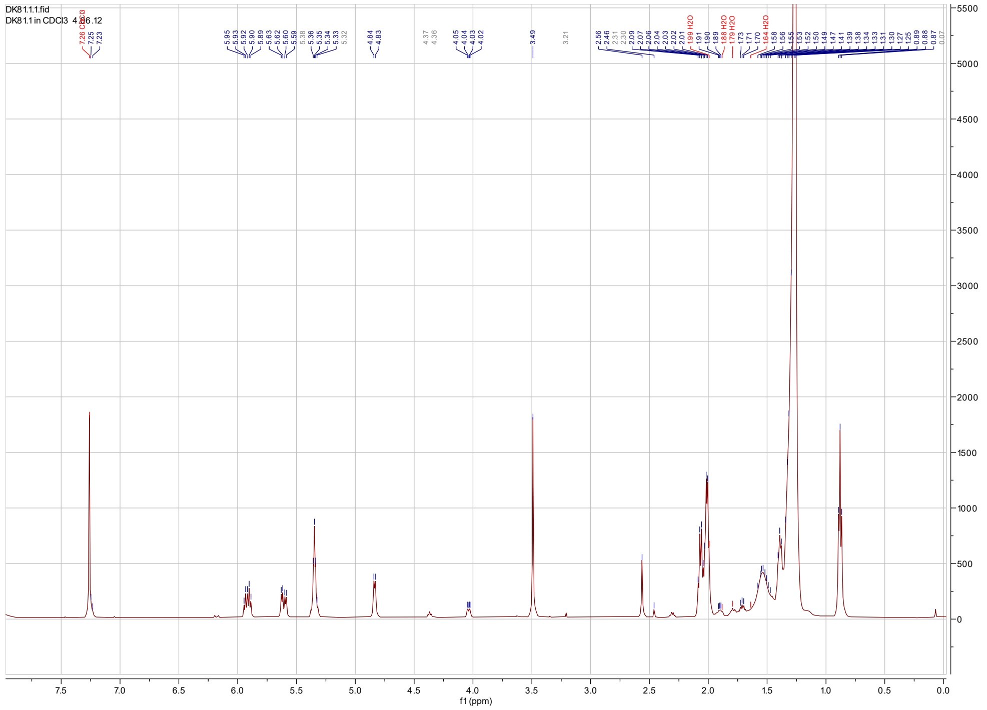


Figure S69. $^{13}$C NMR spectrum of (3*R*)-tetracos-(4*E*,15*Z*)-dien-1-yn-3-ol (**12**) in $CDCl_3$

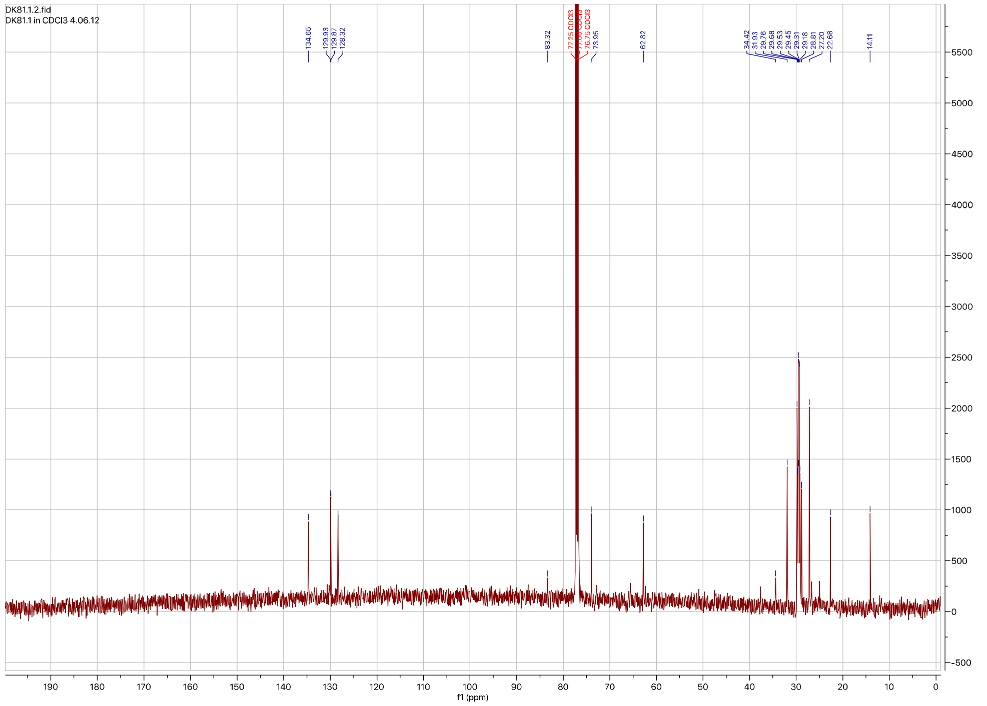

Figure S70. HSQC spectrum of (3*R*)-tetracos-(4*E*,15*Z*)-dien-1-yn-3-ol (**12**) in $CDCl_3$

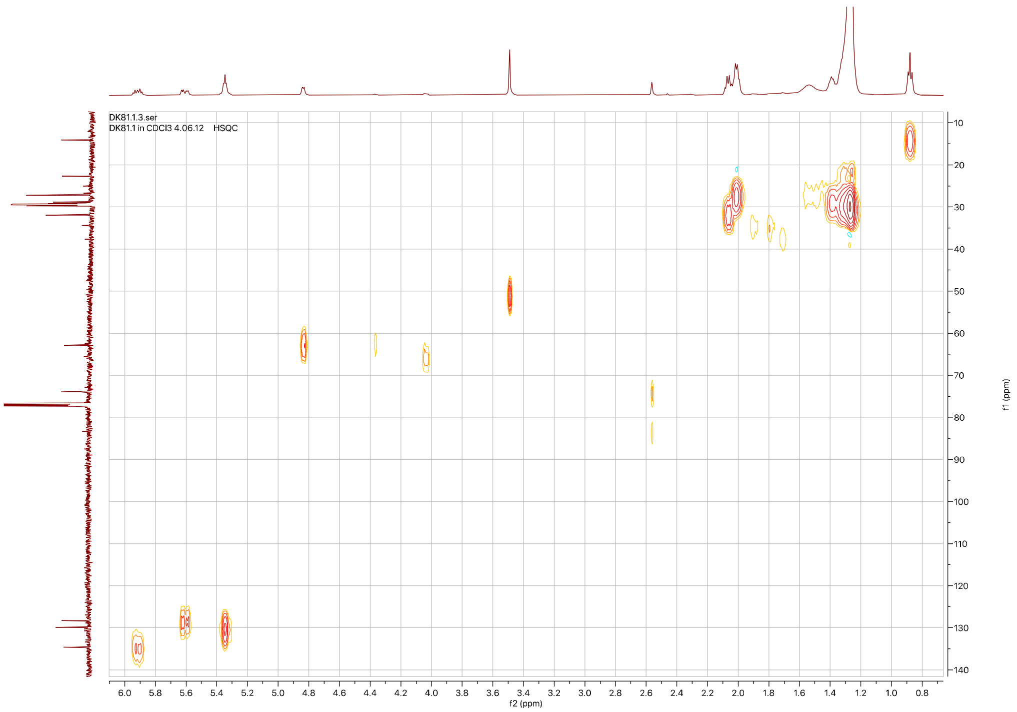


Figure S71. HMBC spectrum of (3*R*)-tetracos-(4*E*,15*Z*)-dien-1-yn-3-ol (**12**) in $CDCl_3$

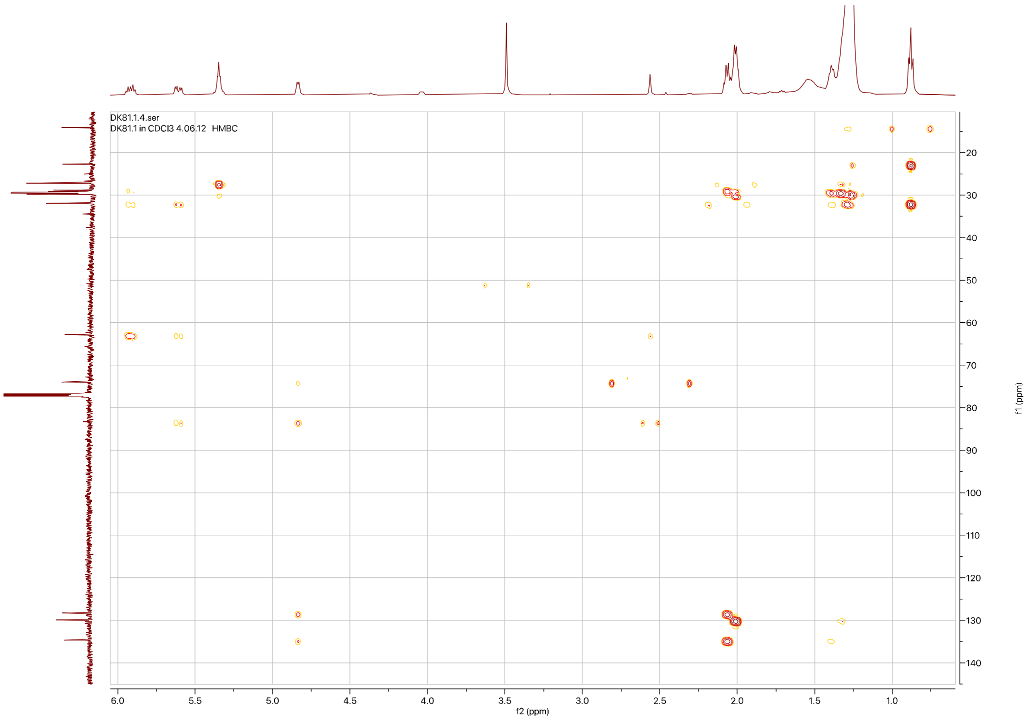

Figure S72. COSY spectrum of (3*R*)-tetracos-(4*E*,15*Z*)-dien-1-yn-3-ol (**12**) in $CDCl_3$

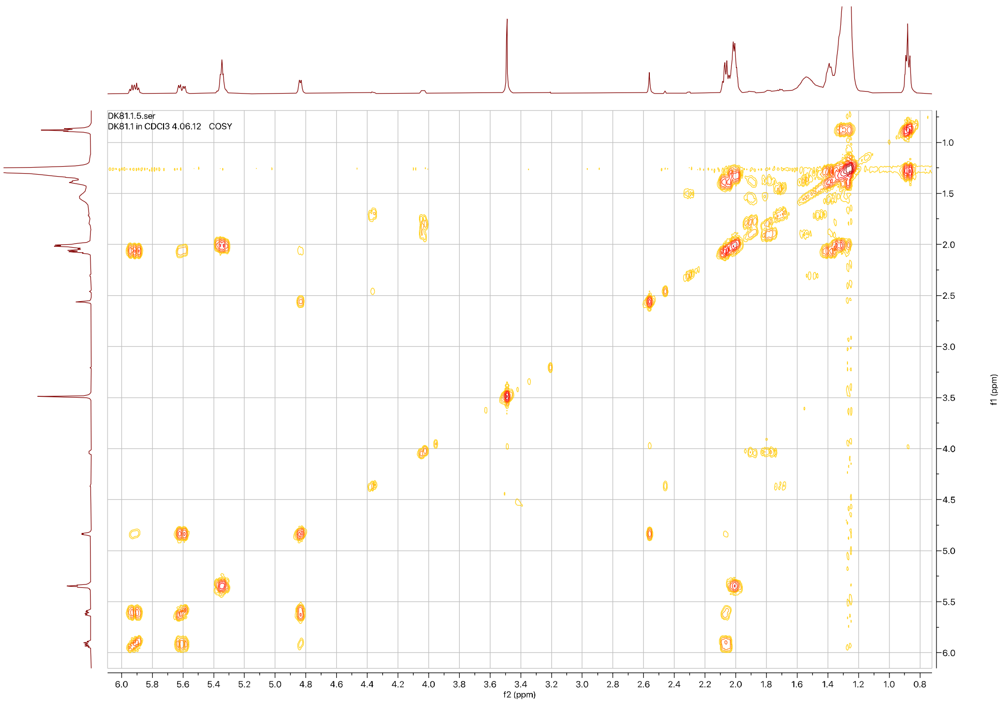


Figure S73. DEPT spectrum of (3*R*)-tetracos-(4*E*,15*Z*)-dien-1-yn-3-ol (**12**) in $CDCl_3$

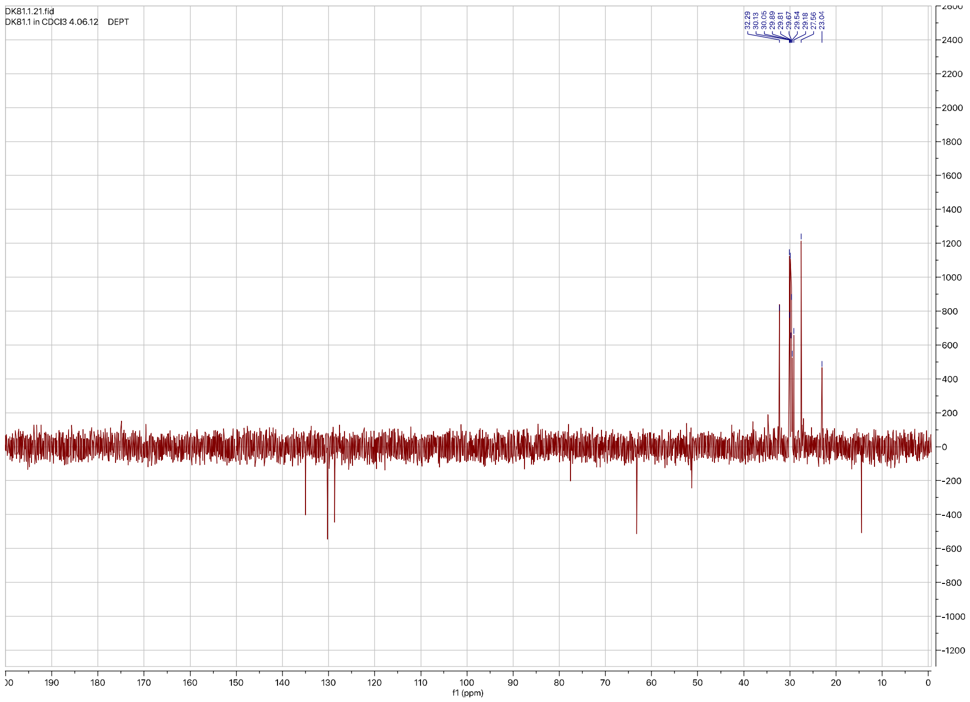

Table S12. NMR data of (3*R*)-tetracos-(4*E*,15*Z*)-dien-1-yn-3-ol (**12**) in $CDCl_3$.[a]

| Position | $\delta_C$, mult.[b] | $\delta_H$, mult. *J* (Hz) | LR H-C Correlations[c] |
|---|---|---|---|
| 1 | 73.9[d] CH | 2.56 brs | 3 |
| 2 | 83.4[e] qC | - | 1, 3, 4 |
| 3 | 62.8 CH | 4.83 brd (6.0) | 1, 4, 5 |
| 4 | 128.3 CH | 5.60 dd (15.3, 6.0) | 3, 6 |
| 5 | 134.6 CH | 5.91 dt (15.3, 7.1) | 3, 6, 7 |
| 6 | 31.9 $CH_2$ | 2.06 q (7.1) | 4, 5, 7, 8 |
| 7 | 28.8 $CH_2$ | 1.38 m | 5, 6, 8 |
| 8-13 | ~29.6[f] 6 × $CH_2$ | 1.22 – 1.32 brm | |
| 14 | 27.2 $CH_2$ | 2.01 m | 13, 15 |
| 15 | 129.9 CH | 5.34 t (5.0) | 13, 14 |
| 16 | 129.9 CH | 5.34 t (5.0) | 17, 18 |
| 17 | 27.2 $CH_2$ | 2.01 m | 16, 18 |
| 18-21 | 29.6[f] 4 × $CH_2$ | 1.22 – 1.32 brm | |
| 22 | 31.9 $CH_2$ | 1.25 m | 21, 23a, 23b, 24 |
| 23a | 22.7 $CH_2$ | 1.32 m | 22, 24 |
| b | | 1.28 m | |
| 24 | 14.1 $CH_3$ | 0.88 t (6.7) | 22, 21a, 21b |

[a]500.13 MHz for $^1H$ and 125.76 MHz for $^{13}C$; [b]Multiplicity and assignment from HSQC experiment; [c]Determined from HMBC experiment; [d] $^1J$ = 251.5 Hz; [e] $^2J$ = 49.4 Hz; [f]Exact $^{13}C$ chemical shifts 29.18, 29.31 (× 2), 29.45, 29.52 (× 4), 29.68, 29.76 ppm.

Figure S74. EIGCMS spectrum of (3$R$)-tetracos-(4$E$,15$Z$)-dien-1-yn-3-ol (**12**)

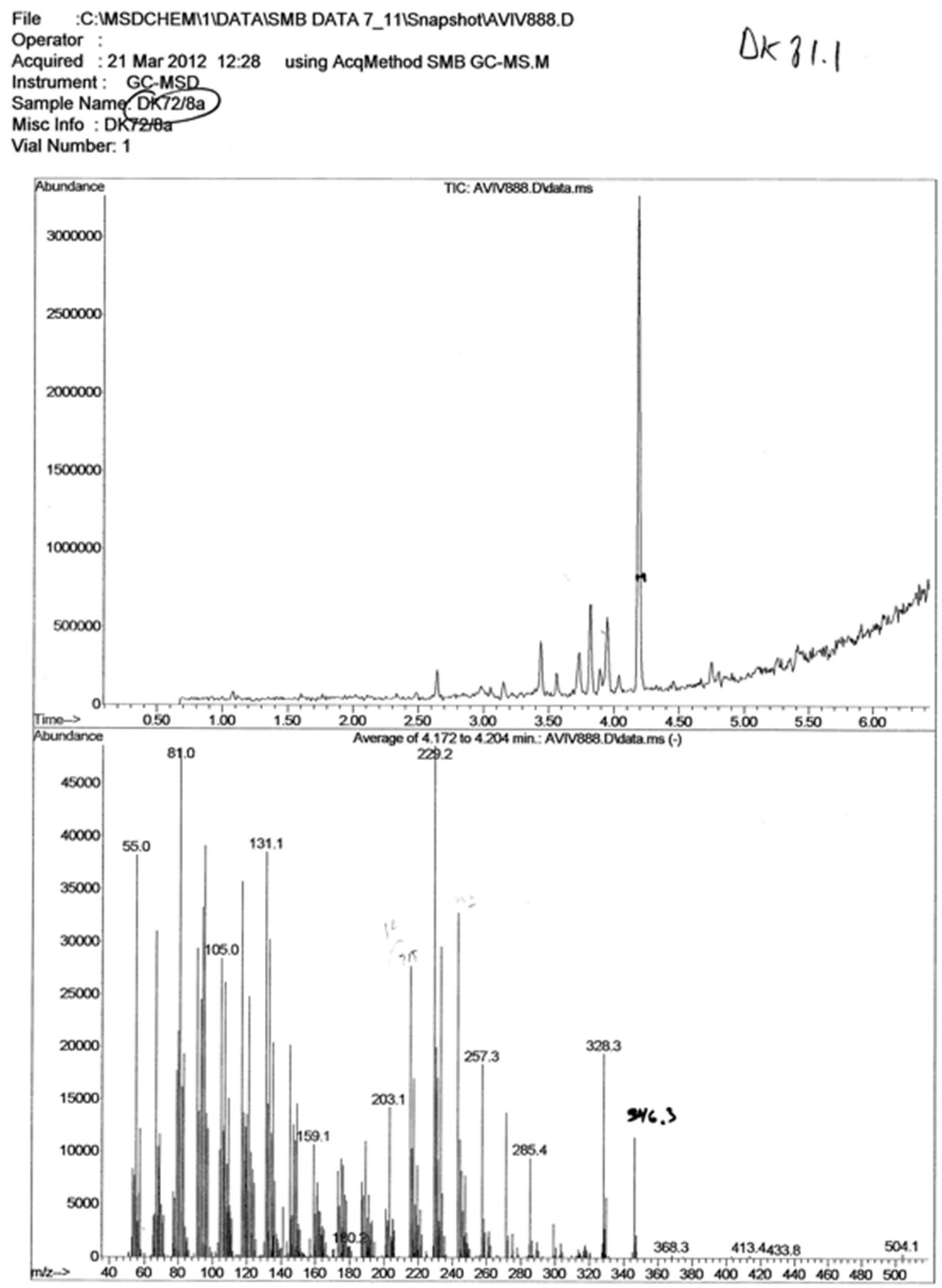

Figure S75. LCMS chromatogram and mass spectrum of the periodate-permanganate oxidation products of (3*R*)-tetracos-(4*E*,15*Z*)-dien-1-yn-3-ol (**12**). 2 Minutes gradient from 100% solvent A (95% $H_2O$, 5% ACN, 0.1% FA) to 100% of solvent B (99.9% ACN, 0.1% FA) and then isocratic elution in 100% of solvent B for 2 more minutes.

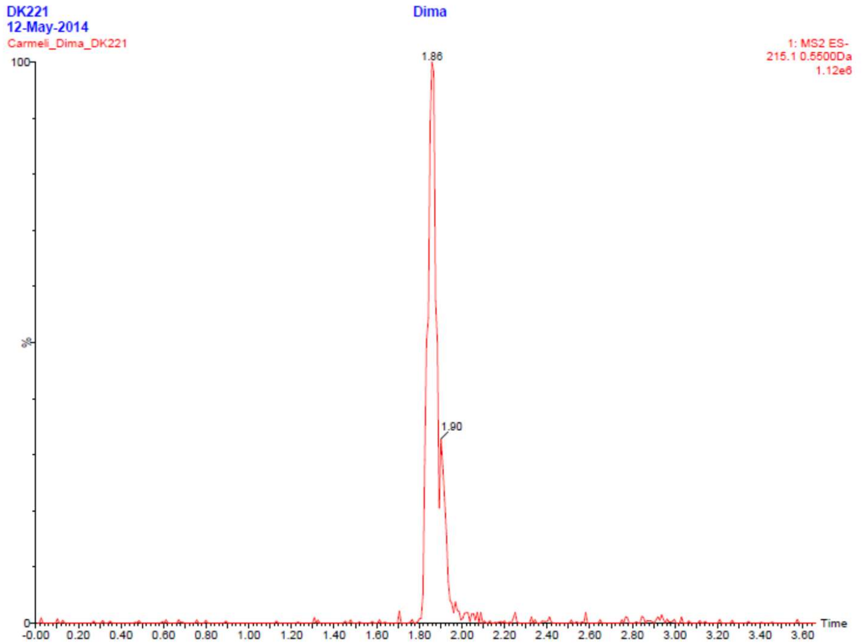


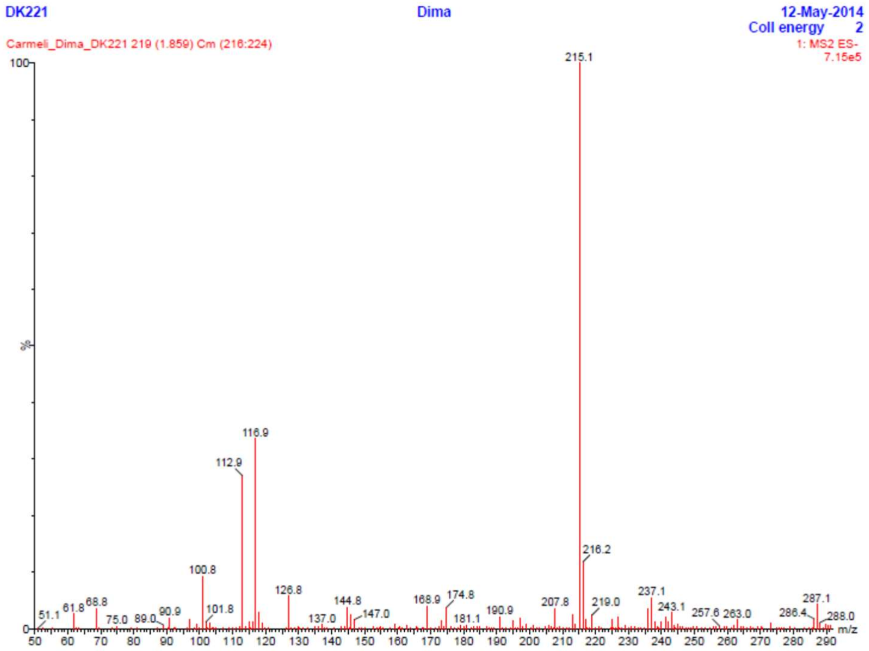

Figure S76. $^{1}$H NMR spectrum of (5*S*)-icos-(3*Z*)-en-1-yn-5-ol (**13**) in $CDCl_3$

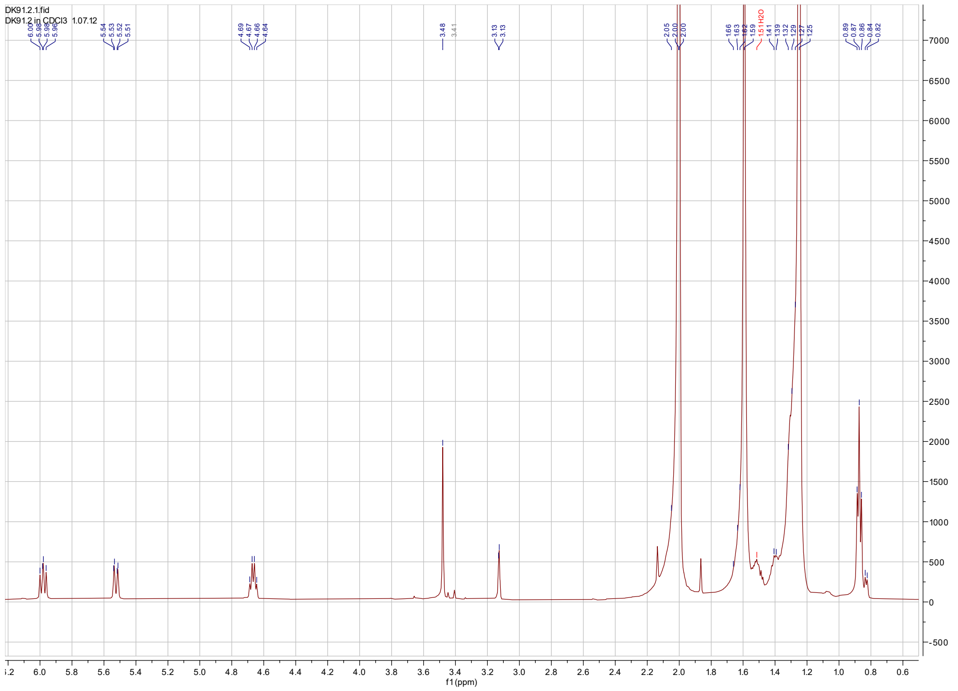

Figure S77. $^{13}$C NMR spectrum of (5*S*)-icos-(3*Z*)-en-1-yn-5-ol (**13**) in $CDCl_3$

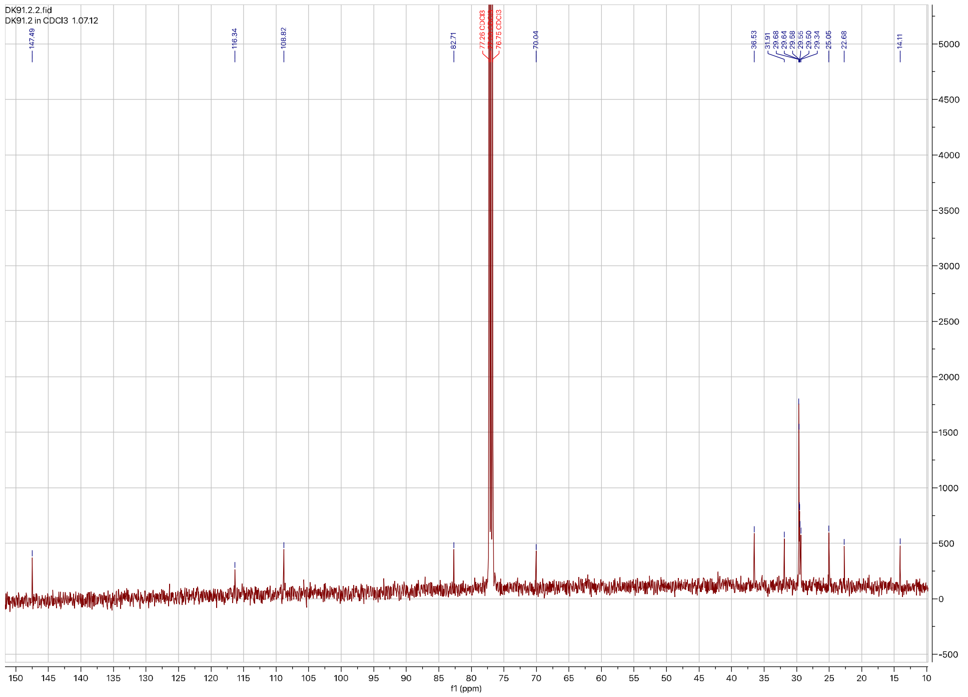

Figure S78. HSQC spectrum of (5*S*)-icos-(3*Z*)-en-1-yn-5-ol (**13**) in $CDCl_3$

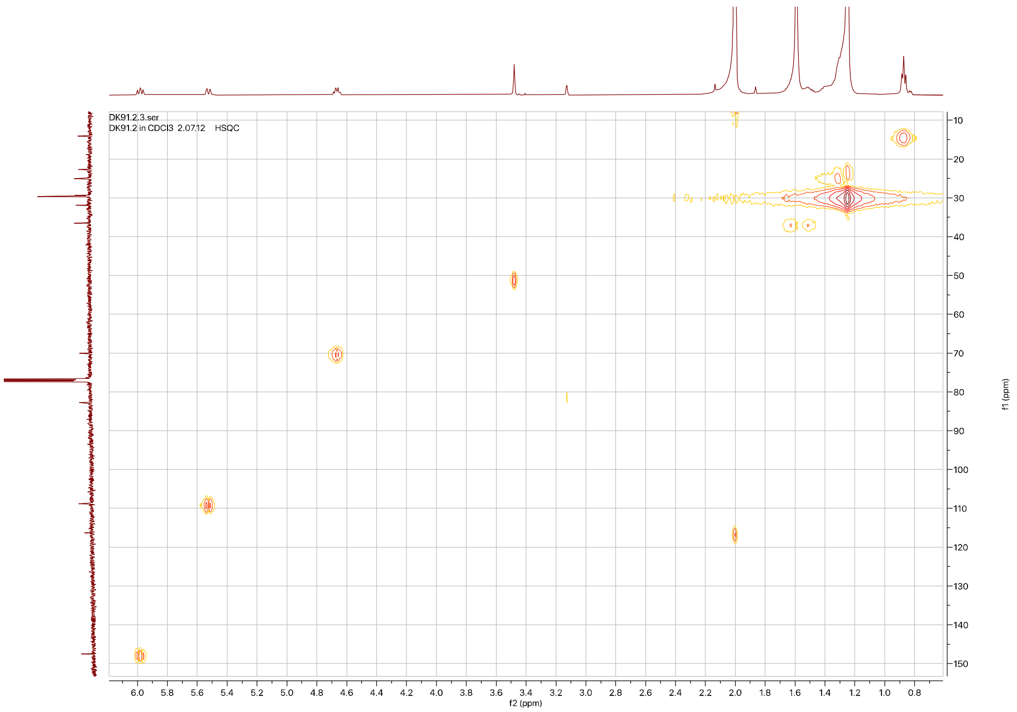


Figure S79. HMBC spectrum of (5*S*)-icos-(3*Z*)-en-1-yn-5-ol (**13**) in $CDCl_3$

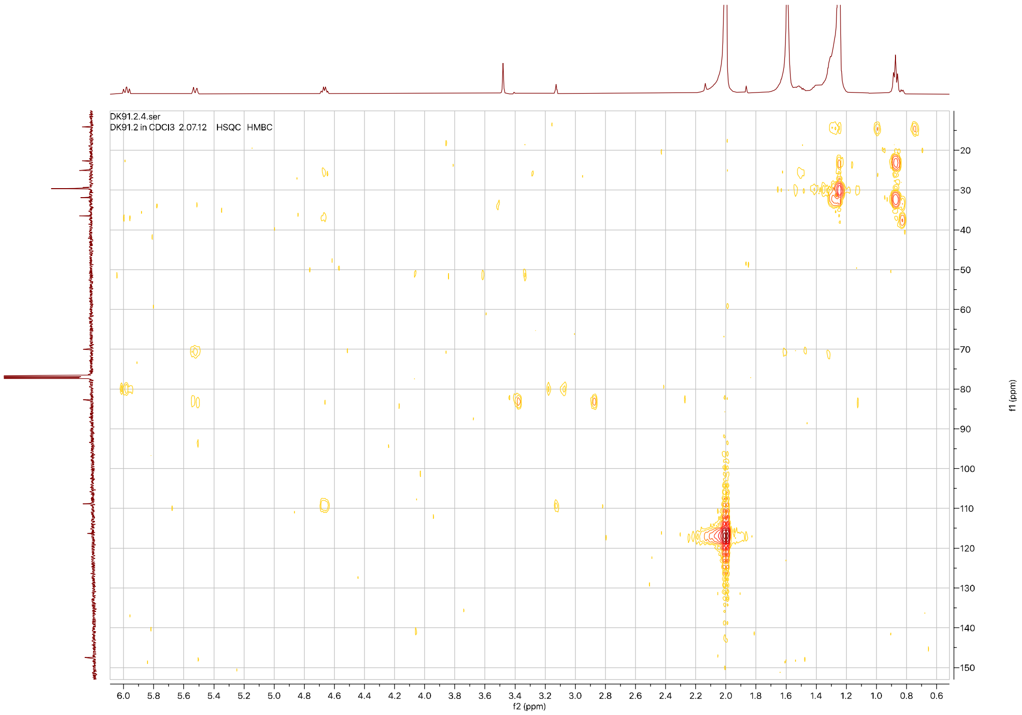

Figure S80. COSY spectrum of (5*S*)-icos-(3*Z*)-en-1-yn-5-ol (**13**) in $CDCl_3$

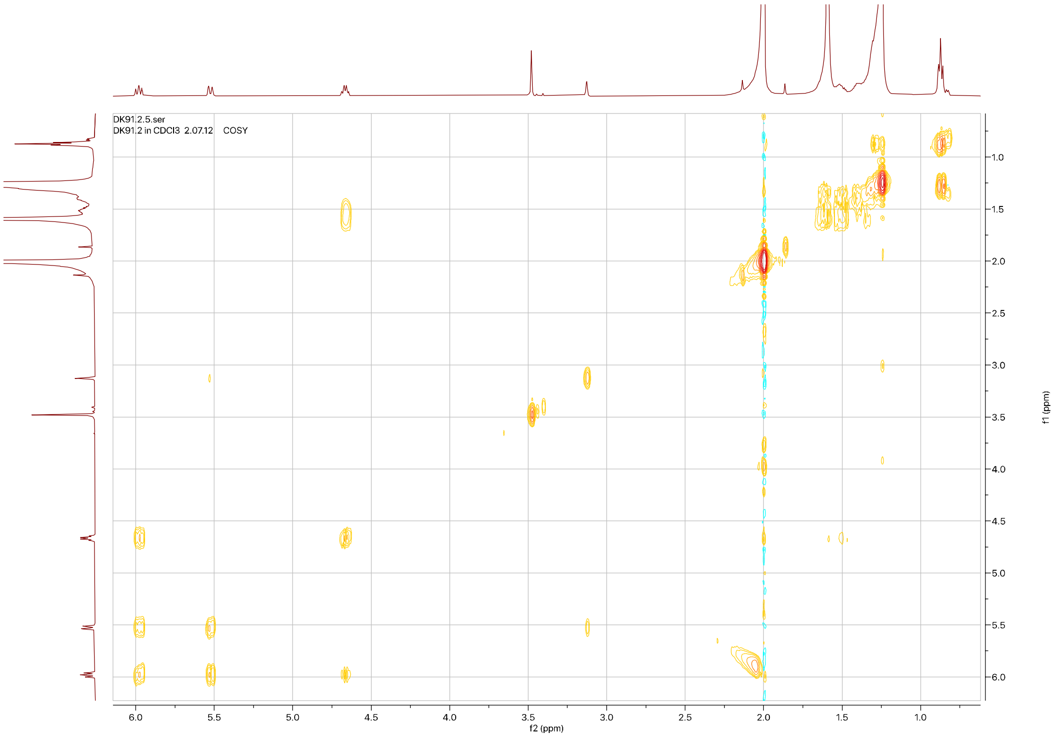


Figure S81. DEPT spectrum of (5*S*)-icos-(3*Z*)-en-1-yn-5-ol (**13**) in $CDCl_3$

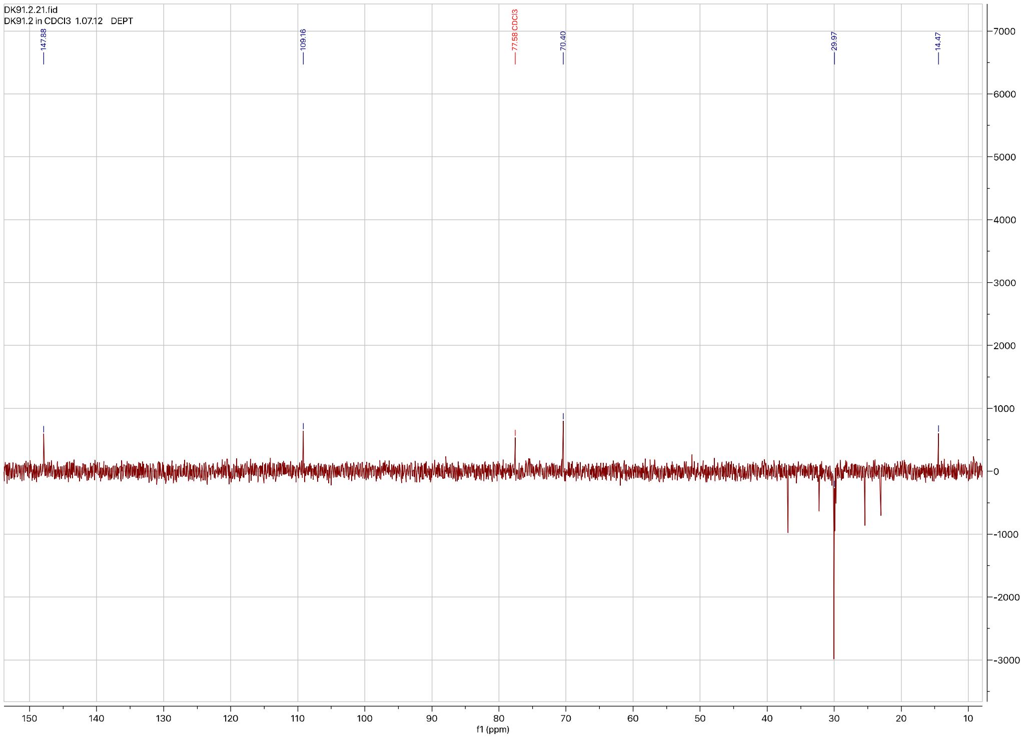

Table S13. NMR data of (5*S*)-icos-(3*Z*)-en-1-yn-5-ol (**13**) in $CDCl_3$.[a]

| Position | $\delta_C$, mult.[b] | $\delta_H$, mult. *J* (Hz) | LR H-C Correlations[c] |
|---|---|---|---|
| 1 | 82.7[d] CH | 3.13 d (1.5) | 3 |
| 2 | 79.5[e] qC | - | 1, 4 |
| 3 | 108.8 CH | 5.52 dd (11.0, 1.5) | 1, 4, 5 |
| 4 | 147.5 CH | 5.98 dd (11.0, 8.9) | 1, 3, 5, 6a, 6b |
| 5 | 70.0 CH | 4.67 dt (8.9, 8.5) | 3, 4, 6a, 6b, 7a, 7b |
| 6a | 36.5 $CH_2$ | 1.61 m | 4, 5, 7a, 7b, 8 |
| b | | 1.52 m | |
| 7a | 28.8 $CH_2$ | 1.41 m | 5, 6a, 6b, 8 |
| b | | 1.32 m | |
| 8-17 | ~29.6[f] 10 × $CH_2$ | 1.23 – 1.35 brm | |
| 18 | 31.9 $CH_2$ | 1.26 m | 17, 19a, 19b, 20 |
| 19a | 22.7 $CH_2$ | 1.31 m | 18, 20 |
| b | | 1.26 m | |
| 20 | 14.1 $CH_3$ | 0.88 t (7.0) | 18, 19a, 19b |

[a]500.13 MHz for $^1H$ and 125.76 MHz for $^{13}C$; [b]Multiplicity and assignment from HSQC experiment; [c]Determined from HMBC experiment; [d] $^1J = 252.0$ Hz; [e] $^2J = 49.1$ Hz; [f]Exact $^{13}C$ chemical shifts 29.34, 29.50, 29.54, 29.54, 29.65 (× 3), 29.67 (× 3) ppm.

Figure S82. EIGCMS spectrum of (5*S*)-icos-(3*Z*)-en-1-yn-5-ol (**13**)

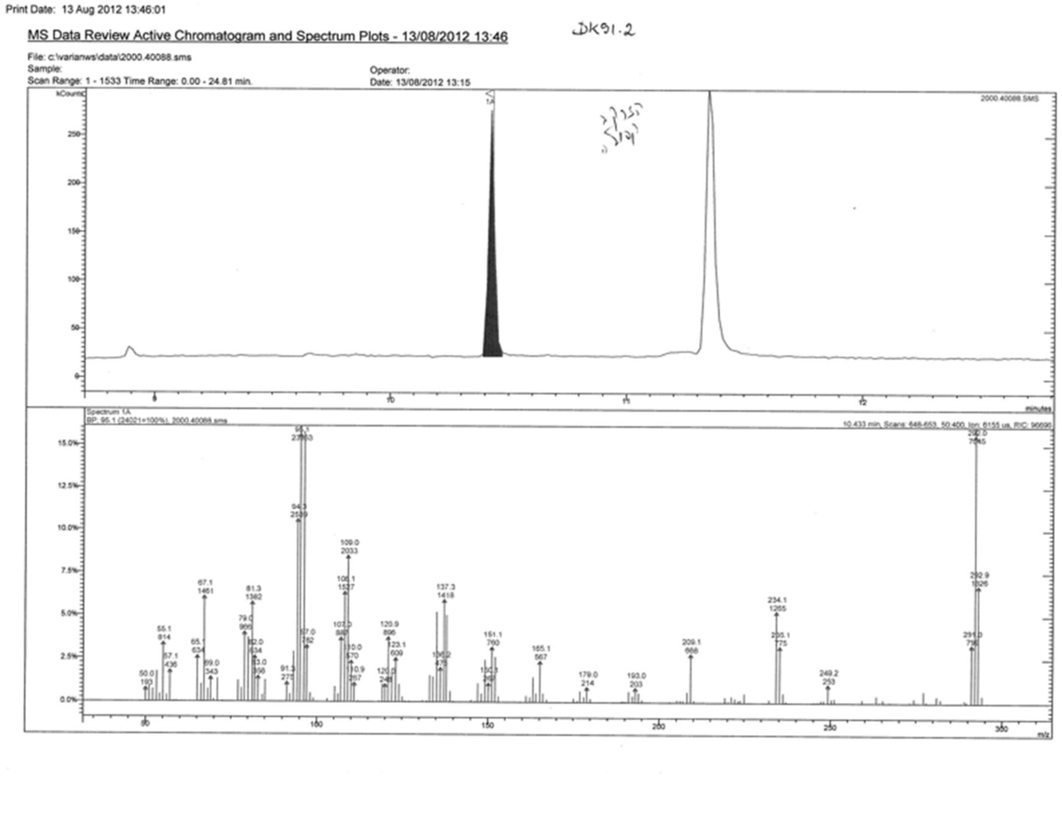

Figure S83. HRCIMS spectrum of (5*S*)-icos-(3*Z*)-en-1-yn-5-ol (**13**)

## Compound Spectrum SmartFormula Report

**Analysis Info**

| | | | |
|---|---|---|---|
| Analysis Name | D:\Data\Eichen\Acq000004.d | Acquisition Date | 5/17/2015 8:28:47 AM |
| Method | GC APCI_pos_Na_Formate.m | Operator | Larisa Panz |
| Sample Name | dk-91-2(17) | Instrument | maXis impact 282001.00128 |
| Comment | uNI | | |

**Acquisition Parameter**

| | | | | | |
|---|---|---|---|---|---|
| Source Type | APCI | Ion Polarity | Positive | Set Nebulizer | 4.0 Bar |
| Focus | Active | Set Capillary | 4000 V | Set Dry Heater | 200 °C |
| Scan Begin | 50 m/z | Set End Plate Offset | -500 V | Set Dry Gas | 2.0 l/min |
| Scan End | 1000 m/z | Set Charging Voltage | 2000 V | Set Divert Valve | Source |
| | | Set Corona | 4000 nA | Set APCI Heater | 450 °C |

**+MS, 6.7-7.0min #403-418**

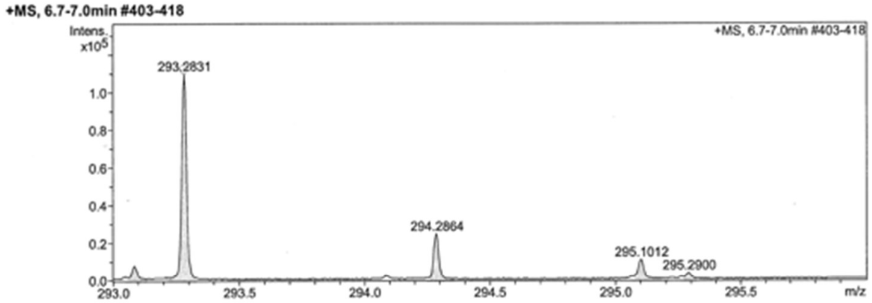


| Meas. m/z | # | Ion Formula | m/z | err [ppm] | mSigma | # mSigma | Score | rdb | e⁻ Conf | N-Rule | \|err\| [mDa] |
|---|---|---|---|---|---|---|---|---|---|---|---|
| 293.2831 | 1 | C20H37O | 293.2839 | 2.8 | 9.4 | 1 | 100.00 | 2.5 | even | ok | 0.8 |

Acq000004.d
Bruker Compass DataAnalysis 4.2 printed: 5/17/2015 9:31:06 AM by: Larisa Panz Page 1 of 1

Figure S84. $^{1}H$ NMR spectrum of (5*S*)-14-methylicos-(3*Z*)-en-1-yn-5-ol (**14**) in $CDCl_3$

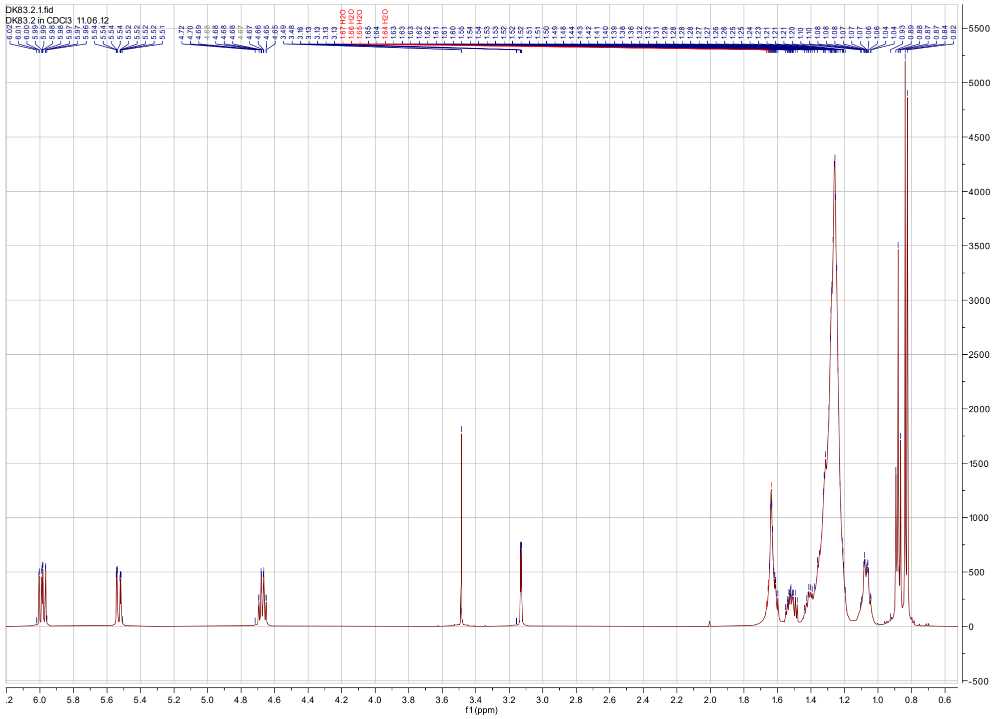


Figure S85. $^{13}C$ NMR spectrum of (5*S*)-14-methylicos-(3*Z*)-en-1-yn-5-ol (**14**) in $CDCl_3$

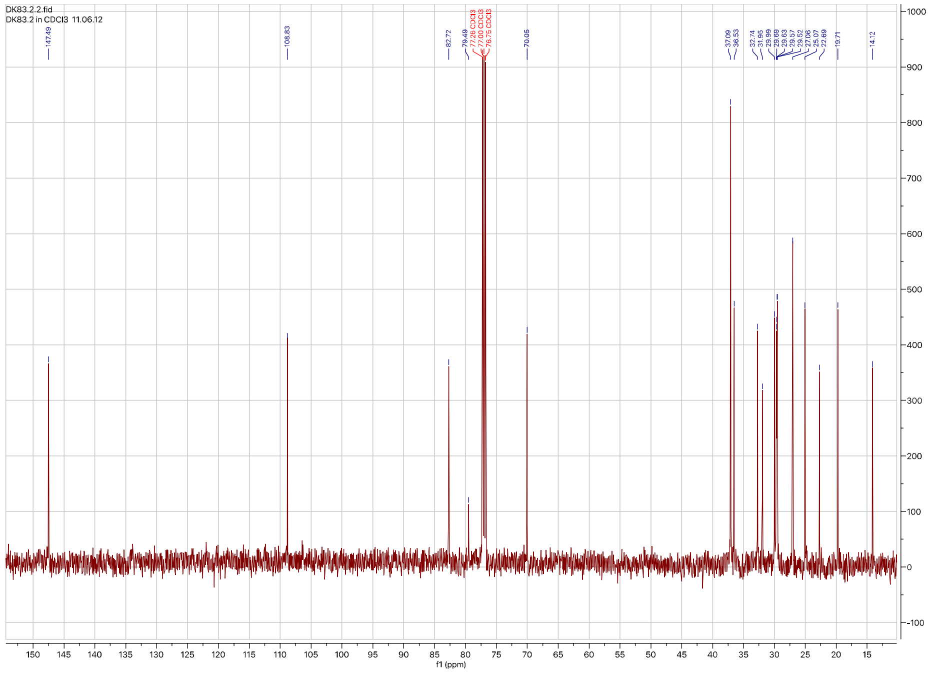

Figure S86. HSQC spectrum of (5*S*)-14-methylicos-(3*Z*)-en-1-yn-5-ol (**14**) in $CDCl_3$

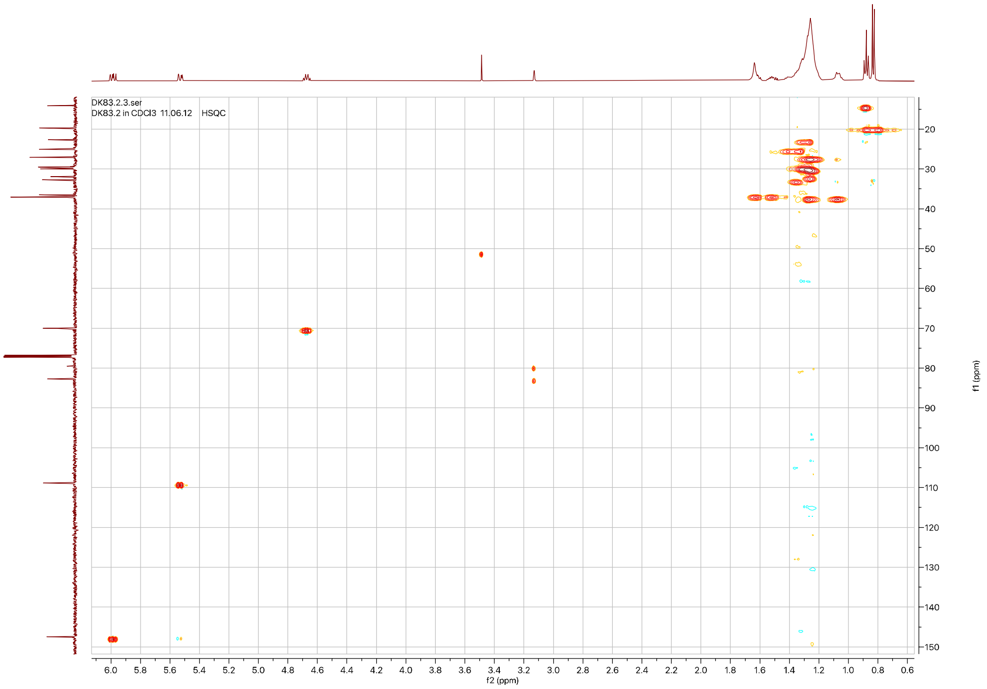


Figure S87. HMBC spectrum of (5*S*)-14-methylicos-(3*Z*)-en-1-yn-5-ol (**14**) in $CDCl_3$

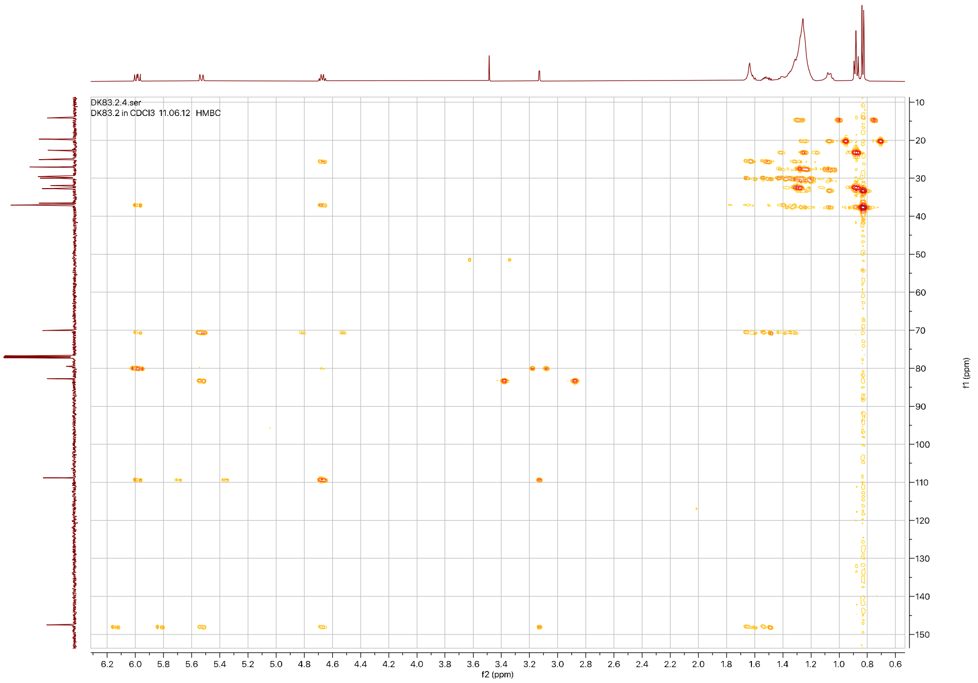

Figure S88. COSY spectrum of (5*S*)-14-methylicos-(3*Z*)-en-1-yn-5-ol (**14**) in $CDCl_3$

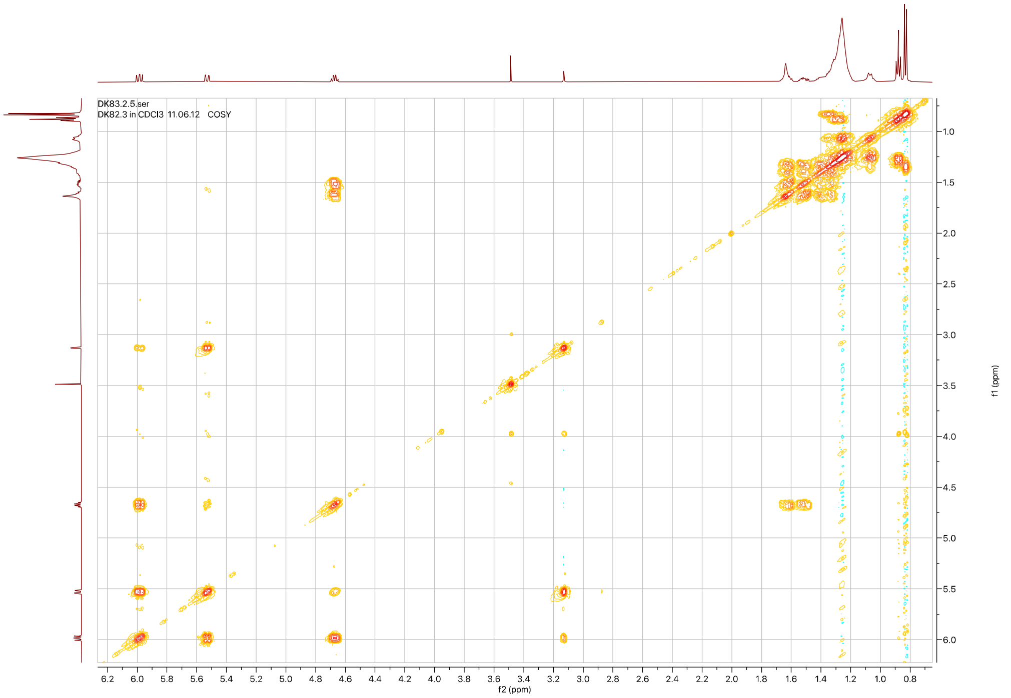


Figure S89. DEPT spectrum of (5*S*)-14-methylicos-(3*Z*)-en-1-yn-5-ol (**14**) in $CDCl_3$

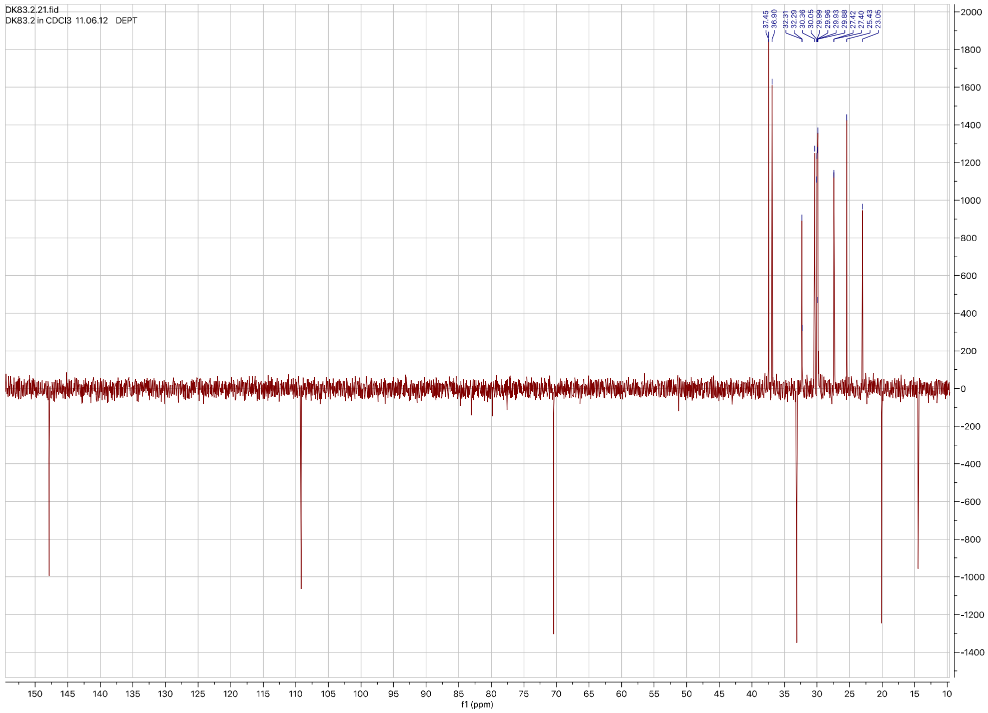

Table S14. NMR data of (5*S*)-14-methylicos-(3*Z*)-en-1-yn-5-ol (**14**) in $CDCl_3$.[a]

| Position | $\delta_C$, mult.[b] | $\delta_H$, mult. *J* (Hz) | LR H-C Correlations[c] |
|---|---|---|---|
| 1 | 82.7[d] CH | 3.13 dd (3.5, 1.0) | 3 |
| 2 | 79.5[e] qC | - | 1, 4 |
| 3 | 108.8 CH | 5.53 dd (11.0, 3.5, 1.0) | 1, 4, 5 |
| 4 | 147.5 CH | 5.98 ddd (11.0, 8.0, 1.0) | 1, 3, 5, 6a, 6b |
| 5 | 70.0 CH | 4.67 qd (8.0, 1.0) | 3, 4, 6a, 6b, 7a, 7b |
| 6a | 36.5 $CH_2$ | 1.62 m | 4, 5, 7a, 7b, 8 |
| b | | 1.52 m | |
| 7a | 25.1 $CH_2$ | 1.41 m | 5, 6a, 6b, 8 |
| b | | 1.32 m | |
| 8-11 | ~29.6[f] 4 × $CH_2$ | 1.21 – 1.33 brm | |
| 12 | 27.1 $CH_2$ | 1.26 m | 11, 13a, 13b |
| 13a | 37.1 $CH_2$ | 1.26 m | 12, 14, 15a, 15b, 21 |
| b | | 1.07 m | |
| 14 | 32.7 CH | 1.35 m | 13a, 13b, 15a, 15a, 21 |
| 15a | 37.1 $CH_2$ | 1.26 m | 13a, 13b, 14, 16, 21 |
| b | | 1.07 m | |
| 16 | 27.1 $CH_2$ | 1.26 m | 15a, 15b, 17 |
| 17 | ~29.6[f] $CH_2$ | 1.21 – 1.33 brm | |
| 18 | 32.3 $CH_2$ | 1.26 m | 17, 19a, 19b, 20 |
| 19a | 22.7 $CH_2$ | 1.31 m | 18, 20 |
| b | | 1.26 m | |
| 20 | 14.1 $CH_3$ | 0.88 t (7.0) | 18, 19a, 19b |
| 21 | 19.7 $CH_3$ | 0.83 d (6.5) | 13b, 15b |

[a]500.13 MHz for $^1H$ and 125.76 MHz for $^{13}C$; [b]Multiplicity and assignment from HSQC experiment; [c]Determined from HMBC experiment; [d] $^1J$ = 252.0 Hz; [e] $^2J$ = 49.1 Hz; [f]Exact $^{13}C$ chemical shifts 29.52, 29.56, 29.63, 29.67, 29.99 ppm.

Figure S90. EIGCMS spectrum and fragmentation of (5*S*)-14-methylicos-(3*Z*)-en-1-yn-5-ol (**14**)

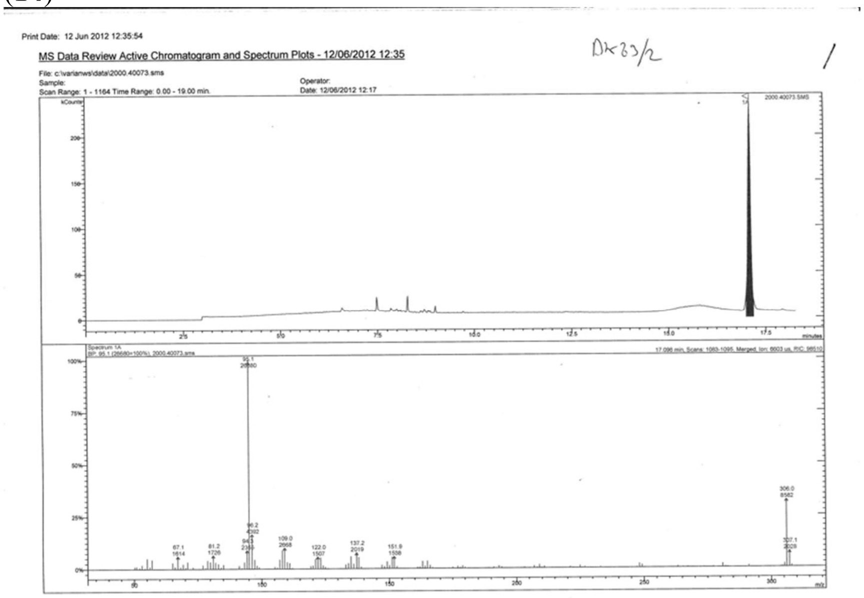


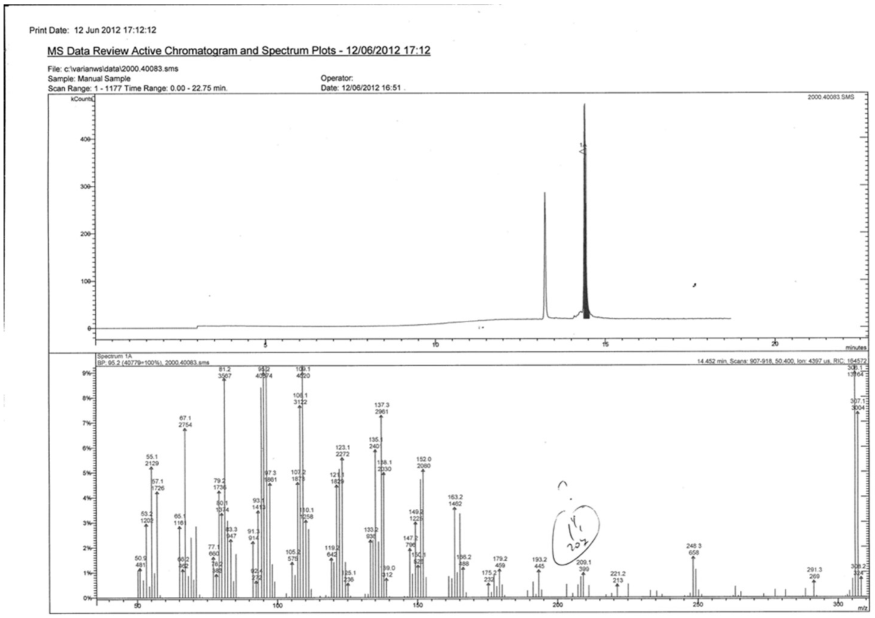


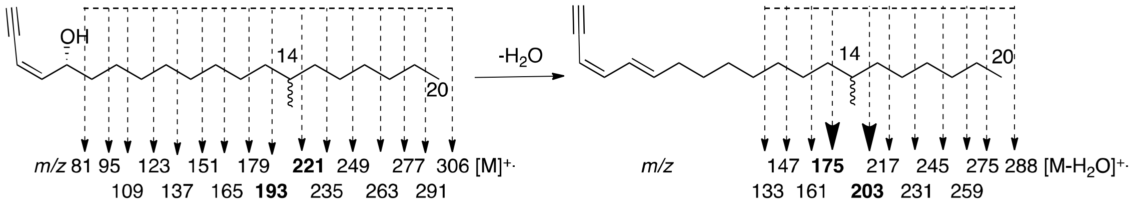

Figure S91. HRCIMS spectrum of (5*S*)-14-methylicos-(3*Z*)-en-1-yn-5-ol (**14**)

## Compound Spectrum SmartFormula Report

**Analysis Info**

| | | | |
|---|---|---|---|
| Analysis Name | D:\Data\Client\CLI_9279_8.d | Acquisition Date | 5/17/2015 10:53:27 AM |
| Method | CLI_9279.m | Operator | Larisa Panz |
| Sample Name | DK_83-2(14) | Instrument | maXis impact 282001.00128 |
| Comment | | | |

**Acquisition Parameter**

| | | | | | |
|---|---|---|---|---|---|
| Source Type | APCI | Ion Polarity | Positive | Set Nebulizer | 4.0 Bar |
| Focus | Active | Set Capillary | 4000 V | Set Dry Heater | 200 °C |
| Scan Begin | 50 m/z | Set End Plate Offset | -500 V | Set Dry Gas | 2.0 l/min |
| Scan End | 1000 m/z | Set Charging Voltage | 2000 V | Set Divert Valve | Source |
| | | Set Corona | 4000 nA | Set APCI Heater | 450 °C |

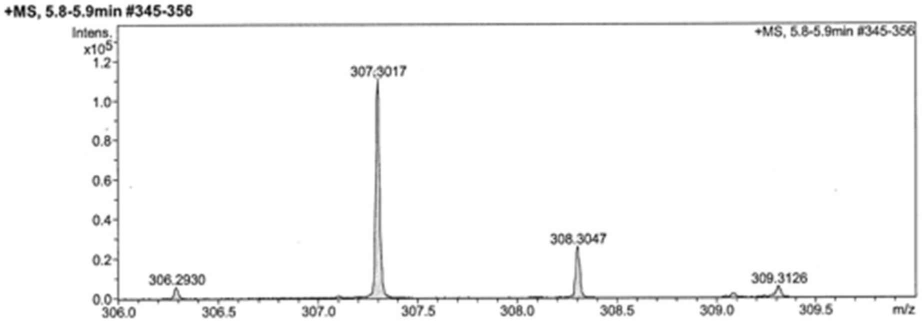


| Meas. m/z | # | Ion Formula | m/z | err [ppm] | mSigma | # mSigma | Score | rdb | e⁻ Conf | N-Rule | \|err\| [mDa] |
|---|---|---|---|---|---|---|---|---|---|---|---|
| 307.3017 | 1 | C21H39O | 307.2995 | -6.9 | 12.7 | 1 | 100.00 | 2.5 | even | ok | 2.1 |

CLI_9279_8.d
Bruker Compass DataAnalysis 4.2 printed: 5/17/2015 11:01:25 AM by: Larisa Panz Page 1 of 1

Figure S92. $^{1}$H NMR spectrum of (5*S*)-18-methylicos-(3*Z*)-en-1-yn-5-ol (**15**) and (5*S*)-19-methylicos-(3*Z*)-en-1-yn-5-ol (**16**) in $CDCl_3$

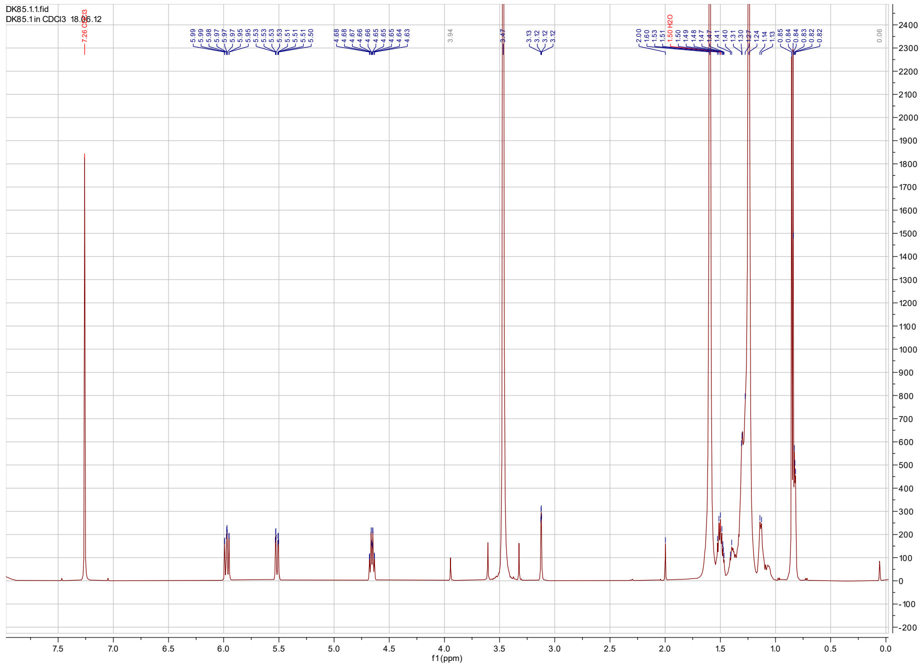


Figure S93. $^{13}$C NMR spectrum of (5*S*)-18-methylicos-(3*Z*)-en-1-yn-5-ol (**15**) and (5*S*)-19-methylicos-(3*Z*)-en-1-yn-5-ol (**16**) in $CDCl_3$

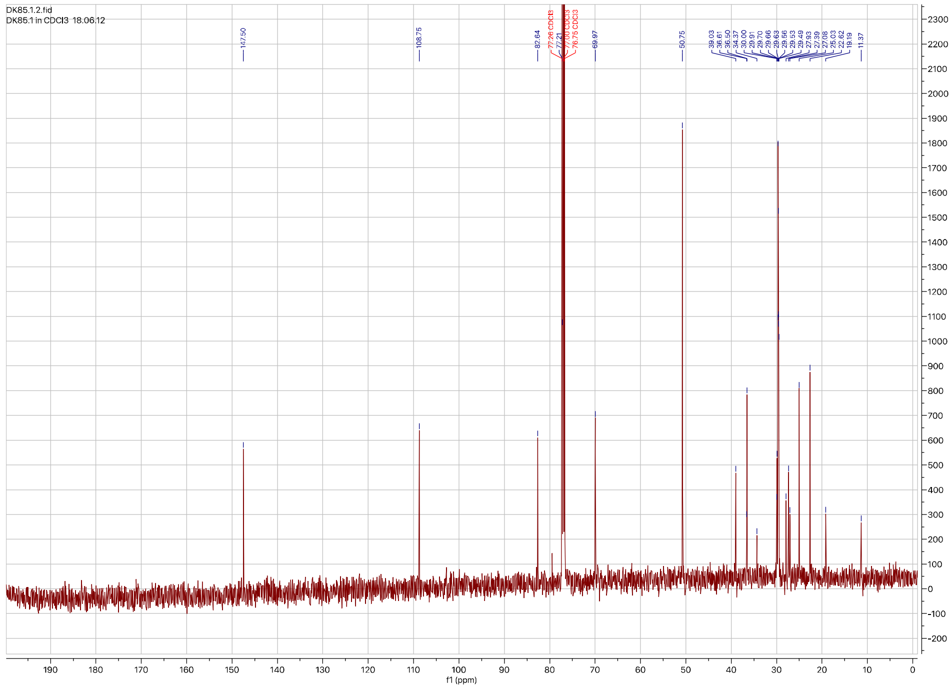

Figure S94. HSQC spectrum of (5*S*)-18-methylicos-(3*Z*)-en-1-yn-5-ol (**15**) and (5*S*)-19-methylicos-(3*Z*)-en-1-yn-5-ol (**16**) in $CDCl_3$

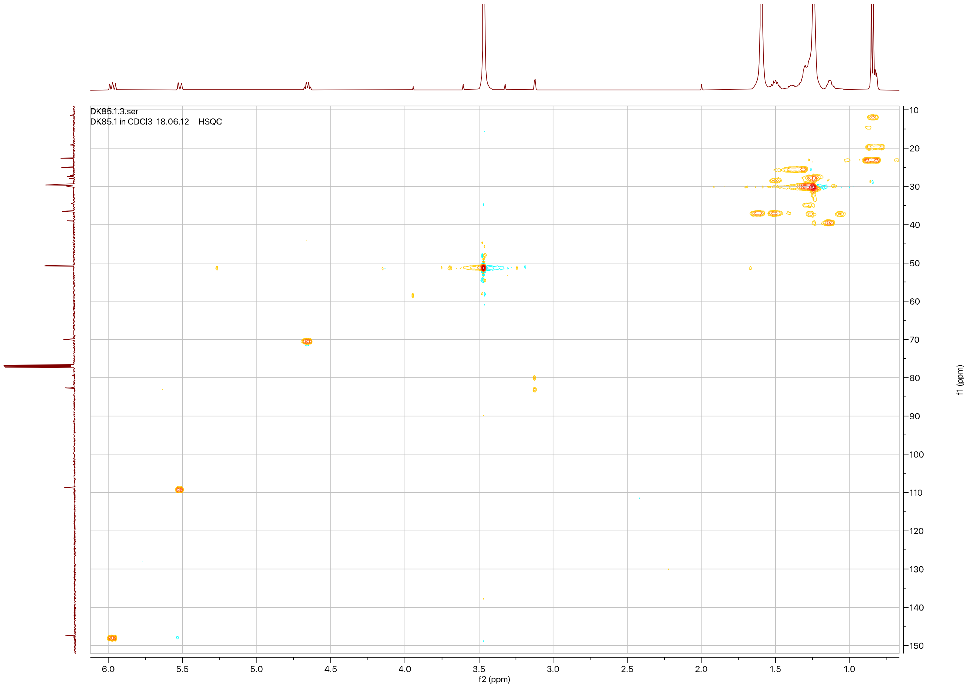


Figure S95. HMBC spectrum of (5*S*)-18-methylicos-(3*Z*)-en-1-yn-5-ol (**15**) and (5*S*)-19-methylicos-(3*Z*)-en-1-yn-5-ol (**16**) in $CDCl_3$

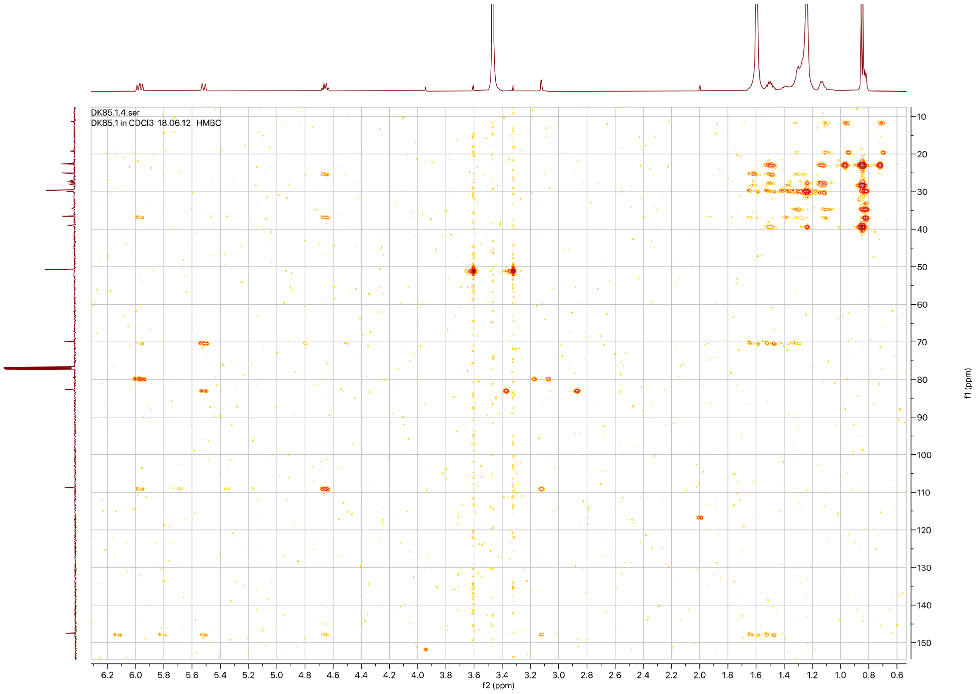

Figure S96. COSY spectrum of (5*S*)-18-methylicos-(3*Z*)-en-1-yn-5-ol (**15**) and (5*S*)-19-methylicos-(3*Z*)-en-1-yn-5-ol (**16**) in $CDCl_3$

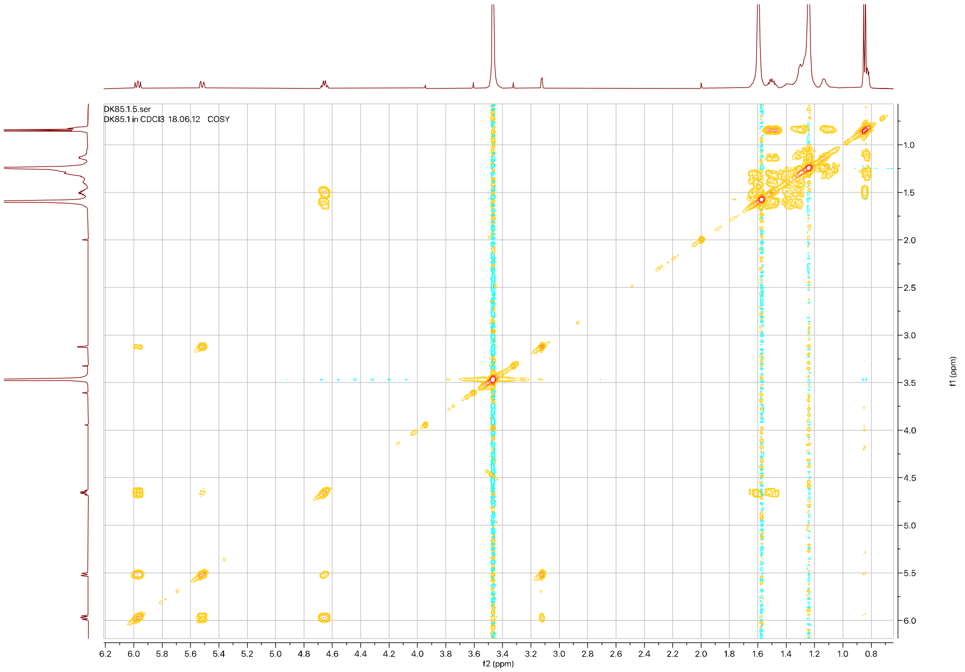


Figure S97. DEPT spectrum of (5*S*)-18-methylicos-(3*Z*)-en-1-yn-5-ol (**15**) and (5*S*)-19-methylicos-(3*Z*)-en-1-yn-5-ol (**16**) in $CDCl_3$

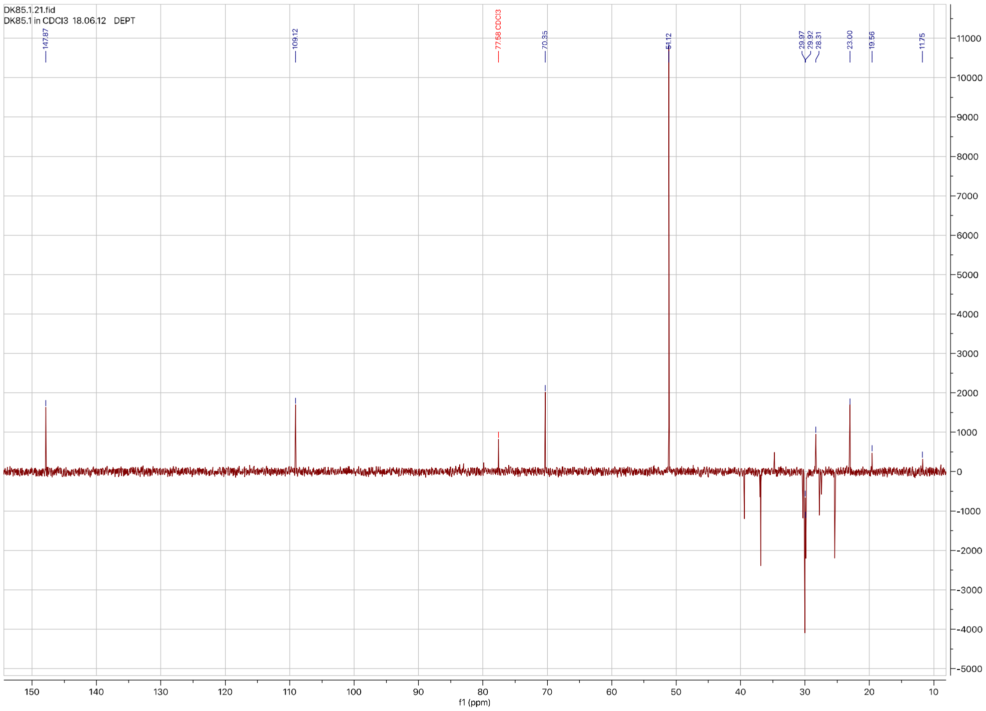

Table S15. NMR data of (5*S*)-18-methylicos-(3*Z*)-en-1-yn-5-ol (**15**) in $CDCl_3$.[a]

| Position | $\delta_C$, mult.[b] | $\delta_H$, mult. *J* (Hz) | LR H-C Correlations[c] |
|---|---|---|---|
| 1 | 82.6[d] CH | 3.13 d (2.0) | 3 |
| 2 | 79.6[e] qC | - | 1, 4 |
| 3 | 108.7 CH | 5.52 dd (11.0, 2.0) | 1, 4, 5 |
| 4 | 147.5 CH | 5.97 dd (11.0, 8.0) | 1, 3, 5, 6a, 6b |
| 5 | 70.0 CH | 4.65 q (8.0) | 3, 4, 6a, 6b, 7a, 7b |
| 6a | 36.5 $CH_2$ | 1.62 m | 4, 5, 7a, 7b, 8 |
| b | | 1.52 m | |
| 7a | 25.0 $CH_2$ | 1.39 m | 5, 6a, 6b, 8 |
| b | | 1.32 m | |
| 8-15 | ~29.6[f] 8 × $CH_2$ | 1.22 – 1.30 brm | |
| 16 | 27.1 $CH_2$ | 1.24 m | 15, 17a, 17b |
| 17a | 36.6 $CH_2$ | 1.06 m | 16, 18, 21 |
| b | | 1.26 m | |
| 18 | 34.4 CH | 1.28 m | 17a, 17b, 19a, 19b, 20, 21 |
| 19a | 22.6 $CH_2$ | 1.31 m | 20, 21 |
| b | | 1.10 m | |
| 20 | 11.4 $CH_3$ | 0.84 m | 19a, 19b |
| 21 | 19.2 $CH_3$ | 0.83 d (6.2) | 17a, 17b, 18, 19a, 19b |

[a]500.13 MHz for $^1H$ and 125.76 MHz for $^{13}C$; [b]Multiplicity and assignment from HSQC experiment; [c]Determined from HMBC experiment; [d] $^1J$ = 251.5 Hz; [e] $^2J$ = 50.6 Hz; [f]Exact $^{13}C$ chemical shifts 29.53, 29.56, 29.66 (× 3), 29.70, 29.91, 30.00 ppm.

Table S16. NMR data of (5*S*)-19-methylicos-(3*Z*)-en-1-yn-5-ol (**16**) in $CDCl_3$.[a]

| Position | $\delta_C$, mult.[b] | $\delta_H$, mult. *J* (Hz) | LR H-C Correlations[c] |
|---|---|---|---|
| 1 | 82.6[d] CH | 3.13 d (2.0) | 3 |
| 2 | 79.6[e] qC | - | 1, 4 |
| 3 | 108.7 CH | 5.52 dd (10.5, 2.0) | 1, 4, 5 |
| 4 | 147.5 CH | 5.97 dd (10.5, 8.0) | 1, 3, 5, 6a, 6b |
| 5 | 70.0 CH | 4.65 q (8.0) | 3, 4, 6a, 6b, 7a, 7b |
| 6a | 36.5 $CH_2$ | 1.62 m | 4, 5, 7a, 7b, 8 |
| b | | 1.52 m | |
| 7a | 25.0 $CH_2$ | 1.39 m | 5, 6a, 6b, 8 |
| b | | 1.32 m | |
| 8-16 | ~29.6[f] 9 × $CH_2$ | 1.22 – 1.30 brm | |
| 17 | 27.4 $CH_2$ | 1.24 m | 16, 18 |
| 18 | 39.0 $CH_2$ | 1.13 m | 17, 19, 20, 21 |
| 19 | 27.9 CH | 1.50 m | 18, 20, 21 |
| 20 | 22.6 $CH_3$ | 0.85 d (6.5) | 18, 19, 21 |
| 21 | 22.6 $CH_3$ | 0.85 d (6.5) | 18, 19, 20 |

[a]500.13 MHz for $^1H$ and 125.76 MHz for $^{13}C$; [b]Multiplicity and assignment from HSQC experiment; [c]Determined from HMBC experiment; [d] $^1J$ = 251.5 Hz; [e] $^2J$ = 50.6 Hz; [f]Exact $^{13}C$ chemical shifts 29.49, 29.53, 29.56, 29.66 (× 6) ppm.

Figure S98. EIGCMS spectrum of (5*S*)-18-methylicos-(3*Z*)-en-1-yn-5-ol (**15**)

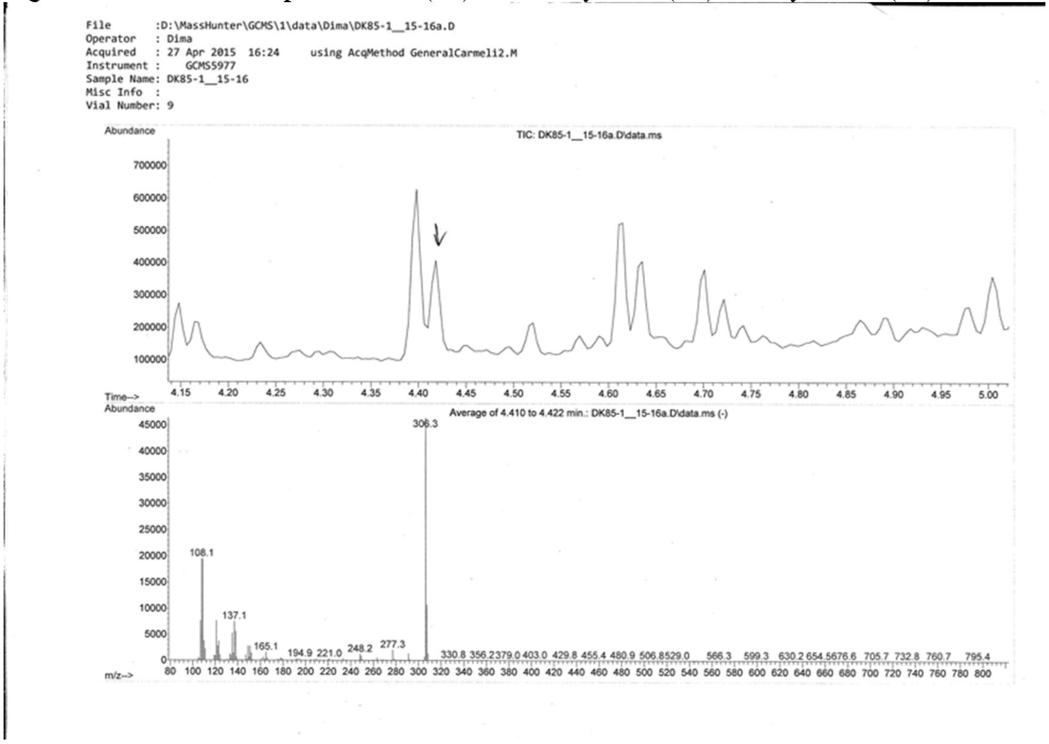


Figure S99. EIGCMS spectrum of (5*S*)-19-methylicos-(3*Z*)-en-1-yn-5-ol (**16**)

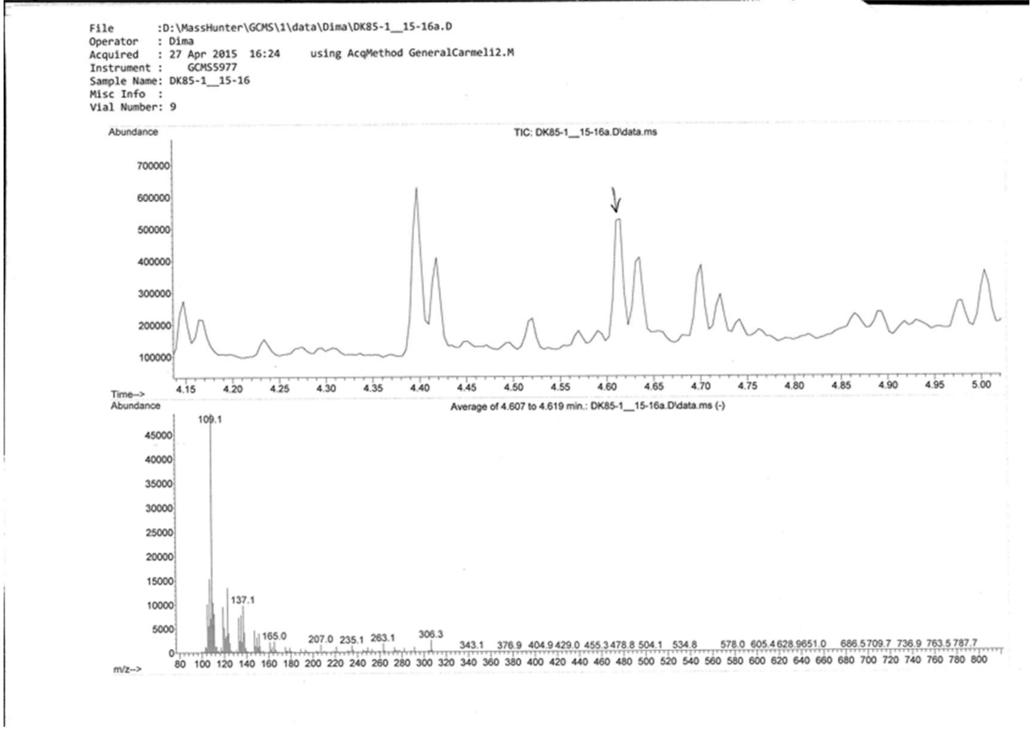

Figure S100. HRCIMS spectrum of (5*S*)-18-methylicos-(3*Z*)-en-1-yn-5-ol (**15**)

## Compound Spectrum SmartFormula Report

**Analysis Info**

| | | | |
|---|---|---|---|
| Analysis Name | D:\Data\Client\CLI_9279_22000001.d | Acquisition Date | 5/17/2015 1:38:53 PM |
| Method | GC APCI_pos_cli_8051.m | Operator | Larisa Panz |
| Sample Name | DK85.1(15+16) (15) | Instrument | maXis impact 282001.00128 |
| Comment | | | |

**Acquisition Parameter**

| | | | | | |
|---|---|---|---|---|---|
| Source Type | APCI | Ion Polarity | Positive | Set Nebulizer | 4.0 Bar |
| Focus | Active | Set Capillary | 4000 V | Set Dry Heater | 200 °C |
| Scan Begin | 50 m/z | Set End Plate Offset | -500 V | Set Dry Gas | 2.0 l/min |
| Scan End | 1000 m/z | Set Charging Voltage | 2000 V | Set Divert Valve | Source |
| | | Set Corona | 4000 nA | Set APCI Heater | 450 °C |

**+MS, 4.0-4.5min #238-268**

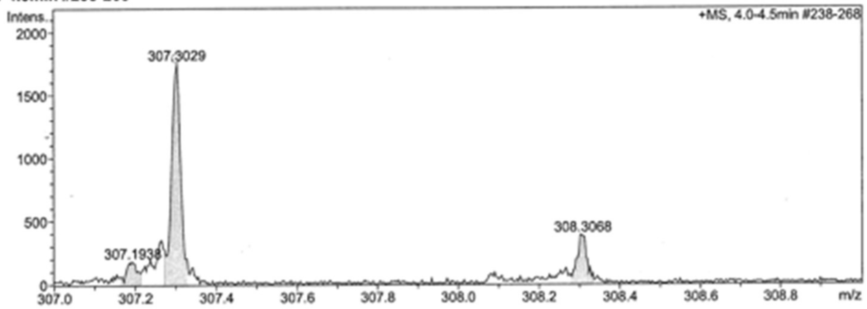


| Meas. m/z | # | Ion Formula | m/z | err [ppm] | mSigma | # mSigma | Score | rdb | e⁻ Conf | N-Rule | \|err\| [mDa] |
|---|---|---|---|---|---|---|---|---|---|---|---|
| 307.3029 | 1 | C21H39O | 307.2995 | -10.9 | 106.6 | 1 | 100.00 | 2.5 | even | ok | 3.3 |

CLI_9279_22000001.d
Bruker Compass DataAnalysis 4.2 printed: 5/17/2015 1:46:58 PM by: Larisa Panz Page 1 of 1

Figure S101. HRCIMS spectrum of (5*S*)-19-methylicos-(3*Z*)-en-1-yn-5-ol (**16**)

## Compound Spectrum SmartFormula Report

**Analysis Info**

Analysis Name D:\Data\Client\CLI_9279_22000001.d
Method GC APCI_pos_cli_8051.m
Sample Name DK85.1(15+16)
Comment

16

Acquisition Date 5/17/2015 1:38:53 PM
Operator Larisa Panz
Instrument maXis impact 282001.00128

**Acquisition Parameter**

| | | | | | |
|---|---|---|---|---|---|
| Source Type | APCI | Ion Polarity | Positive | Set Nebulizer | 4.0 Bar |
| Focus | Active | Set Capillary | 4000 V | Set Dry Heater | 200 °C |
| Scan Begin | 50 m/z | Set End Plate Offset | -500 V | Set Dry Gas | 2.0 l/min |
| Scan End | 1000 m/z | Set Charging Voltage | 2000 V | Set Divert Valve | Source |
| | | Set Corona | 4000 nA | Set APCI Heater | 450 °C |

**+MS, 6.2-6.7min #371-402**

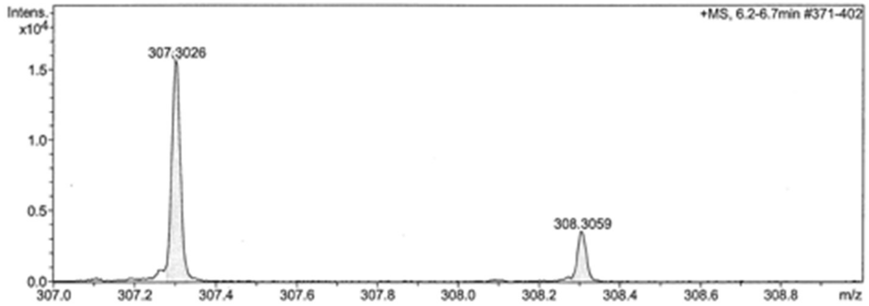


| Meas. m/z | # | Ion Formula | m/z | err [ppm] | mSigma | # mSigma | Score | rdb | e⁻ Conf | N-Rule | \|err\| [mDa] |
|---|---|---|---|---|---|---|---|---|---|---|---|
| 307.3026 | 1 | C21H39O | 307.2995 | -9.9 | 27.5 | 1 | 100.00 | 2.5 | even | ok | 3.0 |

CLI_9279_22000001.d
Bruker Compass DataAnalysis 4.2 printed: 5/17/2015 1:49:56 PM by: Larisa Panz Page 1 of 1

Figure S102. $^{1}H$ NMR spectrum of 14-methyldocos-(3*Z*)-en-1-yn-5,6-diol (**17**) in $CDCl_3$

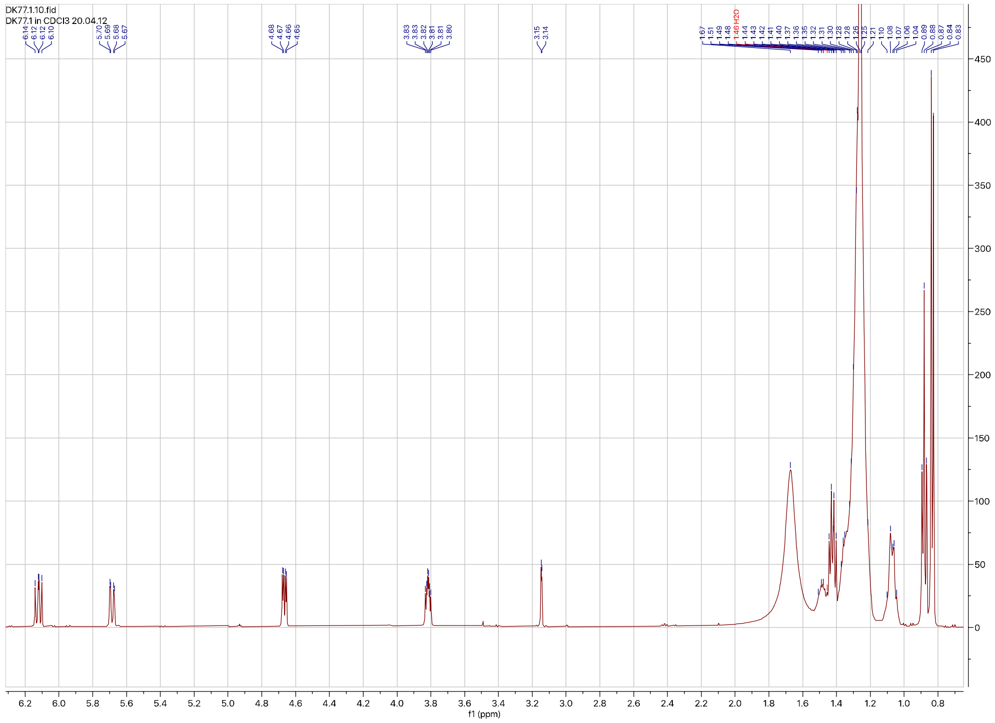


Figure S103. $^{13}C$ NMR spectrum of 14-methyldocos-(3*Z*)-en-1-yn-5,6-diol (**17**) in $CDCl_3$

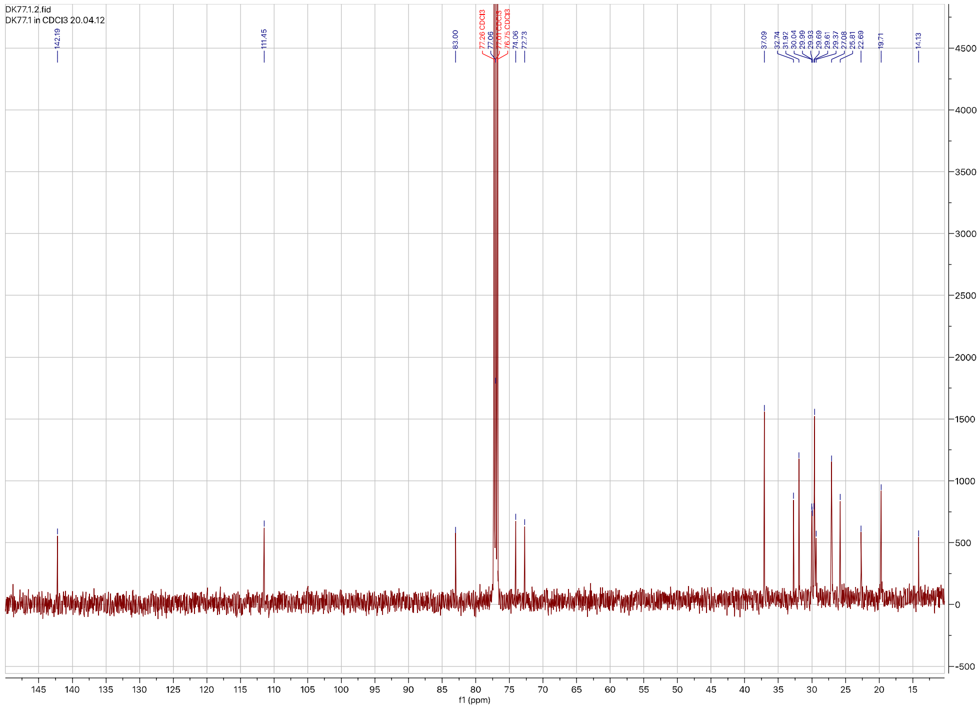

Figure S104. HSQC spectrum of 14-methyldocos-(3*Z*)-en-1-yn-5,6-diol (**17**) in $CDCl_3$

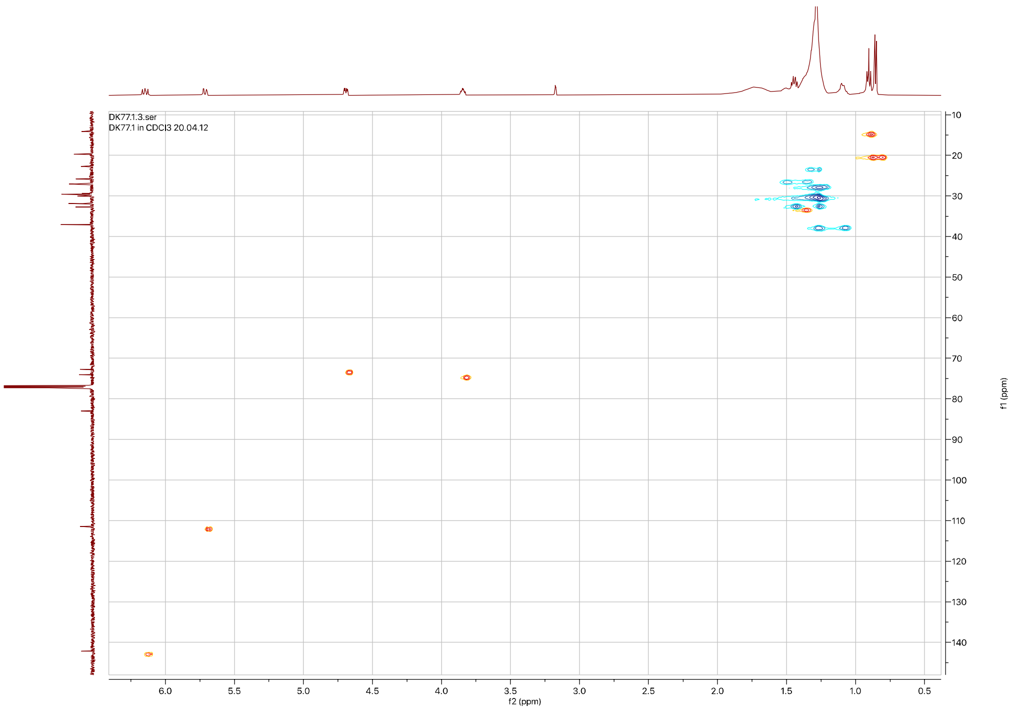


Figure S105. HMBC spectrum of 14-methyldocos-(3*Z*)-en-1-yn-5,6-diol (**17**) in $CDCl_3$

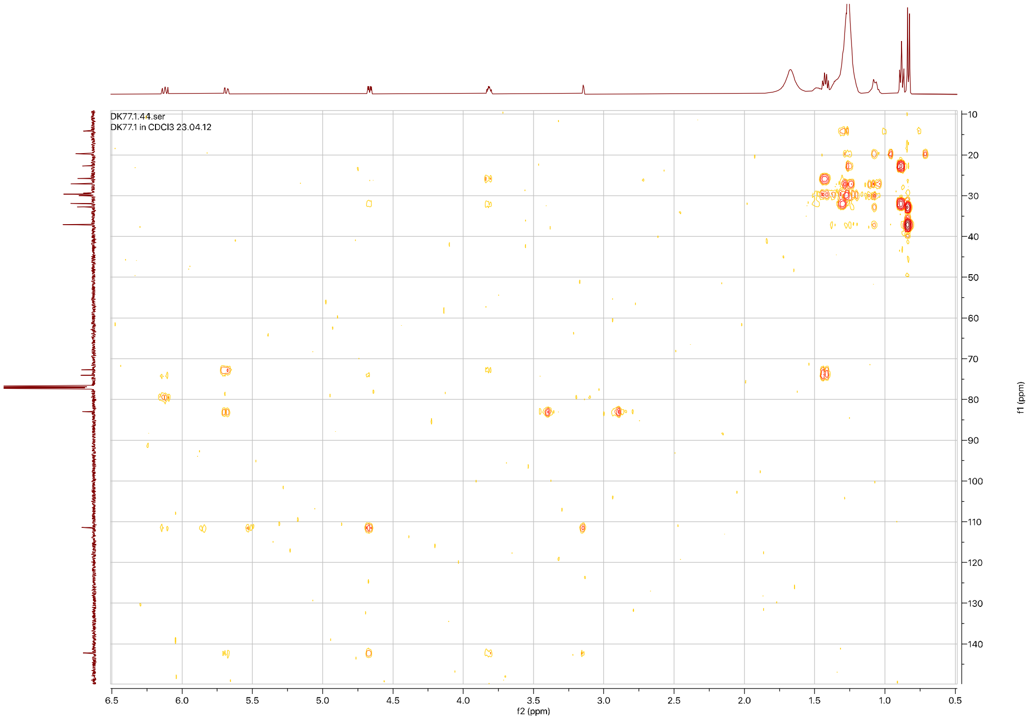

Figure S106. COSY spectrum of 14-methyldocos-(3*Z*)-en-1-yn-5,6-diol (**17**) in $CDCl_3$

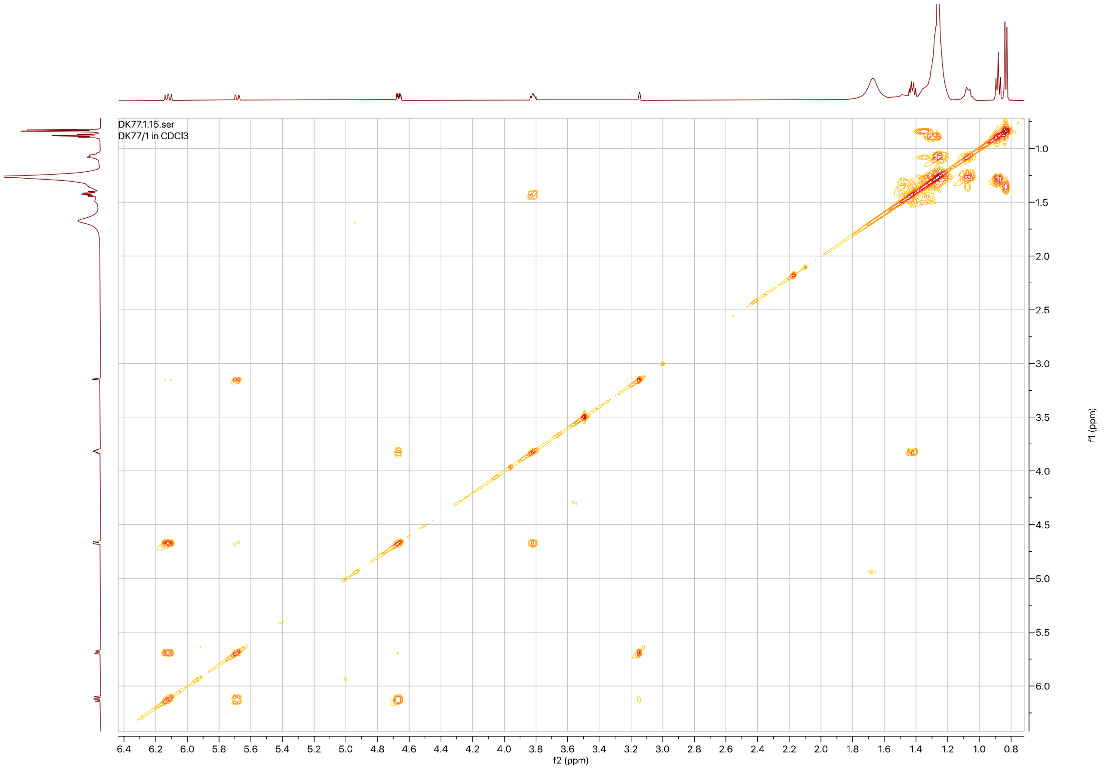

Table S17. NMR data of 14-methyldocos-(3*Z*)-en-1-yn-5,6-diol (**17**) in $CDCl_3$.[a]

| Position | $\delta_C$, mult.[b] | $\delta_H$, mult. *J* (Hz) | LR H-C Correlations[c] |
|---|---|---|---|
| 1 | 83.0[d] CH | 3.16 d (1.5) | 3 |
| 2 | 79.3[e] qC | - | 1, 4 |
| 3 | 111.4 CH | 5.68 dd (11.0, 1.5) | 1, 4, 5 |
| 4 | 147.5 CH | 6.12 dd (11.0, 9.0) | 1, 3, 5, 6 |
| 5 | 72.7 CH | 4.68 dd (9.0,3.0) | 3, 6, 7 |
| 6 | 74.1 CH | 3.82 td (6.5, 3.0) | 4, 5, 7 |
| 7 | 31.9 $CH_2$ | 1.43 q (6.5) | 5, 6, 8a, 8b |
| 8a | 25.8 $CH_2$ | 1.49 m | 6, 7 |
| b | | 1.34 m | |
| 9 | 29.4 $CH_2$ | 1.28 m | 7 |
| 10-11 | ~29.6[f] 2 × $CH_2$ | 1.24 – 1.28 brm | |
| 12 | 27.1 $CH_2$ | 1.25 m | 11, 13a, 13b |
| 13a | 37.1 $CH_2$ | 1.26 m | 14, 15a, 15b, 23 |
| b | | 1.06 m | |
| 14 | 32.7 CH | 1.35 m | 13a, 13b, 15a, 15b, 23 |
| 15a | 37.1 $CH_2$ | 1.26 m | 13a, 13b, 14, 23 |
| b | | 1.06 m | |
| 16 | 27.1 $CH_2$ | 1.25 m | 15a, 15b, 17 |
| 17-19 | ~29.6[f] 3 × $CH_2$ | 1.24 – 1.28 brm | |
| 20 | 31.9 CH | 1.25 m | 19, 21a, 21b, 22 |
| 21a | 22.7 $CH_2$ | 1.32 m | 20, 22 |
| b | | 1.26 m | |
| 22 | 14.1 $CH_3$ | 0.84 t (6.5) | 21a, 21b |
| 23 | 19.7 $CH_3$ | 0.83 d (6.5) | 13a, 13b, 14, 15a, 15b |

[a]500.13 MHz for $^1$H and 125.76 MHz for $^{13}$C; [b]Multiplicity and assignment from HSQC experiment; [c]Determined from HMBC experiment; [d] $^1J$ = 250.5 Hz; [e] $^2J$ = 45.0 Hz; [f]Exact $^{13}$C chemical shifts 29.61, 29.69, 29.93, 29.98, 30.03 ppm.

Figure S107. EIMS and fragmentation pattern of 14-methyldocos-(3*Z*)-en-1-yn-5,6-diol (**17**)

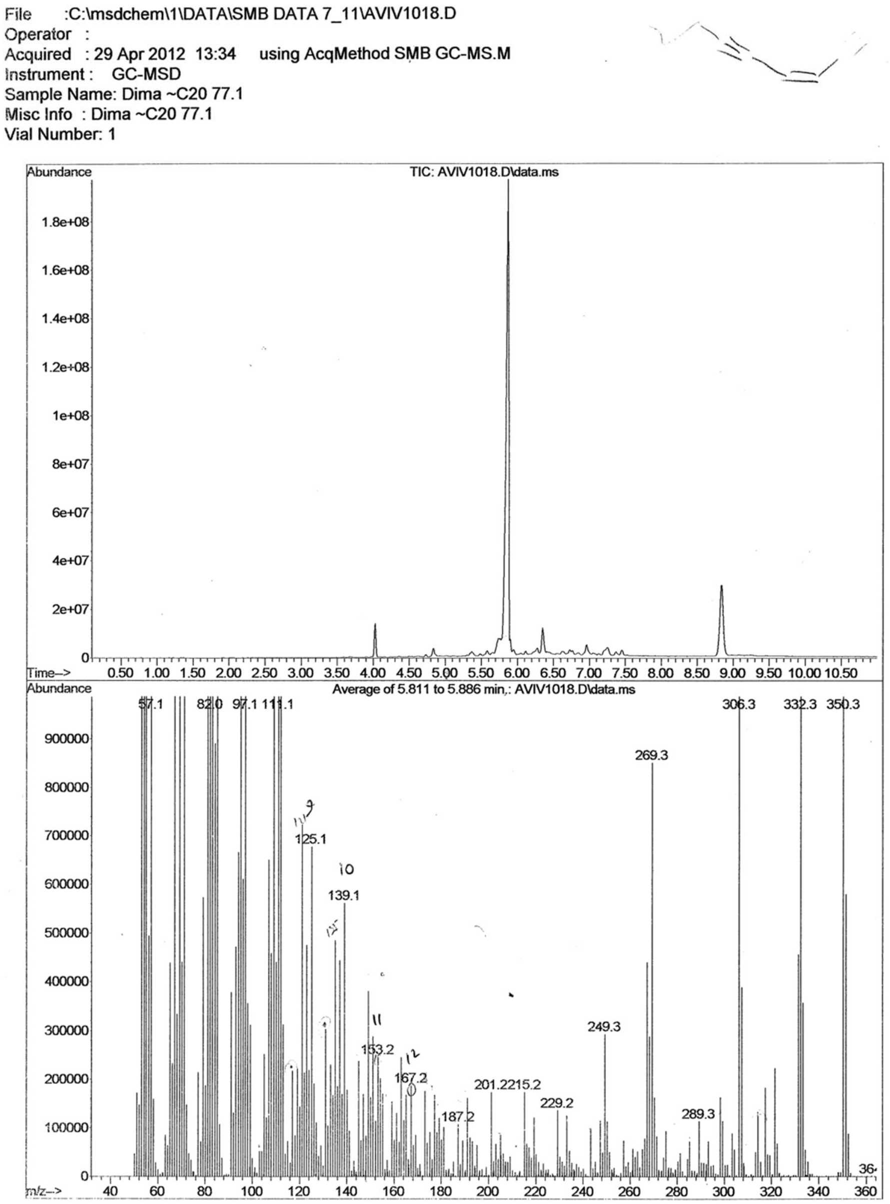


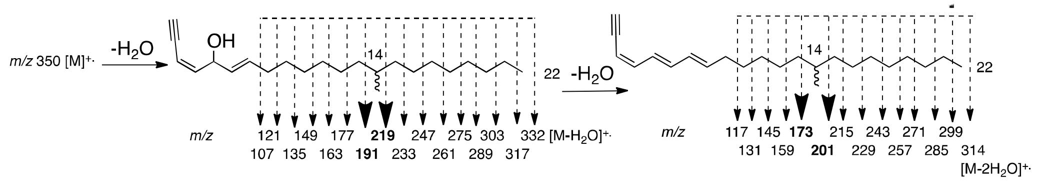

Figure S108. $^{1}$H NMR spectrum of (3*R*)-icos-(4*E*)-en-1-yn-3-ol (**18**) in $CDCl_3$

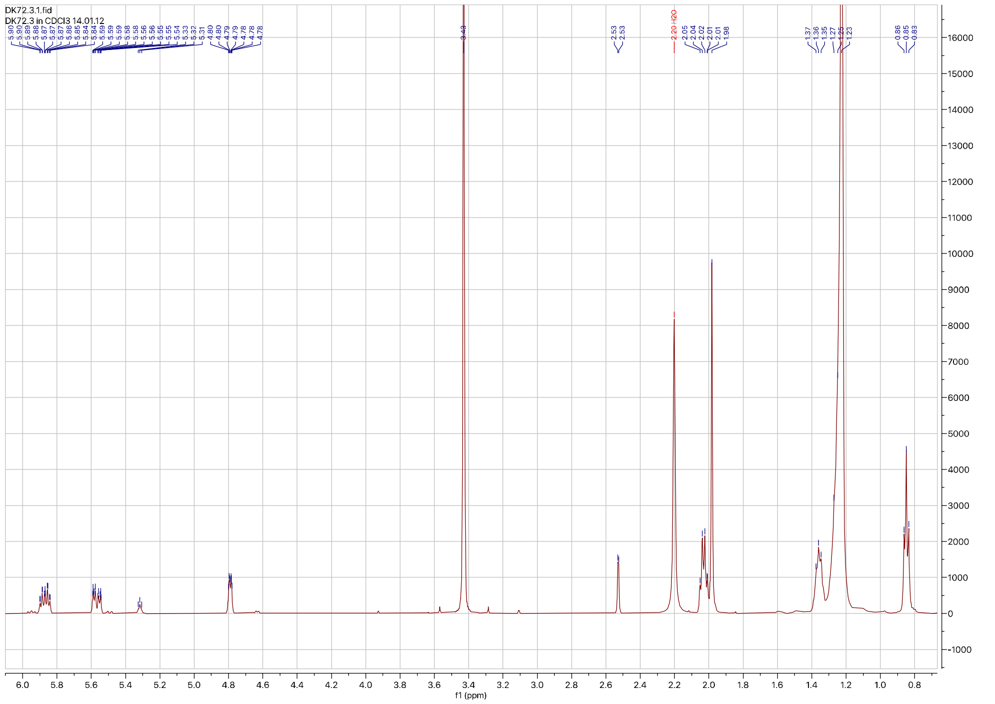

Figure S109. $^{13}$C NMR spectrum of(3*R*)-icos-(4*E*)-en-1-yn-3-ol (**18**) in $CDCl_3$

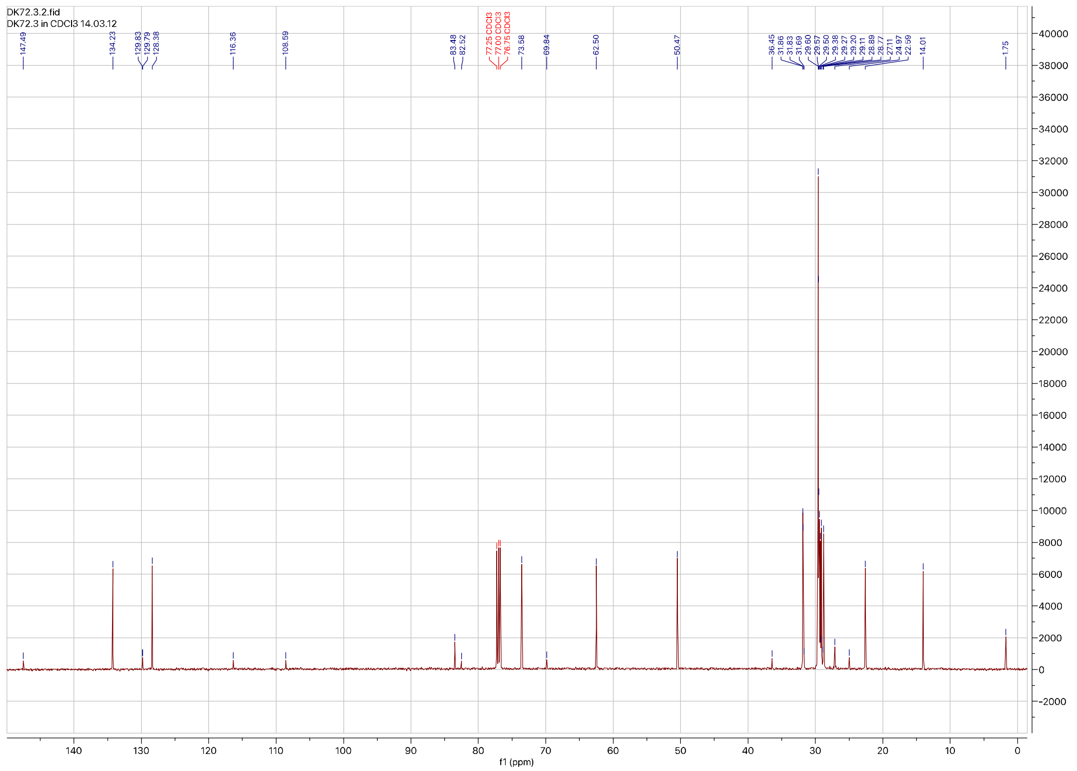

Figure S110. HSQC spectrum of(3*R*)-icos-(4*E*)-en-1-yn-3-ol (**18**) in $CDCl_3$.

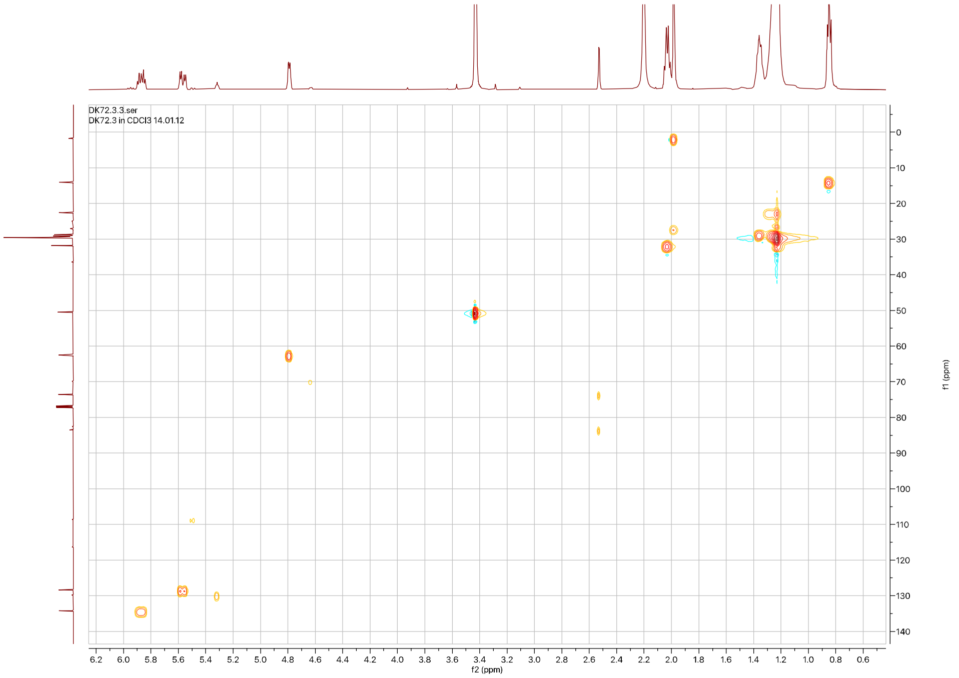


Figure S111. HMBC spectrum of(3*R*)-icos-(4*E*)-en-1-yn-3-ol (**18**) in $CDCl_3$.

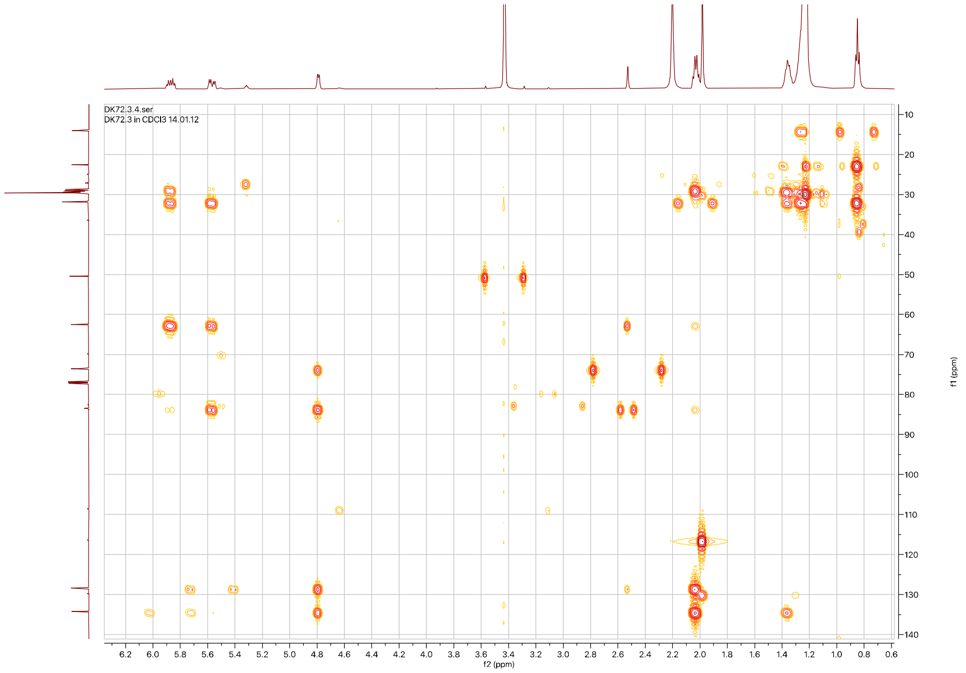

Figure S112. COSY spectrum of(3*R*)-icos-(4*E*)-en-1-yn-3-ol (**18**) in $CDCl_3$.

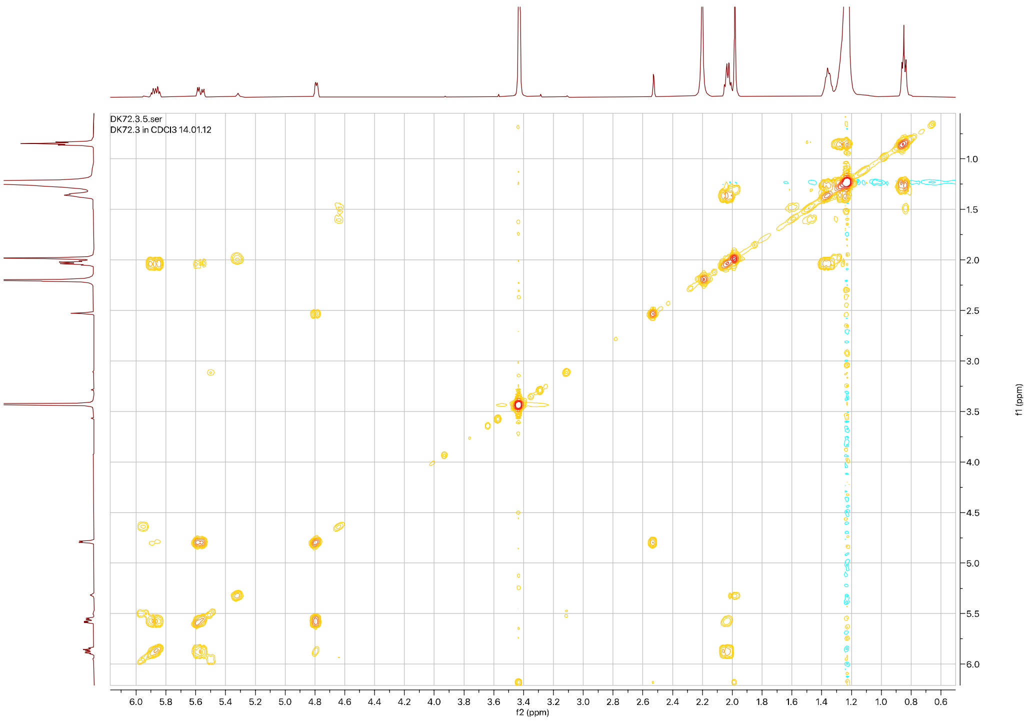

Figure S113. DEPT spectrum of(3*R*)-icos-(4*E*)-en-1-yn-3-ol (**18**) in $CDCl_3$.

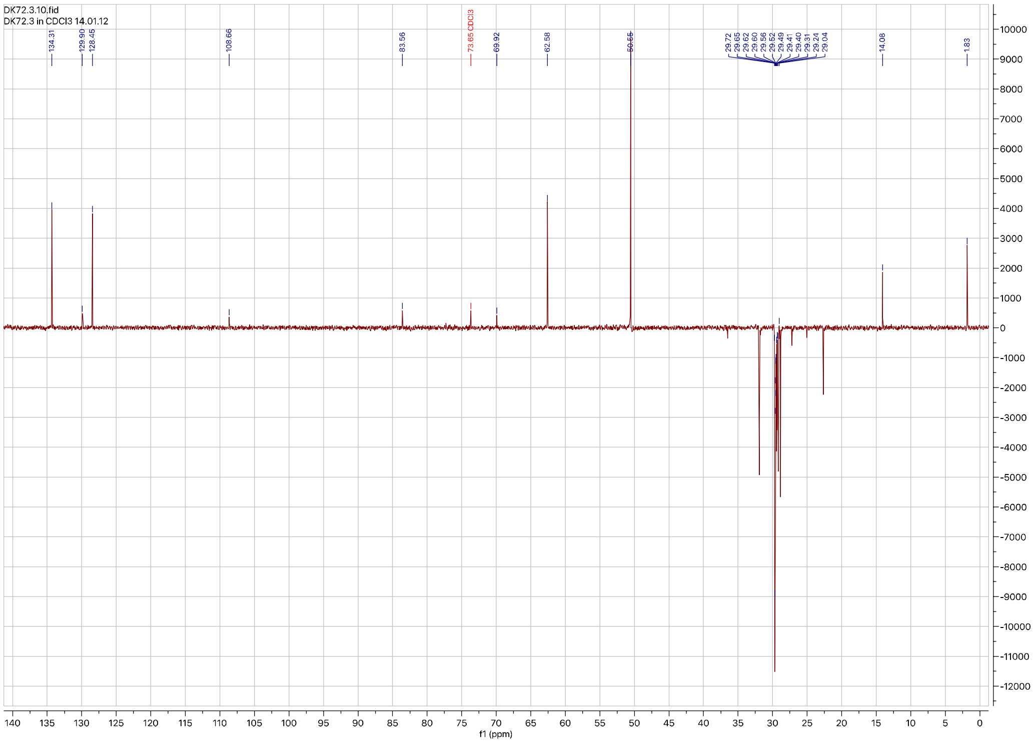

Table S18. NMR data of (3*R*)-icos-(4*E*)-en-1-yn-3-ol (**18**) in $CDCl_3$.[a]

| Position | $\delta_C$, mult.[b] | $\delta_H$, mult. *J* (Hz) | LR H-C Correlations[c] |
|---|---|---|---|
| 1 | 73.6[d] CH | 2.53 d (2.0) | 3 |
| 2 | 83.5[e] qC | - | 1, 3, 4 |
| 3 | 62.5 CH | 4.79 d (6.0) | 1, 4, 5 |
| 4 | 128.4 CH | 5.56 dd (15.5, 6.0) | 3, 6 |
| 5 | 134.5 CH | 5.87 dt (15.5, 7.0) | 3, 6, 7 |
| 6 | 31.9 $CH_2$ | 2.03 q (7.0) | 5, 7, 8 |
| 7 | 28.8 $CH_2$ | 1.36 m | 5, 6, 8 |
| 8-17 | ~29.6[f] 10 × $CH_2$ | 1.21 – 1.28 brm | |
| 18 | 31.8 CH | 1.23 m | 17, 19a, 19b, 20 |
| 19a | 22.6 $CH_2$ | 1.36 m | 18, 20 |
| b | | 1.26 m | |
| 20 | 14.0 $CH_3$ | 0.85 t (7.0) | 18, 19b |

[a]500.13 MHz for $^1H$ and 125.76 MHz for $^{13}C$; [b]Multiplicity and assignment from HSQC experiment; [c]Determined from HMBC experiment; [d] $^1J$ = 250.0 Hz; [e] $^2J$ = 48.4 Hz; [f]Exact $^{13}C$ chemical shifts 29.10, 29.26, 29.38 , 29.59 (× 6), 29.50 ppm.

Figure S114. EIGCMS spectrum of (3*R*)-icos-(4*E*)-en-1-yn-3-ol (**18**)

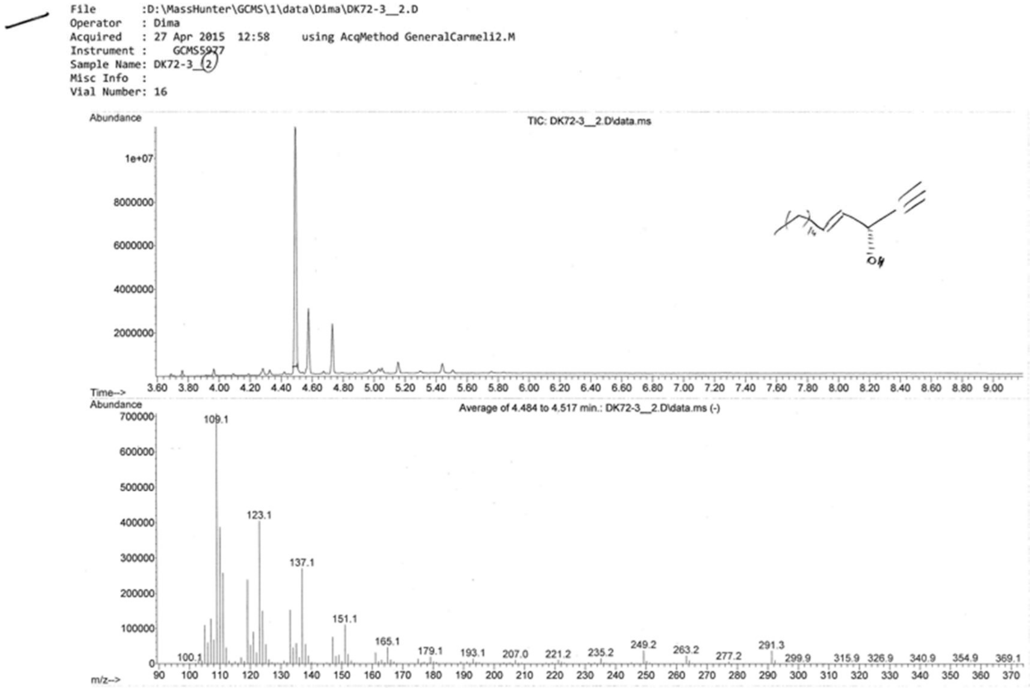


Figure S115. HRCIMS spectrum of (3*R*)-icos-(4*E*)-en-1-yn-3-ol (**18**)

## Compound Spectrum SmartFormula Report

**Analysis Info**

Acquisition Date 5/17/2015 11:35:58 AM

Analysis Name D:\Data\Client\CLI_9279_12.d
Method CLI_9279.m
Sample Name DK_72-3(2)
Comment

Operator Larisa Panz
Instrument maXis impact 282001.00128

**Acquisition Parameter**

| | | | | | |
|---|---|---|---|---|---|
| Source Type | APCI | Ion Polarity | Positive | Set Nebulizer | 4.0 Bar |
| Focus | Active | Set Capillary | 4000 V | Set Dry Heater | 200 °C |
| Scan Begin | 50 m/z | Set End Plate Offset | -500 V | Set Dry Gas | 2.0 l/min |
| Scan End | 1000 m/z | Set Charging Voltage | 2000 V | Set Divert Valve | Source |
| | | Set Corona | 4000 nA | Set APCI Heater | 450 °C |

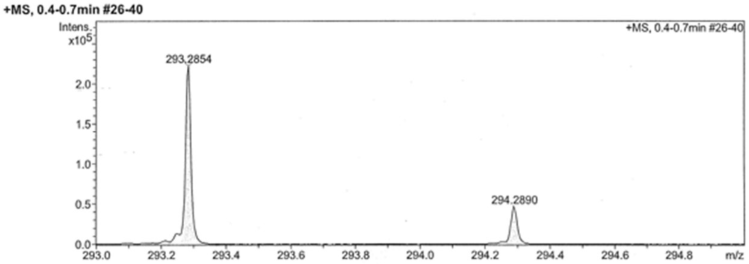


| Meas. m/z | # | Ion Formula | m/z | err [ppm] | mSigma | # mSigma | Score | rdb | e⁻ Conf | N-Rule | \|err\| [mDa] |
|---|---|---|---|---|---|---|---|---|---|---|---|
| 293.2854 | 1 | C20H37O | 293.2839 | -5.2 | 145.6 | 1 | 100.00 | 2.5 | even | ok | 1.5 |

Figure S116. $^{1}$H NMR spectrum of (3*R*)-19-methylicos-(4*E*)-en-1-yn-3-ol (**19**) in $CDCl_3$

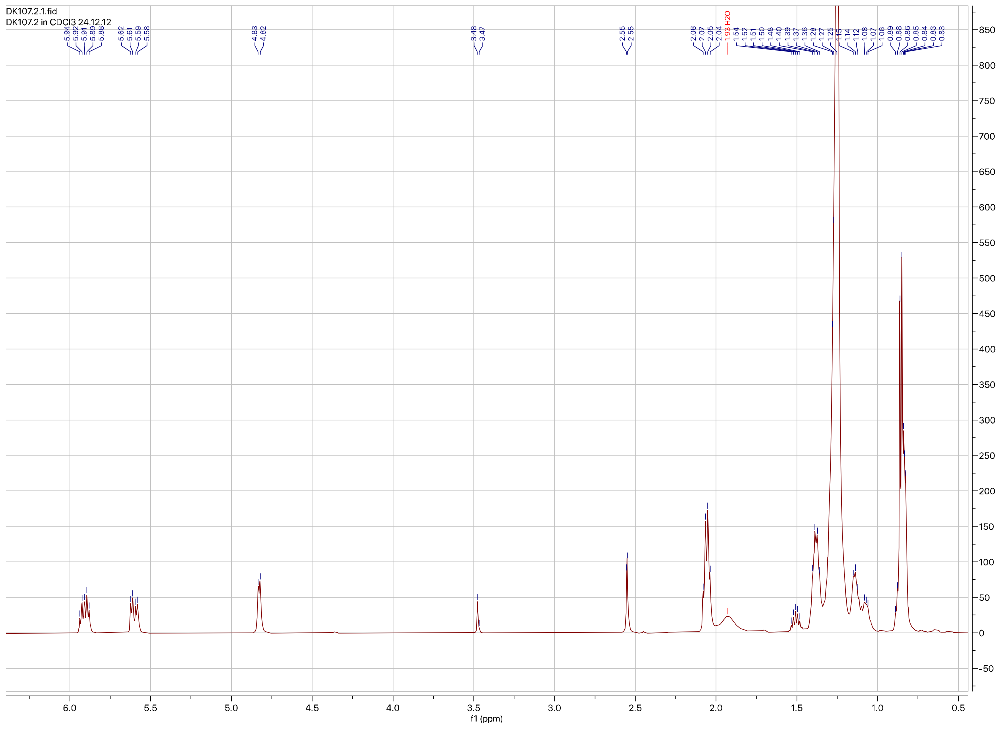


Figure S117. $^{13}$C NMR spectrum of (3*R*)-19-methylicos-(4*E*)-en-1-yn-3-ol (**19**) in $CDCl_3$

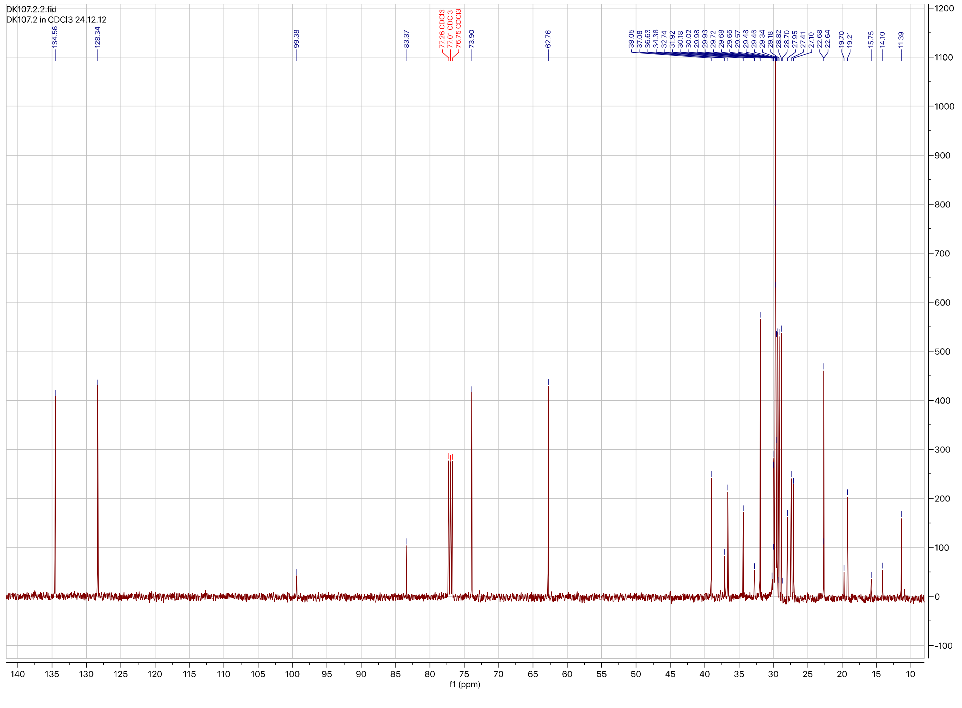

Table S19. NMR data of (3*R*)-19-methylicos-(4*E*)-en-1-yn-3-ol (**19**) in $CDCl_3$.[a]

| Position | $\delta_C$, mult.[b] | $\delta_H$, mult. *J* (Hz) | LR H-C Correlations[c] |
|---|---|---|---|
| 1 | 73.9[d] CH | 2.55 d (2.0) | 3 |
| 2 | 83.4[e] qC | - | 1, 3, 4 |
| 3 | 62.8 CH | 4.82 d (6.0) | 1, 4, 5 |
| 4 | 128.3 CH | 5.59 dd (15.0, 6.0) | 3, 6 |
| 5 | 134.6 CH | 5.90 dt (15.0, 7.0) | 3, 6, 7 |
| 6 | 31.9 $CH_2$ | 2.05 q (7.0) | 4, 5, 7, 8 |
| 7 | 28.8 $CH_2$ | 1.38 m | 5, 6, 8 |
| 8-16 | ~29.6[f] 9 × $CH_2$ | 1.22 – 1.31 brm | |
| 17 | 27.4 $CH_2$ | 1.24 m | 16, 18 |
| 18 | 39.0 $CH_2$ | 1.14 m | 17, 19, 20, 21 |
| 19 | 27.9 CH | 1.50 qqt (6.0, 6.0, 6.0) | 18, 20, 21 |
| 20 | 22.6 $CH_3$ | 0.85 d (6.0) | 18, 19, 21 |
| 21 | 22.6 $CH_3$ | 0.85 d (6.0) | 18, 19, 20 |

[a]500.13 MHz for $^1H$ and 125.76 MHz for $^{13}C$; [b]Multiplicity and assignment from HSQC experiment; [c]Determined from HMBC experiment; [d] $^1J$ = 250.0 Hz; [e] $^2J$ = 49.0 Hz; [f]Exact $^{13}C$ chemical shifts 29.45, 29.57, 29.67 (× 6), 29.71 ppm.

Figure S118. HRCIMS spectrum of (3*R*)-19-methylicos-(4*E*)-en-1-yn-3-ol (**19**)

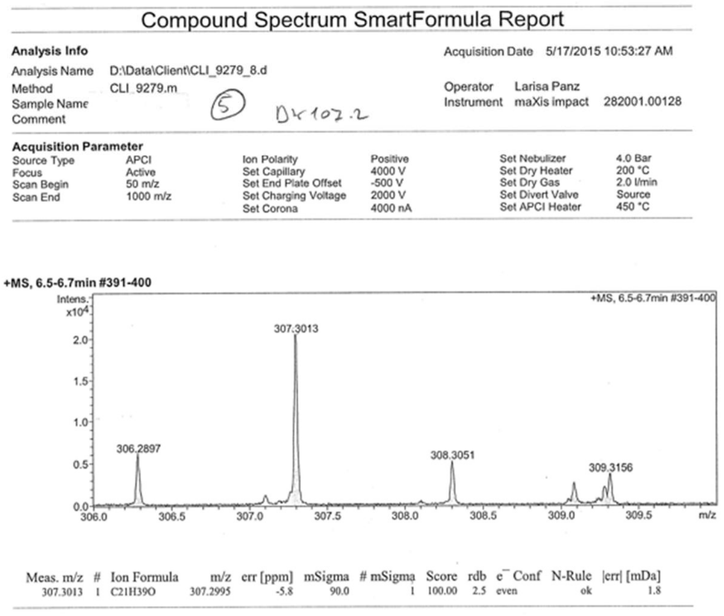

Figure S119. $^{1}H$ NMR spectrum of (3*R*)-henicos-(4*E*)-en-1-yn-3-ol (**20**) in $CDCl_3$

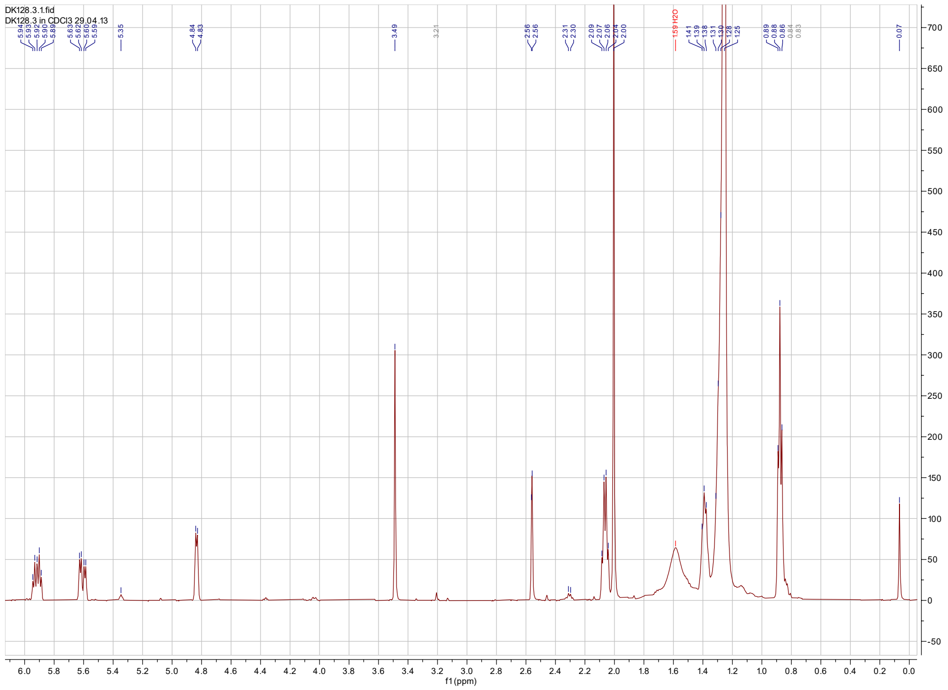


Table S20. NMR data of (3*R*)-henicos-(4*E*)-en-1-yn-3-ol (**20**) in $CDCl_3$.[a]

| Position | $\delta_H$, mult. *J* (Hz) | $\delta_H$, mult. *J* (Hz) published[b] |
|---|---|---|
| 1 | 2.56 s | 2.54 d 12.0) |
| 2 | - | - |
| 3 | 4.83 d (6.0) | 4.82 d (5.5) |
| 4 | 5.61 dd (15.0, 6.0) | 5.58 ddt (15.0, 5.5, 1.0) |
| 5 | 5.90 dt (15.0, 7.0) | 5.90 dtd (15.0, 7.0, 1.0) |
| 6 | 2.07 q (7.0) | 2.04 q (7.0) |
| 7 | 1.39 m | 1.23 - 1.36 m |
| 8-18 | 1.23 – 1.30 brm | 1.23 – 1.36 m |
| 19 | 1.25 m | 1.23 – 1.36 m |
| 20a | 1.39 m | 1.23 – 1.36 m |
| b | 1.28 m | 1.23 – 1.36 m |
| 21 | 0.88 t (6.5) | 0.86 t (7.0) |

[a]500.13 MHz for $^{1}H$ and 125.76 MHz for $^{13}C$; [b]Hallock. Y. F.; Cardelina II, J. H.; Balaschak, M. S.; Alexander, M. R.; Prather, T.R.; Shoemaker, R. H.; Boyd, M. R. *J. Nat. Prod.* **1995**, *58*, 1801-1807.

Figure S120. CIGCMS spectrum of (3*R*)-henicos-(4*E*)-en-1-yn-3-ol (**20**)

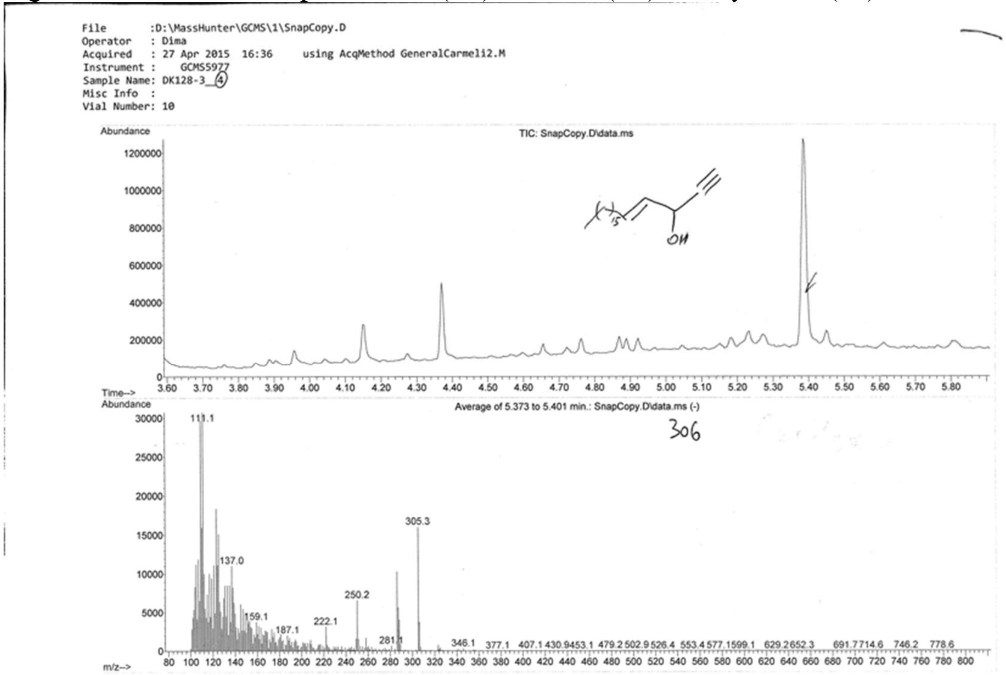


Figure S121. HRCIMS of (3*R*)-henicos-(4*E*)-en-1-yn-3-ol (**20**)

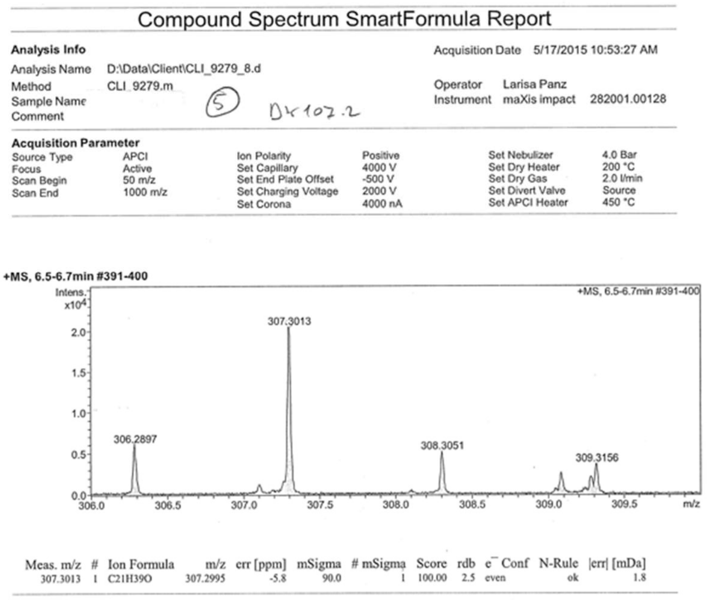

Figure S122. $^{1}$H NMR spectrum of (3*R*)-docos-(4*E*,15*Z*)-dien-1-yn-3-ol (**21**) in $CDCl_3$

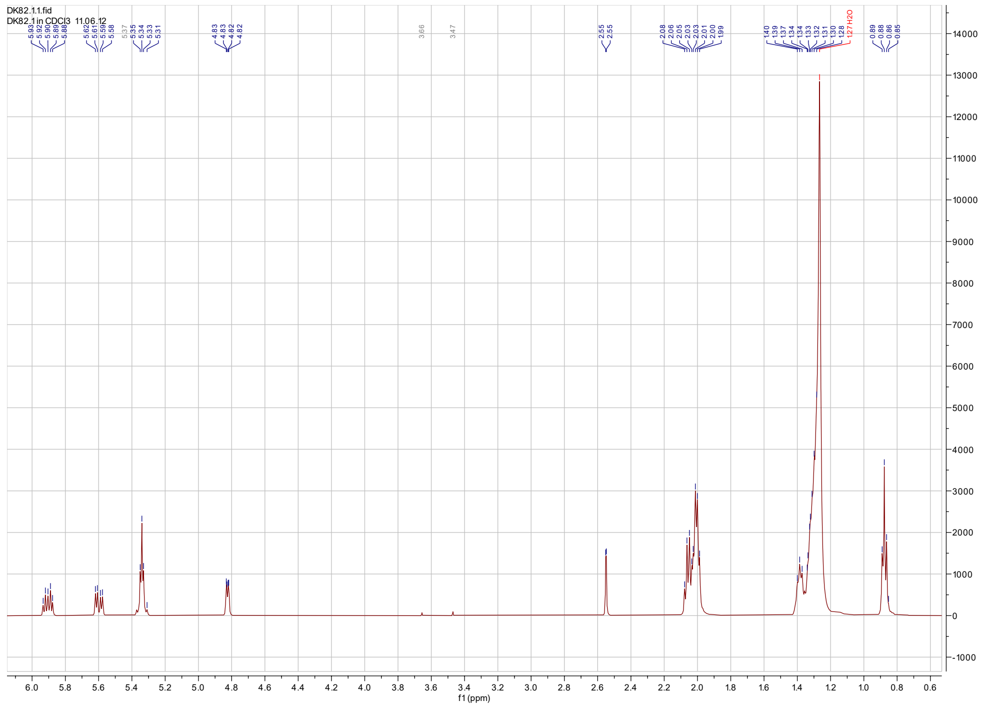

Figure S123. $^{13}$C NMR spectrum of (3*R*)-docos-(4*E*,15*Z*)-dien-1-yn-3-ol (**21**) in $CDCl_3$

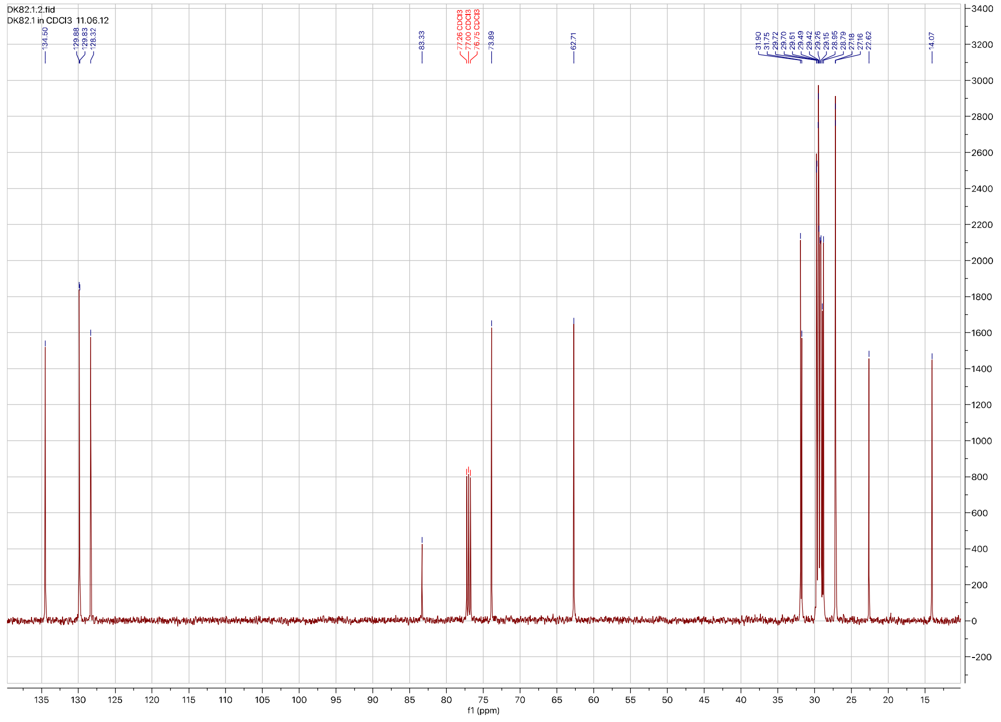

Table S21. NMR data of (3*R*)-docos-(4*E*,15*Z*)-dien-1-yn-3-ol (**21**) in $CDCl_3$.[a]

| Position | $\delta_C$, mult.[b] | $\delta_H$, mult. *J* (Hz) | LR H-C Correlations[c] |
|---|---|---|---|
| 1 | 73.9[d] CH | 2.55 d (2.5) | 3 |
| 2 | 83.3[e] qC | - | 1, 3, 4 |
| 3 | 62.7 CH | 4.82 brd (6.0) | 1, 4, 5 |
| 4 | 128.3 CH | 5.60 dd (15.0, 6.0) | 3, 6 |
| 5 | 134.5 CH | 5.91 dt (15.0, 7.5) | 3, 6, 7 |
| 6 | 31.9 $CH_2$ | 2.05 q (7.5) | 4, 5, 7, 8 |
| 7 | 28.8 $CH_2$ | 1.38 m | 5, 6, 8 |
| 8-13 | ~29.6[f] 6 × $CH_2$ | 1.22 – 1.30 brm | |
| 14 | 27.2 $CH_2$ | 2.01 q (5.5) | 13, 15 |
| 15 | 129.8 CH | 5.34 t (5.5) | 13, 14 |
| 16 | 129.9 CH | 5.34 t (5.5) | 17, 18 |
| 17 | 27.2 $CH_2$ | 2.01 m | 16, 18 |
| 18-19 | 29.6[f] 2 × $CH_2$ | 1.22 – 1.30 brm | |
| 20 | 31.7 $CH_2$ | 1.25 m | 19, 21a, 21b, 22 |
| 21a | 22.6 $CH_2$ | 1.32 m | 22, 24 |
| b | | 1.27 m | |
| 22 | 14.1 $CH_3$ | 0.88 t (6.7) | 20, 21a, 21b |

[a]500.13 MHz for $^1$H and 125.76 MHz for $^{13}$C; [b]Multiplicity and assignment from HSQC experiment; [c]Determined from HMBC experiment; [d] $^1J$ = 252.0 Hz; [e] $^2J$ = 49.0 Hz; [f]Exact $^{13}$C chemical shifts 28.95, 29.15, 29.25, 29.42, 29.49 (× 2), 29.70, 29.72 ppm.

Figure S124. EIGCMS spectrum of (3*R*)-docos-(4*E*,15*Z*)-dien-1-yn-3-ol (**21**)

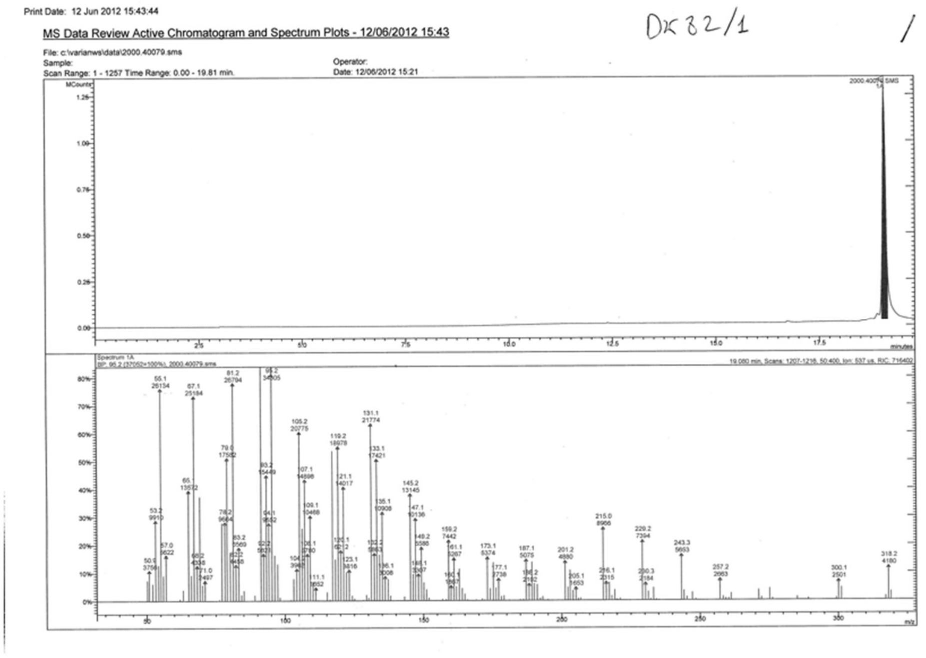

Figure S125. $^{1}H$ NMR spectrum of (3*R*)-21-methyldocos-(4*E*,15*Z*)-dien-1-yn-3-ol (**22**) in $CDCl_3$

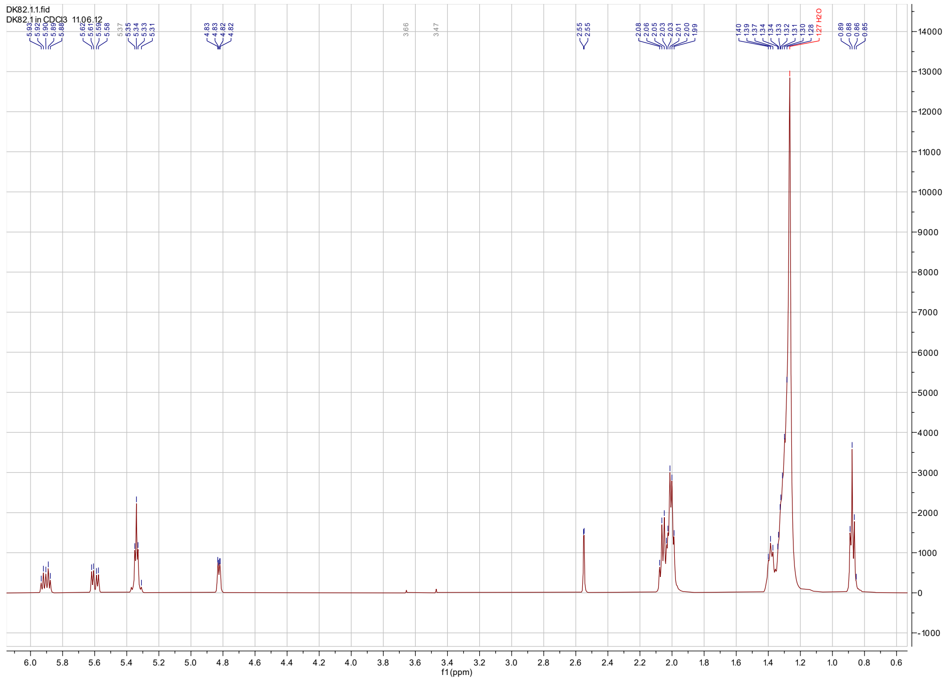


Figure S126. $^{13}C$ NMR spectrum of (3*R*)-21-methyldocos-(4*E*,15*Z*)-dien-1-yn-3-ol (**22**) in $CDCl_3$

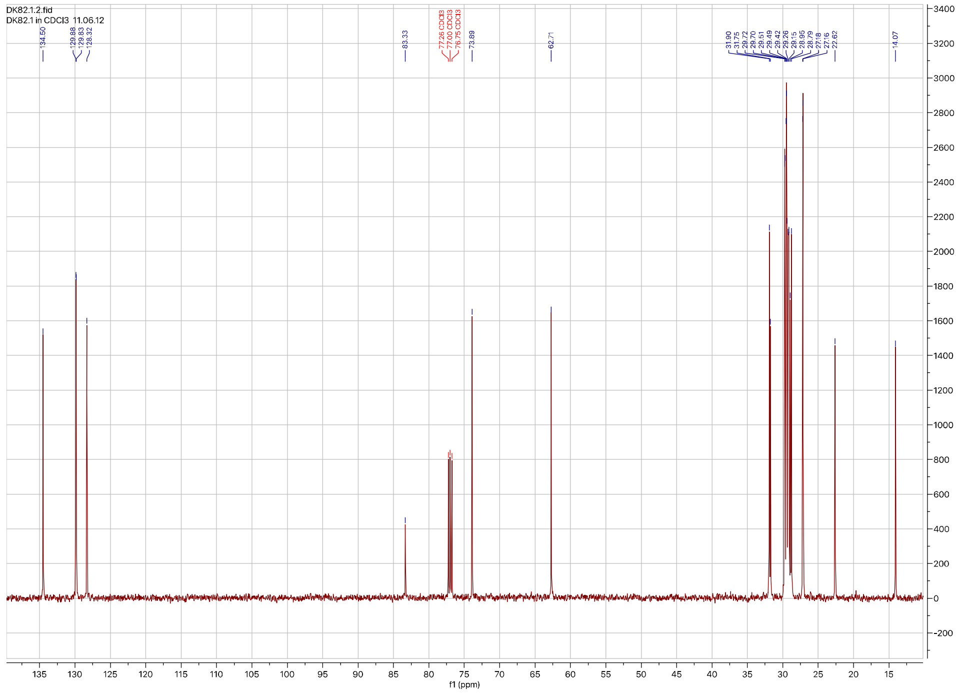

Table S22. NMR data of (3*R*)-21-methyldocos-(4*E*,15*Z*)-dien-1-yn-3-ol (**22**) in $CDCl_3$.[a]

| Position | $\delta_C$, mult.[b] | $\delta_H$, mult. *J* (Hz) | LR H-C Correlations[c] |
|---|---|---|---|
| 1 | 73.9[d] CH | 2.56 s | 3 |
| 2 | 83.3[e] qC | - | 1, 3, 4 |
| 3 | 62.8 CH | 4.83 brd (6.0) | 1, 4, 5 |
| 4 | 128.3 CH | 5.61 dd (15.0, 6.0) | 3, 6 |
| 5 | 134.6 CH | 5.91 dt (15.0, 7.0) | 3, 6, 7 |
| 6 | 31.9 $CH_2$ | 2.06 q (7.0) | 4, 5, 7, 8 |
| 7 | 28.8 $CH_2$ | 1.39 m | 5, 6, 8 |
| 8-13 | ~29.6[f] 6 × $CH_2$ | 1.24 – 1.32 brm | |
| 14 | 27.2 $CH_2$ | 2.01 m | 13, 15 |
| 15 | 129.9 CH | 5.34 t (4.0) | 13, 14 |
| 16 | 129.9 CH | 5.34 t (4.0) | 17, 18 |
| 17 | 27.2 $CH_2$ | 2.01 m | 16, 18 |
| 18 | 30.0 $CH_2$ | 1.31 m | |
| 19 | 27.2 $CH_2$ | 1.28 m | 18,20 |
| 20 | 39.3 $CH_2$ | 1.17 m | 19, 21, 22, 23 |
| 21 | 28.0 CH | | 20, 22, 23 |
| 22 | 22.6 $CH_3$ | 0.85 d (6.5) | 20, 21, 23 |
| 23 | 22.6 $CH_3$ | 0.85 d (6.5) | 20, 21, 22 |

[a]500.13 MHz for $^1H$ and 125.76 MHz for $^{13}C$; [b]Multiplicity and assignment from HSQC experiment; [c]Determined from HMBC experiment; [d] $^1J$ = 251.0 Hz; [e] $^2J$ = 49.5 Hz; [f]Exact $^{13}C$ chemical shifts 29.17, 29.29, 29.44, 29.53 (× 2), 29.73 ppm.

Figure S127. EIMS spectrum of (3*R*)-21-methyldocos-(4*E*,15*Z*)-dien-1-yn-3-ol (**22**)

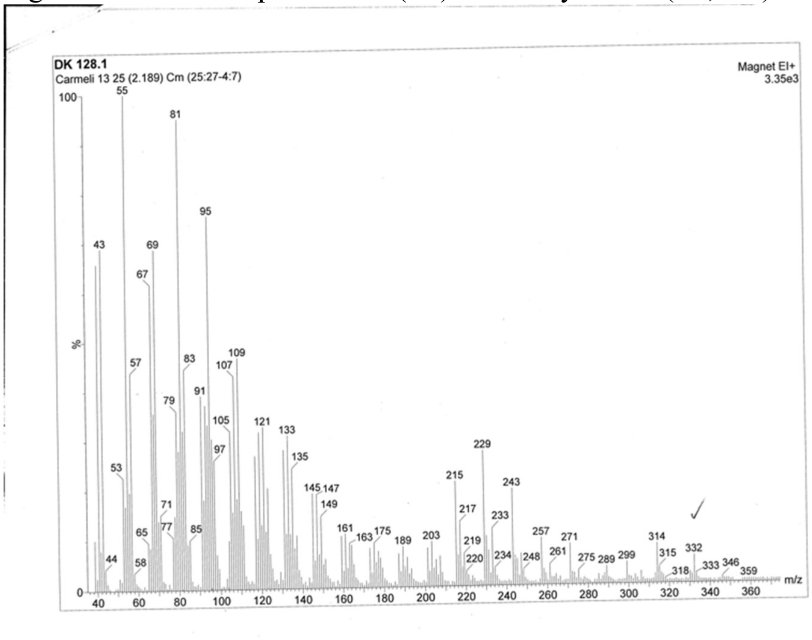

Figure S128. $^{1}$H NMR spectrum of (3*R*)-14-methyldocos-1-yn-3-ol (**23**) in $CDCl_3$

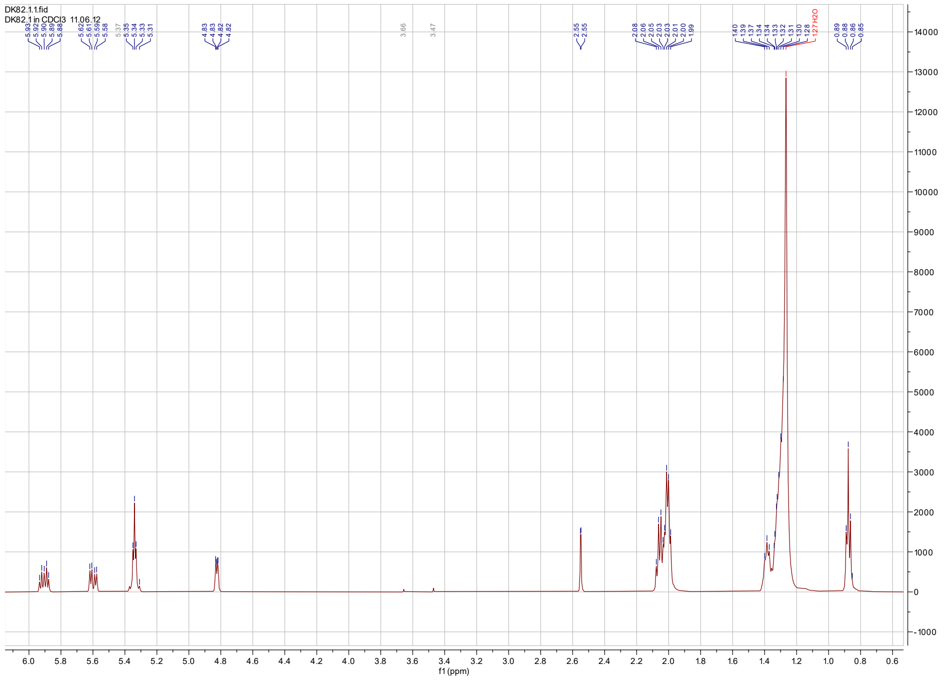

Figure S129. $^{13}$C NMR spectrum of (3*R*)-14-methyldocos-1-yn-3-ol (**23**) in $CDCl_3$

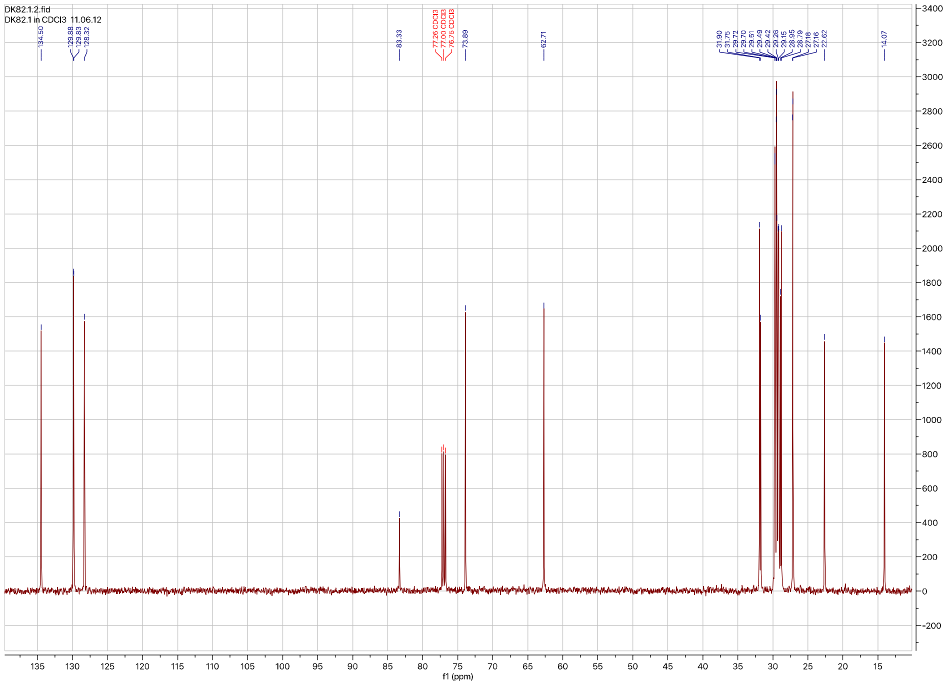

Table S23. NMR data of (3*R*)-14-methyldocos-1-yn-3-ol (**23**) in $CDCl_3$.[a]

| Position | $\delta_C$, mult.[b] | $\delta_H$, mult. *J* (Hz) | LR H-C Correlations[c] |
|---|---|---|---|
| 1 | 73.9[d] CH | 2.55 d (2.0) | 3 |
| 2 | 83.4[e] qC | - | 1, 3, 4 |
| 3 | 62.8 CH | 4.82 brd (5.5) | 1, 4, 5 |
| 4 | 128.3 CH | 5.60 dd (15.3, 6.0) | 3, 6 |
| 5 | 134.5 CH | 5.90 dt (15.3, 7.0) | 3, 6, 7 |
| 6 | 31.9 $CH_2$ | 2.06 q (7.0) | 4, 5, 7, 8 |
| 7 | 28.8 $CH_2$ | 1.38 m | 5, 6, 8 |
| 8-11 | ~29.5[f] 4 × $CH_2$ | 1.23 – 1.26 brm | |
| 12 | 27.1 $CH_2$ | 1.24 m | 11, 13a, 13b |
| 13a | 37.1 $CH_2$ | 1.26 m | 12, 14, 15a, 15b, 23 |
| b | | 1.06 m | |
| 14 | 32.7 CH | 1.34 m | 13a, 13b, 15a, 15a, 23 |
| 15a | 37.1 $CH_2$ | 1.26 m | 13a, 13b, 14, 16, 23 |
| b | | 1.06 m | |
| 16 | 27.0 $CH_2$ | 1.23 m | 15a, 15b, 17 |
| 17-19 | ~29.5[f] 3 × $CH_2$ | 1.23 – 1.26 brm | |
| 20 | 31.9 $CH_2$ | 1.24 m | 19, 21a, 21b, 22 |
| 21a | 22.7 $CH_2$ | 1.31 m | 20, 22 |
| b | | 1.25 m | |
| 22 | 14.1 $CH_3$ | 0.87 t (6.7) | 21a, 21b |
| 23 | 19.7 $CH_3$ | 0.83 d (6.5) | 13b, 15b |

[a]500.13 MHz for $^1H$ and 125.76 MHz for $^{13}C$; [b]Multiplicity and assignment from HSQC experiment; [c]Determined from HMBC experiment; [d] $^1J$ = 250.5 Hz; [e] $^2J$ = 48.3 Hz; [f]Exact $^{13}C$ chemical shifts 29.17, 29.34, 29.46, 29.60, 29.67, 29.98, 30.00 ppm.

Figure S130. EIMS of (3*R*)-14-methyldocos-1-yn-3-ol (**23**)

File :C:\MSDCHEM\1\DATA\SMB DATA 7_11\Snapshot\AVIV884.D
Operator :
Acquired : 15 Mar 2012 11:34 using AcqMethod SMB GC-MS.M
Instrument : GC-MSD
Sample Name: DK72/10
Misc Info : DK72/10
Vial Number: 1

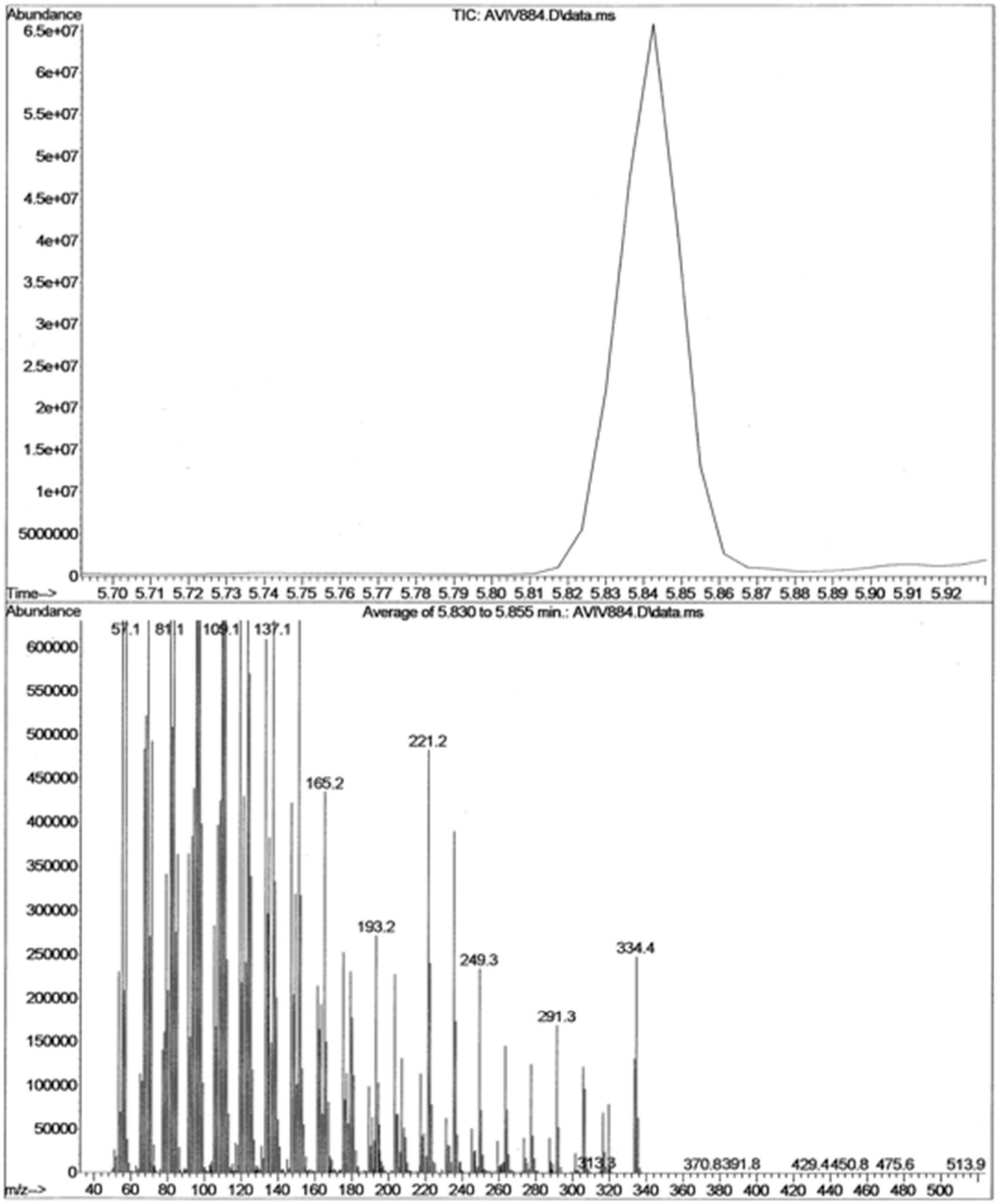

Figure S131. $^{1}$H NMR spectrum of (4*E*,6*E*)-docosa-4,6-dien-1-yn-3-ol (***rac*-27**) in $CDCl_3$

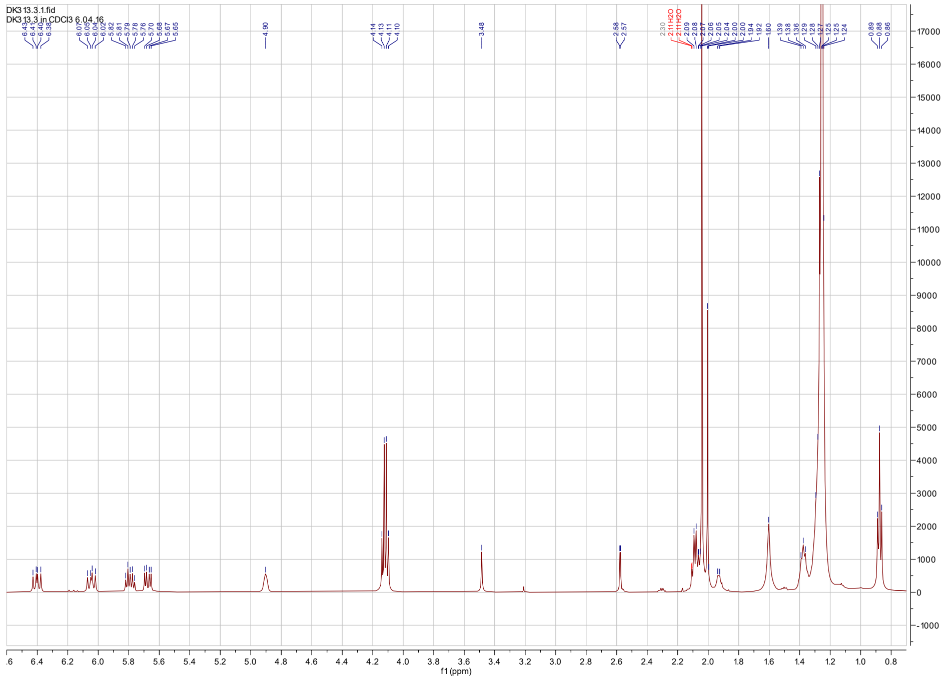


Figure S132. $^{13}$C NMR spectrum of (4*E*,6*E*)-docosa-4,6-dien-1-yn-3-ol (***rac*-27**) in $CDCl_3$

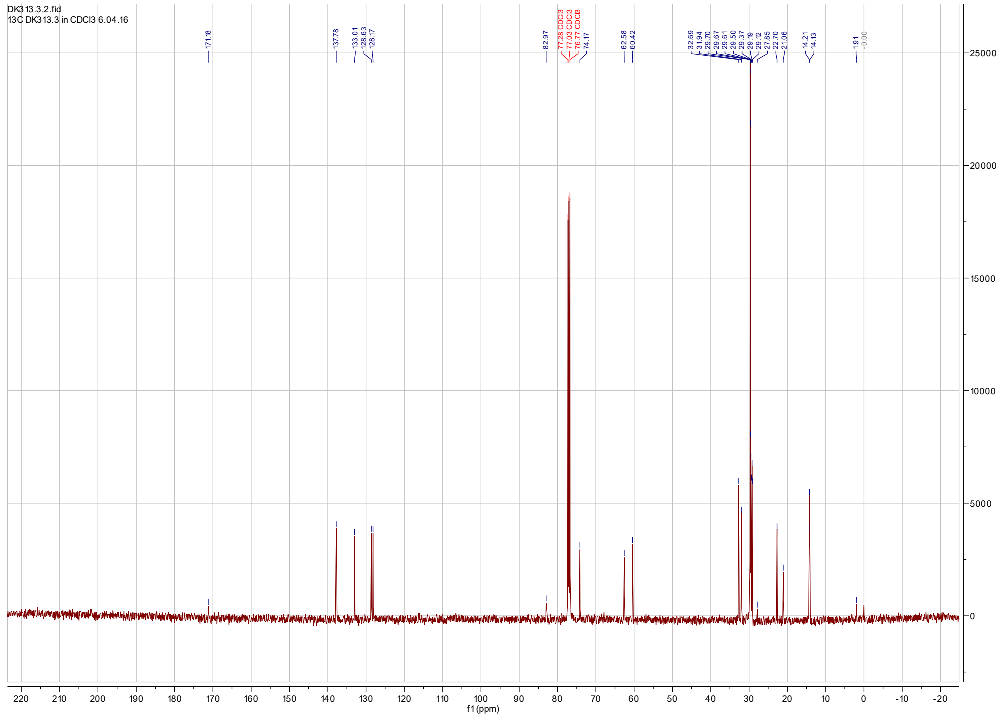

Figure S133. HREIMS of (4*E*,6*E*)-docosa-4,6-dien-1-yn-3-ol (***rac*-27**)

**Elemental Composition Report** **Page 1**

**Single Mass Analysis**
Tolerance = 100.0 PPM / DBE: min = -1.5, max = 50.0
Selected filters: None

Monoisotopic Mass, Odd and Even Electron Ions
24 formula(e) evaluated with 3 results within limits (all results (up to 1000) for each mass)
Elements Used:
C: 15-30 H: 0-50 O: 0-5

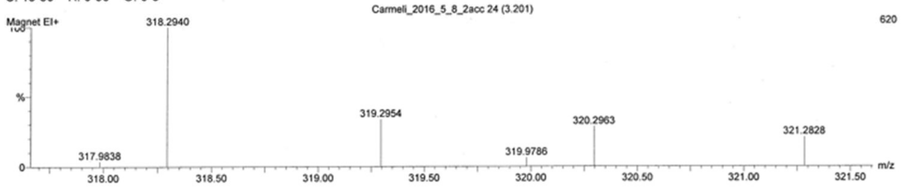


| | | | | | | |
|---|---|---|---|---|---|---|
| Minimum: | | | | -1.5 | | |
| Maximum: | | 5.0 | 100.0 | 50.0 | | |
| Mass | Calc. Mass | mDa | PPM | DBE | i-FIT | Formula |
| 318.2940 | 318.2923 | 1.7 | 5.3 | 4.0 | 72.9 | C22 H38 O |
| | 318.2770 | 17.0 | 53.4 | 0.0 | 89.5 | C18 H38 O4 |
| | 318.3134 | -19.4 | -60.9 | -1.0 | 91.5 | C19 H42 O3 |

Figure S134. $^{1}$H NMR spectrum of (*S*)-((*R*)-icos-(4*E*)-en-1-yn-3-yl-3,3,3-trifluoro-2-methoxy-2-phenylpropanoate (***(S,R)*-29**) in $CDCl_3$

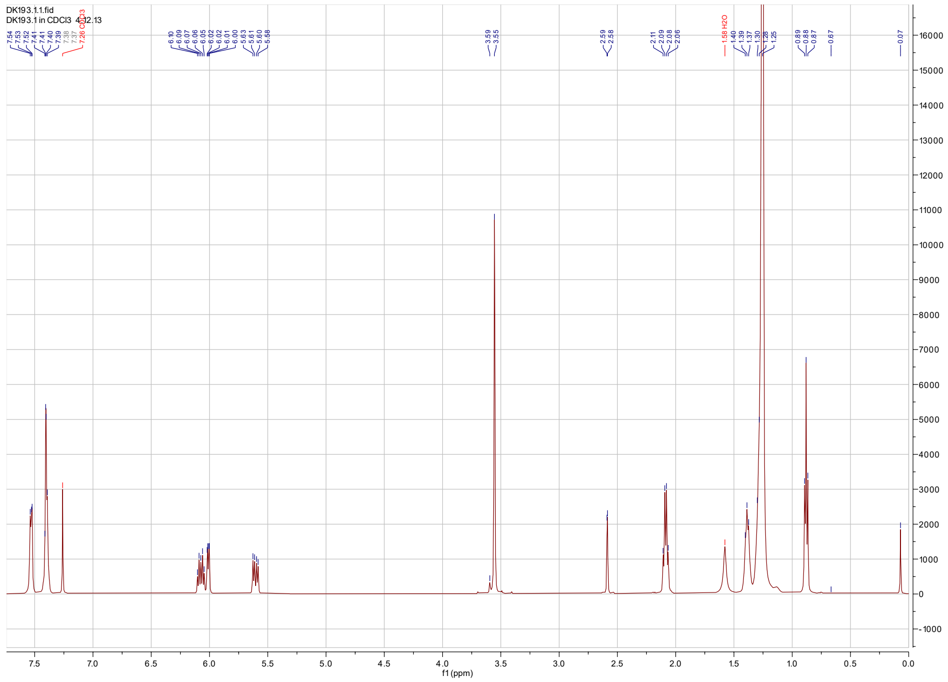


Figure S135. $^{1}$H NMR spectrum of (*S*)-((*S*)-icos-(4*E*)-en-1-yn-3-yl-3,3,3-trifluoro-2-methoxy-2-phenylpropanoate (***(S,S)*-29**) in $CDCl_3$

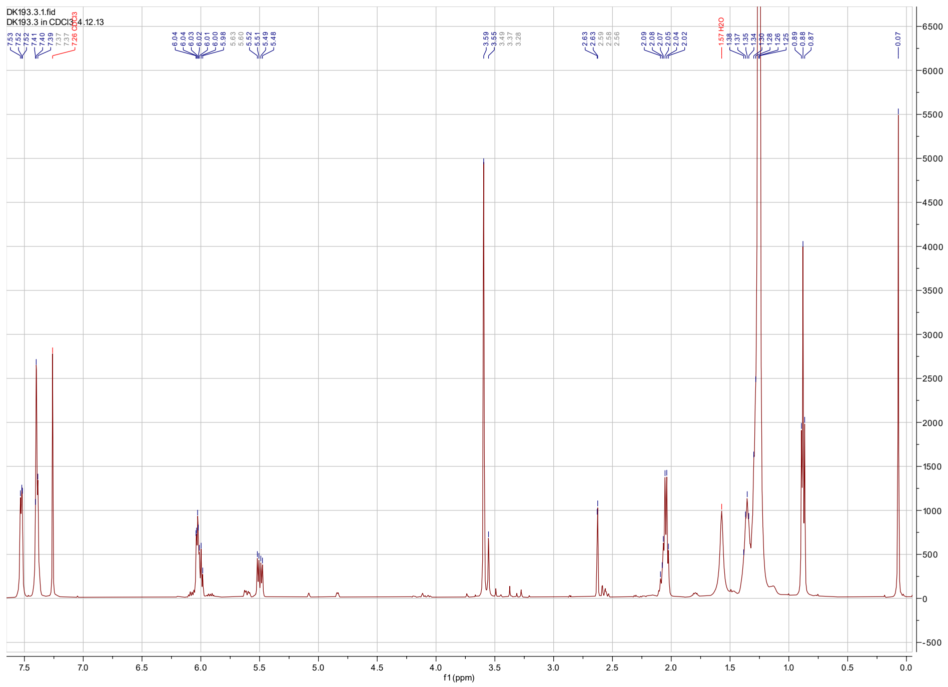

Figure S136. $^{1}$H NMR spectrum of dodec-1-yn-3-ol (***rac*-31**) in $CDCl_3$

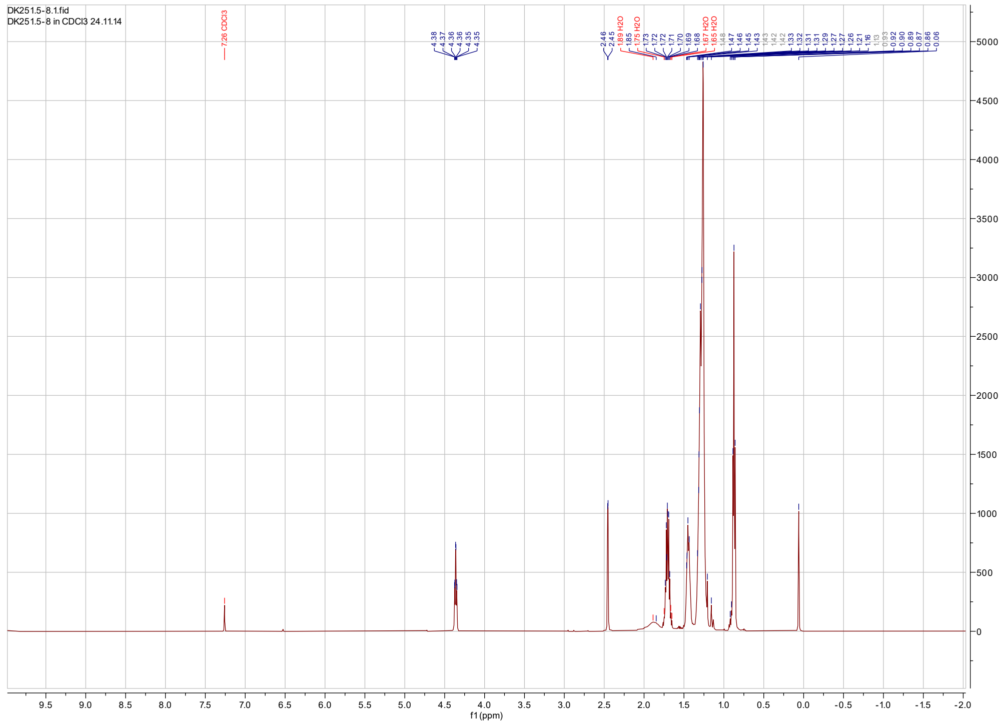

Figure S137. $^{13}$C NMR spectrum of dodec-1-yn-3-ol (***rac*-31**) in $CDCl_3$

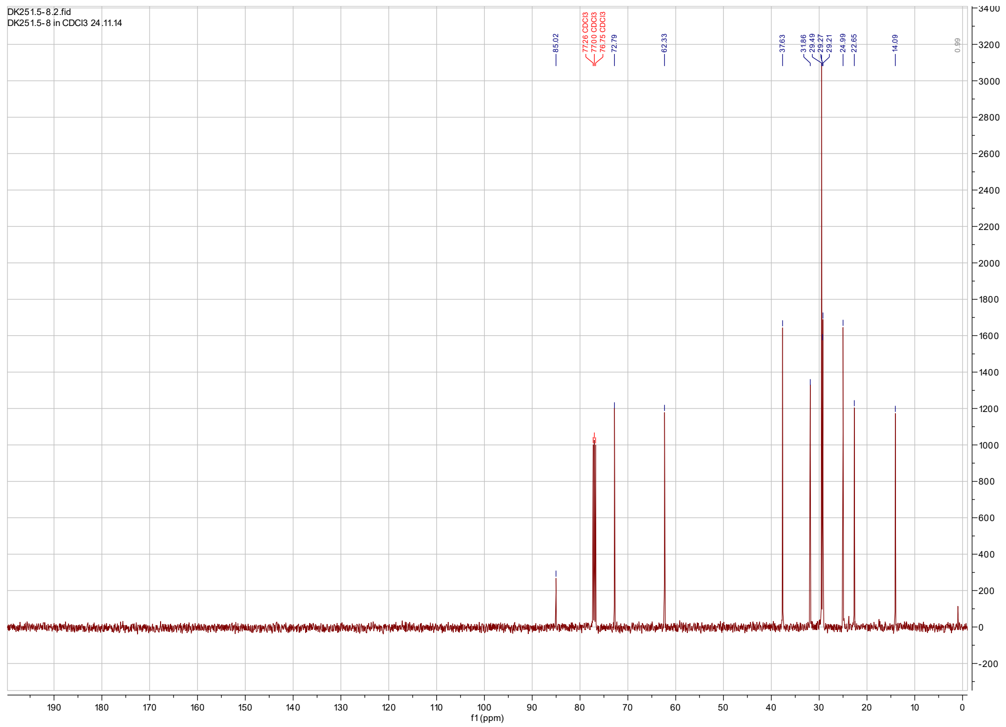

Figure S138. HRCIMS of dodec-1-yn-3-ol (***rac*-31**)

## Compound Spectrum SmartFormula Report

**Analysis Info**

Analysis Name D:\Data\Client\CLI_9279_18.d
Method CLI_9279.m
Sample Name DK_251-5-8
Comment

Acquisition Date 5/17/2015 12:06:46 PM
Operator Larisa Panz
Instrument maXis impact 282001.00128

**Acquisition Parameter**

| | | | | | |
|---|---|---|---|---|---|
| Source Type | APCI | Ion Polarity | Positive | Set Nebulizer | 4.0 Bar |
| Focus | Active | Set Capillary | 4000 V | Set Dry Heater | 200 °C |
| Scan Begin | 50 m/z | Set End Plate Offset | -500 V | Set Dry Gas | 2.0 l/min |
| Scan End | 1000 m/z | Set Charging Voltage | 2000 V | Set Divert Valve | Source |
| | | Set Corona | 4000 nA | Set APCI Heater | 450 °C |

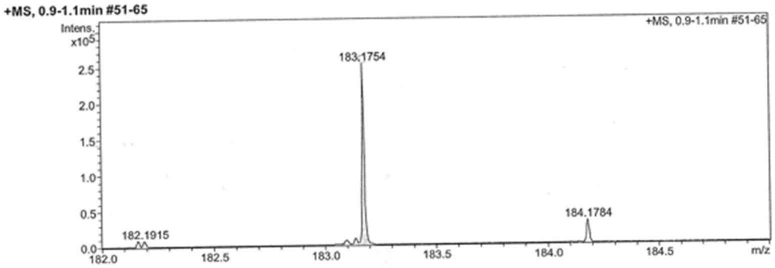


| Meas. m/z | # | Ion Formula | m/z | err [ppm] | mSigma | # mSigma | Score | rdb | e⁻ Conf | N-Rule | \|err\| [mDa] |
|---|---|---|---|---|---|---|---|---|---|---|---|
| 183.1754 | 1 | C12H23O | 183.1743 | -5.7 | 17.2 | 1 | 100.00 | 1.5 | even | ok | 1.1 |

Figure S139. $^{1}$H NMR spectrum of octadec-1-yn-3-ol (***rac*-32**) in $CDCl_3$

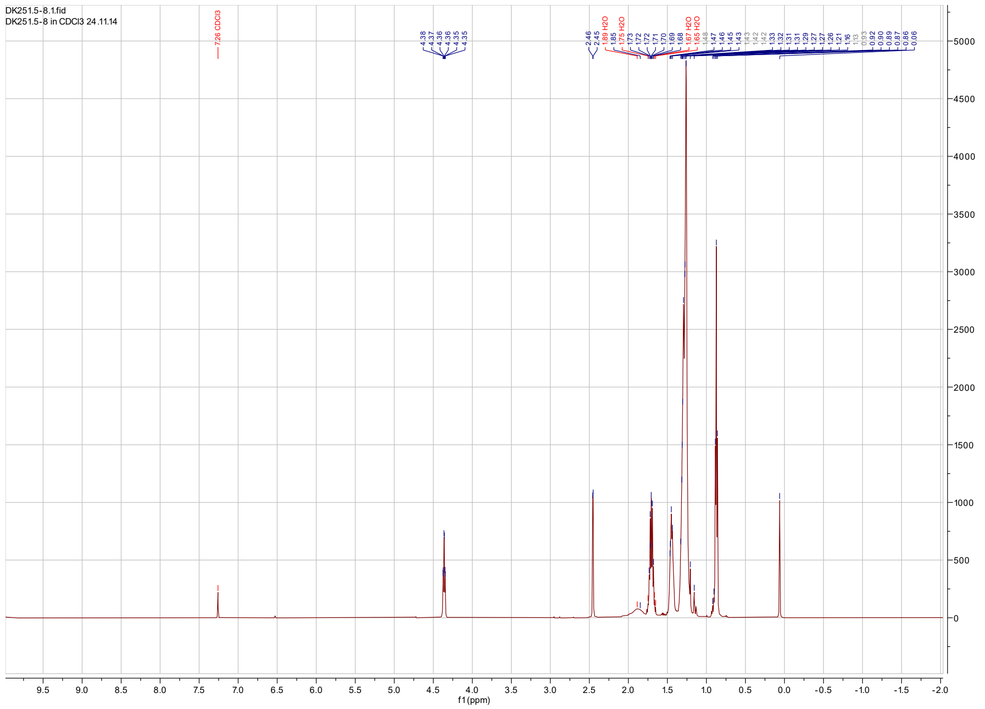


Figure S140. $^{13}$C NMR spectrum of octadec-1-yn-3-ol (***rac*-32**) in $CDCl_3$

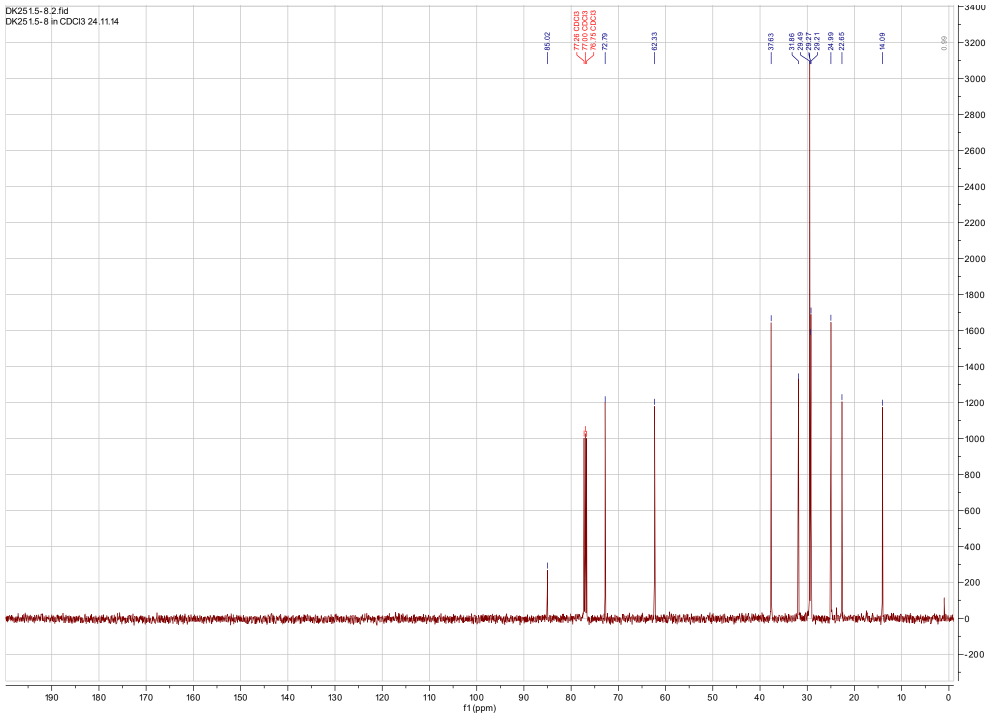

Figure S141. CIGCMS of octadec-1-yn-3-ol (***rac*-32**)

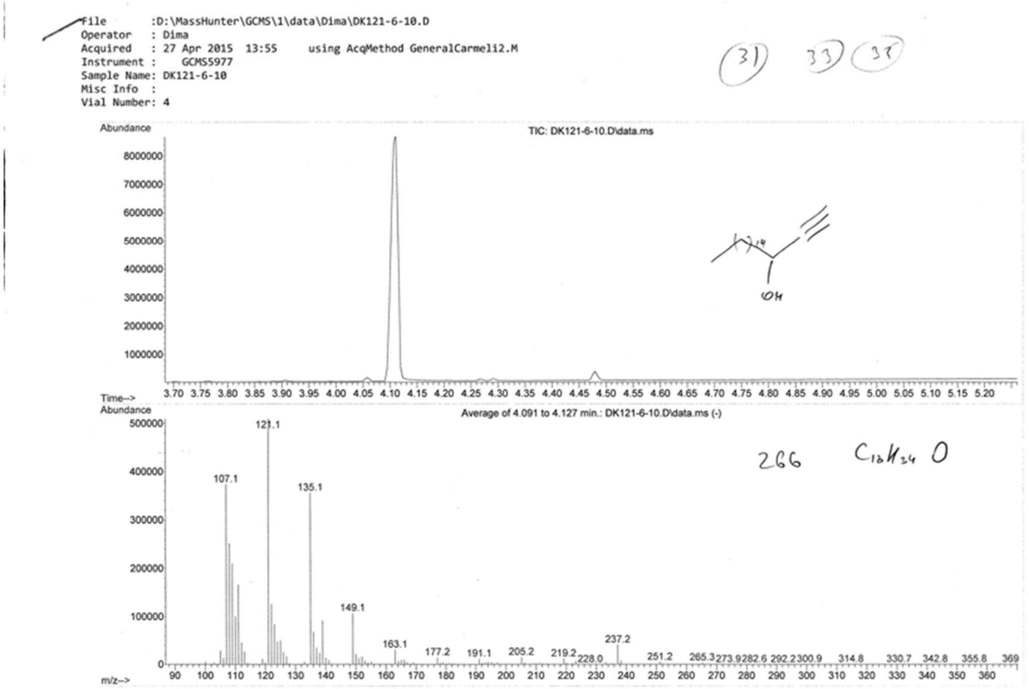


Figure S142. HRCIMS of octadec-1-yn-3-ol (***rac*-32**)

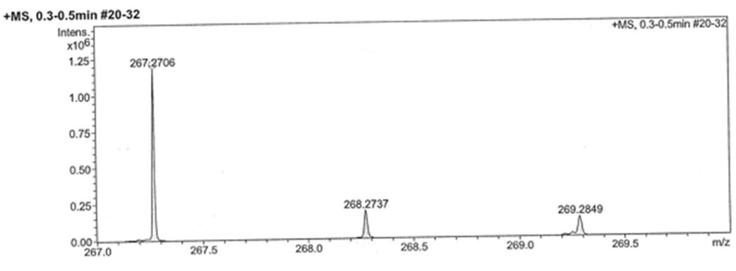
Compound Spectrum SmartFormula Report

**Analysis Info**

Acquisition Date 5/17/2015 11:43:22 AM

Analysis Name D:\Data\Client\CLI_9279_13.d
Method CLI_9279.m
Sample Name DK_121-6-10
Comment

Operator Larisa Panz
Instrument maXis impact 282001.00128

**Acquisition Parameter**

| | | | | | | | |
|---|---|---|---|---|---|---|---|
| Source Type | APCI | Ion Polarity | Positive | | | Set Nebulizer | 4.0 Bar |
| Focus | Active | Set Capillary | 4000 V | | | Set Dry Heater | 200 °C |
| Scan Begin | 50 m/z | Set End Plate Offset | -500 V | | | Set Dry Gas | 2.0 l/min |
| Scan End | 1000 m/z | Set Charging Voltage | 2000 V | | | Set Divert Valve | Source |
| | | Set Corona | 4000 nA | | | Set APCI Heater | 450 °C |



| Meas. m/z | # | Ion Formula | m/z | err [ppm] | mSigma | # mSigma | Score | rdb | e⁻ Conf | N-Rule | \|err\| [mDa] |
|---|---|---|---|---|---|---|---|---|---|---|---|
| 267.2706 | 1 | C18H35O | 267.2682 | -8.9 | 55.9 | 1 | 100.00 | 1.5 | even | ok | 2.4 |

Figure S143. $^{1}$H NMR spectrum of octadec-1-yn-3-ol (***R*-32**) in $CDCl_3$

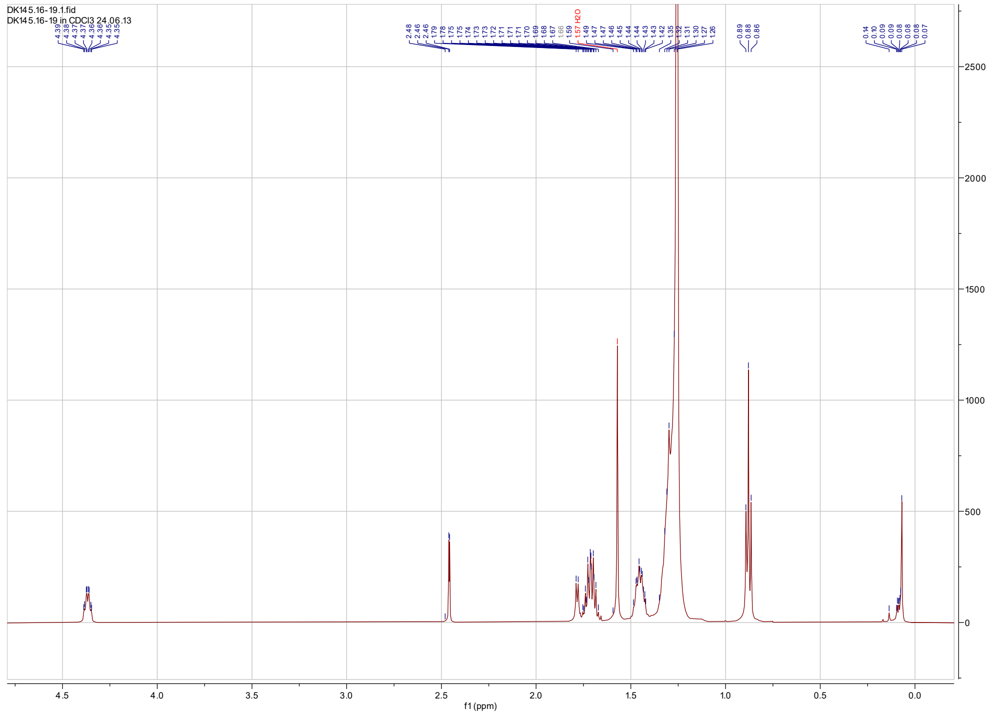


Figure S144. $^{1}$H NMR spectrum of octadec-1-yn-3-ol (***S*-32**) in $CDCl_3$

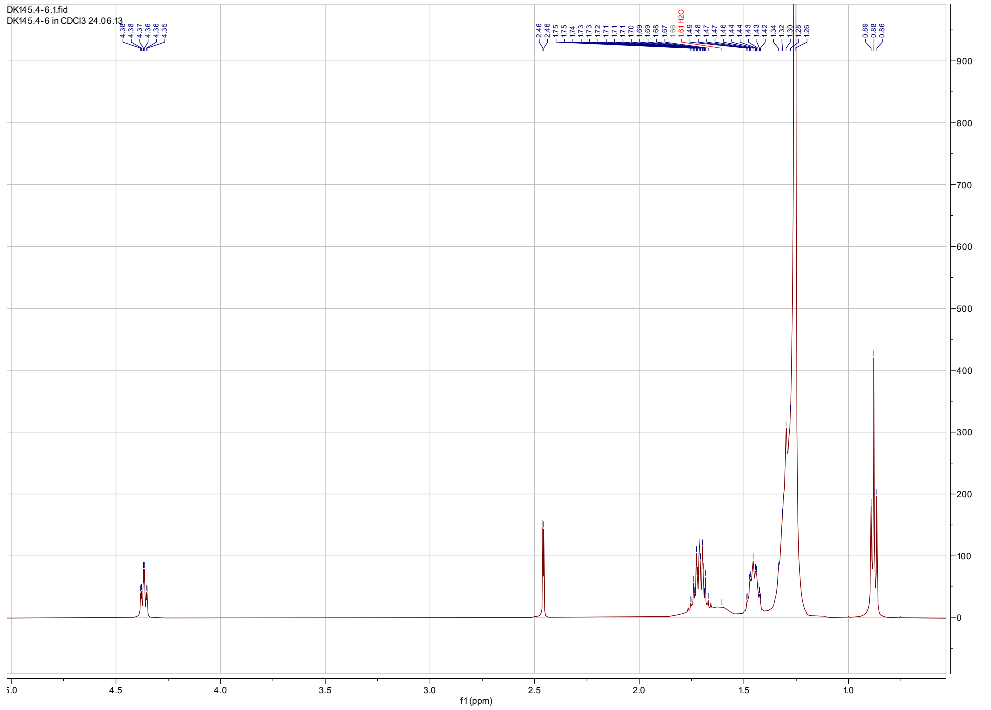

Figure S145. $^{1}H$ NMR spectrum of icos-1-yn-3-ol (***rac*-33**) in $CDCl_3$

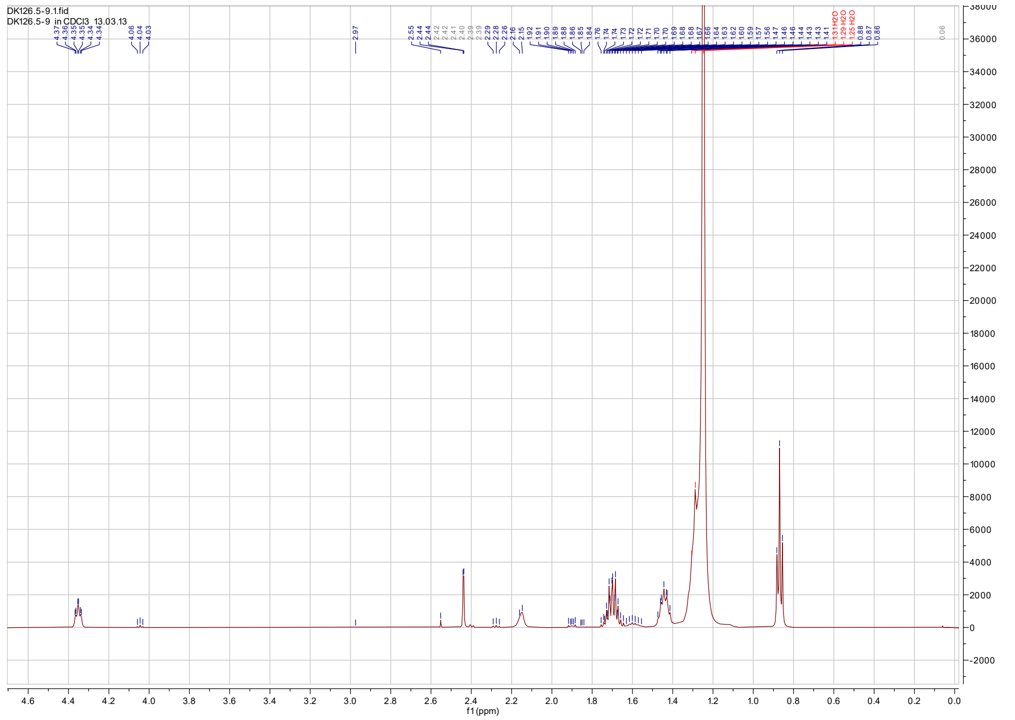


Figure S146. $^{13}C$ NMR spectrum of icos-1-yn-3-ol (***rac*-33**) in $CDCl_3$

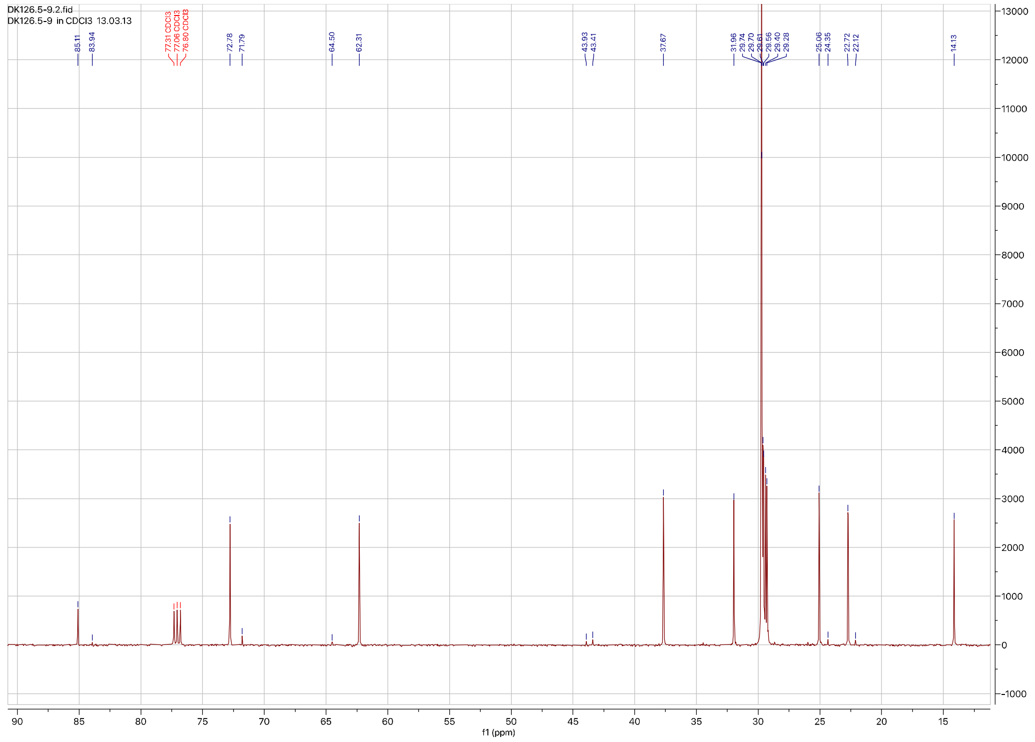

Figure S147. CIGCMS of icos-1-yn-3-ol (***rac*-33**)

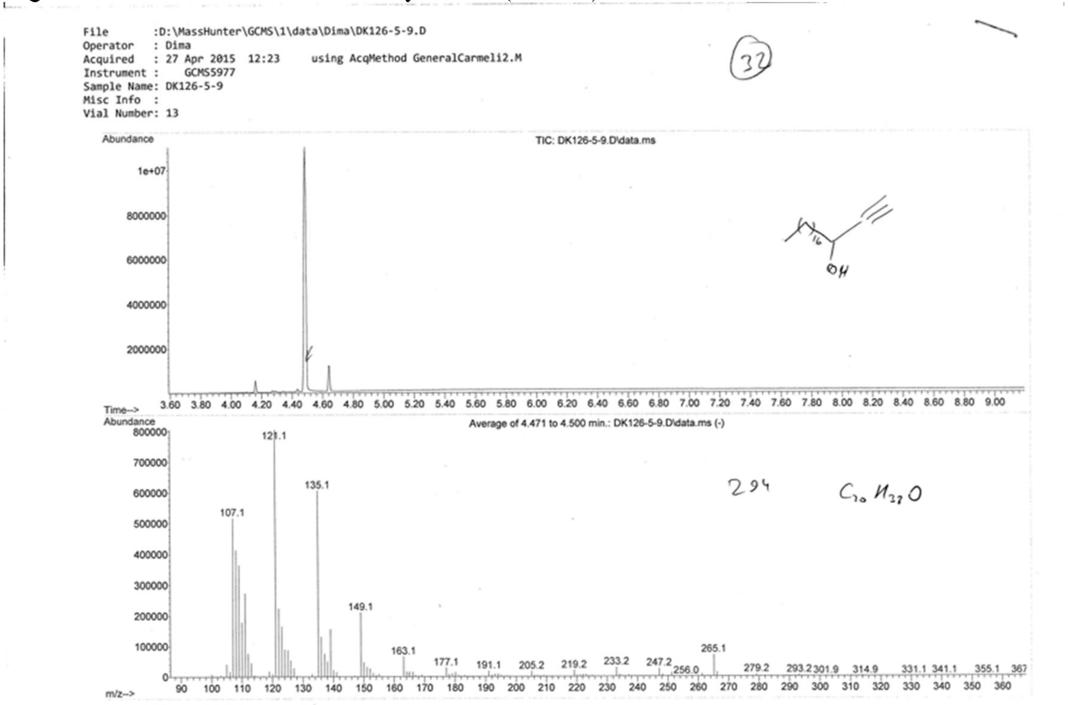


Figure S148. HRCIMS of icosc-1-yn-3-ol (***rac*-33**)

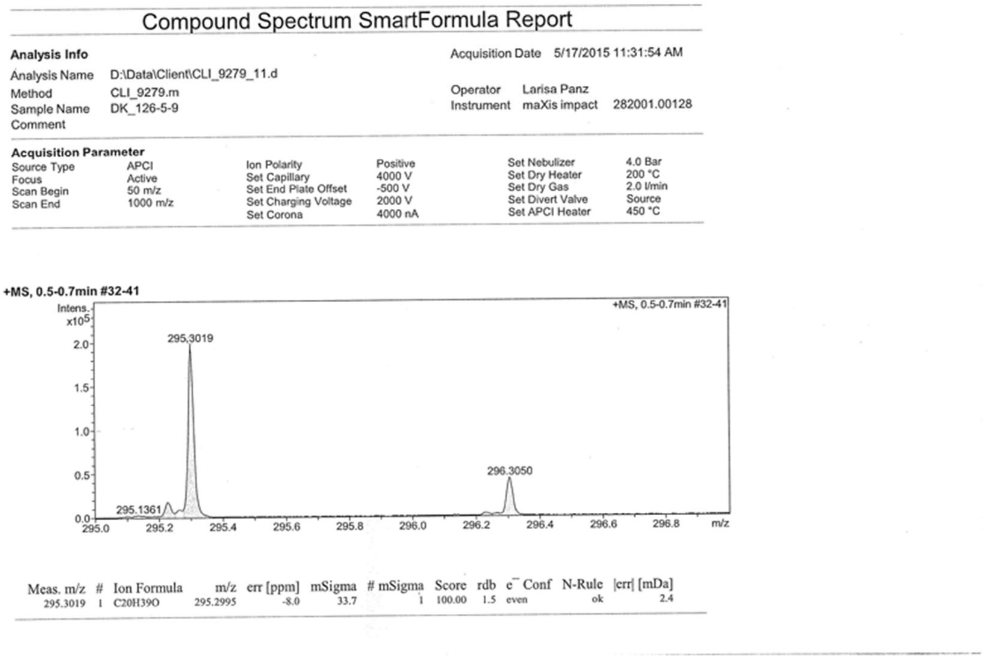

Figure S149. $^{1}H$ NMR spectrum of (*R*)-((*S*)-octadec-1-yn-3-yl)-3,3,3-trifluoro-2-methoxy-2-phenylpropanoate (***(R,S)*-34**) in $CDCl_3$

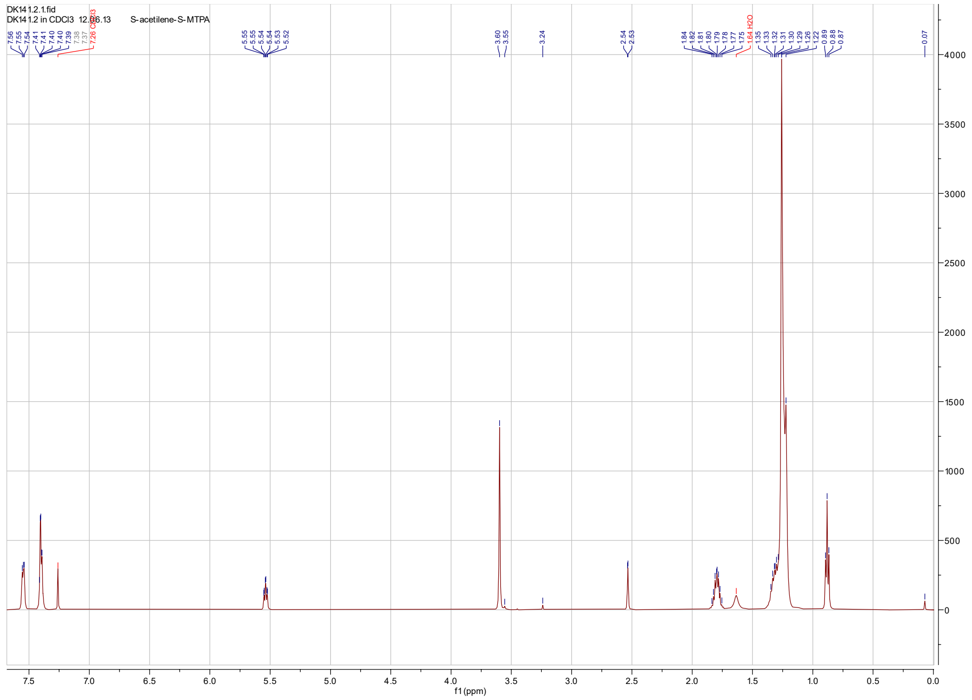

Figure S150. $^{13}C$ NMR spectrum of (*R*)-((*S*)-octadec-1-yn-3-yl)-3,3,3-trifluoro-2-methoxy-2-phenylpropanoate (***(R,S)*-34**) in $CDCl_3$

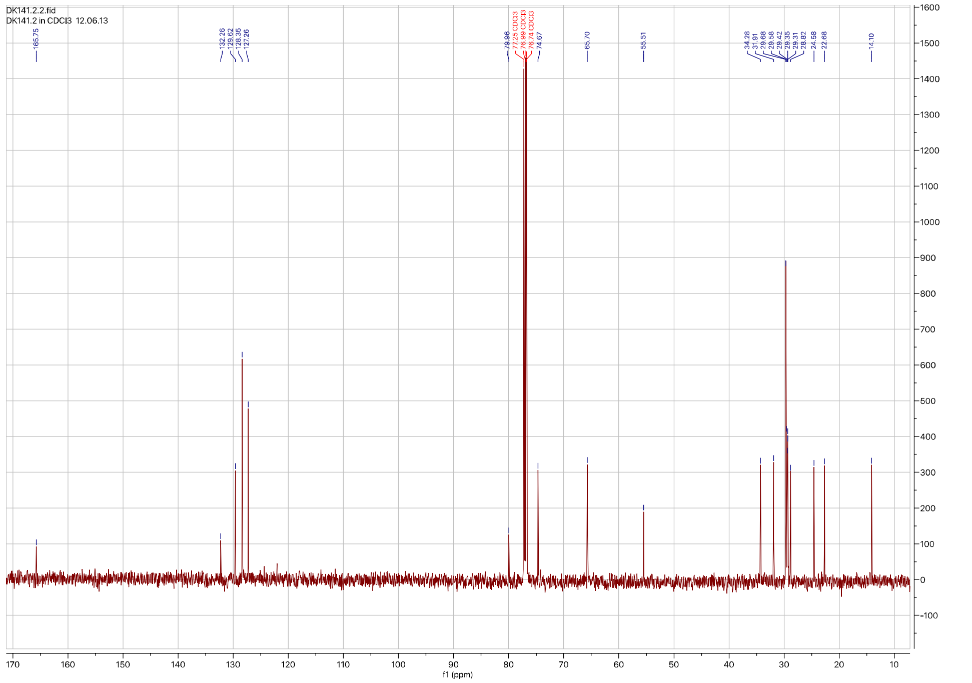

Figure S151. $^1$H NMR spectrum of (*R*)-((*R*)-octadec-1-yn-3-yl)-3,3,3-trifluoro-2-methoxy-2-phenylpropanoate (***(R,R)*-34**) in $CDCl_3$

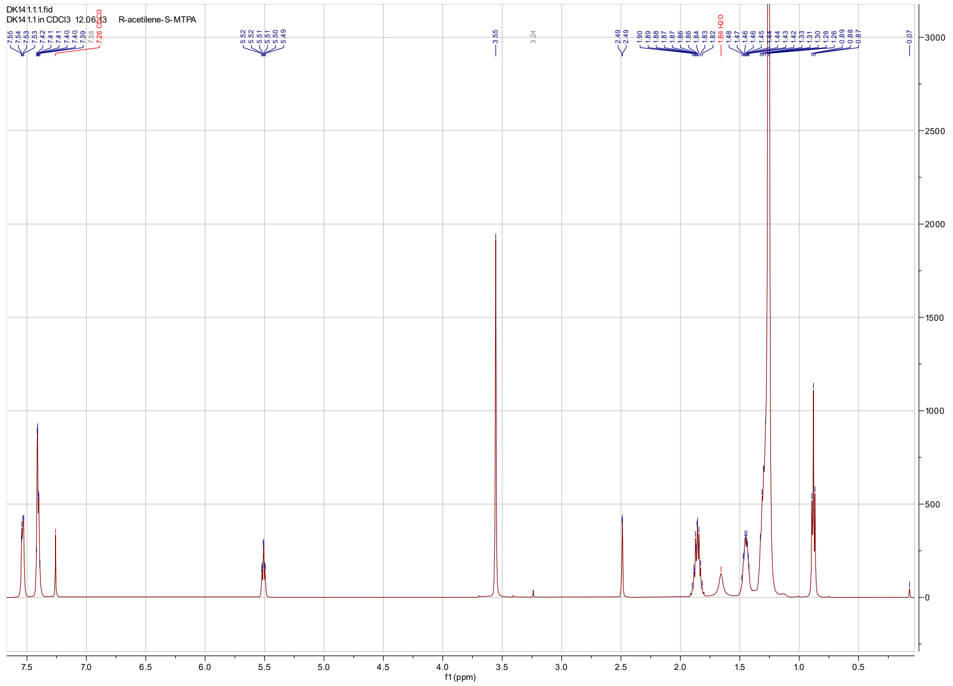

Figure S152. $^{13}$C NMR spectrum of (*R*)-((*R*)-octadec-1-yn-3-yl)-3,3,3-trifluoro-2-methoxy-2-phenylpropanoate (***(R,R)*-34**) in $CDCl_3$

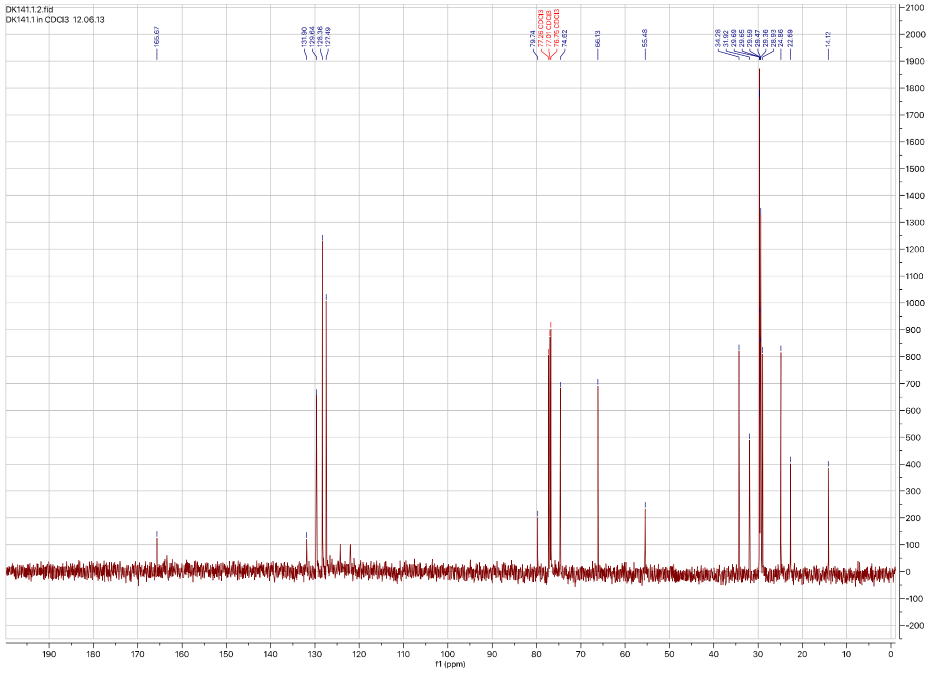

Figure S153. $^{1}$H NMR spectrum of octadec-1-yn-3-yl 4-methylbenzenesulfonate (***rac*-35**) in $CDCl_3$

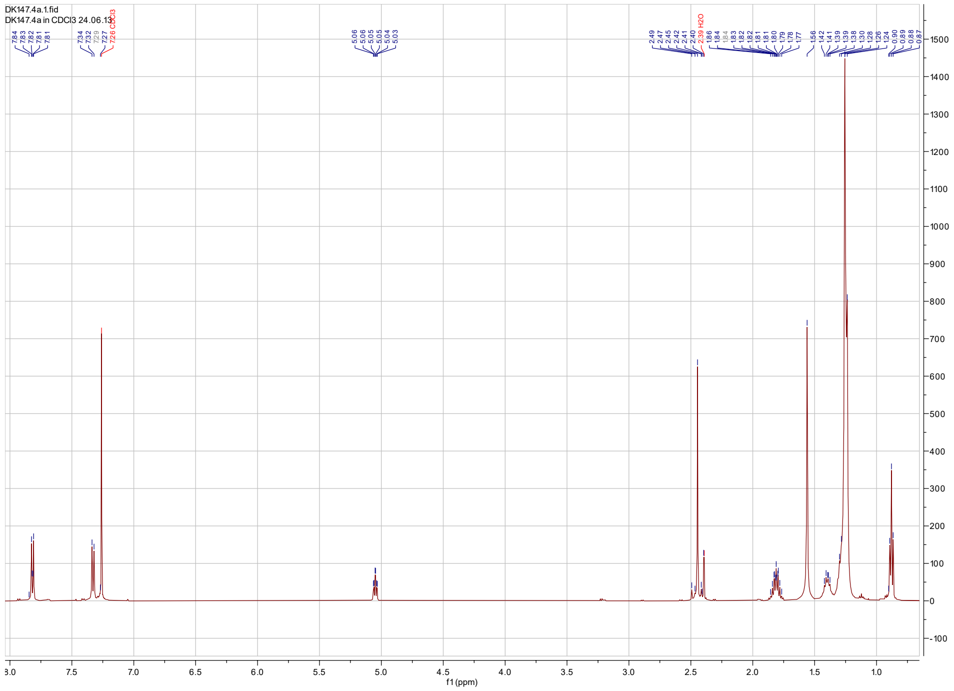


Figure S154. $^{13}$C NMR spectrum of octadec-1-yn-3-yl 4-methylbenzenesulfonate (***rac*-35**) in $CDCl_3$

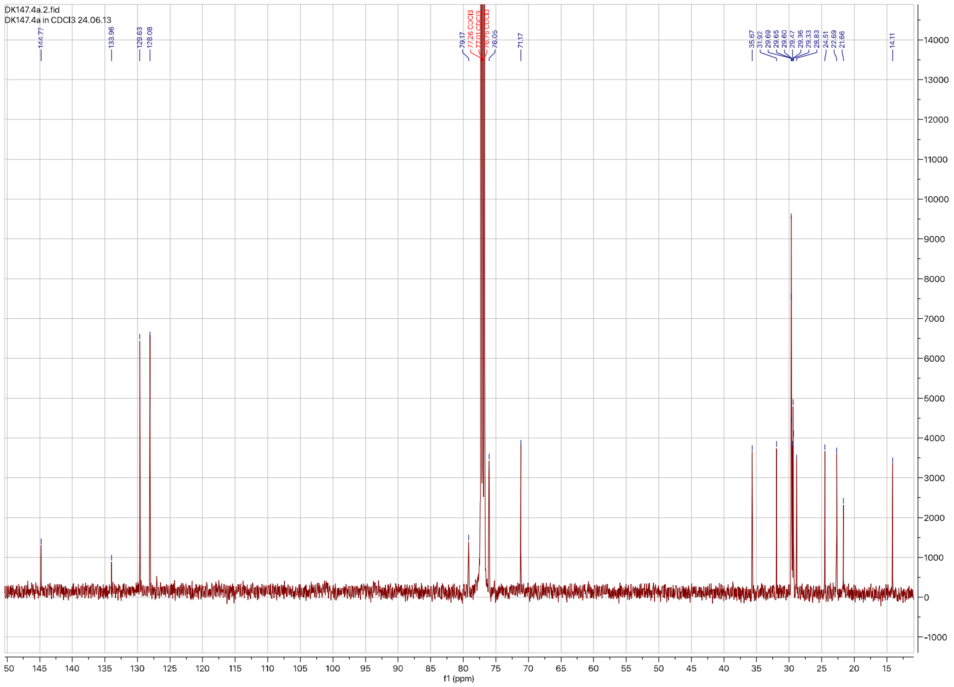

Figure S155. ESIMS of octadec-1-yn-3-yl 4-methylbenzenesulfonate (***rac*-35**)

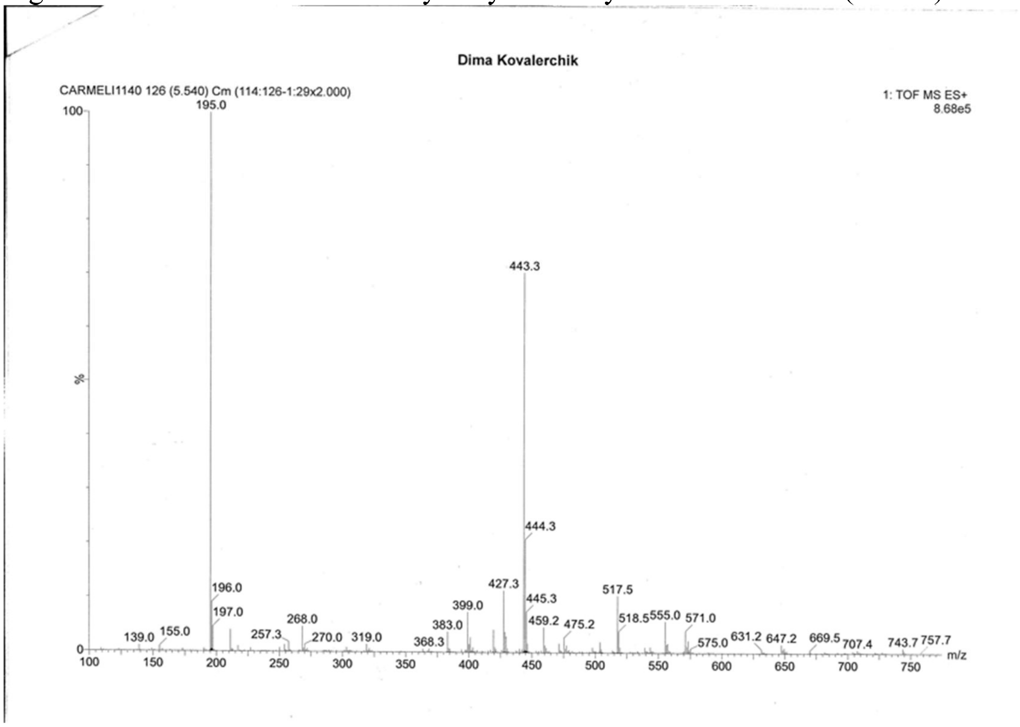


Figure S156. HRESIMS of octadec-1-yn-3-yl 4-methylbenzenesulfonate (***rac*-35**)

**Elemental Composition Report** Page 1

**Single Mass Analysis**
Tolerance = 2.0 mDa / DBE: min = -1.5, max = 50.0
Element prediction: Off
Number of isotope peaks used for i-FIT = 3

Monoisotopic Mass, Even Electron Ions
175 formula(e) evaluated with 3 results within limits (all results (up to 1000) for each mass)
Elements Used:
C: 20-40 H: 35-45 N: 0-5 O: 0-5 Na: 0-1 S: 0-2
1: TOF MS ES+
CARMELI1140 103 (4.541) Cm (101:103)

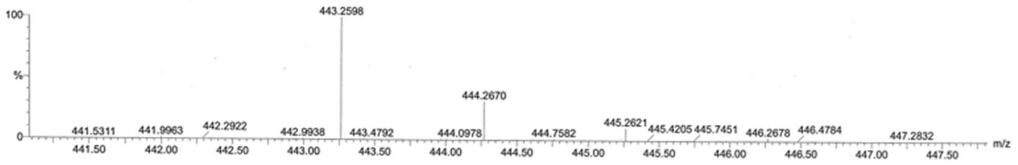


| Mass | Calc. Mass | mDa | PPM | DBE | i-FIT | i-FIT (Norm) | Formula |
|---|---|---|---|---|---|---|---|
| Minimum: | | | | -1.5 | | | |
| Maximum: | | 2.0 | 8000.0 | 50.0 | | | |
| 443.2598 | 443.2596 | 0.2 | 0.5 | 5.5 | 281.7 | 0.0 | C25 H40 O3 Na S ◄ |
| | 443.2580 | 1.8 | 4.1 | 4.5 | 285.2 | 3.5 | C22 H39 N2 O5 S |
| | 443.2586 | 1.2 | 2.7 | 13.5 | 288.6 | 6.9 | C30 H35 O3 |

Figure S157. $^{1}H$ NMR spectrum of 3-chlorooctadec-1-yne (***rac*-36**) in $CDCl_3$

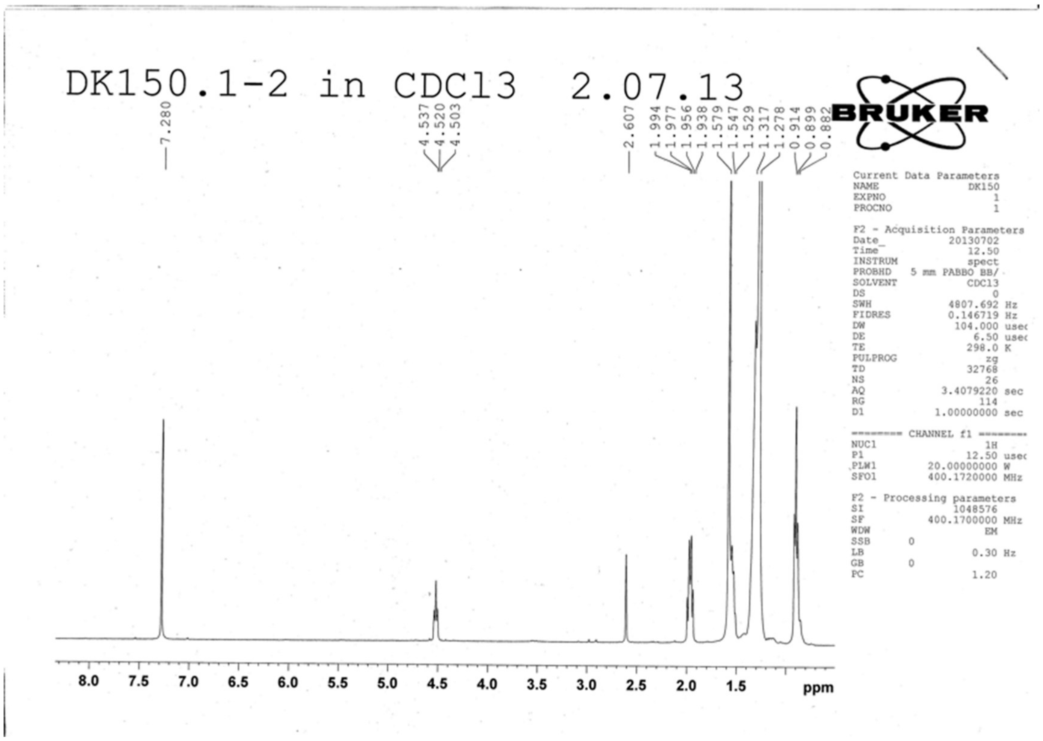


Figure S158. $^{1}H$ NMR spectrum of 3-chlorooctadec-1-yne (***rac*-36**) in $CDCl_3$

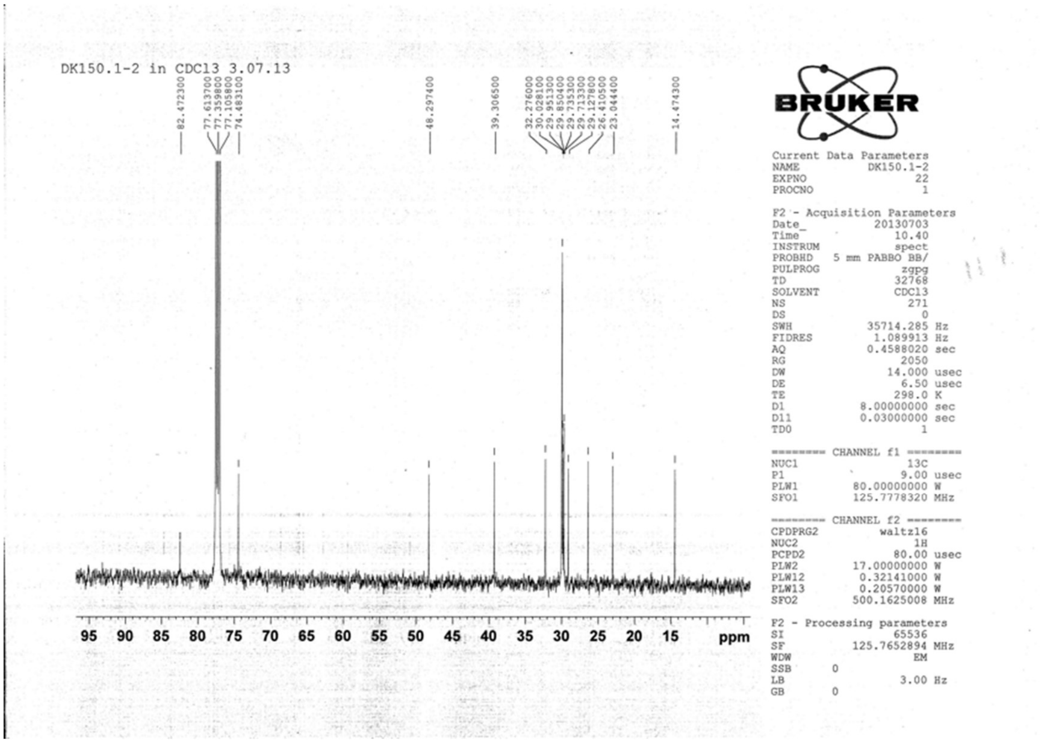

Figure S159. SMBEIMS of 3-chlorooctadec-1-yne (***rac*-36**)

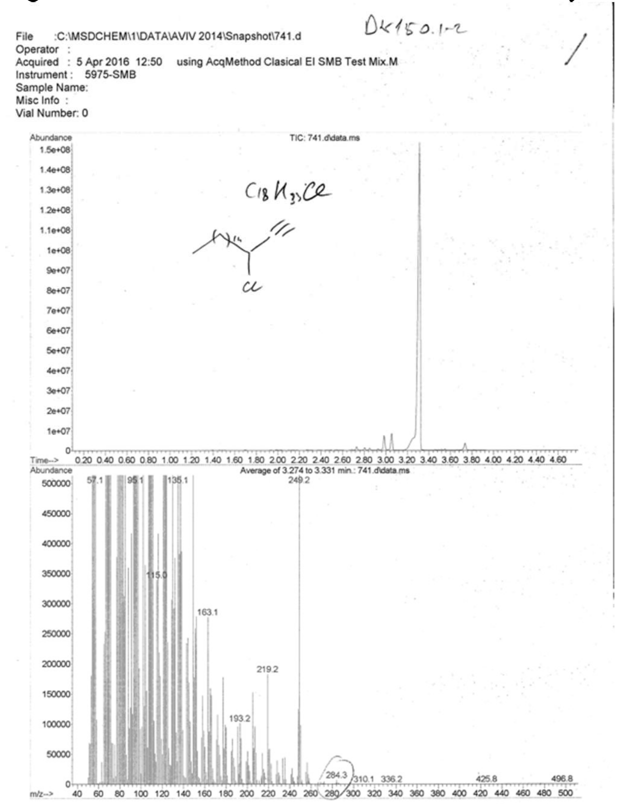


Figure S160. HRESIMS of 3-chlorooctadec-1-yne (***rac*-36**)

**Elemental Composition Report** **Page 1**

**Single Mass Analysis**
Tolerance = 50.0 PPM / DBE: min = -1.5, max = 50.0
Selected filters: None

Monoisotopic Mass, Odd and Even Electron Ions
761 formula(e) evaluated with 4 results within limits (all results (up to 1000) for each mass)
Elements Used:
C: 0-50 H: 0-50 N: 0-5 O: 0-5 S: 0-4 Cl: 0-1

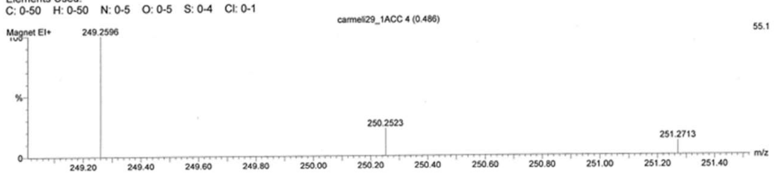


| | | | | | | |
|---|---|---|---|---|---|---|
| Minimum: | | | | -1.5 | | |
| Maximum: | | 5.0 | 50.0 | 50.0 | | |
| Mass | Calc. Mass | mDa | PPM | DBE | i-FIT | Formula |
| 249.2596 | 249.2582 | 1.4 | 5.6 | 2.5 | 2.2 | C18 H33 |
| | 249.2542 | 5.4 | 21.7 | -1.5 | 2.9 | C13 H33 N2 O2 |
| | 249.2654 | -5.8 | -23.3 | -1.5 | 3.2 | C12 H33 N4 O |
| | 249.2529 | 6.7 | 26.9 | -1.0 | 3.4 | C11 H31 N5 O |

Figure S161. $^1$H NMR spectrum of octadec-1-yn-3-amine (***rac*-37**) in $CDCl_3$

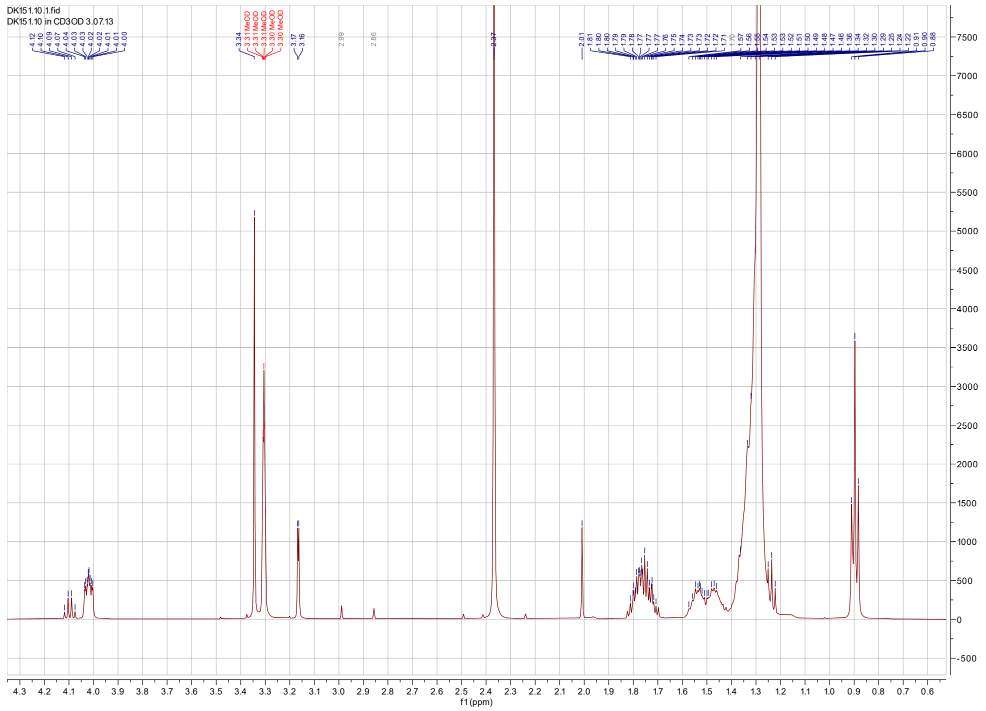

Figure S162. $^{13}$C NMR spectrum of octadec-1-yn-3-amine (***rac*-37**) in $CDCl_3$

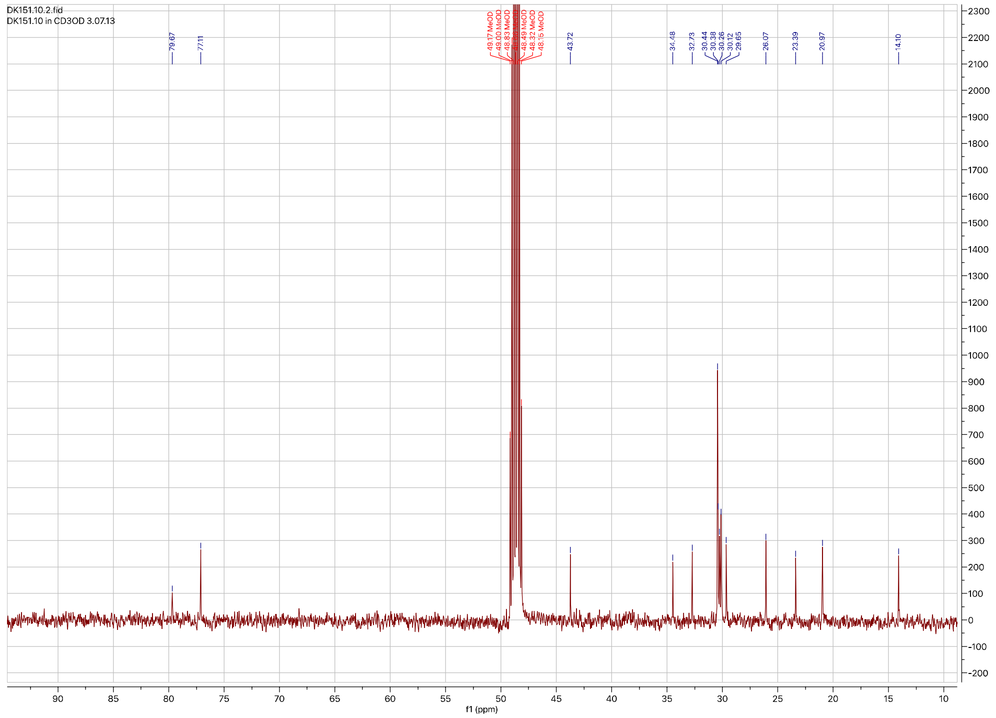

Figure S163. ESIMS of octadec-1-yn-3-amine (***rac*-37**)

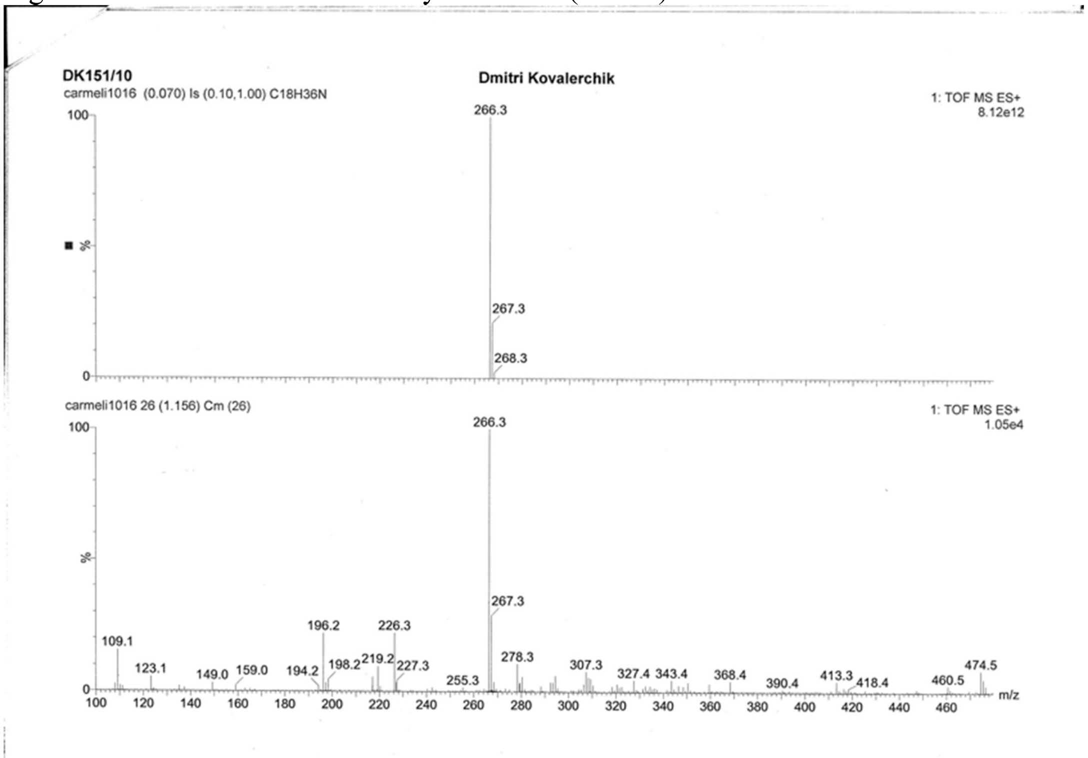


Figure S164. HRESIMS of octadec-1-yn-3-amine (***rac*-37**)

**Elemental Composition Report** Page 1

**Single Mass Analysis**
Tolerance = 10.0 PPM / DBE: min = -1.5, max = 50.0
Element prediction: Off
Number of isotope peaks used for i-FIT = 3

Monoisotopic Mass, Even Electron Ions
539 formula(e) evaluated with 2 results within limits (all results (up to 1000) for each mass)
Elements Used:
C: 1-25 H: 1-50 N: 0-10 O: 0-10 Na: 0-1
DK151/10 Dmitri Kovalerchik
carmeli1016 26 (1.156) Cm (26)

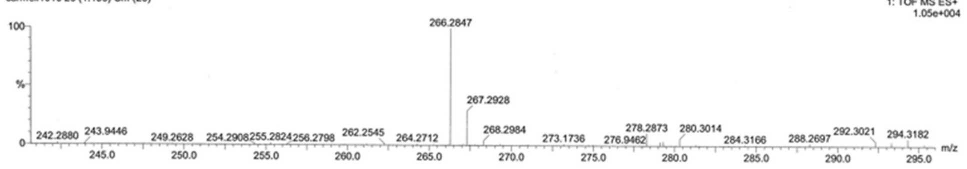


| | Mass | Calc. Mass | mDa | PPM | DBE | i-FIT | i-FIT (Norm) | Formula |
|---|---|---|---|---|---|---|---|---|
| Minimum: | | | | | -1.5 | | | |
| Maximum: | | | 5.0 | 10.0 | 50.0 | | | |
| | 266.2847 | 266.2848 | -0.1 | -0.4 | 1.5 | 240.9 | 0.3 | C18 H36 N ← |
| | | 266.2824 | 2.3 | 8.6 | -1.5 | 242.2 | 1.5 | C16 H37 N Na |

Figure S165. $^{1}$H NMR spectrum of 3-methoxyoctadec-1-yne (***rac*-38**) in $CDCl_3$

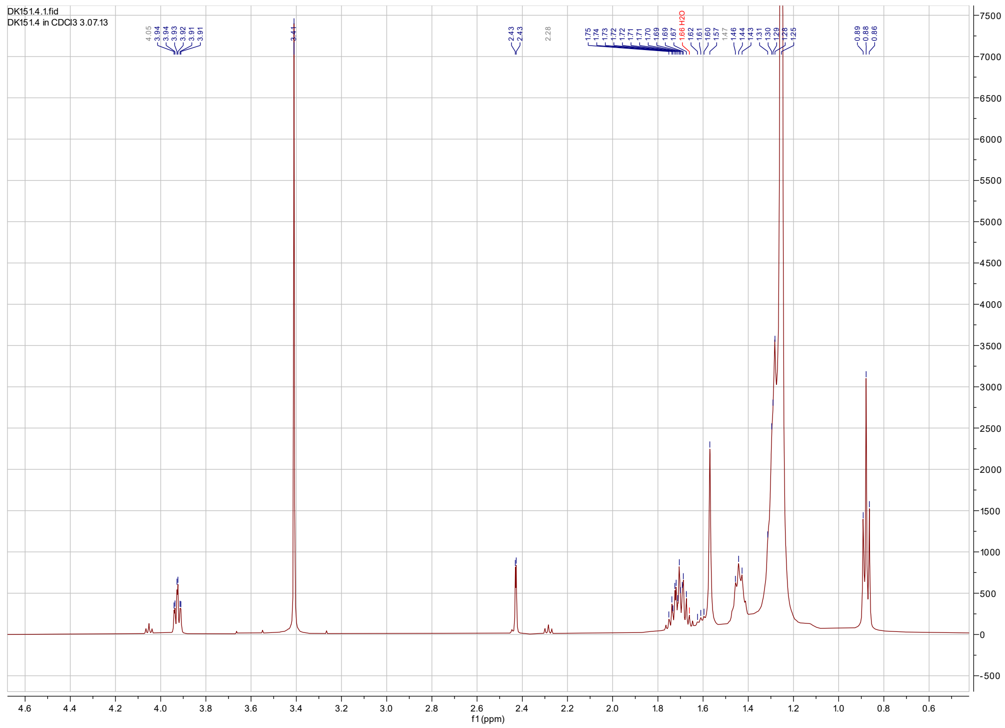


Figure S166. $^{1}$H NMR spectrum of 3-methoxyoctadec-1-yne (***rac*-38**) in $CDCl_3$

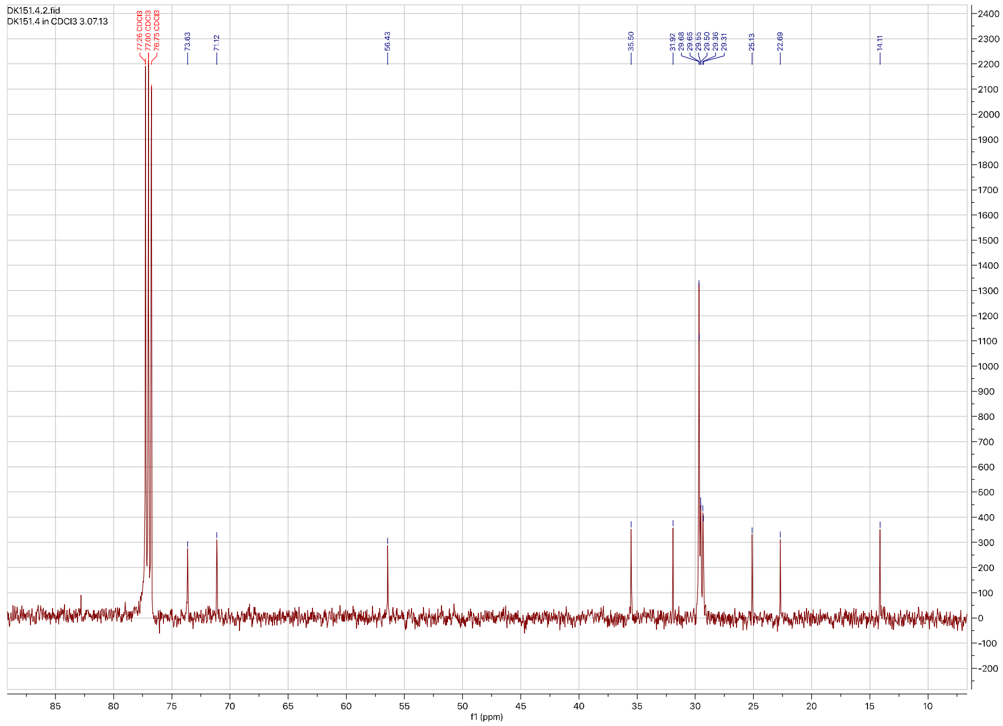

Figure S167. EIGCMS of 3-methoxyoctadec-1-yne (***rac*-38**)

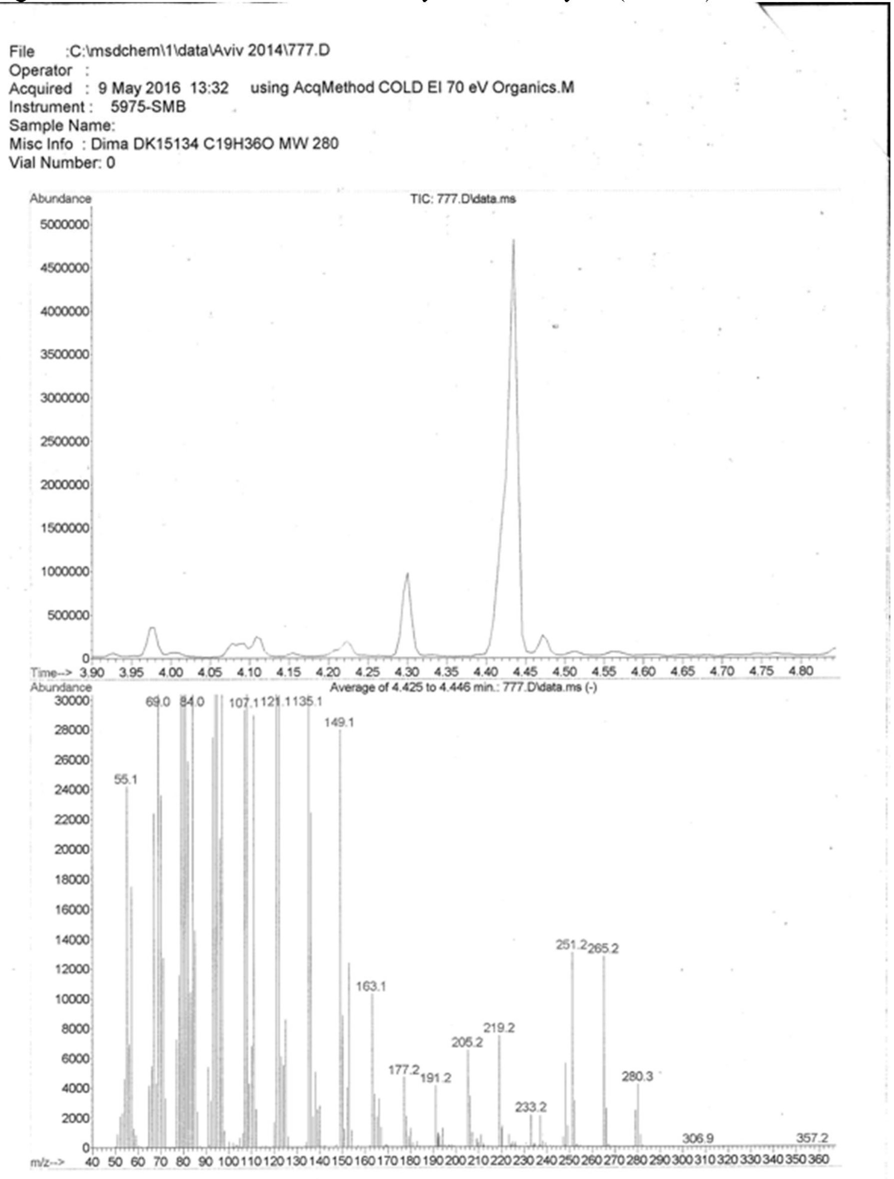

Figure S168. $^{1}$H NMR spectrum of *S*-octadec-1-yn-3-yl ethanethioate (***rac*-39**) in $CDCl_3$

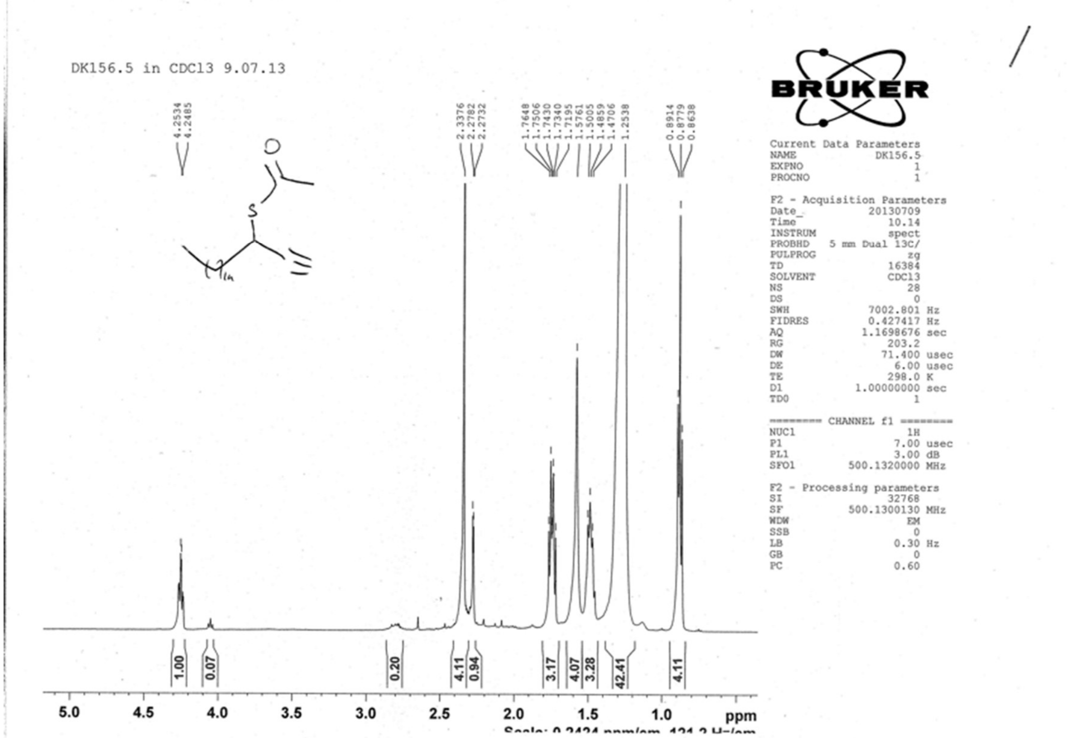


Figure S169. $^{13}$C NMR spectrum of *S*-octadec-1-yn-3-yl ethanethioate (***rac*-39**) in $CDCl_3$

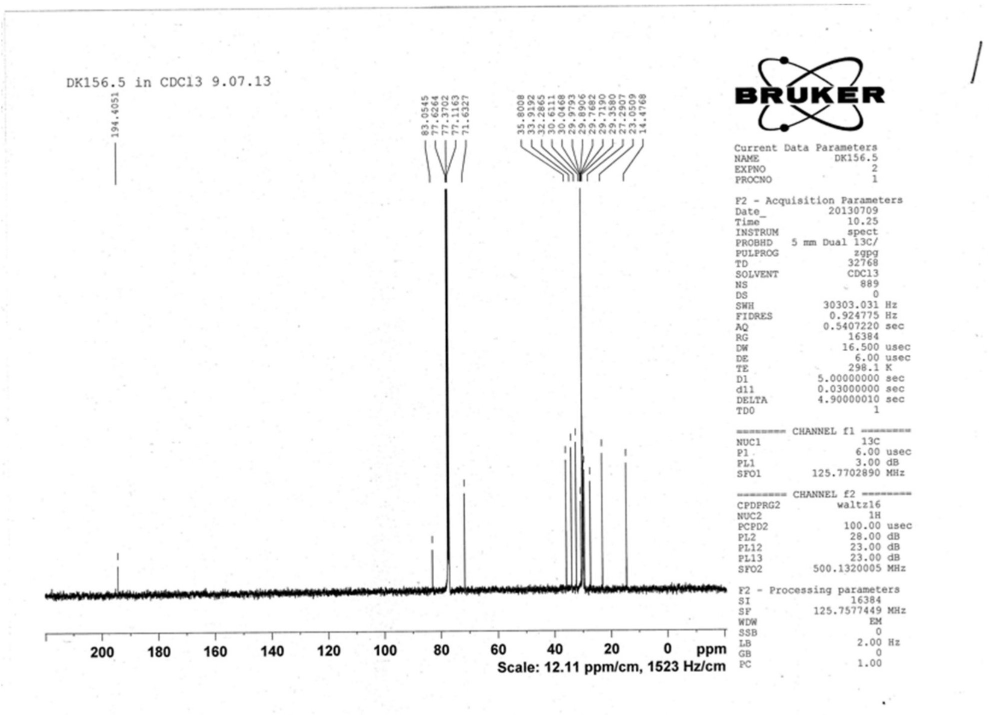

Figure S170. ESIMS of *S*-octadec-1-yn-3-yl ethanethioate (***rac*-39**)

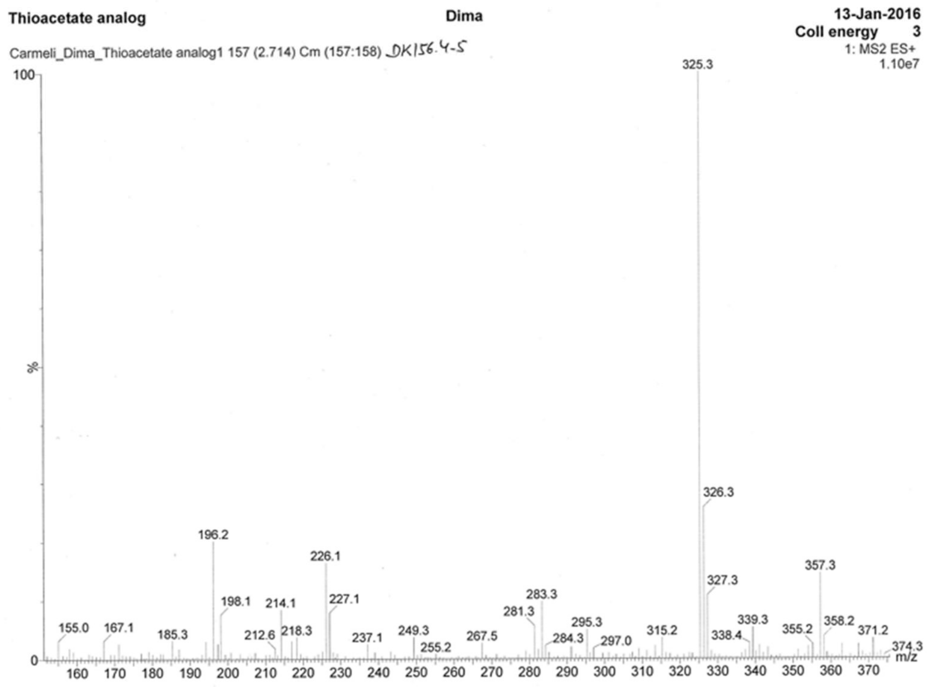

Figure S171. $^{1}$H NMR spectrum of octadec-1-yn-3-thiol (***rac*-40**) in $CDCl_3$

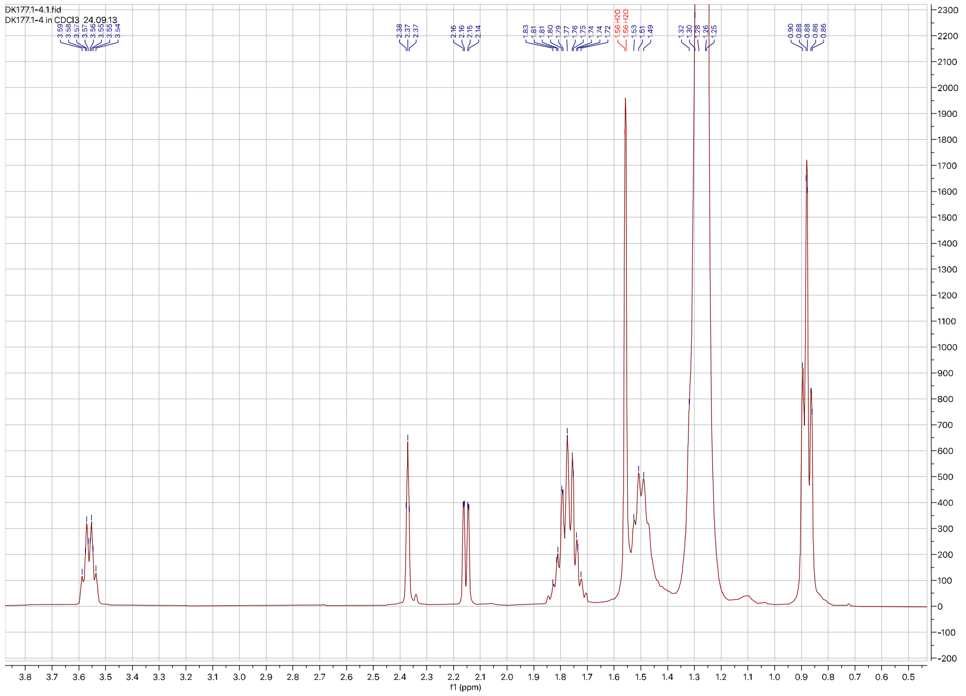

Figure S172. $^{13}$C NMR spectrum of octadec-1-yn-3-thiol (***rac*-40**) in $CDCl_3$

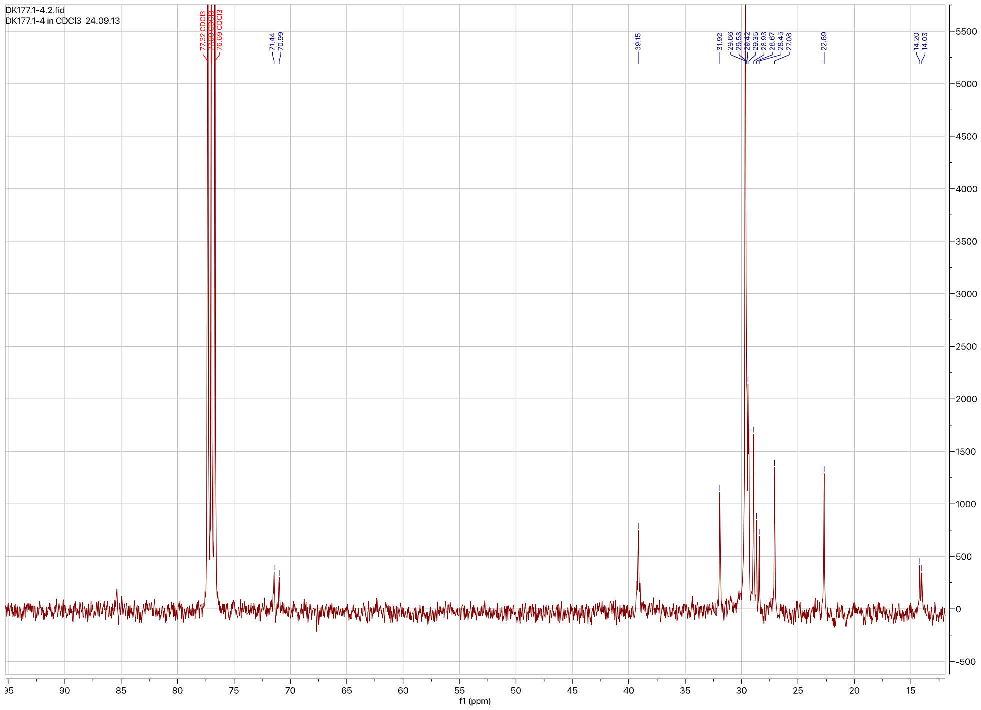

Figure S173. $^{1}H$ NMR spectrum of 3-methylnonadec-1-yn-3-ol (***rac*-41**) in $CDCl_3$

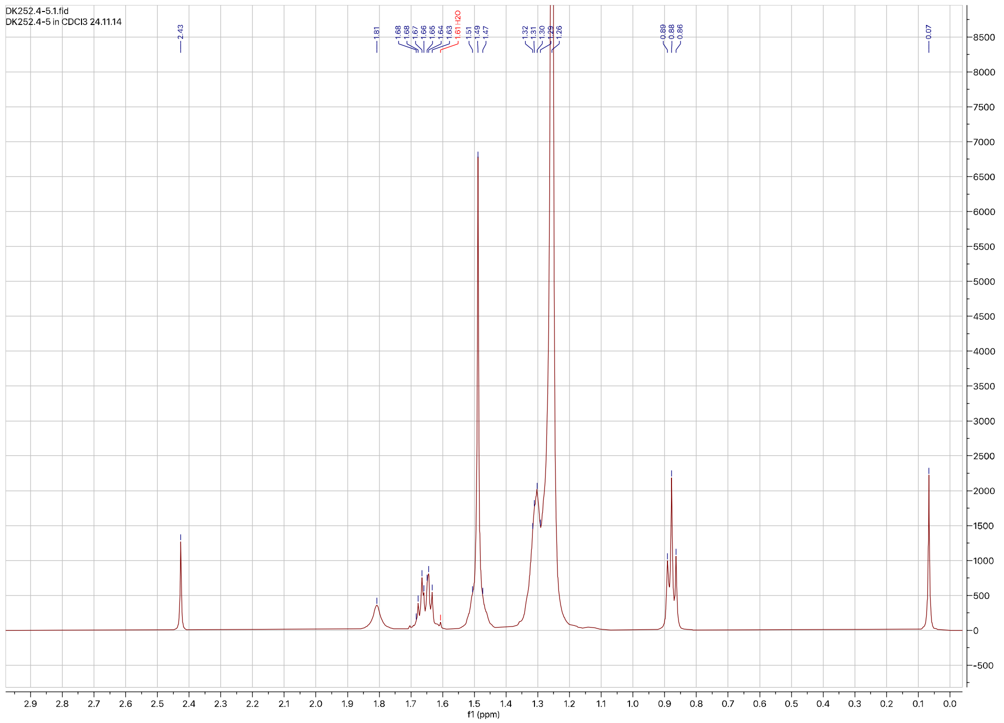

Figure S174. $^{13}C$ NMR spectrum of 3-methylnonadec-1-yn-3-ol (***rac*-41**) in $CDCl_3$

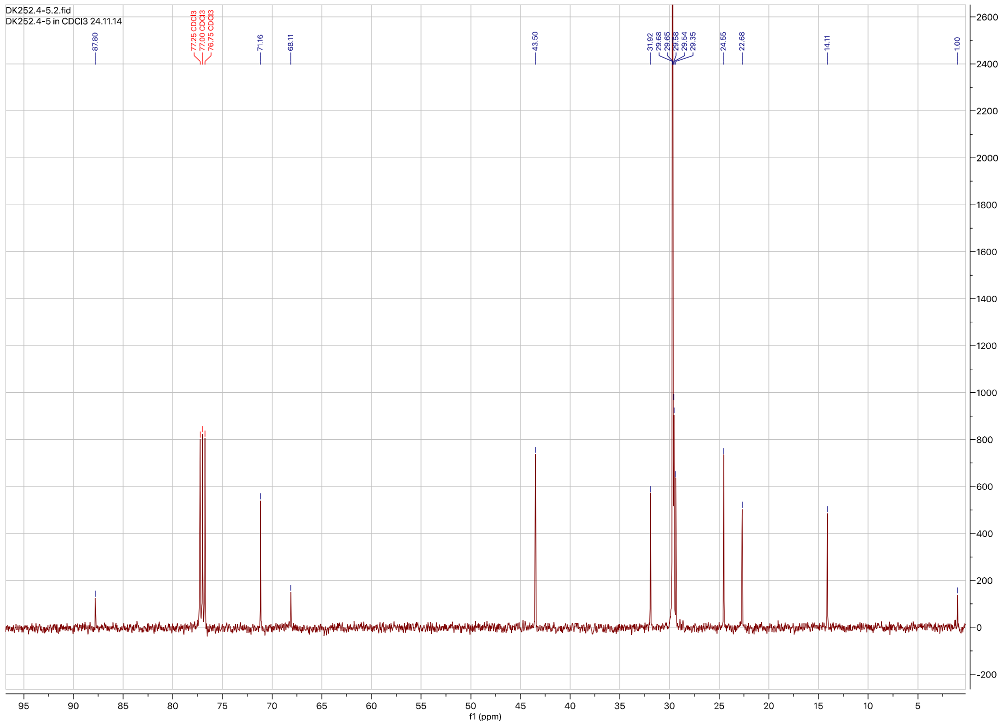

Figure S175. HRCIMS of 3-methylnonadec-1-yn-3-ol (***rac*-41**)

## Compound Spectrum SmartFormula Report

**Analysis Info**

| | | | |
|---|---|---|---|
| Analysis Name | D:\Data\Client\CLI_9279_16.d | Acquisition Date | 5/17/2015 11:57:03 AM |
| Method | CLI_9279.m | Operator | Larisa Panz |
| Sample Name | DK_252-4-7 | Instrument | maXis impact 282001.00128 |
| Comment | | | |

**Acquisition Parameter**

| | | | | | |
|---|---|---|---|---|---|
| Source Type | APCI | Ion Polarity | Positive | Set Nebulizer | 4.0 Bar |
| Focus | Active | Set Capillary | 4000 V | Set Dry Heater | 200 °C |
| Scan Begin | 50 m/z | Set End Plate Offset | -500 V | Set Dry Gas | 2.0 l/min |
| Scan End | 1000 m/z | Set Charging Voltage | 2000 V | Set Divert Valve | Source |
| | | Set Corona | 4000 nA | Set APCI Heater | 450 °C |

**+MS, 0.6-0.7min #37-43**

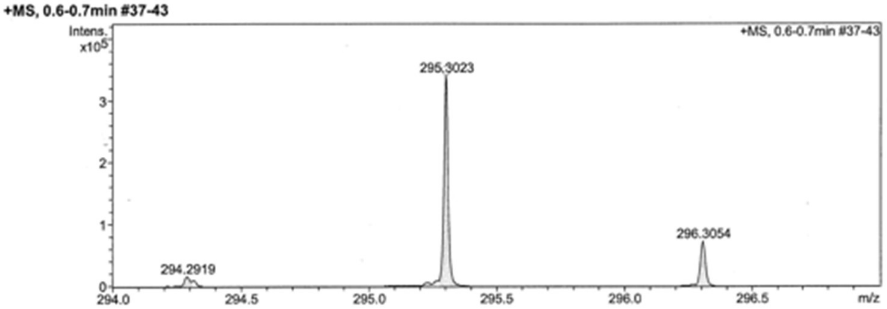


| Meas. m/z | # | Ion Formula | m/z | err [ppm] | mSigma | # mSigma | Score | rdb | e⁻ Conf | N-Rule | \|err\| [mDa] |
|---|---|---|---|---|---|---|---|---|---|---|---|
| 295.3023 | 1 | C20H39O | 295.2995 | -9.2 | 18.0 | 1 | 100.00 | 1.5 | even | ok | 2.7 ← |
| | 2 | C18H35N2O | 295.2744 | -94.4 | 20.1 | 2 | 0.00 | 2.5 | even | ok | 27.9 |

CLI_9279_16.d
Bruker Compass DataAnalysis 4.2 printed: 5/17/2015 12:00:31 PM by: Larisa Panz Page 1 of 1

Figure S176. $^{1}$H NMR spectrum of henicos-2-yn-4-ol (***rac*-42**) in $CDCl_3$

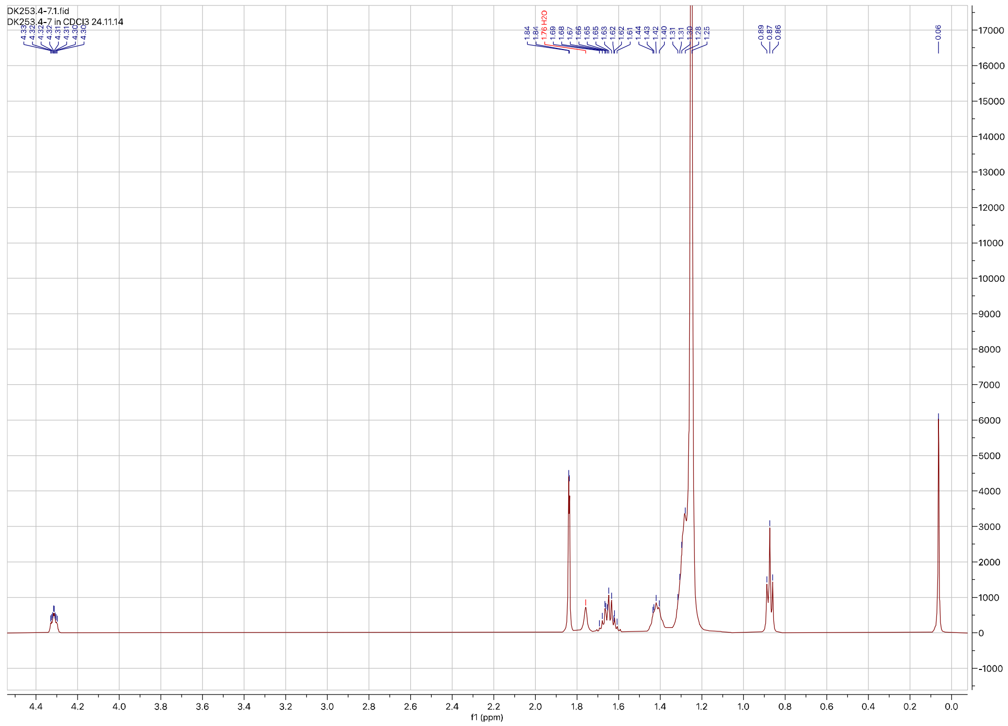


Figure S177. $^{13}$C NMR spectrum of henicos-2-yn-4-ol (***rac*-42**) in $CDCl_3$

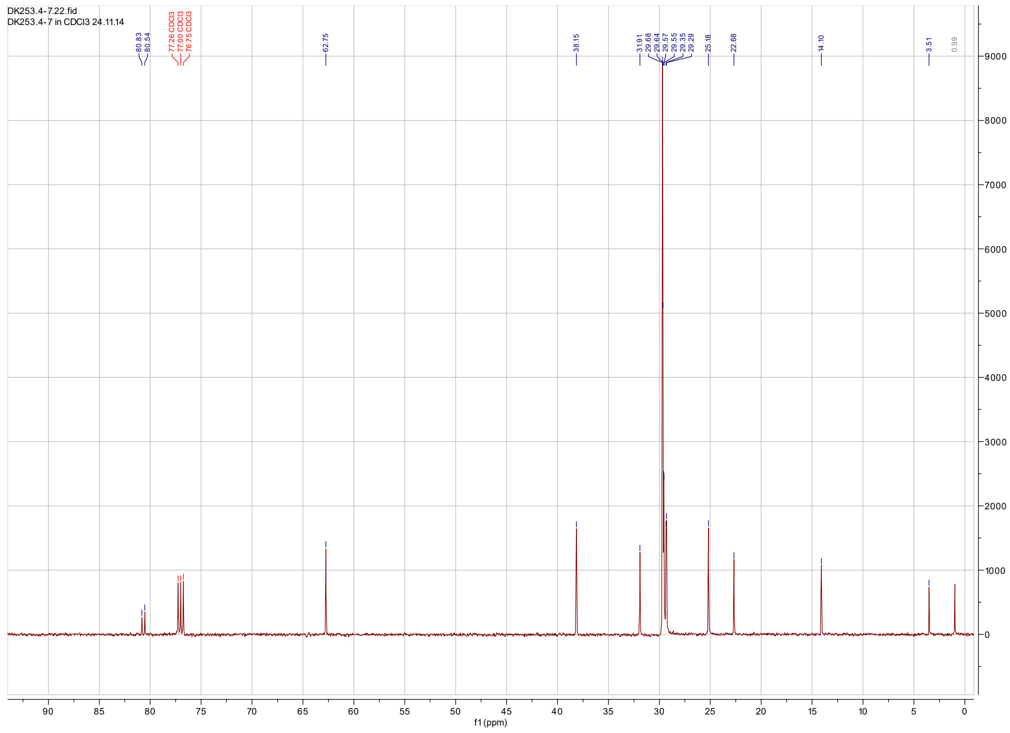

Figure S178. HRCIMS of henicos-2-yn-4-ol (***rac*-42**)

## Compound Spectrum SmartFormula Report

**Analysis Info**

| | | | |
|---|---|---|---|
| Analysis Name | D:\Data\Client\CLI_9279_15.d | Acquisition Date | 5/17/2015 11:51:17 AM |
| Method | CLI_9279.m | Operator | Larisa Panz |
| Sample Name | DK_253-4-7 | Instrument | maXis impact 282001.00128 |
| Comment | | | |

**Acquisition Parameter**

| | | | | | |
|---|---|---|---|---|---|
| Source Type | APCI | Ion Polarity | Positive | Set Nebulizer | 4.0 Bar |
| Focus | Active | Set Capillary | 4000 V | Set Dry Heater | 200 °C |
| Scan Begin | 50 m/z | Set End Plate Offset | -500 V | Set Dry Gas | 2.0 l/min |
| Scan End | 1000 m/z | Set Charging Voltage | 2000 V | Set Divert Valve | Source |
| | | Set Corona | 4000 nA | Set APCI Heater | 450 °C |

**+MS, 1.9-2.1min #113-123**

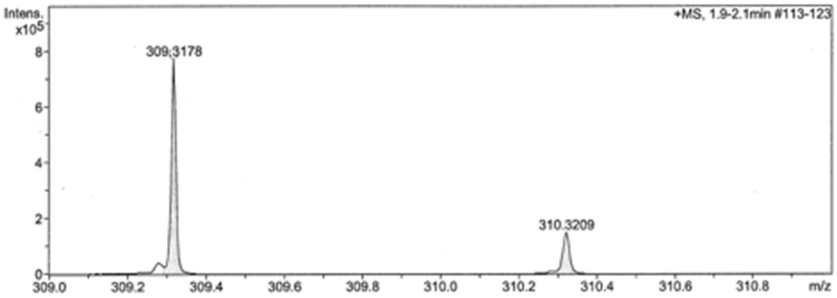


| Meas. m/z | # | Ion Formula | m/z | err [ppm] | mSigma | # mSigma | Score | rdb | e⁻ Conf | N-Rule | \|err\| [mDa] |
|---|---|---|---|---|---|---|---|---|---|---|---|
| 309.3178 | 1 | C19H37N2O | 309.2900 | -89.7 | 17.8 | 1 | 0.00 | 2.5 | even | ok | 27.8 |
| | 2 | C21H41O | 309.3152 | -8.4 | 24.4 | 2 | 100.00 | 1.5 | even | ok | 2.6 |

CLI_9279_15.d
Bruker Compass DataAnalysis 4.2 printed: 5/17/2015 11:55:30 AM by: Larisa Panz Page 1 of 1

Figure S179. $^1$H NMR spectrum of 1-(3-tetradecylphenyl)prop-2-yn-1-ol (***rac*-45**) in $CDCl_3$

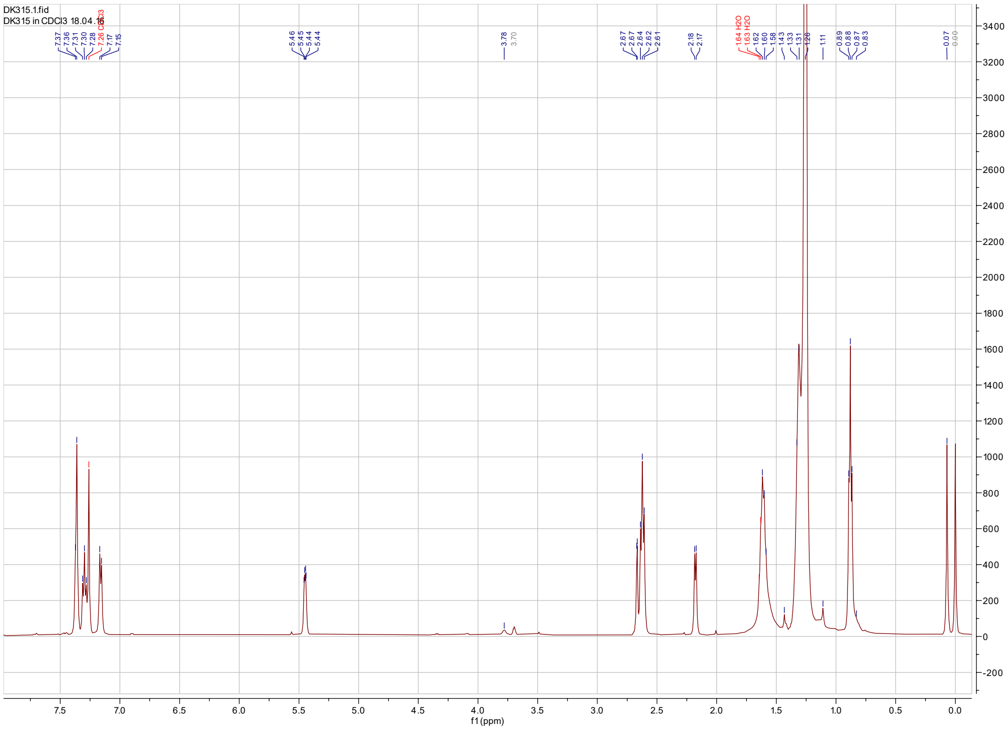


Figure S180. $^{13}$C NMR spectrum of 1-(3-tetradecylphenyl)prop-2-yn-1-ol (***rac*-45**) in $CDCl_3$

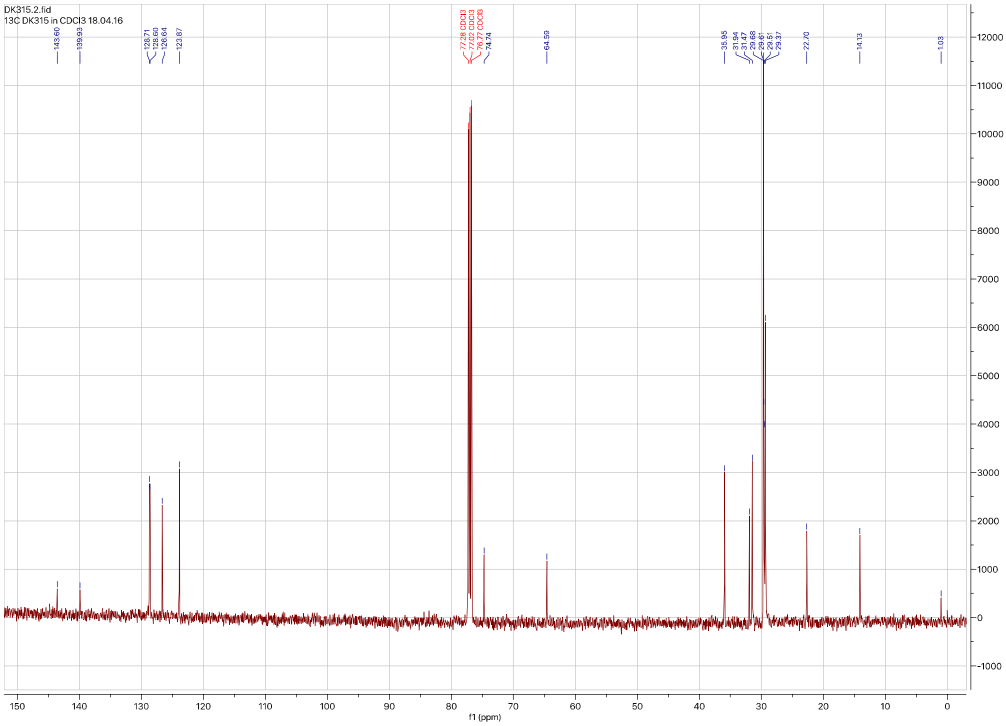

Figure S181. HREIMS of 1-(3-tetradecylphenyl)prop-2-yn-1-ol (***rac*-45**)

**Elemental Composition Report** DK315 **Page 1**

**Single Mass Analysis**
Tolerance = 100.0 PPM / DBE: min = -1.5, max = 50.0
Selected filters: None

Monoisotopic Mass, Odd and Even Electron Ions
26 formula(e) evaluated with 3 results within limits (all results (up to 1000) for each mass)
Elements Used:
C: 15-30 H: 0-50 O: 0-5

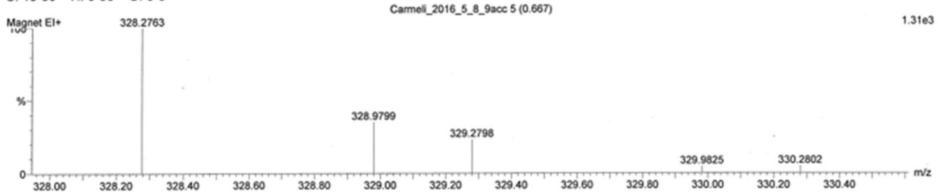


| | | | | | | |
|---|---|---|---|---|---|---|
| Minimum: | | | | -1.5 | | |
| Maximum: | | 5.0 | 100.0 | 50.0 | | |
| Mass | Calc. Mass | mDa | PPM | DBE | i-FIT | Formula |
| 328.2763 | 328.2766 | -0.3 | -0.9 | 6.0 | 4.7 | C23 H36 O ← |
| | 328.2614 | 14.9 | 45.4 | 2.0 | 13.1 | C19 H36 O4 |
| | 328.2977 | -21.4 | -65.2 | 1.0 | 23.3 | C20 H40 O3 |

Figure S182. $^{1}$H NMR spectrum of 1-(2-tetradecylphenyl)prop-2-yn-1-ol (***rac*-48**) in $CDCl_3$

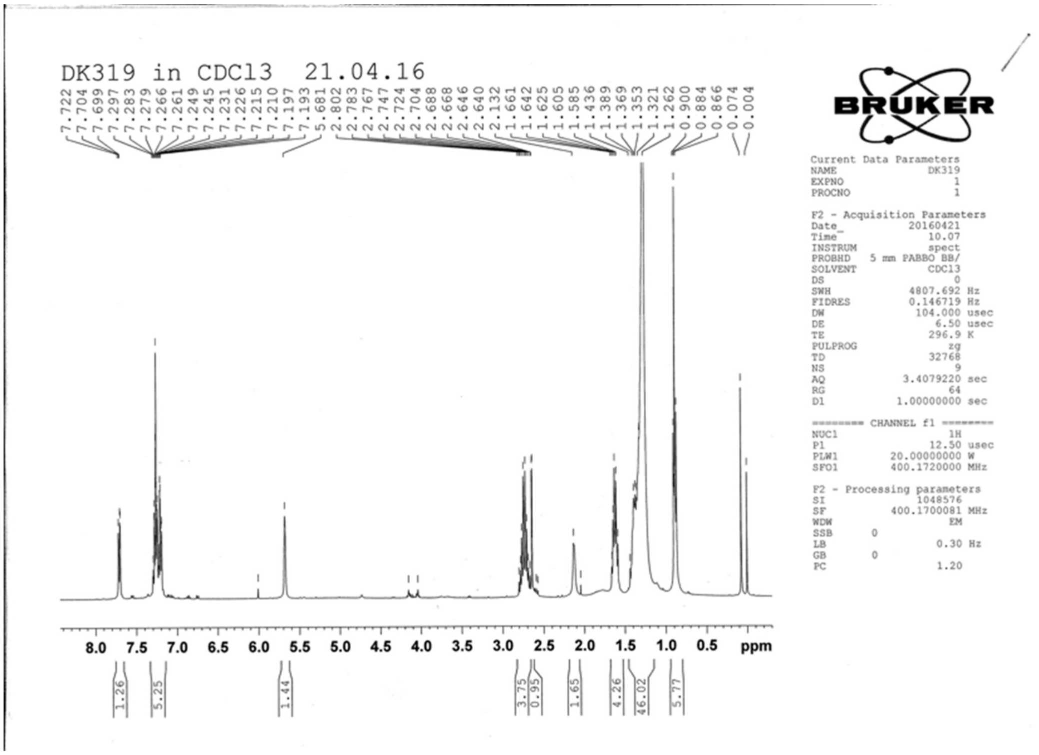


Figure S183. $^{13}$C NMR spectrum of 1-(2-tetradecylphenyl)prop-2-yn-1-ol (***rac*-48**) in $CDCl_3$

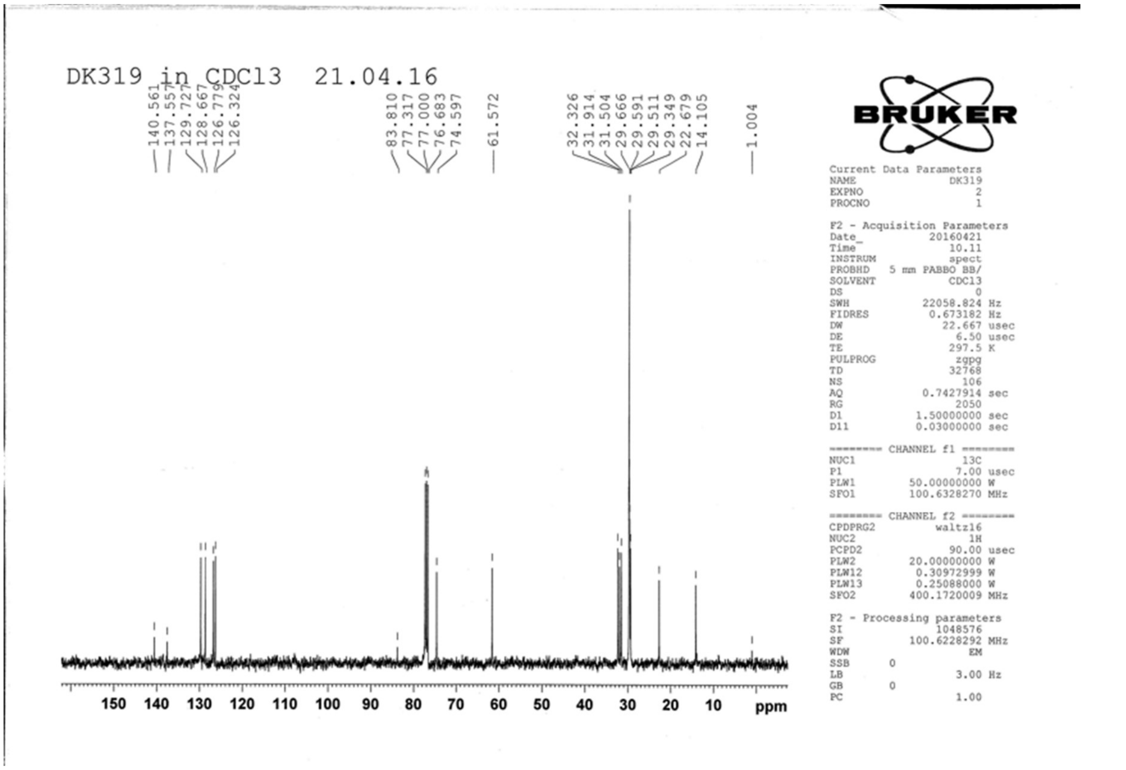

Figure S184. HREIMS of 1-(2-tetradecylphenyl)prop-2-yn-1-ol (***rac*-48**)

**Elemental Composition Report** DK310 **Page 1**

**Single Mass Analysis**
Tolerance = 100.0 PPM / DBE: min = -1.5, max = 50.0
Selected filters: None

Monoisotopic Mass, Odd and Even Electron Ions
26 formula(e) evaluated with 3 results within limits (all results (up to 1000) for each mass)
Elements Used:
C: 15-30 H: 0-50 O: 0-5

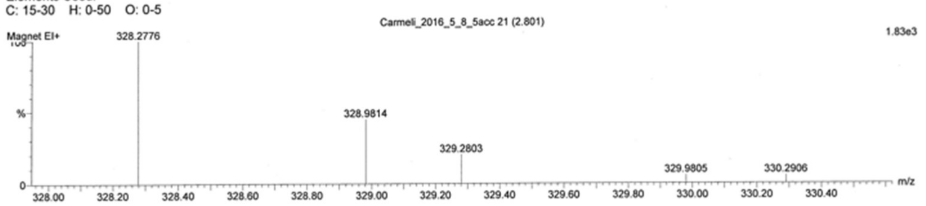


| | | | | | | |
|---|---|---|---|---|---|---|
| Minimum: | | | | -1.5 | | |
| Maximum: | | 5.0 | 100.0 | 50.0 | | |
| Mass | Calc. Mass | mDa | PPM | DBE | i-FIT | Formula |
| 328.2776 | 328.2766 | 1.0 | 3.0 | 6.0 | 20.1 | C23 H36 O ← |
| | 328.2614 | 16.2 | 49.3 | 2.0 | 25.9 | C19 H36 O4 |
| | 328.2977 | -20.1 | -61.2 | 1.0 | 35.6 | C20 H40 O3 |

Figure S185. $^{1}H$ NMR spectrum of 1-phenylprop-2-yn-1-ol (***rac*-49**) in $CDCl_3$

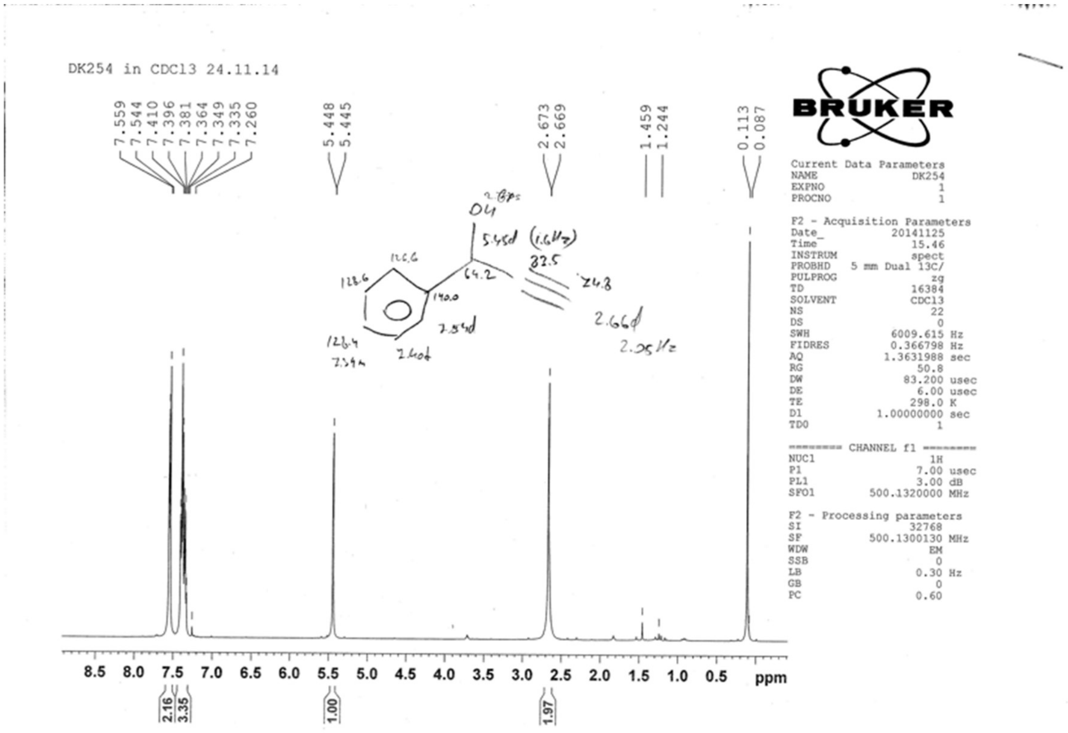


Figure S186. $^{13}C$ NMR spectrum of 1-phenylprop-2-yn-1-ol (***rac*-49**) in $CDCl_3$

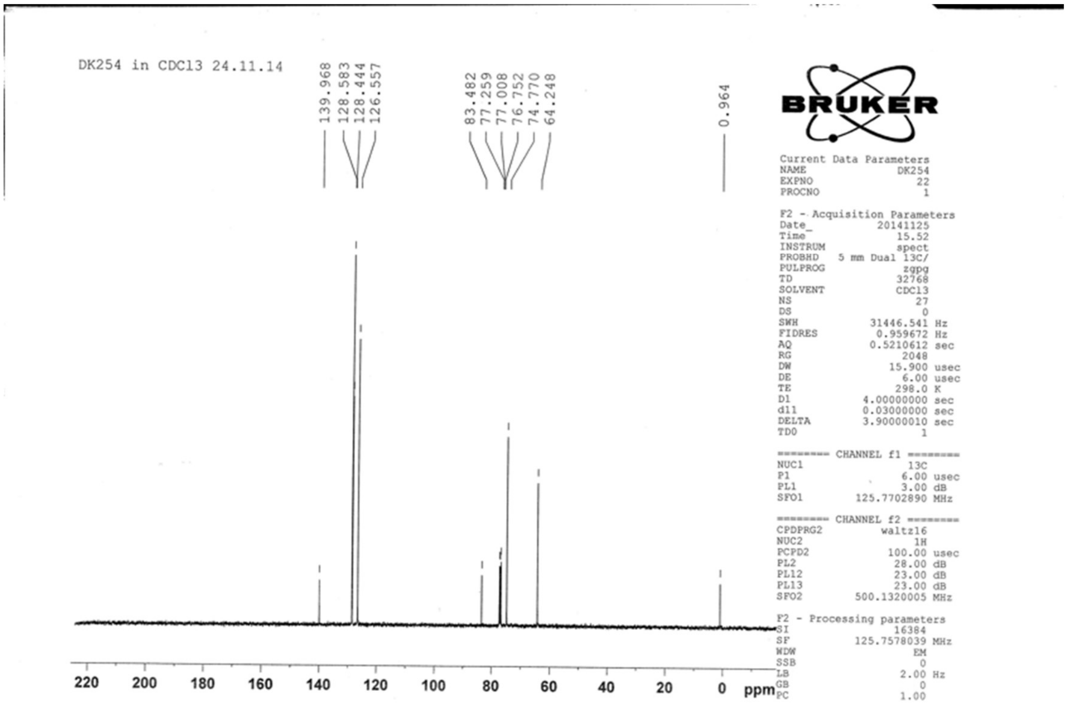

Figure S187. HREIMS of 1-phenylprop-2-yn-1-ol (***rac*-49**)

DK254

..ental Composition Report

Page 1

**Single Mass Analysis**
Tolerance = 50.0 PPM / DBE: min = -1.5, max = 50.0
Element prediction: Off
Number of isotope peaks used for i-FIT = 3

Monoisotopic Mass, Even Electron Ions
44 formula(e) evaluated with 2 results within limits (all results (up to 1000) for each mass)
Elements Used:
C: 1-20 H: 1-20 N: 0-5 O: 0-5
DK254_Dmitri Kovalerchik
TOF MS AP+

CARMELI1017E 34 (1.728) Cm (34)

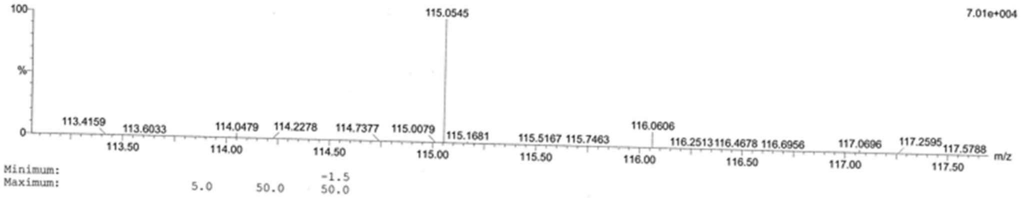


| | | | | | | | |
|---|---|---|---|---|---|---|---|
| Minimum: | | | | -1.5 | | | |
| Maximum: | | 5.0 | 50.0 | 50.0 | | | |
| Mass | Calc. Mass | mDa | PPM | DBE | i-FIT | i-FIT (Norm) | Formula |
| 115.0545 | 115.0548 | -0.3 | -2.6 | 6.5 | 491.5 | 0.0 | C9 H7 ← MH⁺-H₂O |
| | 115.0508 | 3.7 | 32.2 | 2.5 | 494.8 | 3.3 | C4 H7 N2 O2 |

Figure S188. Dose response curves of the compounds described in Table 1 obtained from screening of NSCLC U-1810 cells or diploid fibroblast WI-38 cells are presented. The $IC_{50}$ values were deduced from the cell viability curves.

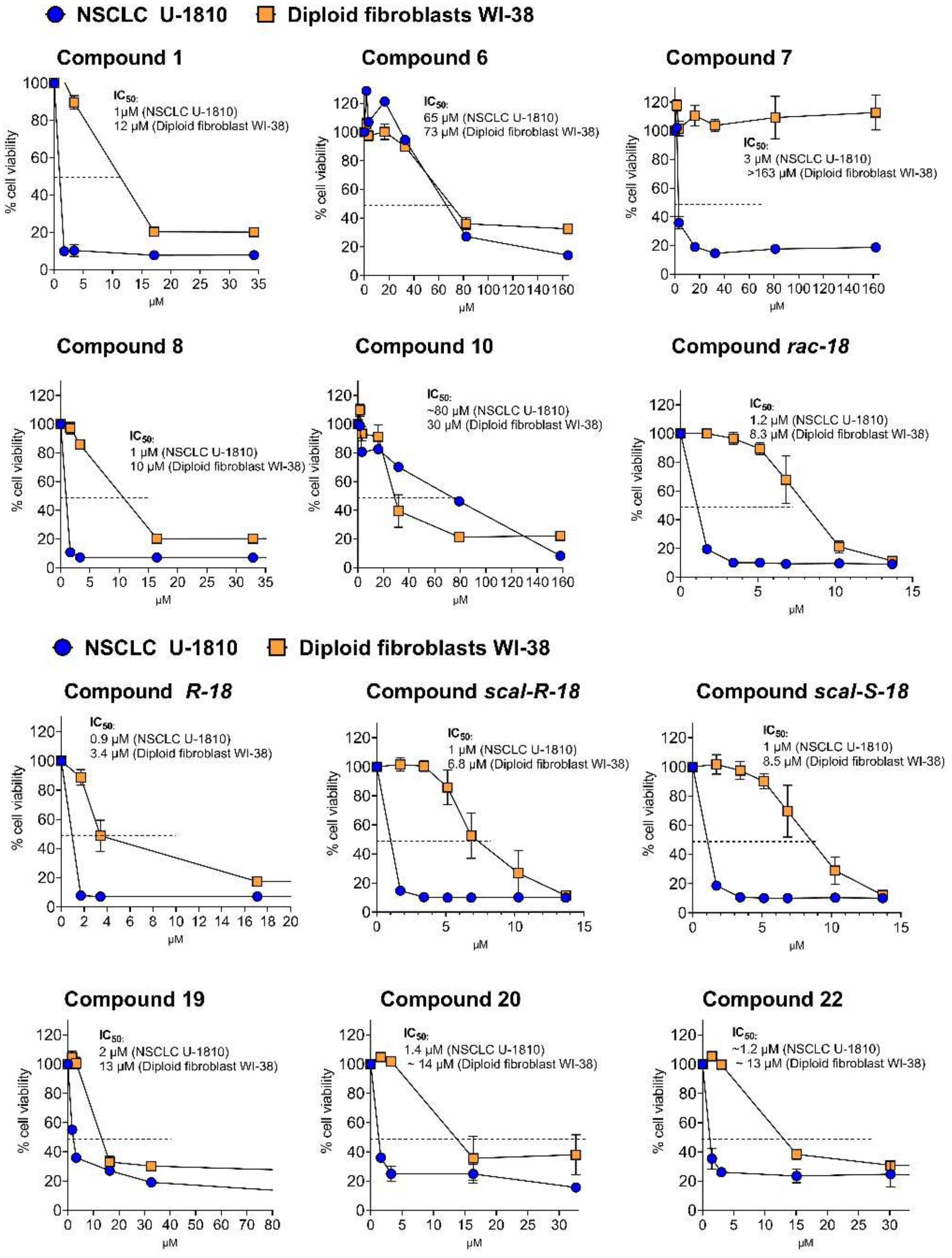

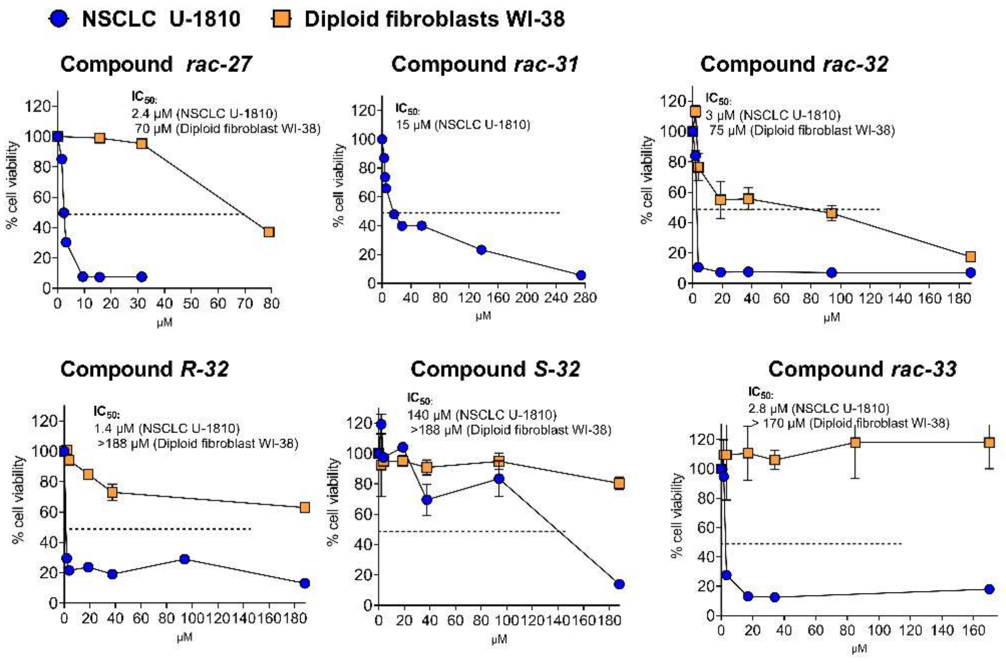
NSCLC U-1810
Diploid fibroblasts WI-38
Compound rac-27
IC50:
2.4 μM (NSCLC U-1810)
70 μM (Diploid fibroblast WI-38)
Compound rac-31
IC50:
15 μM (NSCLC U-1810)
Compound rac-32
IC50:
3 μM (NSCLC U-1810)
75 μM (Diploid fibroblast WI-38)
Compound R-32
IC50:
1.4 μM (NSCLC U-1810)
>188 μM (Diploid fibroblast WI-38)
Compound S-32
IC50:
140 μM (NSCLC U-1810)
>188 μM (Diploid fibroblast WI-38)
Compound rac-33
IC50:
2.8 μM (NSCLC U-1810)
> 170 μM (Diploid fibroblast WI-38)
% cell viability
μM

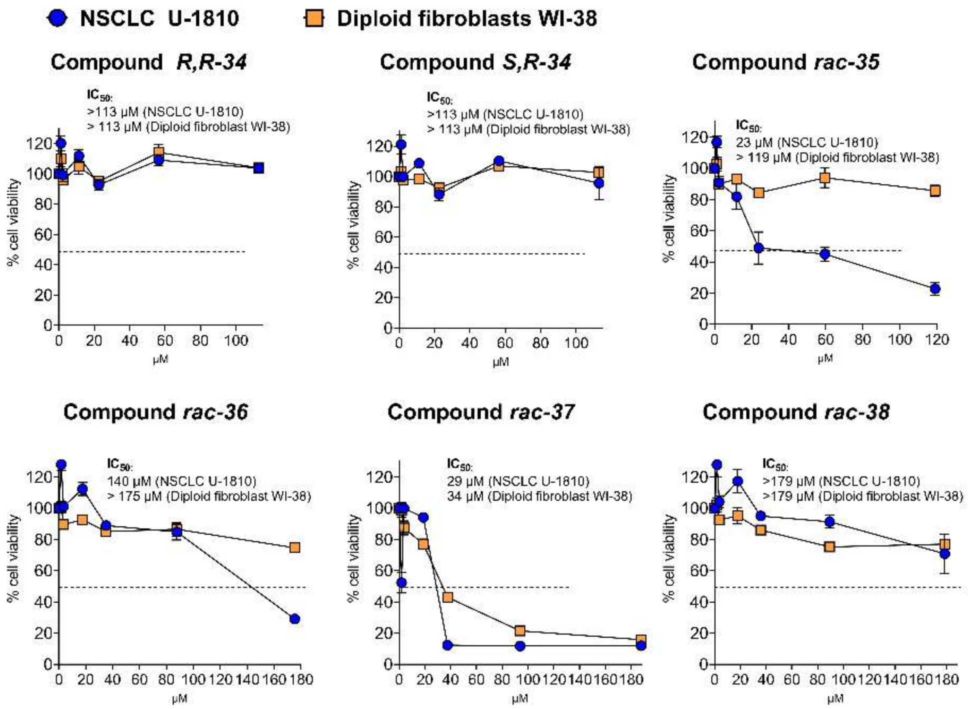
NSCLC U-1810
Diploid fibroblasts WI-38
Compound R,R-34
IC50:
>113 μM (NSCLC U-1810)
> 113 μM (Diploid fibroblast WI-38)
Compound S,R-34
IC50:
>113 μM (NSCLC U-1810)
> 113 μM (Diploid fibroblast WI-38)
Compound rac-35
IC50:
23 μM (NSCLC U-1810)
> 119 μM (Diploid fibroblast WI-38)
Compound rac-36
IC50:
140 μM (NSCLC U-1810)
> 175 μM (Diploid fibroblast WI-38)
Compound rac-37
IC50:
29 μM (NSCLC U-1810)
34 μM (Diploid fibroblast WI-38)
Compound rac-38
IC50:
>179 μM (NSCLC U-1810)
>179 μM (Diploid fibroblast WI-38)
% cell viability
μM

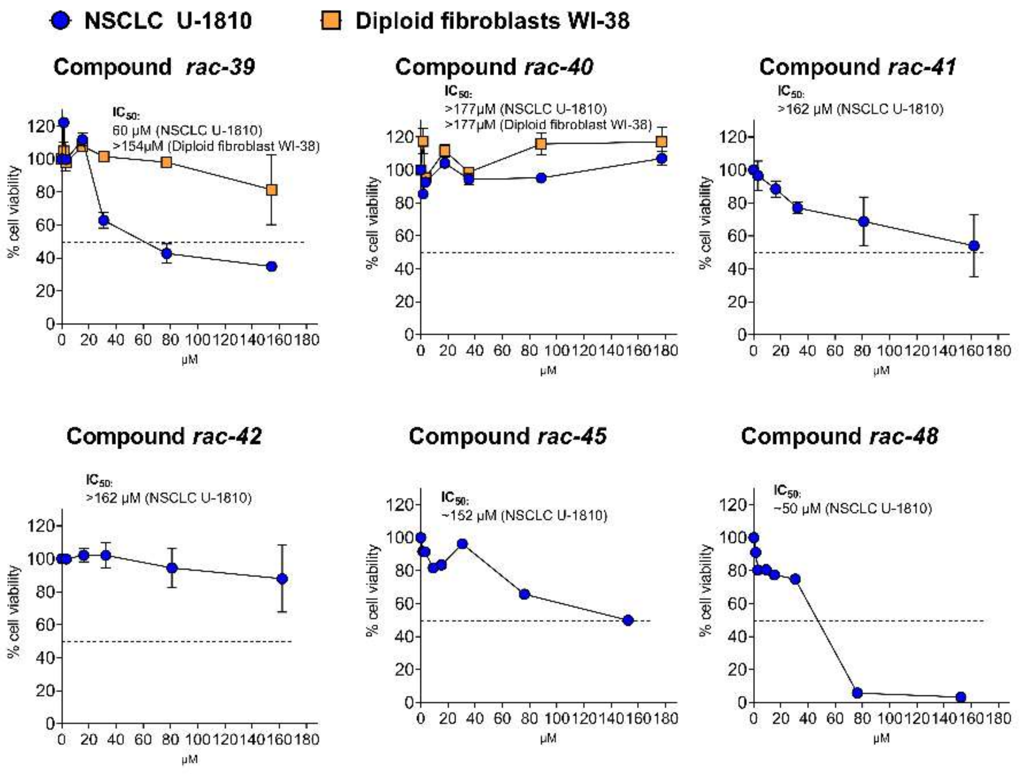
NSCLC U-1810
Diploid fibroblasts WI-38
Compound *rac-39*
IC50:
60 μM (NSCLC U-1810)
>154μM (Diploid fibroblast WI-38)
Compound *rac-40*
IC50:
>177μM (NSCLC U-1810)
>177μM (Diploid fibroblast WI-38)
Compound *rac-41*
IC50:
>162 μM (NSCLC U-1810)
Compound *rac-42*
IC50:
>162 μM (NSCLC U-1810)
Compound *rac-45*
IC50:
~152 μM (NSCLC U-1810)
Compound *rac-48*
IC50:
~50 μM (NSCLC U-1810)
% cell viability
μM

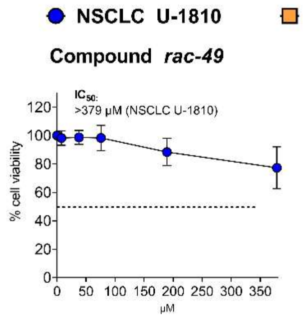
NSCLC U-1810
Diploid fibroblasts WI-38
Compound *rac-49*
IC50:
>379 μM (NSCLC U-1810)
% cell viability
μM
